# Structural Properties of Gas-Phase Molybdenum Oxide Clusters [Mo_4_O_13_]^2−^, [HMo_4_O_13_]^−^, and [CH_3_Mo_4_O_13_]^−^ Studied by Collision-Induced Dissociation

**DOI:** 10.1007/s13361-019-02294-4

**Published:** 2019-08-16

**Authors:** Manuel Plattner, Aristeidis Baloglou, Milan Ončák, Christian van der Linde, Martin K. Beyer

**Affiliations:** Institut für Ionenphysik und Angewandte Physik, Leopold-Franzens-Universität Innsbruck, Technikerstraße 25, 6020 Innsbruck, Austria

**Keywords:** Molybdenum oxide, Catalysis, Hydrogen evolution reaction, Protonation, Collision induced dissociation

## Abstract

Molybdenum oxide-based catalysts are widely used for the ammoxidation of toluene, methanation of CO, or hydrodeoxygenation. As a first step towards a gas-phase model system, we investigate here structural properties of mass-selected [Mo_4_O_13_]^2−^, [HMo_4_O_13_]^−^, and [CH_3_Mo_4_O_13_]^−^ by a combination of collision-induced dissociation (CID) experiments and quantum chemical calculations. According to calculations, the common structural motif is an eight-membered ring composed of four MoO_2_ units and four O atoms. The 13th O atom is located above the center of the ring and connects two to four Mo centers. For [Mo_4_O_13_]^2−^ and [HMo_4_O_13_]^−^, dissociation requires opening or rearrangement of the ring structure, which is quite facile for the doubly charged [Mo_4_O_13_]^2−^, but energetically more demanding for [HMo_4_O_13_]^−^. In the latter case, the hydrogen atom is found to stay preferentially with the negatively charged fragments [HMo_2_O_7_]^−^ or [HMoO_4_]^−^. The doubly charged species [Mo_4_O_13_]^2−^ loses one MoO_3_ unit at low energies while Coulomb explosion into the complementary fragments [Mo_2_O_6_]^−^ and [Mo_2_O_7_]^−^ dominates at elevated collision energies. [CH_3_Mo_4_O_13_]^−^ affords rearrangements of the methyl group with low barriers, preferentially eliminating formaldehyde, while the ring structure remains intact. [CH_3_Mo_4_O_13_]^−^ also reacts efficiently with water, leading to methanol or formaldehyde elimination.

**Electronic supplementary material:**

The online version of this article (10.1007/s13361-019-02294-4) contains supplementary material, which is available to authorized users.

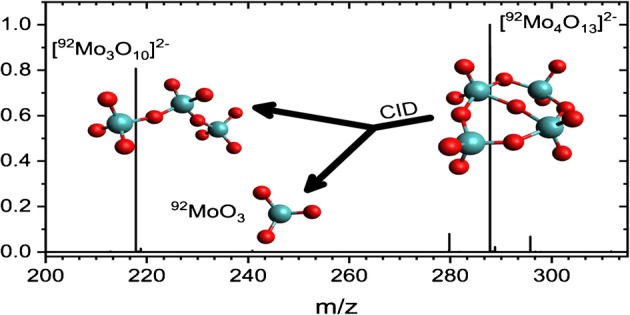

## Introduction

Molybdenum oxide-based catalysts are widely applicable in heterogeneous catalysis, e.g., for ammoxidation of toluene [1], hydrodeoxygenation [2, 3], or sulfur-resistant CO methanation [4]. In situ investigation of surface catalysis leads to a high experimental and computational complexity. For this reason, it is instructive to study well-defined systems, such as gas-phase clusters, to identify reactive intermediates and to understand the intrinsic reactivity [5–9]. Reliable thermochemical data on small clusters are available from the guided ion beam studies of the Armentrout laboratory [5], e.g., for molybdenum oxide [10, 11] and molybdenum sulfide [12] decomposition. Matrix isolation infrared absorption spectroscopy [13], collision-induced dissociation [14], and reactivity studies towards small, organic molecules [15–20] revealed further properties of molybdenum oxide clusters. The Schwarz group even achieved methane activation by MoO_3_^+^ and OMoH^+^ [21, 22]. Nucleation and formation-fragmentation mechanisms of anionic polyoxomolybdates were studied with electrospray ionization mass spectrometry by Cronin, Poblet, and co-workers [23, 24]. Photoelectron spectroscopy of doubly and singly charged Cr, Mo, W dimetalate anions by Wang and co-workers gives insight into the electronic and geometric structure of the corresponding dimolybdates [25]. Furthermore, Waters, O’Hair, and Wedd studied gas-phase catalytic cycles involving molybdates for the oxidation of methanol and the dehydration of acetic acid [26, 27].

Combined with computational methods, i.e., DFT calculations, the studies by Raghavachari, Jarrold, and co-workers map elementary steps with potential relevance for hydrogen evolution reaction (HER) catalysis on molybdenum oxide and sulfide [28, 29]. However, HER with molybdenum oxides lead to highly oxidized, mostly saturated clusters whereby it is necessary to reduce the cluster in a second step to complete the catalytic cycle [29]. Ray et al. calculated a possible cycle with a [Mo_2_O_4_]^−^/[Mo_2_O_5_]^−^ cluster couple and C_2_H_4_ to reduce the cluster after H_2_ production [29]. Due to this multistep process, subsequent barriers of up to 2 eV are present [29]. Later experimental investigations, however, indicate that these barriers can be overcome along the reaction path, providing indirect evidence for the theoretically found catalytic cycle [17]. On the experimental side, Topolski et al. directly investigated the reactivity of water with molybdenum sulfides and oxosulfides and verified energetic as well as structural similarities of these reactions with the corresponding oxides [28]. Only for molybdenum sulfides, however, barrier-free hydrogen producing reaction pathways could be found. For the oxosulfide species, the molybdenum atom bound to oxygen was calculated to be the reactive site for hydrogen evolution. A fundamental understanding of the relevant processes may facilitate targeted HER-catalyst optimization. To this end, we have recently studied the reactivity of molybdenum clusters with dimethyl disulfide [30] as well as the decomposition pathways of molybdenum sulfide clusters [Mo_3_S_13_]^2−^, [HMo_3_S_13_]^−^, and [H_3_Mo_3_S_13_]^+^ [31].

Here, we study the properties of the pure [Mo_4_O_13_]^2−^ cluster as well as its protonated [HMo_4_O_13_]^−^ and methylated [CH_3_Mo_4_O_13_]^−^ forms in the gas phase using mass spectrometry. In particular, we investigate the dissociation pathways of the clusters in the hexapole collision cell of a Fourier transform ion cyclotron resonance mass spectrometer (FT-ICR MS), which is well suited for studying clusters in the gas phase [30, 32–36] as well as investigating catalytic cycles [32, 34, 35, 37, 38]. We show that the precursor ions have closed-ring structures and that the stability of protonated [HMo_4_O_13_]^−^ against collisions is strongly enhanced compared to the pure [Mo_4_O_13_]^2−^ cluster. This could be attributed to hydrogen roaming prior to any dissociation, as suggested by theoretical calculations. Furthermore, we observe formaldehyde elimination by collisional activation of methylated [CH_3_Mo_4_O_13_]^−^, which is a key step in catalytic methanol oxidation, as shown previously by Waters et al. [26].

## Experimental and Computational Details

The experimental setup consists of a Bruker Apex Qe FT-ICR Mass Spectrometer, which is equipped with a 9.4 T superconducting magnet and a combined electrospray ionization (ESI) and matrix-assisted laser desorption ionization (MALDI) Apollo II Dual ESI/MALDI ion source. The instrument features a hexapol collision cell for collision-induced dissociation experiments. See a previous publication for details [39].

Cluster ions in the gas phase are obtained via electrospray ionization (ESI) of a 3 mM solution of isotopically enriched ammonium heptamolybdate (NH_4_)_6_[^92^Mo_7_O_24_], > 99.9% ^92^Mo, in water-methanol (1:1). (NH_4_)_6_[^92^Mo_7_O_24_]·4H_2_O was prepared by dissolving 3.3 mg ^92^MoO_3_ (STB Isotope Germany) in 1 mL ammonium hydroxide solution (5.0 M, Honeywell Research Chemicals) and left to dry under air until colorless crystals were obtained. Subsequently, a stock solution was prepared by dissolving the crystals in 8 mL water-methanol mixture (1:1). Since molybdenum oxide clusters with natural isotope distribution have many isotopologues, we used enriched ^92^MoO_3_ to disentangle the corresponding mass spectra and to improve the signal-to-noise ratio (see Figure S1). Electrospray ionization of the heptamolybdate solution yields predominantly the ions of interest, namely [Mo_4_O_13_]^2−^, [HMo_4_O_13_]^−^, or [CH_3_Mo_4_O_13_]^−^. The presence of [HMo_4_O_13_]^−^ is not surprising since protonation of molybdenum oxides in the presence of alcohols is known [16]. On the other hand, the observation of [CH_3_Mo_4_O_13_]^−^ is noticeable. As shown by O’Hair and co-workers, the reaction of [Mo_2_O_6_(OH)]^−^ with methanol in the gas phase leads to [Mo_2_O_6_(OCH_3_)]^−^ and elimination of water [26]. We assume that the analogous reaction of [HMo_4_O_13_]^−^ with CH_3_OH to from [CH_3_Mo_4_O_13_]^−^ takes place in the electrospray ionization source, either in the desolvation capillary or in the region following the capillary exit, where methanol vapor is abundant.

Each precursor ion is mass selected with the quadrupole mass filter and trapped in the hexapole collision cell (*p* ≈ 10^−3^ mbar) for a collision time of *t*_coll_ = 0.8 s. During this time, the ions are excited by a radio frequency signal of variable amplitude, which allows for a qualitative tuning of the collision energies. The fragments are then cooled and trapped by subsequent collisions with the argon buffer gas. Although high-purity argon (99.999%) is used, traces of water from the ESI solution or from air in the gas inlet system can lead to reactive collisions, as discussed in detail below. The collision energy of the trapped ions with the background gas correlates with the bias voltage *V*_*c*_ of the collision cell. After fragmentation, the ions are electrostatically guided into the ICR cell, where a high-resolution mass spectrum is acquired. By scanning the collision voltage *V*_*c*_ and recording a mass spectrum for each set value, breakdown curves of the ion of interest can be recorded. Unfortunately, quantitative threshold energies cannot be derived from this measurement. It is worth mentioning that the instrument used for the employed CID technique is different from other types of multistage MS instruments like, e.g., the Guided Ion Beam studies of Armentrout used for energy resolved CID [40]. Since low-mass ions are discriminated in our setup due to the time-of-flight between the hexapole collision cell and the ICR cell, only ions with *m*/*z* > 57.75 were detected. Only fragment ions reaching a relative intensity of at least 0.1% in any CID spectrum were considered in the analysis.

In order to comprehend the observed dissociation pathways, quantum chemical computations are necessary. We model the structure of the precursor ions and dissociation products and, based on relative energies, assess the most probable reaction pathways. The respective dissociation energies provide the lower limit of the energy barrier along the dissociation coordinate. For charge reduction reactions, i.e., dissociation of [Mo_4_O_13_]^2−^ into two singly charged fragments, the reverse Coulomb barrier leads to a significant increase of the overall barrier towards dissociation [41, 42]. To gain further insight into the structural flexibility of the clusters, we also performed transition state calculations for transfer reactions of H and CH_3_ on the Mo_4_O_13_ ion.

Density functional theory (DFT) calculations were performed for all observed ions and their neutral counterparts. For each molecule, different initial structures were chosen based on chemical intuition and previous studies [15, 24, 29, 43–46]. The two lowest spin multiplicities were considered (higher spin multiplicity states were considered for selected structures to verify that they lie higher in energy). For most ions, the lowest multiplicity state turned out to be the most stable one, which is in contrast to other calculations for small Cr group oxides with high oxygen to metal ratio [13]. Although the ωB97XD functional was reported to provide reliable results for second-row transition metals [47], we used additionally the B3LYP and the M06 functionals to estimate the error of the calculated energies and possible structure variation; the def2TZVP basis set was used. Benchmarking with respect to decomposition of small molybdenum oxide and sulfide clusters shows that B3LYP provides the best results among the applied functionals (Table S3). Benchmarking calculations with respect to the vertical electron detachment energies of [Mo_2_O_7_]^−^ and [Mo_2_O_7_]^2−^, however, suggest the M06 functional to be the most reliable (see Table S4). Stability of the wave function was tested within all calculations. Transition states were identified by frequency and intrinsic reaction coordinate (IRC) calculations. Charge analysis was performed using the CHELPG method [48], with the radius of Mo chosen to be 1.7 Å. The reported energies include zero-point corrections. Calculations were performed using the Gaussian program [49].

## Results and Discussion

**Figure 1:**
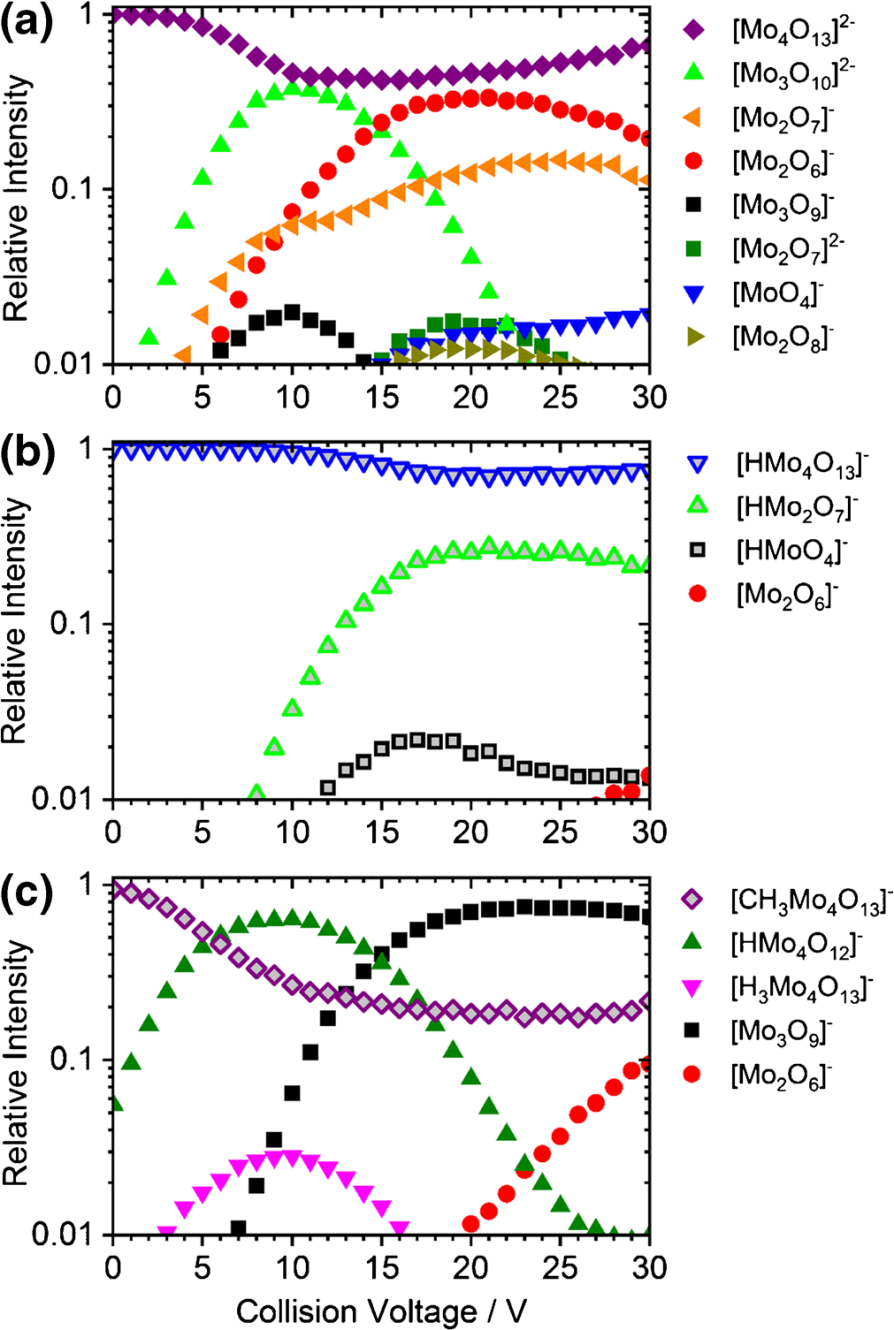
CID breakdown curves of the [Mo_4_O_13_]^2−^ (**a**), [HMo_4_O_13_]^−^ (**b**), and [CH_3_Mo_4_O_13_]^−^ (**c**) clusters. Only ions with relative intensities higher than 1% are shown. See Figure S2 for breakdown curves including all fragment ions down to the noise level

**Figure 2:**
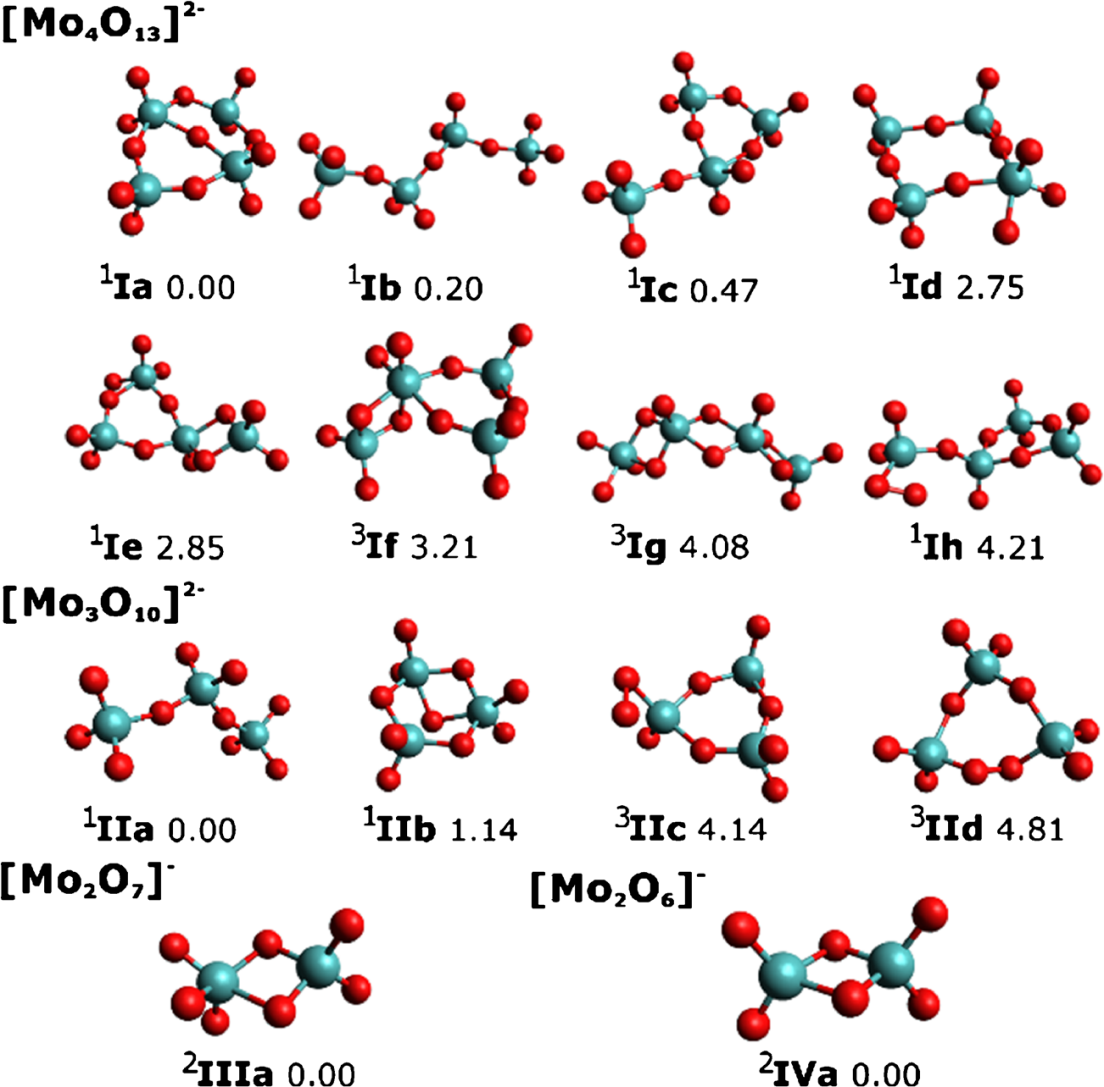
Structure of the [Mo_4_O_13_]^2−^ cluster and its selected fragments, with spin multiplicity given in superscript next to each isomer. Calculated at the ωB97XD/def2TZVP level of theory, relative energy is given in eV

**Figure 3:**
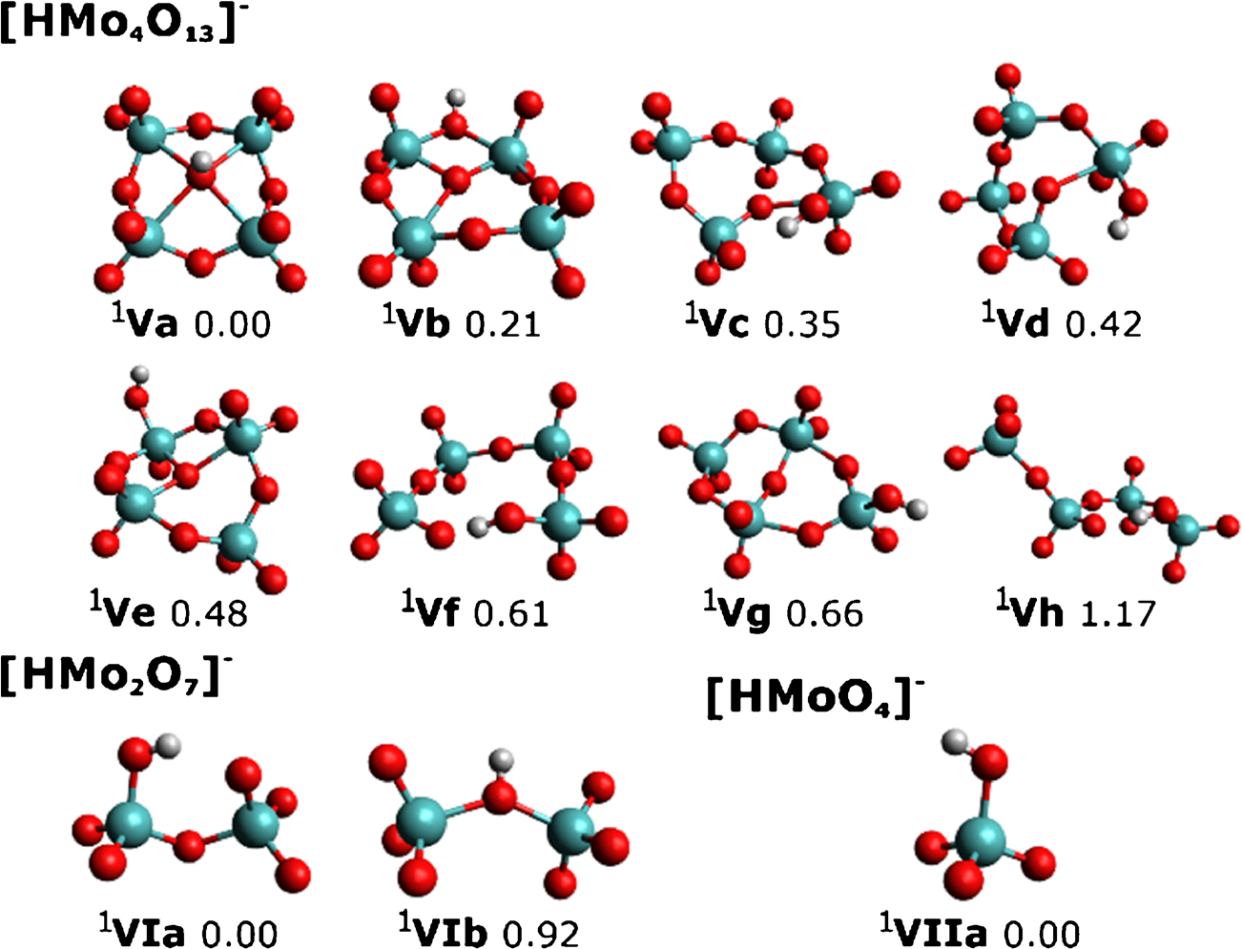
Structure of the [HMo_4_O_13_]^−^ cluster and its selected fragments, with spin multiplicity given in superscript next to each isomer. Calculated at the ωB97XD/def2TZVP level of theory, relative energy is given in eV

**Figure 4:**
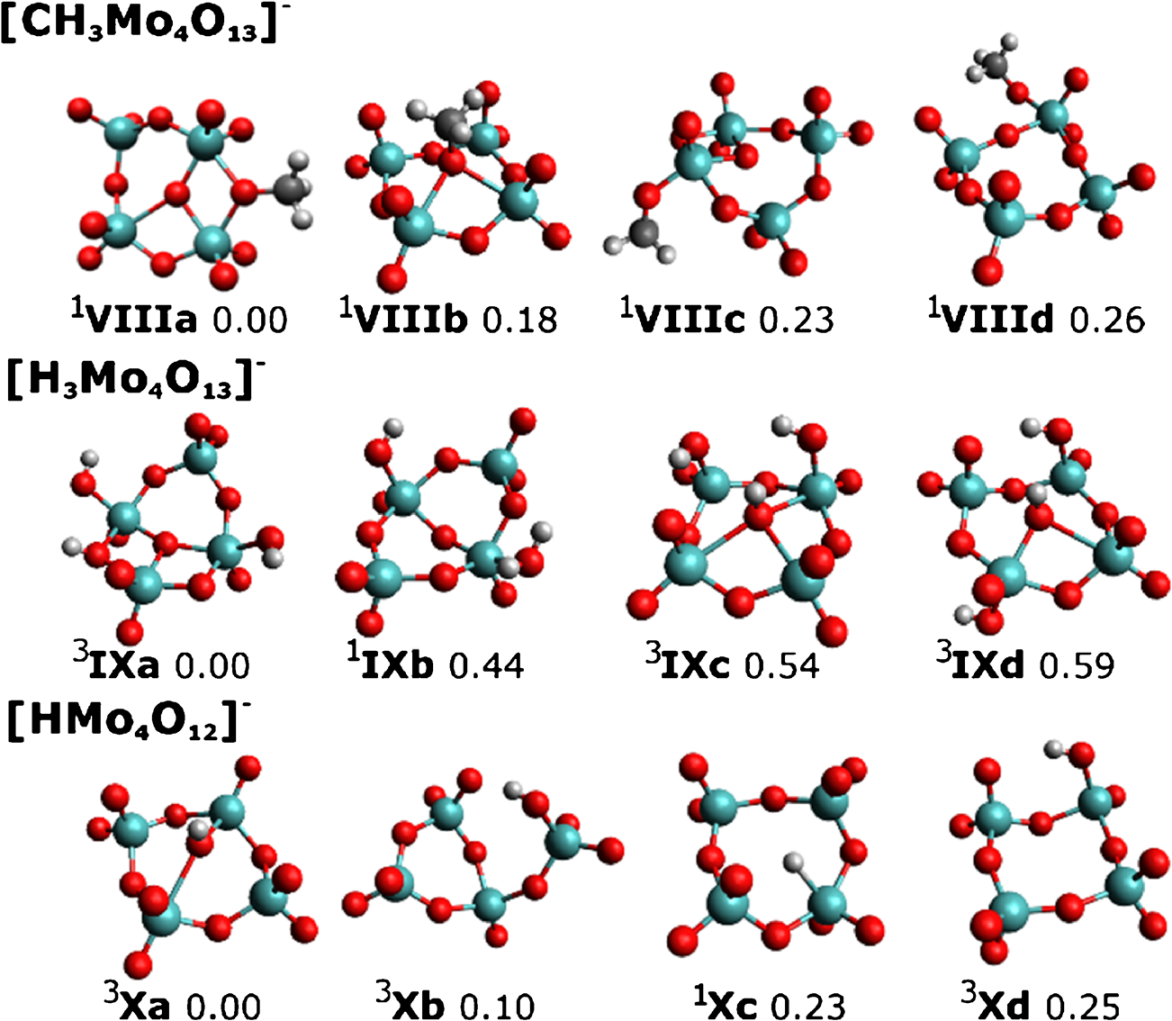
Structure of the [CH_3_Mo_4_O_13_]^−^ cluster and its selected fragments, with spin multiplicity given in superscript next to each isomer. Calculated at the ωB97XD/def2TZVP level of theory, relative energy is given in eV

Breakdown curves of [Mo_4_O_13_]^2−^, [HMo_4_O_13_]^−^, and [CH_3_Mo_4_O_13_]^−^ are provided in Figure 1a–c. For the sake of clarity and due to the large number of fragments, only ions exceeding a relative intensity of 1% are displayed (see Figure S2 for complete CID breakdown curves). Selected molecular structures are shown in Figures 2, 3, and 4. Calculated structures and energies of all observed molecules are listed in the Supporting Information (SI).

### Dissociation Patterns of [Mo_4_O_13_]^2−^

The breakdown curves of [Mo_4_O_13_]^2−^ are shown in Figure 1a. Fragments containing three and two molybdenum atoms are formed at low energies. The trimolybdate species undergo further dissociation at higher energies. At about *V*_*c*_ ≈ 15 V, fragmentation reaches a maximum of 58%. The precursor ion dissociates mainly to [Mo_3_O_10_]^2−^, with a branching ratio of 37% at *V*_*c*_ ≈ 10 V. In the mass spectrum, [HMo_2_O_7_]^−^ was also found, which cannot originate from dissociation of [Mo_4_O_13_]^2−^. We assign [HMo_2_O_7_]^−^ as a product of collisions with water, due to traces of air in the collision cell or solvent from the ESI source. It should be noted that in the collision cell, the collision rate is on the order of 10^5^ s^−1^, i.e., very minor amounts of water are sufficient to cause these signals.

Calculated dissociation energies for all observed channels of [Mo_4_O_13_]^2−^ are listed in Table 1. Comparing the energies calculated using different functionals leads to a mean standard deviation of 0.22 eV, and the relative order of the dissociation channels is independent of the functional. The given values constitute the energy difference of the lowest local minima found for the precursor ion and the fragments. Therefore, the energies represent also a lower limit of the energy barrier to overcome during fragmentation. For singly charged fragments, however, the reverse Coulomb barrier must be added, since Coulomb explosion is associated with significant kinetic energy release [41, 42, 50–52]. We estimated the barrier based on a point-charge separation of 4.1 Å at the transition state of Coulomb explosion, which corresponds to the molecule diameter. This leads to a potential energy of 3.5 eV, representing a rough approximation for the reverse Coulomb barrier, which must be added to the energy of the fragments at infinite separation.

**Table 1:** Dissociation Energy (in eV) of Fragmentation Channels of [Mo_4_O_13_]^2−^ Observed in the Experiment, along with Dissociated Molecules used to Calculate the Dissociation Energy. Calculated Using the ωB97XD, B3LYP, and M06 Functionals along with the def2TZVP Basis Set. All Listed Ions were Observed during the CID Experiment unless Indicated Otherwise

	Precursor		Fragment 1		Fragment 2		Fragment 3	*E*_ωB97XD_	*E*_B3LYP_	*E*_M06_
1.1	[Mo_4_O_13_]^2−^	**→**	[Mo_3_O_10_]^2−^	+	[MoO_3_]			4.29	3.94	4.37
1.2	[Mo_4_O_13_]^2−^	**→**	[Mo_2_O_7_]^2−^	+	[MoO_3_]	+	[MoO_3_]	9.05	8.53	8.98
1.2’	[Mo_4_O_13_]^2−^	**→**	[Mo_2_O_7_]^2−^	+	[Mo_2_O_6_]			4.22	3.92	4.31
1.3	[Mo_4_O_13_]^2−^	**→**	[Mo_2_O_7_]^−^	+	[Mo_2_O_6_]^−^			2.57	2.14	2.41
1.4	[Mo_4_O_13_]^2−^	**→**	[Mo_2_O_6_]^−^	+	[MoO_4_]^−^	+	[MoO_3_]	6.41	5.78	6.25
1.5	[Mo_4_O_13_]^2−^	**→**	[Mo_2_O_7_]^−^	+	[MoO_3_]^−^	+	[MoO_3_]	7.62	7.00	7.50
1.6	[Mo_4_O_13_]^2−^	**→**	[Mo_3_O_9_]^−^	+	[MoO_4_]^−^			2.06	1.70	2.14
1.7	[Mo_4_O_13_]^2−^	**→**	[Mo_3_O_10_]^−^	+	[MoO_3_]^−^			3.75	3.41	3.68
1.8	[Mo_4_O_13_]^2−^	**→**	[Mo_3_O_10_]^−^	+	[MoO_3_]	+	e^−^	6.84	6.62	6.65
1.9	[Mo_4_O_13_]^2−^	**→**	[Mo_2_O_8_]^−^	+	[Mo_2_O_5_]^−a^			6.54	6.12	6.34
1.10	[Mo_4_O_13_]^2−^ + O_2_	**→**	[Mo_2_O_8_]^−^	+	[Mo_2_O_7_]^−^			1.73	1.33	1.55
1.11	[Mo_4_O_13_]^2−^ + H_2_O	**→**	[HMo_2_O_7_]^−^	+	[HMo_2_O_7_]^−^			− 1.18	− 1.33	− 1.14

^a^[Mo_2_O_5_]^−^ was not observed in the experiment

The most stable isomer of [Mo_4_O_13_]^2−^ at the ωB97XD level has a cyclic structure with one bridging oxygen atom, see isomer **Ia** in Figure 2, in agreement with the structure found on the BP86/TZP level by Vilà-Nadal et al. [24]. Two isomers (**Ib**, **Ic**) with more open structures are predicted to lie within 0.5 eV. At the B3LYP level, calculations even predict the open structure **Ib** to be the most stable isomer. The observed loss of MoO_3_, reaction (1.1) in Table 1, already at nominally *V*_*c*_ = 0 V, indicates that this moiety is weakly bound and requires ring-opening prior to dissociation. When the energy of the system increases during the multi-collision CID in the hexapole collision cell, isomerization to **Ib** or **Ic** prior to dissociation is very likely. Entropy will favor the more flexible, lower-lying isomer **Ib**, which can be expected to dominate at elevated energies. All employed DFT functionals (ωB97XD, B3LYP, M06) predict the lowest energy structure for [Mo_3_O_10_]^2−^ to be the linear isomer **IIa**, in agreement with previous calculations [24], further indicating that ring opening takes place. At elevated energies, two MoO_3_ units may be lost from [Mo_4_O_13_]^2−^, leading to [Mo_2_O_7_]^2−^, reaction (1.2). Alternatively, an intact Mo_2_O_6_ molecule may be lost, reaction (1.2′), but this neutral product would not explain the high appearance energy of this ion.

Most fragments are singly charged, and assignment to specific dissociation channels is not straightforward in every case. During Coulomb explosion, low-mass-charged fragments gain most of the kinetic energy release and may be lost from the hexapole trap. Loss of fragments due to their high kinetic energy is also the most plausible reason for the increasing relative intensity of the precursor ion from 15 to 30 V collision voltage. Electron detachment may occur from hot doubly charged fragments, possibly with the contribution of tunneling [53]. Both effects disturb the quantitative evaluation of the ion signal and may explain the apparent increase of the precursor ion signal at higher energies. The dominant charge reduction channel is dissociation into [Mo_2_O_7_]^−^ and [Mo_2_O_6_]^−^, reaction (1.3). At elevated energies, both ions may lose MoO_3_, resulting in [MoO_4_]^−^ and [MoO_3_]^−^, reactions (1.4) and (1.5), respectively. [MoO_4_]^−^ can be also directly formed from [Mo_4_O_13_]^2−^, together with [Mo_3_O_9_]^−^, reaction (1.6). However, it is not observed in the corresponding intensity, suggesting that some [MoO_4_]^−^ ions are lost due to excessive kinetic energy release as observed before in CID of [Mo_3_S_13_]^2−^ [31]. Traces of [Mo_3_O_10_]^−^ are observed, which may result from Coulomb explosion with concomitant formation of [MoO_3_]^−^, reaction (1.7). Alternatively, electron detachment may occur, reaction (1.8).

Dissociation to produce [Mo_3_O_10_]^2−^, the most abundant fragment ion in Figure 1a requires about 4 eV (Table 1). [Mo_2_O_7_]^−^ and [Mo_2_O_6_]^−^ are complementary fragments and result from the dissociation reaction (1.3), most likely from isomer **Ib**. For higher collision energies, we observe more [Mo_2_O_6_]^−^ than [Mo_2_O_7_]^−^. Formation of [Mo_3_O_9_]^−^ is calculated to be the channel with the lowest dissociation energy, reaction (1.6). However, we observed this fragmentation only at a maximum relative intensity of about 2%. The cyclic [Mo_3_O_9_]^−^ results most likely from dissociation of isomer **Ic**, which is significantly less populated than isomer **Ib**, explaining the low intensity of reaction (1.6). Further loss of MoO_2_, resulting in [Mo_2_O_7_]^−^, would also explain both: the low overall abundance and the decrease at higher energies accompanied with increasing [Mo_2_O_7_]^−^ signal. The calculations predict a minimum energy of 9.21 eV for this two-step process, therefore making reactions (1.3) and (1.5) more plausible.

The calculated dissociation energy of 6.54 eV to form [Mo_2_O_8_]^−^, reaction (1.9), is the highest among all calculated energies for simple dissociations; the ion is produced only in a small yield, slightly above 1%. However, we do not observe [Mo_2_O_5_]^−^ formed via eq. (1.9) (Table 1 and Figure S2), and complete loss of this ion from the collision cell is not very likely. Interestingly, the intensity of [Mo_2_O_8_]^−^ is parallel to the [Mo_2_O_6_]^−^ signal, which suggests that [Mo_2_O_8_]^−^ is rather formed in collisions with O_2_, reaction (1.10). Along the same lines, the [HMo_2_O_7_]^−^ fragment is most likely formed in collisions with H_2_O, reaction (1.11).

Compared to our recent work on thiomolybdates [31], the observed dissociation patterns differ considerably. In the case of thiomolybdates, S_2_ ligands are present on the cluster surface, which are lost preferentially. In the present case, no O_2_ moieties are present on the cluster surface (see Figure 2). Dissociation of MoO_3_ as a thermodynamically stable entity takes place instead.

### Dissociation Patterns of [HMo_4_O_13_]^−^

Breakdown curves of [HMo_4_O_13_]^−^ are shown in Figures 1b and S2. There are only four fragments for the protonated form, and only two of them exceed 1% relative intensity at collision voltages up to 20 V, where about 30% of the precursor ions dissociate. Upon comparing the dissociation patterns of singly and doubly charged ions, one has to consider that doubly charged ions experience twice the acceleration of singly charged ions of the same mass in the electric field. Even then, however, this indicates a higher stability of the protonated ions, as also observed for thiomolybdates [31].

The observed fragments together with plausible neutral products are listed in Table 2. The two protonated fragments, [HMo_2_O_7_]^−^ and [HMoO_4_]^−^, are dominant and arise at low collision energies, reactions (2.1) and (2.2). According to the calculations, reaction (2.2) requires slightly less energy than (2.1), with Mo_3_O_9_ formed as a neutral product. In the experiment, the breakdown curves of (2.1) and (2.2) behave parallel, which is consistent with their similar threshold energy. However, reaction (2.2) with the lower threshold is significantly less probable. This can be explained by the required reorganization of the [HMo_4_O_13_]^−^ precursor. As shown in Figure S3.33, the lowest energy structure of Mo_3_O_9_ features a six-membered ring of alternating Mo and O atoms, while dissociation from a ring-opened precursor ion would result in isomer II of Mo_3_O_9_, which lies 1.38 eV higher in energy. Consequently, reaction (2.2) requires reorganization to one of the isomers X, XI, or XIII of [HMo_4_O_13_]^−^, see Figure S3.21, which lie 1.3–1.4 eV above the minimum. The ring-opened structure VI, which affords dissociation into the minimum geometry of [HMo_2_O_7_]^−^, lies at 0.61 eV and is thus much more populated. Overall, the population of the precursor prior to dissociation decides which product ion is formed.

**Table 2:** Dissociation Energy *E* (in eV) of Fragmentation Channels of [HMo_4_O_13_]^−^ Observed in the Experiment, along with Dissociated Molecules used to Calculate the Dissociation Energy. Calculated using the ωB97XD, B3LYP, and M06 Functionals along with the def2TZVP Basis Set. All Listed Ions were Observed during the CID Experiment

	Precursor		Ions		Neutrals	*E*_ωB97XD_	*E*_B3LYP_	*E*_M06_
2.1	[HMo_4_O_13_]^−^	**→**	[HMo_2_O_7_]^−^	+	Mo_2_O_6_	3.43	2.95	3.49
2.2	[HMo_4_O_13_]^−^	**→**	[HMoO_4_]^−^	+	Mo_3_O_9_	3.13	2.73	3.29
2.3	[HMo_4_O_13_]^−^	**→**	[Mo_2_O_6_]^−^	+	HMo_2_O_7_	5.65	4.96	5.67
2.4	[HMo_4_O_13_]^−^	**→**	[Mo_3_O_9_]^−^	+	HMoO_4_	4.61	4.03	4.74

At higher energies, fragments with the same stoichiometry are formed, but the charge stays at the former neutral product, reactions (2.3) and (2.4). [Mo_3_O_9_]^−^ is formed with an overall relative intensity below 0.2% (see Figure S2) with the simultaneous decrease of the [HMoO_4_]^−^ signal at *V*_*c*_ = 19 V. Therefore, it seems entirely plausible that Mo_3_O_9_ is formed in reaction (2.2), and at higher energy has a slim chance to scavenge the electron, in line with the reorganization of [HMo_4_O_13_]^−^ discussed above.

The most stable structure of the protonated form (isomer **Va** in Figure 3) is similar to the lowest energy structure of [Mo_4_O_13_]^2−^ (isomer **Ia**), with the proton bound to the central oxygen atom. This is consistent with the central oxygen atom in **Ia** carrying the most negative partial charge (− 0.97 e) as calculated within the CHELPG population analysis. For the five most stable isomers found, only the protonation site is different. The flexibility in protonation could be the reason for the lower fragmentation yield observed, as the cluster is able to use the available energy to mobilize the proton, without disrupting the Mo-O cage. The energy difference between different protonation sites lies within 0.5 eV; ωB97XD/def2TZVP transition state calculations between isomers **Va**,**b,e** predict that 0.9–1.4 eV is necessary for intramolecular proton transfer, Table 3. Thus, hydrogen roaming would take place before any dissociation.

**Table 3:** Energy Barriers (eV) between Different Isomers of [HMo_4_O_13_]^−^ and [CH_3_Mo_4_O_13_]^−^, Relative to the Lowest Energy Isomer. Calculated at the ωB97XD/def2TZVP Level of Theory

Ion	Isomer 1		Isomer2	*E*
[HMo_4_O_13_]^−^	**Va**	**→**	**Vb**	1.44
	**Va**	**→**	**Ve**	0.94
	**Vb**	**→**	**Ve**	1.30
[CH_3_Mo_4_O_13_]^−^	**VIIIa**	**→**	**VIIIb**	3.22
	**VIIIa**	**→**	**VIIIc**	3.17
	**VIIIb**	**→**	**VIIIc**	2.94

### Dissociation Patterns of [CH_3_Mo_4_O_13_]^−^

Breakdown curves for [CH_3_Mo_4_O_13_]^−^ are shown in Figure 1c. Fragmentation yield reaches a maximum of 83%, far exceeding the CID ratios of the other investigated species under similar conditions. In [CH_3_Mo_4_O_13_]^−^, the methyl group is predicted to be on a peripheral bridging oxygen atom (structure **VIIIa** in Figure 4), different from the preferred protonation site at the central oxygen atom. Three other structures with the methyl group connected to different O atoms lie, however, within 0.3 eV.

The most efficient fragmentation channel is formaldehyde loss, forming [HMo_4_O_12_]^−^, the dominant ion at low energies, reaction (3.1) in Table 4. Two more ions behave exactly parallel, but with lower abundance, [H_3_Mo_4_O_13_]^−^ and [HMo_4_O_13_]^−^, the latter one only visible in Figure S2. For stoichiometric reasons, these ions are best explained if one invokes collisions with H_2_O, enabling release of neutral CH_2_O and CH_3_OH, reactions (3.2) and (3.3), respectively. All these reactions exhibit reaction energies below 2 eV, consistent with the observed early onset of the fragmentation. The alternative to methanol elimination, subsequent elimination of H_2_ from [H_3_Mo_4_O_13_]^−^, is mechanistically very demanding, with a barrier of 3.2 eV (ωB97XD/def2TZVP), and therefore not realistic. [H_3_Mo_4_O_13_]^−^, structure **IXa**, with protons positioned on the peripheral oxygen atoms, is calculated to be a biradical (see SI). The alternative isomer with H_2_O as a ligand, **IXb**, lies about 0.5 eV higher in energy. At higher energies, [HMo_2_O_6_]^−^ is observed (Figure S2), most likely formed together with Mo_2_O_6_ as fragments of the initially dominant [HMo_4_O_12_]^−^, reaction (3.4).

**Table 4:** Dissociation Energy *E* (in eV) of Fragmentation Channels of [CH_3_Mo_4_O_13_]^−^ Observed in the Experiment, along with Dissociated Molecules used to Calculate the Dissociation Energy. Calculated using the ωB97XD, B3LYP and M06 Functionals along with the def2TZVP Basis Set

	Precursor				Ions		Neutrals	*E*_ωB97XD_	*E*_B3LYP_	*E*_M06_
3.1	[CH_3_Mo_4_O_13_]^−^			→	[HMo_4_O_12_]^−^	+	CH_2_O	1.89	1.70	1.84
3.2	[CH_3_Mo_4_O_13_]^−^	+	H_2_O	→	[H_3_Mo_4_O_13_]^−^	+	CH_2_O	0.50	0.50	0.55
3.2’	[CH_3_Mo_4_O_13_]^−^	+	H_2_O	→	[HMo_4_O_12_]^−^.H_2_O	+	CH_2_O	0.94	0.86	0.94
3.3	[CH_3_Mo_4_O_13_]^−^	+	H_2_O	→	[HMo_4_O_13_]^−^	+	CH_3_OH	− 0.03	− 0.04	− 0.09
3.4	[CH_3_Mo_4_O_13_]^−^			→	[HMo_2_O_6_]^−^	+	CH_2_O + 2MoO_3_	10.05	9.22	9.76
3.4’	[CH_3_Mo_4_O_13_]^−^			→	[HMo_2_O_6_]^−^	+	CH_2_O + Mo_2_O_6_	5.22	4.62	5.08
3.5	[CH_3_Mo_4_O_13_]^−^			→	[Mo_3_O_9_]^−^	+	CH_3_MoO_4_	4.44	3.85	4.51
3.5’	[CH_3_Mo_4_O_13_]^−^			→	[Mo_3_O_9_]^−^	+	CH_2_O + HMoO_3_	2.64	2.03	2.59
3.6	[CH_3_Mo_4_O_13_]^−^	+	O_2_	→	[Mo_3_O_11_]^−^	+	CH_3_MoO_4_	3.84	3.31	4.44
3.6’	[CH_3_Mo_4_O_13_]^−^	+	O_2_	→	[Mo_3_O_11_]^−^	+	CH_2_O + HMoO_3_	2.04	1.49	2.52
3.7	[CH_3_Mo_4_O_13_]^−^			→	[Mo_4_O_12_]^−^	+	CH_2_OH	2.80	2.37	3.03
3.8	[CH_3_Mo_4_O_13_]^−^			→	[Mo_2_O_6_]^−^	+	CH_3_Mo_2_O_7_	5.47	4.77	5.43
3.8’	[CH_3_Mo_4_O_13_]^−^			→	[Mo_2_O_6_]^−^	+	CH_2_O + HMo_2_O_6_	4.98	4.30	4.80
3.9	[CH_3_Mo_4_O_13_]^−^	+	O_2_	→	[Mo_2_O_8_]^−^	+	CH_3_Mo_2_O_7_	4.63	3.95	5.27
3.9’	[CH_3_Mo_4_O_13_]^−^	+	O_2_	→	[Mo_2_O_8_]^−^	+	CH_2_O + HMo_2_O_6_	4.14	3.49	4.64
3.10	[CH_3_Mo_4_O_13_]^−^			→	[CH_3_Mo_2_O_7_]^−^	+	2MoO_3_	8.17	7.47	8.04
3.10’	[CH_3_Mo_4_O_13_]^−^			→	[CH_3_Mo_2_O_7_]^−^	+	Mo_2_O_6_	3.34	2.87	3.36

The [Mo_3_O_9_]^−^ fragment is formed at slightly higher energies and quickly becomes the dominant product, with relative intensity up to 75%. The corresponding dissociation energy of CH_3_MoO_4_ was calculated to be relatively high, about 4 eV for reaction (3.5) in Table 4. Two-step fragmentation into formaldehyde and HMoO_3_ via [HMo_4_O_12_]^−^ as an intermediate **Xb** requires only ~ 2.5 eV, reaction (3.5′). Reaction (3.5) looks like the equivalent of reaction (2.4), but there does not seem to be a pronounced analogy to the fragmentation behavior of [HMo_4_O_13_]^−^; otherwise, a prominent [CH_3_MoO_4_]^−^ ion should be observed, which is not the case. The [Mo_3_O_11_]^−^ ion follows [Mo_3_O_9_]^−^ almost perfectly parallel (see Figure S2), but with two orders of magnitude lower intensity. This ion most likely arises from collisions with O_2_, reaction (3.6) or (3.6′), with the identical neutrals formed as in reaction (3.5) or (3.5′), respectively.

The loss of methoxy radical is indeed an option, as evidenced by the [Mo_4_O_12_]^−^ fragment, reaction (3.7). At even higher energies, [Mo_2_O_6_]^−^ is observed, formed through reaction (3.8), where the methoxy group may be bound to the neutral Mo_2_O_6_ moiety. Alternatively, the neutral fragments may be formaldehyde and HMo_2_O_6_, reaction (3.8′). In collisions with O_2_, [Mo_2_O_8_]^−^ is formed via reaction (3.9), with an intensity exactly parallel to [Mo_2_O_6_]^−^ (see Figure S2), but about a factor of 25 reduced intensity.

Analogous to (2.1), [CH_3_Mo_2_O_7_]^−^ is formed via reaction (3.10), but with intensity below 0.2%. This shows that the analogy of the methoxy species [CH_3_Mo_4_O_13_]^−^ and the corresponding hydroxy species [HMo_4_O_13_]^−^ in their fragmentation behavior is not at all pronounced. Compared to the proton mobility on the [HMo_4_O_13_]^−^ cluster, the barriers between [CH_3_Mo_4_O_13_]^−^ isomers with different positions of the CH_3_ group are much higher. Namely, isomers **VIIIa–c** are separated by barriers of 2.8–3.2 eV (see Table 3). Therefore, dissociation involving activation of the methyl group is more likely than methyl transfer, explaining the different fragmentation patterns of [CH_3_Mo_4_O_13_]^−^ and [HMo_4_O_13_]^−^.

Overall, the only detected carbon containing fragment ion was [CH_3_Mo_2_O_7_]^−^. This indicates that also the neutral counterparts might not contain carbon groups, but, for instance, dissociate into formaldehyde and a molybdenum oxide cluster with an additional H atom, which is reflected in the energetics of the product channels.

## Conclusions

From electrospray ionization of heptamolybdate dissolved in water-methanol (1:1), we obtained predominantly [Mo_4_O_13_]^2−^, [HMo_4_O_13_]^−^, and [CH_3_Mo_4_O_13_]^−^. As molybdenum oxides are protonated in the presence of alcohols [16], protonated [HMo_4_O_13_]^−^ is formed in solution. Presumably, this cluster reacts with CH_3_OH in the gas phase to form the methylated [CH_3_Mo_4_O_13_]^−^ under the elimination of H_2_O, analogous to the case of [Mo_2_O_6_(OH)]^−^ [26]. We performed CID experiments on [Mo_4_O_13_]^2−^, [HMo_4_O_13_]^−^, and [CH_3_Mo_4_O_13_]^−^ supported by theoretical calculations on all observed fragment ions and neutral counterparts. In agreement with literature, our calculations predict a similar ring structure for all three precursor ions, with a central oxygen atom as an energetically favorable location for protonation [24]. The calculations show that in [HMo_4_O_13_]^−^, or rather [Mo_4_O_12_(OH)]^−^, the proton becomes highly mobile prior to dissociation as the barriers were calculated to be around 1 eV. These intramolecular proton transfers are relevant for water activation at molybdenum oxide. In contrast to similar experiments with thiomolybdates [31], where pure S_*x*_ (*x* = 2–6) and H_*x*_S_*y*_ (*x* = 1–3, *y* = 2–6) neutral fragments dominate due to the presence of disulfide ligands, the investigated oxomolybdates do not possess dioxo ligands. Therefore, no pure oxygen dissociations were observed. In contrast, [CH_3_Mo_4_O_13_]^−^, or rather [Mo_4_O_12_(OCH_3_)]^−^, is more stable with the methyl group bound to one of the bridging oxygen atoms. Here, the presence of several isomers is expected, as the six lowest lying isomers are within 0.5 eV relative energy. Furthermore, we found that the most effective dissociation pathway for [Mo_4_O_12_(OCH_3_)]^−^ is the elimination of formaldehyde to form [Mo_4_O_11_(OH)]^−^. Presumed that subsequent oxidation of [Mo_4_O_11_(OH)]^−^ is possible, [Mo_4_O_12_(OH)]^−^ could be used to utilize catalytic oxidation of methanol to formaldehyde. This is consistent with the study of Waters et al. where formaldehyde elimination was observed on collisional activated [Mo_2_O_6_(OCH_3_)]^−^ [26].

## Electronic supplementary material

ESM 1 (PDF 1959 kb)
