# Supplementary material for: Structural Properties of Gas-Phase Molybdenum Oxide Clusters [Mo4O13]2−, [HMo4O13]−, and [CH3Mo4O13]− Studied by Collision-Induced Dissociation
Source: J Am Soc Mass Spectrom. 2019 Aug 16;30(10):1946–55. doi: 10.1007/s13361-019-02294-4 (PMC6805806; doi:10.1007/s13361-019-02294-4)
Supplement: Supplementary file 1 — (PDF 1959 kb) [file 13361_2019_2294_MOESM1_ESM.pdf]

Supporting Information

Structural Properties of Gas Phase Molybdenum Oxide Clusters $[Mo_4O_{13}]^{2-}$, $[HMo_4O_{13}]^-$, and $[CH_3Mo_4O_{13}]^-$ Studied by Collision-Induced Dissociation

*Manuel Plattner, Aristeidis Baloglou, Milan Ončák, Christian van der Linde, and Martin K. Beyer**

Institut für Ionenphysik und Angewandte Physik, Universität Innsbruck, Technikerstraße 25, 6020 Innsbruck, Austria

** E-mail: martin.beyer@uibk.ac.at*

Mass spectra of natural and isotopically enriched molybdate clusters

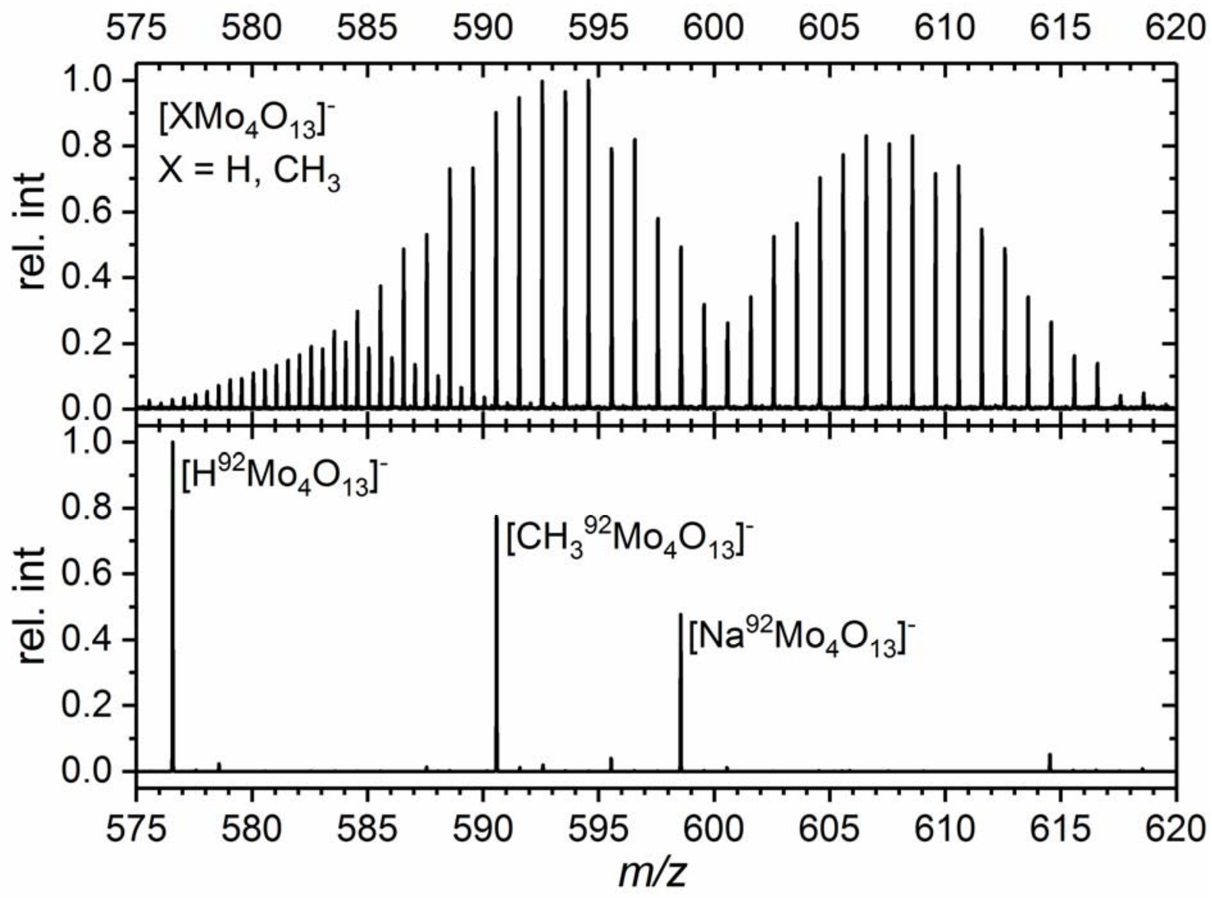

Figure S1: Isotopologues of natural tetramolybdate clusters (top) and the corresponding mass spectrum of the tetramolybdate clusters containing only isotopically enriched ^{92}Mo (bottom). Unambiguous identification of the mass peaks is only possible for the isotopically enriched case. The doubly charged isotope distribution in the lower mass range is $[Mo_8O_{25}]^{2-}$. The mass peak $[Na^{92}Mo_4O_{13}]^-$ is irrelevant for the present study and probably originates from Na^+ impurities inside the ESI inlet system.

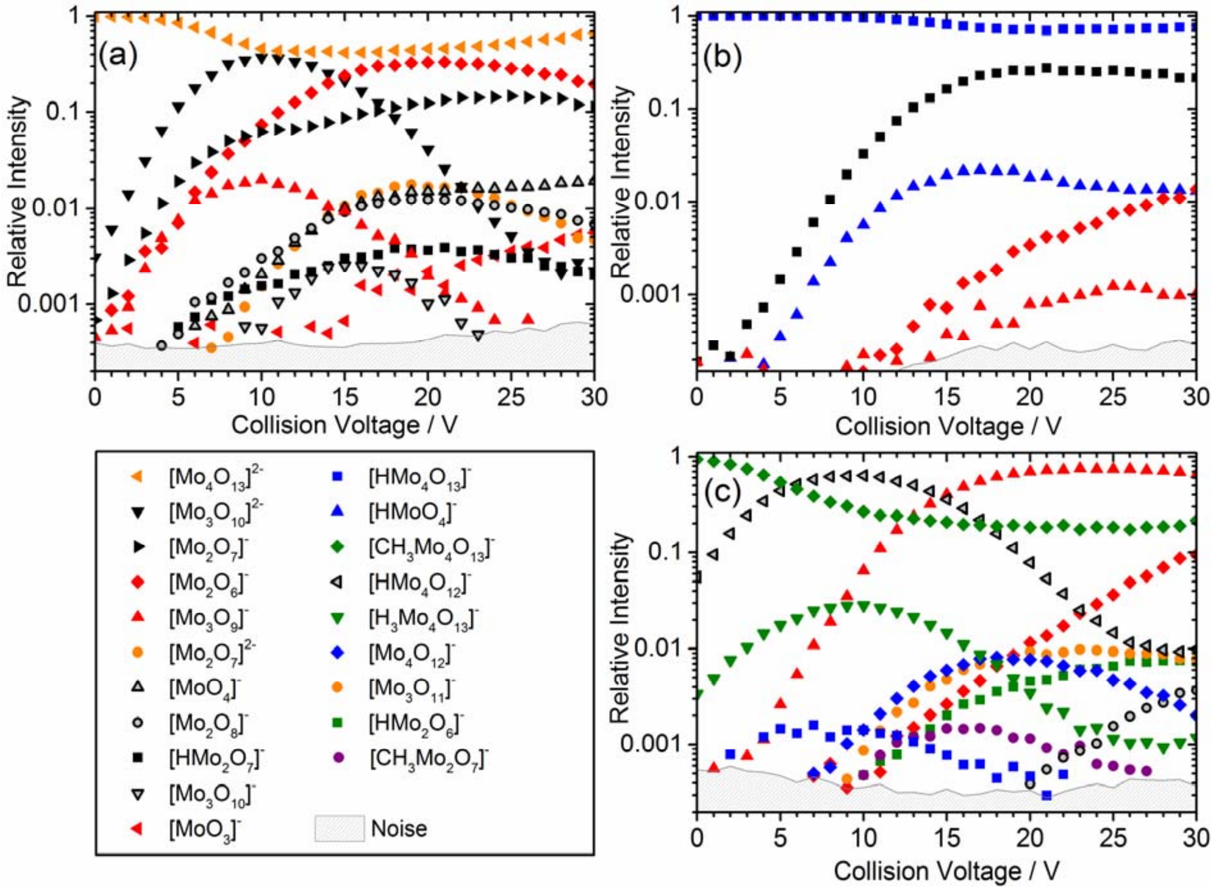

Figure S2: CID breakdown curves of the $[Mo_4O_{13}]^{2-}$ (a), $[HMo_4O_{13}]^-$ (b) and $[CH_3Mo_4O_{13}]^-$ (c) clusters.

Table S1: Overview of all measured fragment ions, sorted by parent ion.

$[Mo_4O_{13}]^{2-}$ Fragment	Measured m/z	Exact m/z
$Mo_4O_{13}^{2-}$	287.780	287.781
$Mo_3O_{10}^{2-}$	217.834	217.835
$Mo_2O_7^{-}$	295.778	295.779
$Mo_2O_6^{-}$	279.783	279.784
$Mo_3O_9^{-}$	419.675	419.675
$Mo_2O_7^{2-}$	147.887	147.890
MoO_4^{-}	155.885	155.887
$Mo_2O_8^{-}$	311.773	311.773
$HMo_2O_7^{-}$	296.786	296.786
$Mo_3O_{10}^{-}$	435.670	435.670
MoO_3^{-}	139.889	139.892

$[HMo_4O_{13}]^{-}$ Fragment	Measured m/z	Exact m/z
$HMo_4O_{13}^{-}$	576.568	576.570
$HMo_2O_7^{-}$	296.786	296.786
$HMoO_4^{-}$	156.892	156.894
$Mo_2O_6^{-}$	279.782	279.784
$Mo_3O_9^{-}$	419.675	419.675

$[CH_3Mo_4O_{13}]^{-}$ Fragment	Measured m/z	Exact m/z
$CH_3Mo_4O_{13}^{-}$	590.583	590.585
$HMo_4O_{12}^{-}$	560.574	560.575
$H_3Mo_4O_{13}^{-}$	578.584	578.585
$Mo_4O_{12}^{-}$	559.566	559.567
$Mo_3O_{11}^{-}$	451.665	451.665
$Mo_2O_6^{-}$	279.783	279.784
$HMo_2O_6^{-}$	280.791	280.791
$CH_3Mo_2O_7^{-}$	310.802	310.802
$Mo_3O_9^{-}$	419.675	419.675
$HMo_4O_{13}^{-}$	576.569	576.570
$Mo_2O_8^{-}$	311.773	311.773

Table S2: Energies of calculated structures on the wB97XD level of theory. See Figures for the respective structures.

Ion	Isomer	Singlet/Douplet	Triplet/Quartet	Pentet/Hextet
$[HMo_2O_6]^-$	I	0.00	-	-
	II	0.25	0.64	-
	III	0.67	0.71	-
	IV	2.25	0.71	-
$[CH_3OH]$	I	0.00	-	-
$[Mo_2O_6]^-$	I	0.00	3.41	-
$[Mo_2O_8]^-$	I	0.00	2.89	-
	II	2.62	2.60	-
	III	2.71	2.72	-
$[CO_2]$	I	0.00	4.49	-
$[Mo_4O_{13}]^{2-}$	I	0.00	2.47	-
	II	0.20	3.32	-
	III	0.47	3.25	-
	IV	2.75	2.75	-
	V	2.85	2.85	-
	VI	3.26	3.21	-
	VII	4.08	4.08	-
	VIII	4.21	4.31	-
$[CH_2O_2]$	I	0.00	1.51	-
$[Mo_2O_8]^{2-}$	I	0.00	2.01	-
	II	2.12	2.12	-
	III	2.53	-	-
$[Mo_2O_6]^{2-}$	I	0.00	0.25	-
$[HMo_3O_{10}]^-$	I	0.00	2.95	-
	II	0.17	3.02	-
	III	0.50	3.07	-
	IV	0.77	2.91	-
	V	0.87	-	-
	VI	1.18	3.61	-
	VII	1.20	3.92	-

$[Mo_2O_7]^{2-}$	I	0.00	3.39	-
$[CH_3O]$	I	0.00	-	-
$[MoO_4]^-$	I	0.00	3.49	-
	II	3.49	3.89	7.33
$[HMoO_4]^-$	I	0.00	3.19	-
	II	5.02	5.41	-
	III	5.39	5.43	-
	IV	6.05	-	-
$[Mo_2O_5]^-$	I	0.00	0.02	3.13
$[HMo_4O_{12}]^-$	I	0.30	0.00	-
	II	1.22	0.10	-
	III	0.23	0.33	-
	IV	0.36	0.25	-
	V	0.35	0.35	-
	VI	0.39	2.28	-
	VII	1.75	1.25	-
$[MoO_3]^-$	I	0.00	2.96	6.49
$[Mo_3O_{10}]^-$	I	0.00	2.76	-
	II	0.32	2.29	-
	III	0.45	3.05	-
$[CH_3Mo_2O_7]$	I	0.00	3.26	-
$[CH_2O]$	I	0.00	2.92	-
$[HMo_4O_{13}]^-$	I	0.00	3.14	-
	II	0.21	2.70	-
	III	0.35	3.51	-
	IV	0.42	3.73	-
	V	0.48	2.61	-
	VI	0.61	3.77	-
	VII	0.66	2.72	-
	VIII	1.17	3.46	-
	IX	1.17	3.41	-
	X	1.31	2.95	-
	XI	1.40	3.74	-
	XII	1.73	4.20	-
	XIII	1.40	3.74	-
$[HMoO_3]$	I	0.00	0.37	-
$[HMo_2O_7]$	I	0.00	5.44	-

	II	0.69	2.91	-
[$CH_3Mo_2O_5$]	I	0.00	2.23	-
	II	1.19	1.28	-
	III	1.52	1.32	-
	IV	1.63	1.87	-
	V	2.33	2.35	-
[Mo_3O_9]$^-$	I	0.00	3.30	-
	II	0.27	3.45	-
[Mo_2O_6]	I	0.00	2.71	5.57
[HMo_2O_7]$^-$	I	0.00	2.73	-
	II	0.92	-	-
[$HMoO_4$]	I	0.00	3.41	-
	II	1.49	3.12	-
	III	2.59	4.97	-
	IV	3.25	4.64	-
[Mo_2O_7]$^-$	I	0.00	3.12	-
[O_2]	I	0.50	0.00	-
[$CH_3Mo_4O_{13}$]$^-$	I	0.00	2.66	-
	II	0.18	2.55	-
	III	0.23	2.48	-
	IV	0.26	3.55	-
	V	0.37	2.66	-
	VI	0.45	2.66	-
	VII	0.52	2.68	-
	VIII	0.92	-	-
	IX	0.97	1.71	-
	X	1.10	-	-
	XI	1.10	-	-
	XII	1.21	-	-
	XIII	1.22	-	-
	XIV	1.22	-	-
	XV	1.22	-	-
	XVI	1.45	-	-
	XVII	1.52	-	-
	XVIII	1.52	-	-
	XIX	1.54	3.12	-

	XX	1.54	-	-
	XXI	1.65	-	-
	XXII	1.65	-	-
	XXIII	1.65	-	-
	XXIV	1.65	-	-
	XXV	1.66	-	-
	XXVI	1.67	-	-
	XXVII	1.72	-	-
	XXVIII	1.74	-	-
	XXIX	1.84	-	-
	XXX	1.95	-	-
	XXXI	2.05	-	-
	XXXII	2.19	-	-
	XXXIII	2.25	-	-
	XXXIV	2.31	-	-
	XXXV	2.35	-	-
	XXXVI	2.49	-	-
	XXXVII	2.57	-	-
	XXXVIII	2.66	-	-
	XXXIX	2.66	-	-
	XL	2.76	-	-
	XLI	2.93	-	-
[H_2O]	I	0.00	-	-
[CH_3MoO_2]	I	0.00	2.47	-
[CH_3MoO_4]	I	0.00	3.23	-
	II	1.49	3.17	-
	III	1.62	-	-
	IV	2.45	4.76	-
[Mo_3O_{10}]$^{2-}$	I	0.00	3.54	-
	II	1.14	3.58	-
	III	4.24	4.14	-
	IV	4.98	4.81	-
[Mo_3O_9]	I	0.00	3.08	-
	II	1.38	3.61	-
[Mo_4O_{12}]$^{-}$	I	0.00	3.33	-

	II	0.36	3.47	-
	III	0.80	3.44	-
	IV	1.11	-	-
	V	1.53	3.71	-
$[CH_2Mo_2O_7]$	I	0.00	3.23	-
$[H_3Mo_4O_{13}]^-$	I	0.01	0.00	-
	II	0.44	0.61	-
	III	0.55	0.54	-
	IV	0.59	0.59	-
	V	1.05	1.01	-
	VI	1.22	1.24	-
	VII	1.26	1.24	-
	VIII	1.58	1.61	-
	IX	2.00	2.00	-
	X	2.04	2.03	-
	XI	2.32	2.32	-
	XII	2.59	2.57	-
$[CH_3Mo_2O_7]^-$	I	0.00	3.16	-
	II	0.94	3.81	-
$[H_2]$	I	0.00	-	-
$[MoO_3]$	I	0.00	1.70	4.41
$[Mo_3O_{11}]^-$	I	0.00	-	-
	II	2.38	2.67	-
$[Mo_2O_7]$	I	0.00	0.00	-
	II	0.71	0.67	-
$[MoO_4]$	I	0.02	0.00	-
$[Mo_2O_5]$	I	0.00	0.53	3.12

Table S3: Benchmarking various properties of small molybdenum oxide and sulfide clusters using different DFT functionals along with the def2TZVP basis set. Experimental values taken from Ref. [1–3].

quantity	ωB97XD	B3LYP	M06	exp.
$D_0(Mo^+\text{-}O)$	4.76	5.07	5.10	5.06 ± 0.02
$D_0(OMo^+\text{-}O)$	5.32	5.25	5.24	5.57 ± 0.14
IE(MoO)	7.38	7.57	7.48	7.79 ± 0.22
$D_0(Mo^+–S)$	3.43	3.73	3.75	3.68 ± 0.06
$D_0(SMo^+–S)$	3.89	3.87	4.35	4.10 ± 0.10
$D_0(Mo^+–S2)$	2.81	3.17	3.53	3.42 ± 0.11
$D_0(S_2Mo^+–S)$	3.45	3.48	3.71	3.37 ± 0.09
$D_0(SMo^+–S_2)$	2.83	2.92	3.50	3.11 ± 0.13

References

1. Sievers, M.R., Chen, Y.-M., Armentrout, P.B.: Metal oxide and carbide thermochemistry of Y^+, Zr^+, Nb^+, and Mo^+. J. Chem. Phys. **105**, 6322–6333 (1996)
2. Sievers, M.R., Armentrout, P.B.: Reactions of CO and CO_2 with Gas-Phase Mo^+, MoO^+, and MoO_2^+. J. Phys. Chem. A **102**, 10754–10762 (1998)
3. Kretzschmar, I., Schröder, D., Schwarz, H., Armentrout, P.B.: Structure, Thermochemistry, and Reactivity of MS_n^+ Cations (M=V, Mo; n=1–3) in the Gas Phase. Int. J. Mass Spectrom. **228**, 439–456 (2003)
4. Zhai, H.-J., Huang, X., Waters, T., Wang, X.-B., O'Hair, R.A.J., Wedd, A.G., Wang, L.-S.: Photoelectron spectroscopy of doubly and singly charged group VIB dimetalate anions: $M_2O_7^{2-}$, $MM'O_7^{2-}$, and $M_2O_7^-$ (M, M' = Cr, Mo, W). J. Phys. Chem. A **109**, 10512–10520 (2005)
5. Zhai, H.-J., Kiran, B., Cui, L.-F., Li, X., Dixon, D.A., Wang, L.-S.: Electronic structure and chemical bonding in MO(n)- and MO(n) clusters (M = Mo, W; n = 3-5): a photoelectron spectroscopy and ab initio study. J. Am. Chem. Soc. **126**, 16134–16141 (2004)

Table S4: Benchmarking vertical detachment energies (in eV) for various molybdenum oxides using different DFT functionals along with the def2TZVP basis set. Experimental values are taken from [4, 5].

Ion	ωB97XD	B3LYP	M06	exp.
$[MoO_3]^-$	3.29	3.41	3.26	3.33(5)
$[MoO_4]^-$	5.49	5.37	5.44	5.45(5)
$[MoO_5]^-$	5.48	5.39	5.41	5.40(5)
$[Mo_2O_7]^-$	6.90	6.60	6.76	~ 6.5
$[Mo_2O_7]^{2-}$	2.30	1.80	1.98	~ 2.2

Calculated structures on the wB97XD/def2TZVP level of theory

Zero-point corrected energies are given in eV relative to the most stable structure in Figures S3.1-S3.43.

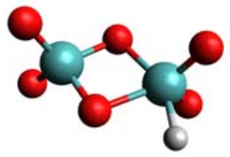

^{1}I E=0.00

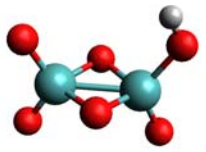

II E=0.25

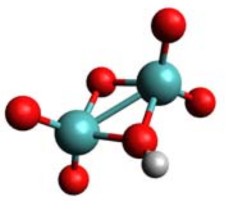

III E=0.67

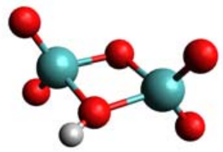

IV* E=0.71

Figure S3.1: Calculated Molecules of $[HMo_2O_6]^-$ in lowest multiplicity unless marked with *.

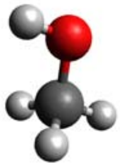

I E=0.00

Figure S3.2: Calculated Molecules of $[CH_3OH]$ in lowest multiplicity unless marked with *.

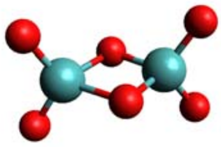

I E=0.00

Figure S3.S3.3: Calculated Molecules of $[Mo_2O_6]^-$ in lowest multiplicity unless marked with *.

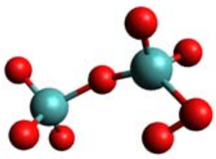

I E=0.00

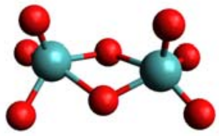

II* E=2.60

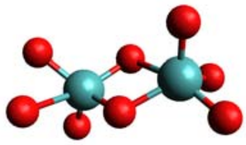

III E=2.71

Figure S3.4: Calculated Molecules of $[Mo_2O_8]^-$ in lowest multiplicity unless marked with *.

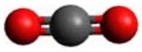

I E=0.00

Figure S3.5: Calculated Molecules of [CO_2] in lowest multiplicity unless marked with *.

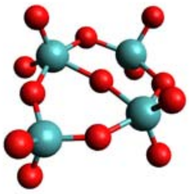

I E=0.00

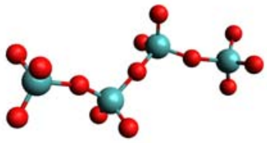

II E=0.20

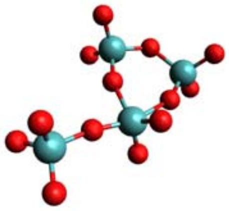

III E=0.47

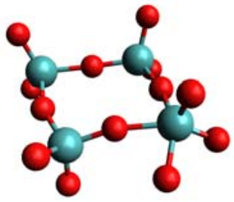

IV E=2.75

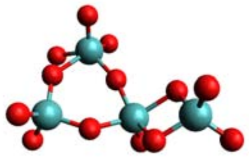

V E=2.85

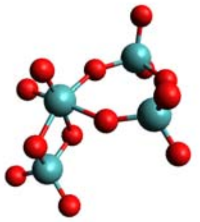

VI* E=3.21

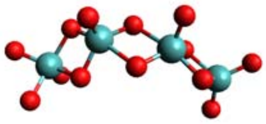

VII* E=4.08

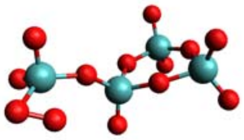

VIII E=4.21

Figure S3.6: Calculated Molecules of $[Mo_4O_{13}]^{2-}$ in lowest multiplicity unless marked with *.

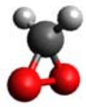

I E=0.00

Figure S3.7: Calculated Molecules of [CH_2O_2] in lowest multiplicity unless marked with *.

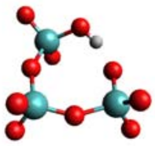

I E=0.00

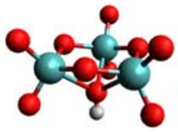

II E=0.17

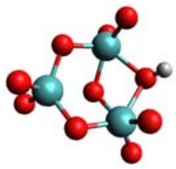

III E=0.50

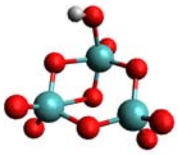

IV E=0.77

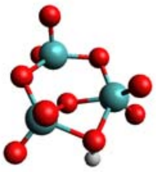

V E=0.87

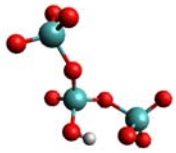

VI E=1.18

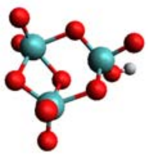

VII E=1.20

Figure S3.10: Calculated Molecules of $[HMo_3O_{10}]^-$ in lowest multiplicity unless marked with *.

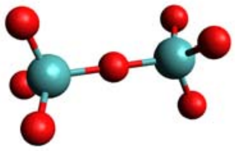

I E=0.00

Figure S3.11: Calculated Molecules of $[Mo_2O_7]^{2-}$ in lowest multiplicity unless marked with *.

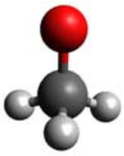

I E=0.00

Figure S3.12: Calculated Molecules of $[CH_3O]$ in lowest multiplicity unless marked with *.

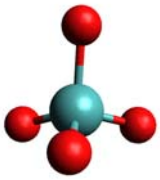

I E=0.00

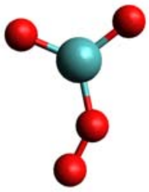

II E=3.49

Figure S3.13: Calculated Molecules of $[MoO_4]^-$ in lowest multiplicity unless marked with *.

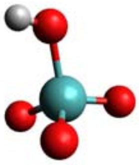

I E=0.00

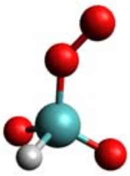

II E=5.02

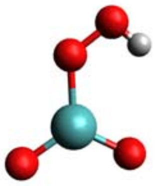

III E=5.39

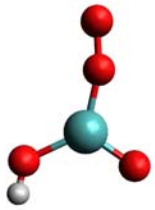

IV E=6.05

Figure S3.14: Calculated Molecules of $[HMoO_4]^-$ in lowest multiplicity unless marked with *.

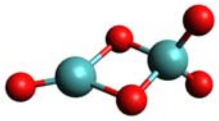

I E=0.00

Figure S3.16: Calculated Molecules of $[Mo_2O_5]^-$ in lowest multiplicity unless marked with *.

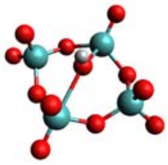

I* E=0.00

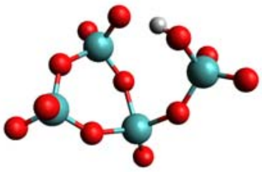

II* E=0.10

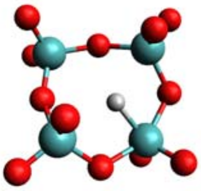

III E=0.23

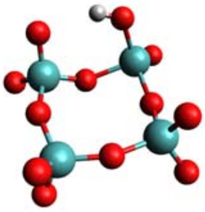

IV* E=0.25

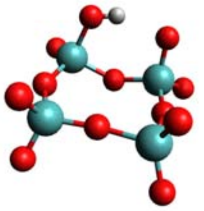

V E=0.35

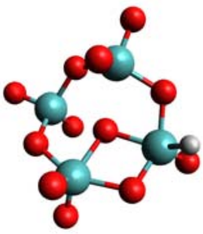

VI E=0.39

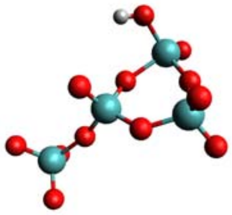

VII* E=1.25

Figure S3.17: Calculated Molecules of $[HMo_4O_{12}]^-$ in lowest multiplicity unless marked with *.

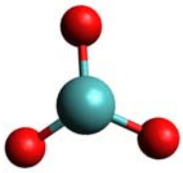

I E=0.00

Figure S3.18: Calculated Molecules of $[MoO_3]^-$ in lowest multiplicity unless marked with *.

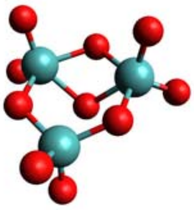

I E=0.00

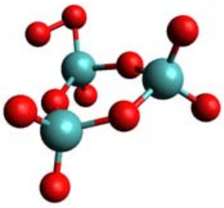

II E=0.32

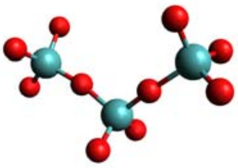

III E=0.45

Figure S3.19: Calculated Molecules of $[Mo_3O_{10}]^-$ in lowest multiplicity unless marked with *.

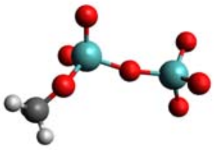

I E=0.00

Figure S3.20: Calculated Molecules of [$CH_3Mo_2O_7$] in lowest multiplicity unless marked with *.

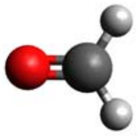

I E=0.00

Figure S3.21: Calculated Molecules of [CH_2O] in lowest multiplicity unless marked with *.

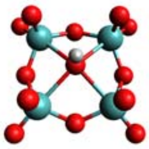

I E=0.00

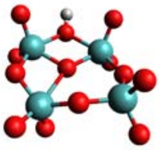

II E=0.21

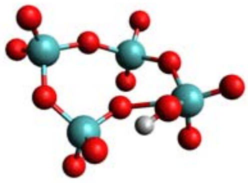

III E=0.35

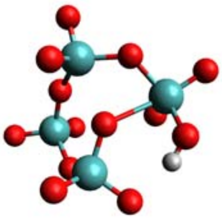

IV E=0.42

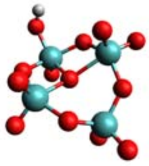

V E=0.48

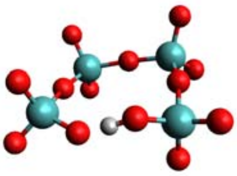

VI E=0.61

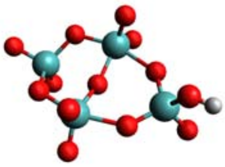

VII E=0.66

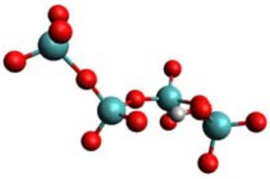

VIII E=1.17

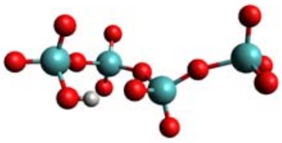

IX E=1.17

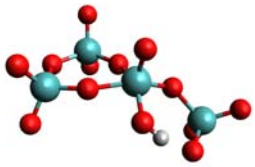

X E=1.31

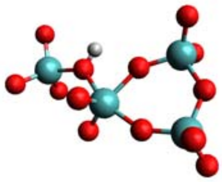

XI E=1.40

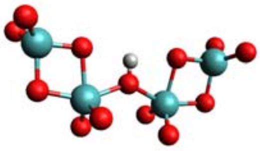

XII E=1.73

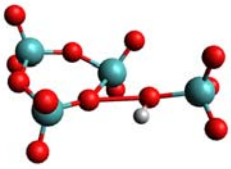

XIII E=1.40

Figure S3.21: Calculated Molecules of $[HMo_4O_{13}]^-$ in lowest multiplicity unless marked with *.

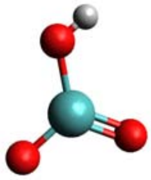

I E=0.00

Figure S3.22: Calculated Molecules of $[HMoO_3]$ in lowest multiplicity unless marked with *.

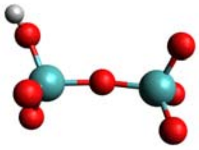

I E=0.00

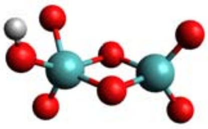

II E=0.69

Figure S3.23: Calculated Molecules of $[HMo_2O_7]$ in lowest multiplicity unless marked with *.

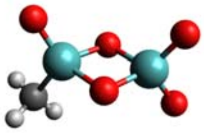

I E=0.00

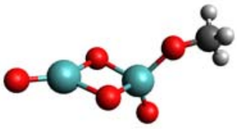

II E=1.19

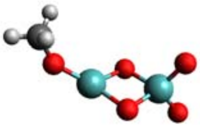

III* E=1.32

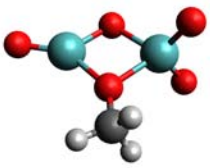

IV E=1.63

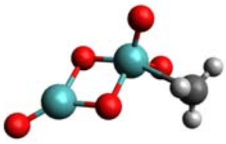

V E=2.33

Figure S3.24: Calculated Molecules of $[CH_3Mo_2O_5]$ in lowest multiplicity unless marked with *.

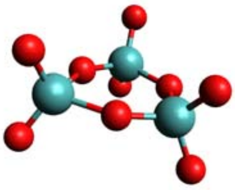

I E=0.00

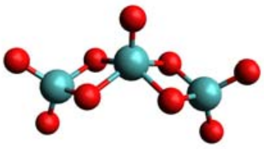

II E=0.27

Figure S3.25: Calculated Molecules of $[Mo_3O_9]^-$ in lowest multiplicity unless marked with *.

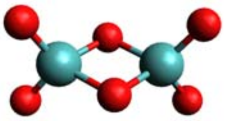

I E=0.00

Figure S3.26: Calculated Molecules of $[Mo_2O_6]$ in lowest multiplicity unless marked with *.

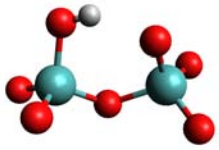

I E=0.00

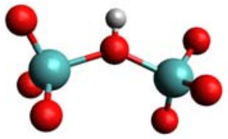

II E=0.92

Figure S3.27: Calculated Molecules of $[HMo_2O_7]^-$ in lowest multiplicity unless marked with *.

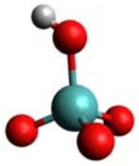

I E=0.00

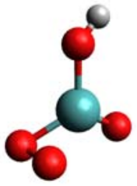

II E=1.49

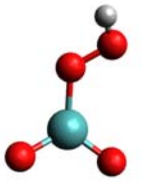

III E=2.59

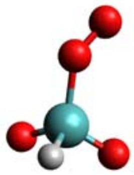

IV E=3.25

Figure S3.28: Calculated Molecules of $[HMoO_4]$ in lowest multiplicity unless marked with *.

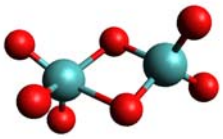

I E=0.00

Figure S3.29: Calculated Molecules of $[Mo_2O_7]^-$ in lowest multiplicity unless marked with *.

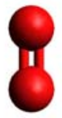

I* E=0.00

Figure S3.30: Calculated Molecules of [O_2] in lowest multiplicity unless marked with *.

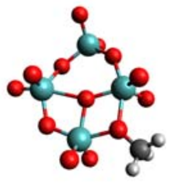

I E=0.00

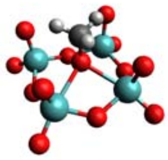

II E=0.18

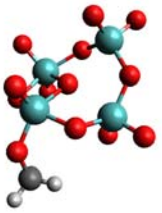

III E=0.23

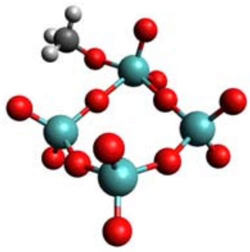

IV E=0.26

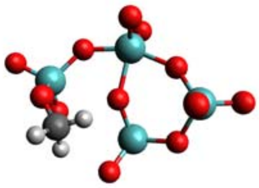

V E=0.37

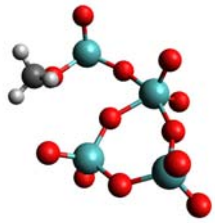

VI E=0.45

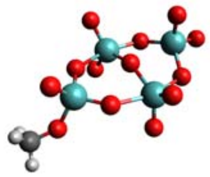

VII E=0.52

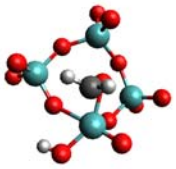

VIII E=0.92

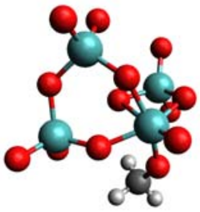

IX E=0.97

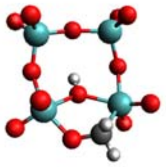

X E=1.10

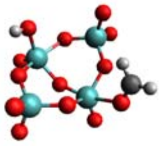

XI E=1.10

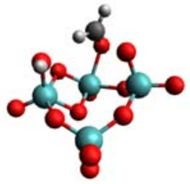

XII E=1.21

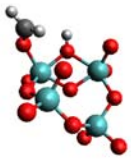

XIII E=1.22

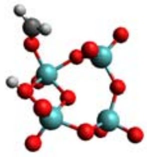

XIV E=1.22

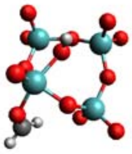

XV E=1.22

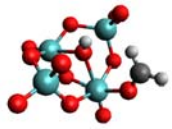

XVI E=1.45

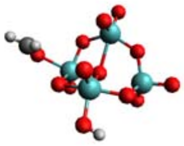

XVII E=1.52

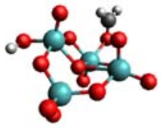

XVIII E=1.52

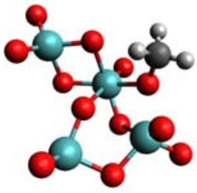

XIX E=1.54

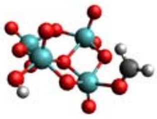

XX E=1.54

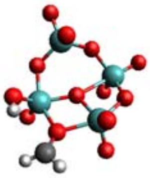

XXI E=1.65

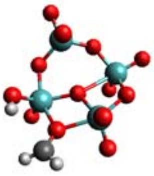

XXII E=1.65

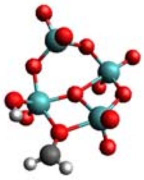

XXIII E=1.65

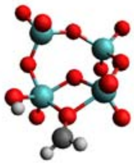

XXIV E=1.65

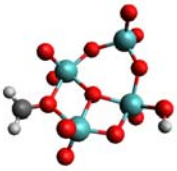

XXV E=1.66

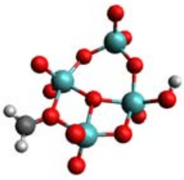

XXVI E=1.67

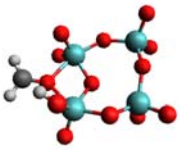

XXVII E=1.72

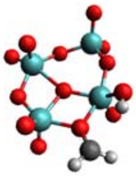

XXVIII E=1.74

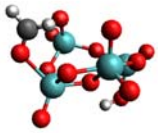

XXIX E=1.84

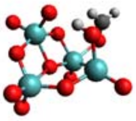

XXX E=1.95

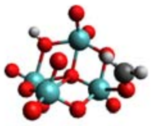

XXXI E=2.05

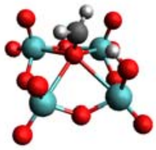

XXXII E=2.19

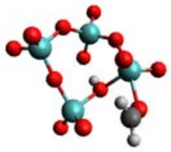

XXXIII E=2.25

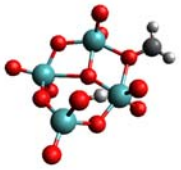

XXXIV E=2.31

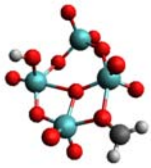

XXXV E=2.35

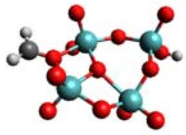

XXXVI E=2.49

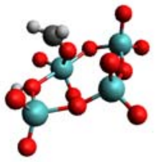

XXXVII E=2.57

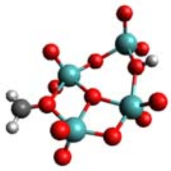

XXXVIII E=2.66

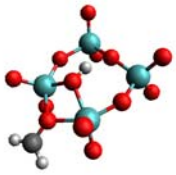

XXXIX E=2.66

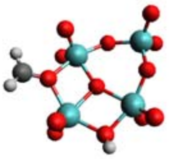

XL E=2.76

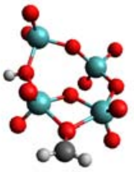

XLI E=2.93

Figure S3.28: Calculated Molecules of $[CH_3Mo_4O_{13}]^-$ in lowest multiplicity unless marked with *.

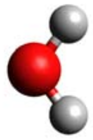

I E=0.00

Figure S3.29: Calculated Molecules of [H_2O] in lowest multiplicity unless marked with *.

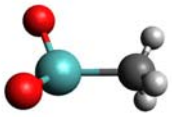

I E=0.00

Figure S3.30: Calculated Molecules of [CH_3MoO_2] in lowest multiplicity unless marked with *.

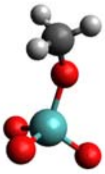

I E=0.00

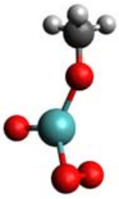

II E=1.49

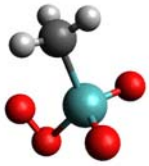

III E=1.62

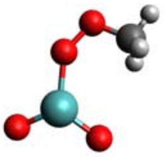

IV E=2.45

Figure S3.31: Calculated Molecules of [CH_3MoO_4] in lowest multiplicity unless marked with *.

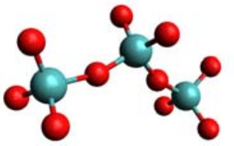

I E=0.00

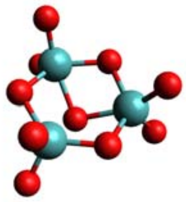

II E=1.14

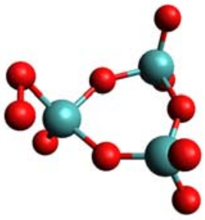

III* E=4.14

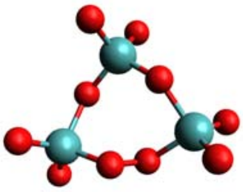

IV* E=4.81

Figure S3.32: Calculated Molecules of $[Mo_3O_{10}]^{2-}$ in lowest multiplicity unless marked with *.

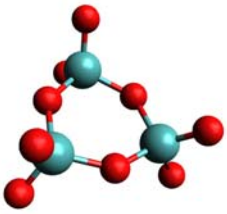

I E=0.00

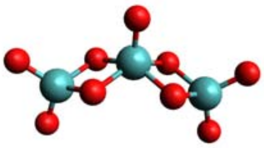

II E=1.38

Figure S3.33: Calculated Molecules of $[Mo_3O_9]$ in lowest multiplicity unless marked with *.

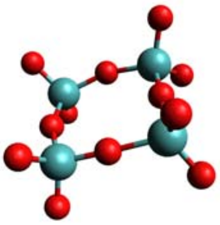

I E=0.00

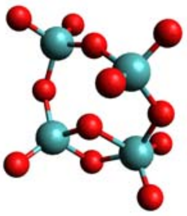

II E=0.36

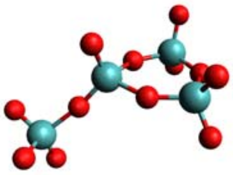

III E=0.80

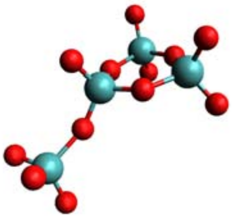

IV E=1.11

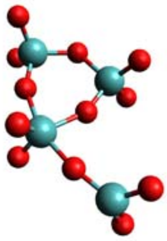

V E=1.53

Figure S3.34: Calculated Molecules of $[Mo_4O_{12}]^-$ in lowest multiplicity unless marked with *.

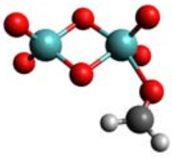

I E=0.00

Figure S3.35: Calculated Molecules of $[CH_2Mo_2O_7]$ in lowest multiplicity unless marked with *.

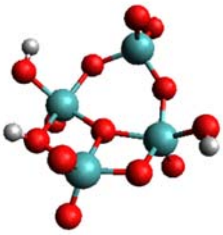

I* E=0.00

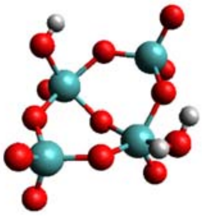

II E=0.44

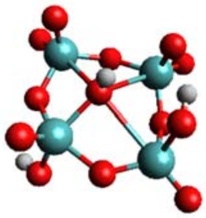

III* E=0.54

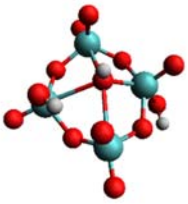

IV* E=0.59

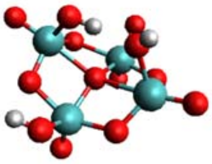

V* E=1.01

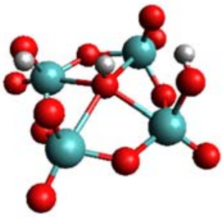

VI E=1.22

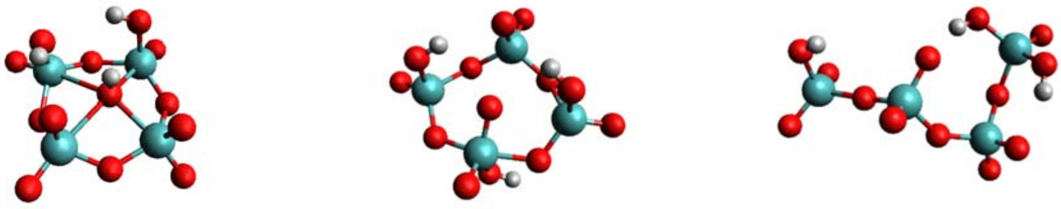

VII* E=1.24 VIII E=1.58 IX* E=2.00

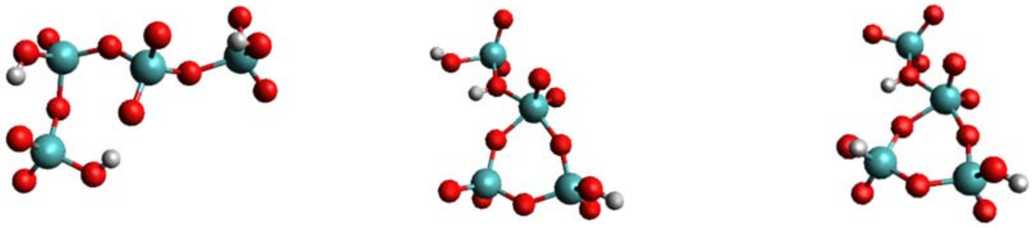

X* E=2.03 XI E=2.32 XII* E=2.57

Figure S3.36: Calculated Molecules of $[H_3Mo_4O_{13}]^-$ in lowest multiplicity unless marked with *.

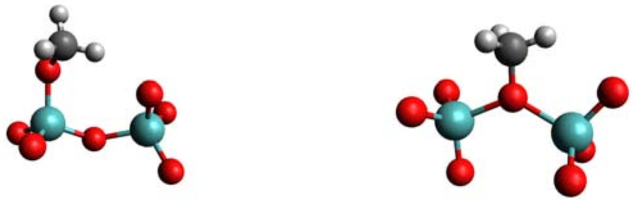

I E=0.00 II E=0.94

Figure S3.37: Calculated Molecules of $[CH_3Mo_2O_7]^-$ in lowest multiplicity unless marked with *.

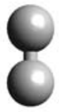

I E=0.00

Figure S3.38: Calculated Molecules of [H_2] in lowest multiplicity unless marked with *.

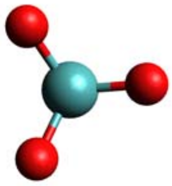

I E=0.00

Figure S3.39: Calculated Molecules of [MoO_3] in lowest multiplicity unless marked with *.

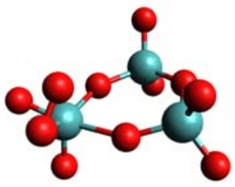

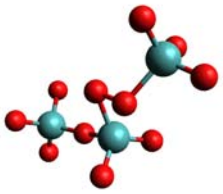

I E=0.00 II E=2.38

Figure S3.40: Calculated Molecules of [Mo_3O_{11}]$^-$ in lowest multiplicity unless marked with *.

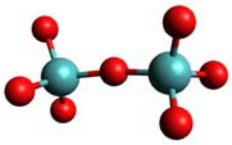

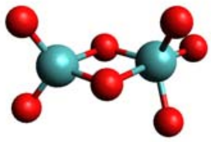

I E=0.00 II* E=0.67

Figure S3.41: Calculated Molecules of [Mo_2O_7] in lowest multiplicity unless marked with *.

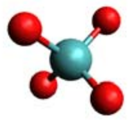

I* E=0.00

Figure S3.42: Calculated Molecules of [MoO_4] in lowest multiplicity unless marked with *.

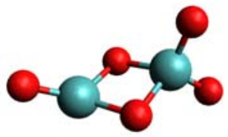

I E=0.00

Figure S3.43: Calculated Molecules of [Mo_2O_5] in lowest multiplicity unless marked with *.

Calculated transition states at the wB97XD/def2TZVP level of theory

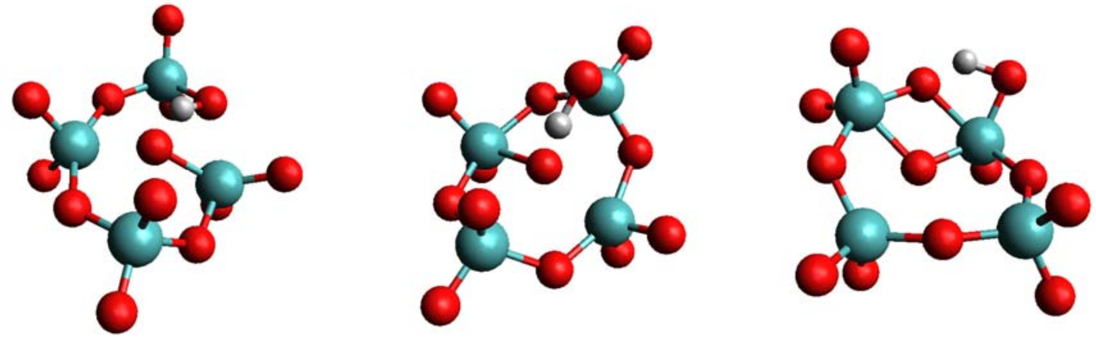

I → II E=1.44 | I→V E=0.94 | II→V E=1.30

Figure S3.44: Calculated transition states of $[HMo_4O_{13}]^-$ in lowest multiplicity. The given energies are the barriers in eV.

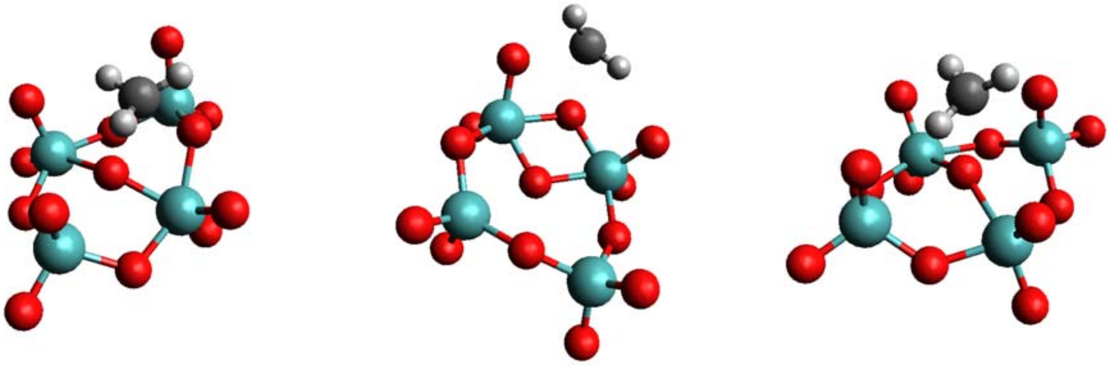

I → II E=3.22 | I→III E=3.17 | II→III E=2.94

Figure S3.45: Calculated transition states of $[CH_3Mo_4O_{13}]^-$ in lowest multiplicity. The given energies are the barriers in eV.

Cartesian coordinates (in Ångstrom) of transition states optimized at the wB97XD/def2TZVP level of theory along the respective ZPE-corrected energy (in eV)

$[HMo_4O_{13}]^-$ I → II

E = -34075.44

Mo	0.05649	1.91937	0.16136
O	3.08540	-0.12063	-1.80126
O	0.04093	3.16713	-0.95099
O	-0.09795	2.57053	1.69645
O	-1.89258	1.38214	0.07042
Mo	2.85571	0.00605	-0.14047
Mo	-3.01481	-0.02602	-0.15868
O	-3.74932	-0.53663	1.26517
O	-4.20224	0.28928	-1.31135
O	1.98349	1.56754	0.32256
O	0.22923	-0.02030	-0.60424
O	4.38060	0.05915	0.55800
Mo	0.17281	-1.90016	0.17668
O	1.99015	-1.53733	0.55696
O	-0.53363	-2.19807	1.66559
O	-1.69751	-1.24718	-0.81416
O	0.20581	-3.31151	-0.72524
H	-0.88717	-0.48095	-1.05622

$[HMo_4O_{13}]^-$ I → V

E = -34075.94

Mo	2.53305	-0.00005	-0.48350
O	0.12887	-3.39184	-0.30941
O	1.70077	-0.00003	-2.05801
O	4.19800	-0.00002	-0.66424
O	1.98669	1.47142	0.40667
Mo	-0.00054	-1.95088	0.53090
Mo	-0.00061	1.95099	0.53082
O	0.03360	2.27275	2.17218
O	0.12915	3.39180	-0.30970
O	1.98669	-1.47166	0.40652
O	-0.00240	-0.00008	-0.38908
O	0.03361	-2.27228	2.17233
Mo	-2.53126	-0.00006	-0.48627
O	-1.87108	-1.56810	0.30651
O	-4.18129	0.00011	-0.18013
O	-1.87091	1.56814	0.30630
O	-2.35553	-0.00023	-2.16001
H	0.64385	-0.00008	-1.46121

$[HMo_4O_{13}]^-$ II → V

E = -34075.59

Mo	2.71602	-0.22221	-0.40588
O	0.05383	2.22969	2.02049
O	3.97030	-0.13217	0.70728
O	3.38741	-0.37535	-1.93795
O	1.61895	-1.69162	-0.02832
Mo	0.09812	2.22516	0.34434
Mo	-0.18921	-2.00227	0.47769
O	-0.65576	-3.36977	-0.58542
O	-0.18231	-2.53784	2.05505
O	1.64467	1.27440	-0.32431
O	-0.57868	-0.18544	0.46808
O	0.44012	3.80311	-0.10523
Mo	-2.44651	0.13281	-0.42625
O	-1.56319	1.89710	-0.39577
O	-2.85948	0.10413	-2.05469
O	-2.17612	-1.79386	-0.16101
O	-3.82445	0.42541	0.48397
H	-1.69579	-2.78857	-0.71331

$[CH_3Mo_4O_{13}]^-$ I → II

E = -35142.15

O	0.00933	-3.24832	-1.34011
O	-0.06774	-2.70725	1.30551
Mo	0.10367	-2.00949	-0.21280
O	1.97051	-1.62734	-0.46587
O	-1.66642	-1.26018	-0.64446
O	4.25350	-0.02644	-0.58102
O	2.89085	0.16430	1.74076
Mo	2.70393	0.03169	0.06740
O	0.37054	0.06772	0.41798
Mo	-2.82640	0.01281	0.00435
O	-3.88431	0.57554	-1.17935
O	-3.70391	-0.58746	1.31873
O	1.98270	1.61055	-0.68773
O	-1.67843	1.32692	0.65220
Mo	0.13270	1.91350	-0.26995
O	-0.56442	2.25702	-1.76184
O	0.21922	3.28457	0.70492
C	-0.65284	0.40768	2.32081
H	0.39157	0.61302	2.50668
H	-1.37882	1.10249	2.70928
H	-0.93096	-0.63600	2.28322

$[CH_3Mo_4O_{13}]^-$ I → III

E = -35142.20

O	3.89823	-0.39649	1.07355
O	3.57749	-1.20229	-1.51849
Mo	2.77380	-0.60157	-0.16397
O	1.45062	-1.73936	0.36042
O	1.94098	1.01429	-0.60184
O	-0.71071	-2.97013	1.65887
O	-0.56817	-2.94832	-1.09623
Mo	-0.50765	-1.99730	0.30631
O	-0.44999	0.04482	0.43255
Mo	0.56985	2.20939	-0.03593
O	0.70693	2.52917	1.60017
O	1.06540	3.61081	-0.81005
O	-2.32589	-1.32122	0.06263
O	-1.26750	2.17579	-0.55022
Mo	-2.25907	0.68655	0.08320
O	-3.02340	1.16106	1.49276
O	-3.45011	0.37870	-1.16053
C	-3.41622	-1.63514	-1.68305
H	-2.55324	-2.27771	-1.80841
H	-4.20756	-1.99516	-1.04664
H	-3.72399	-1.08780	-2.55935

$[CH_3Mo_4O_{13}]^-$ II → III

E = -35142.43

O	-4.31806	0.03598	-0.33167
O	-2.69118	-0.09687	1.86591
Mo	-2.71256	0.01694	0.16945
O	-1.86209	-1.44320	-0.57559
O	-1.89067	1.56560	-0.38273
O	-0.02625	-3.01584	-1.71687
O	0.01103	-2.93927	1.00258
Mo	0.00551	-1.99652	-0.38419
O	0.13562	0.03590	0.30010
Mo	0.03501	1.98076	-0.39600
O	-0.01290	2.76428	-1.88009
O	0.13952	3.10782	0.84708
O	1.90969	-1.53572	-0.64025
O	1.97868	1.52888	-0.60887
Mo	2.60633	-0.01610	0.13209
O	4.25890	-0.05319	-0.16045
O	2.38871	-0.02137	1.85213
C	0.31107	0.12007	2.57104
H	0.88278	-0.56034	3.17808
H	0.52597	1.17500	2.65481
H	-0.68325	-0.17247	2.27405

Cartesian coordinates (in Ångstrom) of species optimized at the wB97XD/def2TZVP level of theory along the respective ZPE-corrected energy (in eV)

$[HMo_2O_6]^-$ I 1/2

E = -16022.04

Mo	1.495589	0.000346	-0.095315
Mo	-1.460429	0.000224	0.018274
O	-0.054987	0.006121	1.217293
O	-0.253002	-0.004390	-1.338410
O	2.366369	-1.388003	0.294968
O	2.373747	1.383161	0.299087
O	-2.433005	1.396627	0.068489
O	-2.430946	-1.397108	0.080205
H	1.977878	0.004796	-1.737337

$[HMo_2O_6]^-$ II 1/2

E = -16021.78

Mo	1.257240	-0.097016	0.000235
Mo	-1.289490	0.020129	-0.000024
O	0.007134	0.092026	1.444152
O	0.007495	0.092778	-1.443787
O	1.938898	-1.634238	-0.000627
O	2.349119	1.480105	-0.000325
O	-2.167427	1.496727	0.000077
O	-2.210493	-1.418180	-0.000511
H	1.956694	2.355479	-0.000709

$[HMo_2O_6]^-$ II 3/4

E = -16021.40

Mo	1.467921	-0.083624	-0.000125
Mo	-1.526250	0.012285	-0.000005
O	0.055343	-0.099425	1.230436
O	0.055299	-0.099058	-1.230653
O	2.360379	-1.516965	0.000102
O	2.412831	1.582952	0.000432
O	-2.263823	1.558752	-0.000038
O	-2.555825	-1.353552	0.000283
H	1.936188	2.414593	0.000952

$[HMo_2O_6]^-$ III 1/2

E = -16021.37

Mo	1.315171	0.000050	-0.023936
Mo	-1.315124	0.000078	-0.024098
O	0.000005	0.000216	-1.433584
O	-0.000194	0.000100	1.530038
O	2.183434	1.475835	-0.038560
O	2.182072	-1.476479	-0.038962
O	-2.182533	-1.476202	-0.038666
O	-2.182968	1.476097	-0.038561
H	-0.000528	-0.001946	2.483829

$[HMo_2O_6]^-$ III 3/4

E = -16021.33

Mo	1.601707	-0.000004	-0.009434
Mo	-1.601702	-0.000006	-0.009430
O	-0.000006	-0.000067	-1.091775
O	0.000008	-0.000461	1.339080
O	2.502784	1.445914	-0.108557
O	2.503357	-1.445558	-0.108809
O	-2.503312	-1.445575	-0.108807
O	-2.502877	1.445857	-0.108544
H	0.000159	-0.000456	2.291588

$[HMo_2O_6]^-$ IV 1/2

E = -16019.79

Mo	-1.349844	-0.047641	-0.000010
Mo	1.349748	-0.047598	0.000025
O	0.000008	0.297387	1.346471
O	0.000228	0.299022	-1.345884
O	-2.270897	1.388689	-0.000051
O	-2.440215	-1.342460	-0.000256
O	2.439637	-1.342873	-0.000713
O	2.271808	1.388017	0.000460
H	-0.000489	-1.502217	-0.000847

$[HMo_2O_6]^-$ IV 3/4

E = -16021.33

Mo	-1.601689	0.000187	0.009501
Mo	1.601700	0.000094	0.009396
O	0.000111	0.003639	1.091808
O	-0.000176	0.006676	-1.339090
O	-2.508607	1.442256	0.111017
O	-2.497615	-1.449087	0.106157
O	2.499229	-1.448006	0.109178
O	2.507051	1.443320	0.108173
H	-0.000384	-0.002199	-2.291610

$[CH_3OH]$ I 1/2

E = -3147.90

C	0.659632	-0.020077	0.000019
H	1.024029	-0.539354	0.893766
H	1.023104	-0.549797	-0.887931
H	1.082029	0.983939	-0.006169
O	-0.743325	0.121832	0.000016
H	-1.140359	-0.748982	0.000092

$[Mo_2O_6]^-$ I 1/2

E = -16006.13

Mo	1.403437	0.000044	0.000054
Mo	-1.446626	0.000056	-0.000169
O	0.094879	0.000873	-1.314181
O	0.094674	-0.000593	1.314302
O	2.373825	1.396229	0.001130
O	2.373153	-1.396494	-0.001190
O	-2.354370	-1.438436	0.000728
O	-2.355414	1.437896	-0.000184

$[Mo_2O_6]^-$ I 3/4

E = -16002.72

Mo	1.484339	-0.044199	0.000165
Mo	-1.518875	0.013273	-0.000210
O	0.076084	-0.033485	-1.227412
O	-2.346481	1.509351	-0.000129
O	2.495595	1.618392	-0.000184
O	2.352218	-1.494367	-0.000053
O	0.075406	-0.032261	1.227275
O	-2.471509	-1.405271	0.000741

$[Mo_2O_8]^-$ I 1/2

E = -20097.82

Mo	1.995961	0.011890	-0.005209
Mo	-1.707964	-0.256045	-0.015308
O	-1.622347	1.835191	-0.054689
O	0.075407	-0.469774	-0.219335
O	-2.467261	-1.018309	-1.311297
O	-2.165620	-0.989854	1.428957
O	2.568889	0.636216	-1.491856
O	2.873163	-1.388239	0.437358
O	-2.872569	1.473771	0.097896
O	2.098357	1.202812	1.220681

$[Mo_2O_8]^-$ I 3/4

E = -20094.93

Mo	1.626513	0.036895	-0.024119
Mo	-1.312880	-0.191671	-0.114347
O	-1.642221	1.929641	-0.158447
O	0.254872	-0.222288	-1.292729
O	-2.630507	-0.906287	-0.864071
O	-1.074098	-1.785012	0.976397
O	2.578754	1.412450	-0.342652
O	2.624579	-1.317418	0.234108
O	-2.158579	1.398184	0.913969
O	0.400625	0.303306	1.260375

$[Mo_2O_8]^-$ II 1/2

E = -20095.20

Mo	1.682747	0.061810	-0.017638
Mo	-1.682723	-0.062230	-0.017382
O	-0.042654	-1.036184	0.413789
O	-2.497989	1.553699	0.584515
O	0.042323	1.036893	0.410790
O	1.915047	0.173717	-1.679398
O	2.500669	-1.549717	0.591546
O	-2.833044	-1.037234	0.773470
O	2.832037	1.042745	0.767624
O	-1.916519	-0.181713	-1.678481

$[Mo_2O_8]^-$ II 3/4

E = -20095.22

Mo	1.700893	-0.053153	0.022893
Mo	-1.700870	0.053308	0.022644
O	-0.106217	1.046504	-0.372338
O	-2.390622	-1.514504	-0.809642
O	0.106116	-1.046699	-0.370927
O	1.927898	0.041753	1.686592
O	2.392493	1.510937	-0.814361
O	-2.905244	1.067973	-0.624760
O	2.905588	-1.070498	-0.619976
O	-1.930133	-0.036279	1.686342

$[Mo_2O_8]^-$ III 1/2

E = -20095.11

Mo	1.573720	-0.129478	-0.010758
Mo	-1.441329	0.193983	0.013583
O	-0.153335	1.024499	0.900307
O	-2.588482	-1.553854	-0.579878
O	-2.216702	1.286524	-1.016576
O	1.987165	-1.024694	1.352999
O	2.141038	1.682473	0.119750
O	-2.575252	-0.468941	1.209101
O	2.740911	-0.464856	-1.196843
O	-0.030394	-0.819802	-0.803690

$[Mo_2O_8]^-$ III 3/4

E = -20095.11

Mo	1.572846	-0.129474	-0.010311
Mo	-1.442220	0.194960	0.014133
O	-0.151757	1.025334	0.897615
O	-2.586394	-1.554423	-0.581395
O	-2.219472	1.287747	-1.014301
O	1.985675	-1.027736	1.351595
O	2.154452	1.678109	0.120825
O	-2.575121	-0.470150	1.209125
O	2.738517	-0.464706	-1.198662
O	-0.031687	-0.817980	-0.804873

$[CO_2]$ I 1/2

E = -5131.94

C	-0.000000	0.000356	0.000000
O	1.155926	-0.000294	0.000000
O	-1.155926	0.000027	0.000000

$[CO_2]$ I 3/4

E = -5127.45

C	-0.000000	0.460418	0.000000
O	1.059676	-0.172654	0.000000
O	-1.059676	-0.172659	0.000000

$[Mo_4O_{13}]^{2-}$ I 1/2

E = -34060.70

Mo	0.120352	2.061413	-0.145228
O	-3.490710	0.001552	1.708918
O	-0.116543	3.180903	1.098048
O	0.048301	2.871607	-1.624947
O	1.997105	1.828387	0.035802
Mo	-2.862425	-0.000004	0.125238
Mo	2.580880	0.000036	0.157782
O	3.754513	0.001054	-1.066054
O	3.352821	-0.001365	1.666091
O	-1.780299	1.429650	-0.144483
O	0.460807	0.000081	-0.018858
O	-4.159798	-0.000873	-0.980908
Mo	0.120305	-2.061280	-0.145286
O	-1.780782	-1.430766	-0.140885
O	0.046312	-2.872108	-1.624569
O	1.997283	-1.828303	0.032763
O	-0.114354	-3.180687	1.098426

$[Mo_4O_{13}]^{2-}$ I 3/4
E = -34058.23

Mo	-0.138892	-2.060152	-0.249408
O	3.376377	-0.219651	1.830828
O	0.194693	-3.217133	0.936670
O	-0.153638	-2.830050	-1.751722
O	-1.979105	-1.793031	0.115489
Mo	2.820180	0.027007	0.241666
Mo	-2.550040	0.062303	0.291041
O	-3.828338	0.083323	-0.821174
O	-3.180829	0.027671	1.862989
O	1.729810	-1.334759	-0.285882
O	-0.486857	0.001913	-0.188693
O	4.168068	0.137575	-0.794081
Mo	-0.087858	1.941805	-0.353207
O	1.820574	1.550410	0.158216
O	-0.023110	2.536777	-1.915928
O	-1.941001	1.885385	0.179008
O	0.075562	3.324008	1.041298

$[Mo_4O_{13}]^{2-}$ II 1/2
E = -34060.51

Mo	4.937626	-0.328274	0.358191
O	5.137318	-0.838112	1.980385
O	5.910439	1.047503	0.051492
O	5.362890	-1.596223	-0.710732
Mo	1.531446	1.075551	-0.354368
Mo	-1.529628	-1.067495	-0.358781
O	-1.392852	-2.439083	0.608376
O	-1.600834	-1.579827	-1.961378
O	-3.032182	-0.148866	0.067872
O	0.000700	0.003863	-0.114121
O	1.601400	1.598061	-1.953850
O	1.396743	2.440884	0.621808
O	3.033779	0.153188	0.064386
Mo	-4.939281	0.320929	0.359597
O	-5.188568	0.631420	2.024866
O	-5.314422	1.716319	-0.558613
O	-5.915270	-0.992859	-0.144847

$[Mo_4O_{13}]^{2-}$ II 3/4
E = -34057.39

Mo	4.952040	-0.260041	0.339038
O	5.169474	-0.735631	1.970104
O	5.874581	1.146068	0.011967
O	5.429798	-1.528785	-0.707824
Mo	1.495044	1.010357	-0.374163
Mo	-1.560729	-1.156999	-0.289182
O	-1.474930	-2.437806	0.807311
O	-1.653837	-1.789491	-1.851833
O	-2.997911	-0.127486	0.055004
O	0.016873	-0.112406	-0.130487
O	1.538420	1.540548	-1.973679
O	1.319661	2.368819	0.608196
O	3.036966	0.141080	0.034394
Mo	-4.880815	0.490912	0.345474
O	-5.235675	0.854075	1.978220
O	-5.445360	1.553255	-0.869463
O	-5.607149	-1.314443	-0.043037

$[Mo_4O_{13}]^{2-}$ III 1/2
E = -34060.23

Mo	0.557904	-1.118736	0.040469
Mo	4.119201	0.105042	-0.020887
Mo	-1.543666	1.997985	0.009299
Mo	-3.337439	-0.906776	-0.028312
O	-3.135690	1.001430	-0.042073
O	-1.507885	2.955564	1.403812
O	2.220291	-0.361301	0.044158
O	5.046733	-1.255907	0.462110
O	-4.214690	-1.410630	1.340407
O	-1.669596	-1.447699	0.027211
O	-0.233494	0.758940	0.012495
O	4.543615	0.551831	-1.622340
O	4.420136	1.415913	1.044282
O	-1.450596	3.009113	-1.344268
O	-4.134531	-1.437823	-1.435419
O	0.583222	-2.075655	1.423257
O	0.603484	-2.110733	-1.316614

$[Mo_4O_{13}]^{2-}$ III 3/4
E = -34057.45

Mo	-0.427421	-1.170923	-0.025775
Mo	-4.086446	0.043677	0.031601
Mo	1.498567	2.019405	0.002846
Mo	3.321614	-0.896743	0.009138
O	3.094780	1.001412	0.017810
O	1.452864	2.998007	-1.385559
O	-2.390818	-0.411099	-0.023577
O	-5.057551	-0.921274	-0.994156
O	4.195448	-1.374393	-1.372461
O	1.675255	-1.528699	-0.012373
O	0.181646	0.816296	-0.002991
O	-4.690658	0.025138	1.624221
O	-4.354243	1.810295	-0.659134
O	1.430672	3.007745	1.383654
O	4.166838	-1.390115	1.403091
O	-0.644852	-1.988196	-1.507212
O	-0.667525	-2.021049	1.435185

$[Mo_4O_{13}]^{2-}$ IV 1/2
E = -34057.95

Mo	3.030845	0.019416	0.113343
O	4.021029	0.034236	-1.277468
O	3.854842	-0.140485	1.600365
O	1.749307	1.513653	0.150498
Mo	0.094188	-2.371253	-0.127559
Mo	0.127155	2.335863	-0.090166
O	0.009110	2.887785	-1.691517
O	0.028530	3.675369	0.954986
O	-1.205940	1.159062	0.278030
O	0.182239	-3.310923	-1.539259
O	-1.488631	-1.431444	-0.123843
O	0.138012	-3.411941	1.212858
O	1.500368	-1.231039	-0.068192
Mo	-2.854257	-0.060307	0.138616
O	-4.041012	-0.766203	-0.864385
O	-3.407598	-0.212160	1.722222
O	-3.429396	1.634567	-0.534026

$[Mo_4O_{13}]^{2-}$ IV 3/4
E = -34057.95

Mo	-3.030287	-0.017944	0.118233
O	-4.025732	-0.032514	-1.268896
O	-3.848167	0.142723	1.608562
O	-1.750667	-1.514522	0.150715
Mo	-0.093733	2.371398	-0.132201
Mo	-0.128849	-2.336293	-0.094679
O	-0.014196	-2.884481	-1.697612
O	-0.029975	-3.678900	0.946481
O	1.208571	-1.164943	0.273498
O	-0.177981	3.302949	-1.549490
O	1.487155	1.428345	-0.118674
O	-0.139580	3.419526	1.202400
O	-1.499529	1.231183	-0.069474
Mo	2.854030	0.059266	0.143655
O	4.044865	0.774341	-0.848955
O	3.400116	0.201183	1.730570
O	3.439025	-1.626130	-0.542919

$[Mo_4O_{13}]^{2-}$ V 1/2
E = -34057.86

Mo	-2.017909	1.894809	-0.223339
O	2.250821	-1.285055	0.128885
O	-2.152822	-2.330153	-1.756042
O	-2.803622	2.561882	1.135542
O	-2.665077	0.276665	-0.610070
Mo	3.581899	-0.138905	-0.380931
Mo	-2.148704	-1.779780	-0.159798
O	-3.918891	-1.473182	0.503778
O	-2.141881	-3.181996	0.815529
O	-2.210806	2.951697	-1.549009
O	2.486914	1.286485	-0.035234
O	4.036058	-0.283264	-2.019188
Mo	0.930542	0.178506	0.750985
O	4.969072	-0.200316	0.613651
O	-0.214526	1.639882	0.153549
O	-0.476129	-1.035349	0.282971
O	1.025292	0.260895	2.404327

$[Mo_4O_{13}]^{2-}$ V 3/4
E = -34057.85

Mo	2.295237	1.761852	0.148378
O	-2.021094	-1.234273	-0.010337
O	3.177187	0.096081	0.363200
O	2.442777	2.689739	1.562502
O	2.981535	2.615738	-1.149337
Mo	-3.578552	0.082534	0.388680
Mo	2.459612	-1.663431	0.235743
O	3.278835	-2.543017	-0.966305
O	2.621656	-2.468233	1.723295
O	-3.582533	0.340658	2.064845
O	-2.178722	1.157559	-0.373939
O	-4.444564	-1.604110	0.021552
Mo	-0.783862	-0.102163	-0.797034
O	-4.952060	0.865339	-0.279302
O	0.599234	1.324585	-0.177610
O	0.756978	-1.402699	-0.189245
O	-0.739516	-0.251025	-2.462104

$[Mo_4O_{13}]^{2-}$ VI 1/2
E = -34057.44

Mo	-2.404935	-1.340211	-0.606620
Mo	-1.654031	1.924639	0.135727
Mo	0.837260	-0.682734	1.069419
O	-2.719765	0.530002	-0.601133
O	-0.850283	-1.532110	0.235809
O	-0.142684	1.135621	0.631495
O	-1.394477	3.138724	-1.020686
O	-2.453688	2.607190	1.471971
O	-3.650675	-2.139503	0.229453
O	-2.359643	-1.925592	-2.198525
O	2.551038	0.364228	1.133033
O	1.605359	-0.656631	-1.065912
Mo	3.099539	0.229083	-0.635806
O	3.260584	1.739336	-1.421809
O	4.538521	-0.674009	-0.856913
O	0.472688	-0.869046	2.686740
O	1.784404	-2.404789	0.972196

$[Mo_4O_{13}]^{2-}$ VI 3/4
E = -34057.49

Mo	-2.418710	-1.343779	-0.596909
Mo	-1.667242	1.923070	0.131172
Mo	0.839437	-0.671801	1.054745
O	-2.745349	0.525229	-0.580129
O	-0.848126	-1.525276	0.214732
O	-0.145910	1.136798	0.600975
O	-1.429643	3.131505	-1.036177
O	-2.442111	2.611163	1.479199
O	-3.644190	-2.154036	0.259205
O	-2.395672	-1.923924	-2.191734
O	2.542599	0.407559	1.123200
O	1.639303	-0.685753	-1.059842
Mo	3.121644	0.224095	-0.627595
O	3.288066	1.712253	-1.454187
O	4.570428	-0.675486	-0.794817

O	0.470067	-0.867415	2.668383
O	1.796110	-2.383441	0.973775

$[Mo_4O_{13}]^{2-}$ VII 1/2
E = -34056.62

Mo	3.750691	0.867301	-0.074099
Mo	1.396384	-0.999473	-0.100117
Mo	-1.718869	-0.915823	0.145773
Mo	-3.933920	1.016109	-0.045287
O	2.124988	0.288907	1.024896
O	2.814468	-0.482460	-1.188270
O	-3.074600	-0.299403	1.229858
O	-2.375356	0.344558	-1.080967
O	-0.000545	-0.897978	-1.198853
O	-0.293304	-0.836977	1.221760
O	1.749688	-2.591882	0.323967
O	-2.132792	-2.509141	-0.208237
O	-5.447659	0.523874	-0.672211
O	4.306072	1.241552	1.728562
O	3.456779	2.352316	-0.819722
O	-3.828537	2.648390	0.447776
O	5.355795	0.385644	-0.421474

$[Mo_4O_{13}]^{2-}$ VII 3/4
E = -34056.62

Mo	-3.750691	-0.867300	-0.074099
Mo	-1.396381	0.999471	-0.100119
Mo	1.718869	0.915821	0.145771
Mo	3.933919	-1.016108	-0.045289
O	-2.124991	-0.288909	1.024901
O	-2.814471	0.482460	-1.188269
O	3.074599	0.299401	1.229861
O	2.375359	-0.344559	-1.080969
O	0.000549	0.897981	-1.198849
O	0.293299	0.836981	1.221761
O	-1.749692	2.591881	0.323971
O	2.132788	2.509141	-0.208239
O	5.447659	-0.523868	-0.672209
O	-4.306071	-1.241550	1.728561
O	-3.456781	-2.352320	-0.819719
O	3.828539	-2.648388	0.447781
O	-5.355791	-0.385640	-0.421469

$[Mo_4O_{13}]^{2-}$ VIII 1/2
E = -34056.49

Mo	-2.521087	-1.866509	-0.230795
O	-3.168092	0.024069	-0.478030
O	-0.796458	-1.152729	0.298721
O	-3.328587	-2.653031	1.054624
O	-2.466974	-2.707408	-1.717376
Mo	-2.532976	1.708289	-0.228995
O	-2.704391	2.618855	-1.653326
O	-3.397071	2.475788	1.017457
O	-0.741656	1.583927	0.231249
Mo	0.432708	0.139833	0.829896
O	0.510115	0.203599	2.505753
O	1.973008	0.072233	-0.165094
O	3.987593	-1.428406	0.804675
O	4.111041	-0.086949	-2.093522
Mo	3.932086	0.095519	-0.413053
O	4.349826	1.666252	0.088223
O	5.290311	-1.021146	0.332118

$[Mo_4O_{13}]^{2-}$ VIII 3/4
E = -34056.39

Mo	2.506033	1.873890	-0.209688
O	3.160250	-0.016702	-0.164097
O	0.700709	1.299483	0.349383
O	3.227210	2.856352	0.988911
O	2.522794	2.497363	-1.799023
Mo	2.547438	-1.724817	-0.202310
O	2.796201	-2.435413	-1.725761
O	3.317673	-2.665677	0.985844
O	0.739872	-1.553736	0.148163
Mo	-0.430122	-0.128721	0.780119
O	-0.538413	-0.320114	2.444453
O	-1.953998	-0.055553	-0.253852
O	-3.969076	1.097371	1.125012
O	-4.158782	0.521679	-2.010789
Mo	-3.915212	-0.072396	-0.436356
O	-4.284781	-1.729705	-0.331660
O	-5.277380	0.777884	0.601648

$[CH_2O_2]$ I 1/2
E = -5159.39

C	0.728185	0.000002	-0.000001
O	-0.435662	-0.734371	-0.000002
O	-0.435667	0.734369	-0.000001
H	1.300785	0.000004	-0.927022
H	1.300738	0.000003	0.927051

$[CH_2O_2]$ I 3/4
E = -5157.88

C	0.000000	-0.625618	0.000002
O	-0.940771	0.390179	0.000002
O	0.940770	0.390181	0.000002
H	0.000004	-1.244549	-0.907547
H	0.000004	-1.244620	0.907501

$[Mo_2O_8]^{2-}$ I 1/2
E = -20098.31

Mo	2.018499	0.020559	0.000010
Mo	-1.772003	-0.191211	0.000022
O	-1.737771	1.762541	0.000007
O	0.130168	-0.201327	0.000370
O	-2.199326	-1.063617	-1.407016
O	-2.199870	-1.063276	1.407105
O	2.517966	0.884525	-1.407374
O	2.764806	-1.536531	0.000204
O	-3.088426	1.228461	-0.000363
O	2.518346	0.885149	1.406897

$[Mo_2O_8]^{2-}$ I 3/4
E = -20096.30

Mo	-1.996431	0.001045	0.000034
Mo	1.791174	-0.279718	-0.000223
O	2.124155	1.792060	0.659253
O	-0.109458	0.058002	-0.001240
O	2.338283	-1.067666	1.428127
O	2.341667	-1.064680	-1.428942
O	-2.597565	0.661537	1.480358
O	-2.539161	-1.633473	-0.151493
O	2.126038	1.792660	-0.656672
O	-2.606360	0.924595	-1.328403

$[Mo_2O_8]^{2-}$ II 1/2
E = -20096.19

Mo	1.562489	0.099518	-0.011757
Mo	-1.562433	-0.099530	0.011786
O	-0.153340	-0.854592	-0.880670
O	-2.844504	-0.156489	-1.144965
O	-2.048046	-1.086198	1.320497
O	2.047870	1.086333	-1.320425
O	2.050168	-1.764284	-0.288464
O	-2.050513	1.764123	0.288210
O	2.844591	0.156456	1.144954
O	0.153480	0.854710	0.880710

$[Mo_2O_8]^{2-}$ II 3/4
E = -20096.19

Mo	1.562491	0.099682	-0.011534
Mo	-1.562470	-0.099670	0.011530
O	-0.154955	-0.849159	-0.886347
O	-2.847316	-0.152637	-1.142290
O	-2.045408	-1.091747	1.317177
O	2.045494	1.091646	-1.317240
O	2.050343	-1.763846	-0.293086
O	-2.050655	1.763795	0.293146
O	2.847338	0.152502	1.142333
O	0.155046	0.849383	0.886330

$[Mo_2O_8]^{2-}$ III 1/2
E = -20095.78

Mo	-1.461567	0.001695	0.005065
Mo	1.619393	-0.000716	0.001295
O	0.390326	-0.120015	1.326307
O	2.636573	-1.392921	-0.116816
O	-0.977524	-1.847801	0.316029
O	-0.953662	1.843701	-0.330425
O	-2.526447	0.443920	1.263169
O	2.632286	1.390771	0.159096
O	-2.484448	-0.441600	-1.289243
O	0.454307	0.118808	-1.361507

$[Mo_2O_6]^{2-}$ I 1/2
E = -16003.98

Mo	-1.293334	-0.000009	-0.000023
Mo	1.293292	-0.000007	0.000025
O	0.000139	0.000003	-1.459863
O	0.000016	0.000005	1.459867
O	-2.194556	-1.486643	0.000024
O	-2.194436	1.486692	0.000004
O	2.194483	1.486624	-0.000011
O	2.194578	-1.486597	-0.000029

$[Mo_2O_6]^{2-}$ I 3/4
E = -16003.73

Mo	-1.522558	-0.000056	-0.000003
Mo	1.522557	-0.000045	0.000003
O	0.000010	-0.000367	-1.226491
O	-0.000017	-0.000746	1.226491
O	-2.438950	-1.475411	-0.000074
O	-2.438196	1.475770	0.000078
O	2.437404	1.476228	0.000080
O	2.439754	-1.474939	-0.000083

$[HMo_3O_{10}]^{-}$ I 1/2
E = -26073.63

Mo	2.324310	-0.878157	0.023527
O	1.477853	0.886876	0.152700
O	-1.351960	-2.080932	-0.982659
O	1.017623	-2.030321	-0.040274
O	3.309870	-1.155511	1.378401
Mo	0.001232	1.946197	-0.012236
O	-0.150188	2.953160	1.324862
O	0.119269	2.917941	-1.378808
O	-1.506619	0.831502	-0.163398
Mo	-2.308404	-0.869182	0.008909
O	-3.890788	-0.835321	-0.556609
O	-2.328048	-1.299866	1.632798
O	3.263530	-0.951407	-1.389725
H	-0.404108	-2.240978	-0.666697

$[HMo_3O_{10}]^{-}$ I 3/4
E = -26070.68

Mo	2.101320	-0.698653	-0.034054
O	1.405719	1.036128	0.259581
O	-0.120887	-1.109414	-0.699322
O	2.502627	-1.001699	-1.641625
O	1.413124	-2.213099	0.854703
Mo	-0.243535	1.934088	0.070816
O	-0.249184	3.317144	1.035810
O	-0.478523	2.399616	-1.532151
O	-1.551338	0.793990	0.614446
Mo	-2.040818	-1.063653	0.033701
O	-3.169224	-1.111346	-1.235198
O	-2.341608	-2.115619	1.328017
O	3.552922	-0.688532	0.829655
H	-0.021627	-1.672213	-1.470757

$[HMo_3O_{10}]^{-}$ II 1/2
E = -26073.46

Mo	-0.854565	-1.695387	0.035991
O	1.008523	-1.537973	0.432785
O	-1.836747	-0.104682	0.432464
O	-1.261625	-2.503350	-1.382409
O	-1.367970	-2.712432	1.273708
Mo	1.895895	0.107882	0.035790
O	2.801760	0.158787	-1.380805
O	0.000685	0.000639	-1.266272

Mo	-1.041115	1.587695	0.035801
O	0.827648	1.642674	0.432685
O	-1.665156	2.538003	1.275436
O	3.030311	0.171370	1.276214
O	-1.538416	2.346291	-1.381088
H	-0.001119	-0.002575	-2.220164

$[HMo_3O_{10}]^-$ II 3/4
E = -26070.61

Mo	-2.128973	0.008002	0.166279
O	-0.722686	-1.381929	0.400533
O	-0.713126	1.388871	0.393960
O	-2.726216	0.005588	-1.442776
O	-3.273877	0.014589	1.413064
Mo	1.044052	-1.712059	0.103727
O	1.287883	-2.578369	-1.320948
O	-0.130536	-0.007859	-2.567943
Mo	1.056906	1.705602	0.101446
O	1.866002	-0.006285	0.050621
O	1.701555	2.599945	1.375257
O	1.684843	-2.610833	1.376419
O	1.311780	2.568926	-1.323057
H	-1.108368	-0.006045	-2.442022

$[HMo_3O_{10}]^-$ III 1/2
E = -26073.14

Mo	-1.637386	0.867104	-0.043176
O	-1.743709	-0.985752	0.338771
O	0.019149	2.224073	0.013915
O	-2.750400	1.323559	-1.215263
O	-2.099899	1.567964	1.428112
Mo	-0.016101	-1.840321	0.027725
O	-0.022986	-2.810684	-1.340717
O	0.001257	0.124908	-0.917863
Mo	1.652245	0.838944	-0.042945
O	1.724836	-1.014807	0.342105
O	2.128393	1.534295	1.426608
O	-0.026106	-2.882878	1.350580
O	2.772946	1.272555	-1.216260
H	0.024274	2.813582	0.772750

$[HMo_3O_{10}]^-$ III 3/4
E = -26070.57

Mo	-1.695541	0.809344	-0.052136
O	-1.810609	-1.012827	0.337742
O	0.000547	2.093405	0.091232
O	-2.729666	1.236740	-1.304828
O	-2.261909	1.569870	1.349299
Mo	-0.000744	-1.761674	-0.053712
O	-0.000703	-3.094816	-1.055012
O	0.000168	0.031349	-0.942406
Mo	1.696459	0.807907	-0.051818
O	1.810244	-1.014256	0.337179
O	2.262447	1.567618	1.350344
O	-0.002918	-2.192869	1.864584
O	2.731443	1.235444	-1.303966
H	0.000306	2.708512	0.828643

$[HMo_3O_{10}]^-$ IV 1/2
E = -26072.87

Mo	0.108018	1.725603	-0.092436
O	-1.694200	0.930278	0.475817
O	1.697176	0.871369	0.543430
O	0.338834	3.059915	-1.072024
O	-0.247867	2.617342	1.563673
Mo	-1.720631	-0.868052	-0.012573
O	-2.488481	-1.191631	-1.473935
O	-0.081801	0.380105	-1.231069
Mo	1.665580	-0.971394	0.052610
O	-0.025776	-1.761495	0.277716
O	2.643418	-1.726760	1.190512
O	-2.685496	-1.580688	1.169701
O	2.376801	-1.276054	-1.437223
H	-0.885473	2.202349	2.147936

$[HMo_3O_{10}]^-$ IV 3/4
E = -26070.72

Mo	-0.087345	-1.738494	-0.093600
O	1.709101	-1.045899	0.399246
O	-1.816348	-0.863247	0.375125
O	-0.134970	-2.889688	-1.293502
O	-0.198924	-2.656061	1.593376
Mo	1.771128	0.772702	0.001235
O	2.878110	1.104471	-1.218689
O	0.016138	0.121685	-0.994010
Mo	-1.662684	0.956620	-0.022670
O	0.111326	1.982184	-0.067825
O	-2.175339	1.719164	1.394370
O	2.352608	1.487834	1.415745
O	-2.717669	1.414921	-1.247170
H	-1.078412	-2.617680	1.978134

$[HMo_3O_{10}]^-$ V 1/2
E = -26072.76

Mo	-0.834900	-1.636925	-0.015247
O	1.039604	-1.748679	0.239357
O	-2.311918	-0.027466	-0.140309
O	-1.390343	-2.807277	-1.094072
O	-1.410685	-1.962362	1.532283
Mo	1.849923	0.022794	-0.011366
O	2.885163	0.037296	-1.332753
O	-0.092598	-0.001392	-0.905619
Mo	-0.876322	1.615410	-0.015089
O	0.995423	1.772213	0.241692
O	-1.460720	1.930537	1.531418
O	2.811594	0.033663	1.371568
O	-1.457560	2.770724	-1.096482
H	-2.689130	-0.031770	-1.025179

$[HMo_3O_{10}]^-$ VI 1/2
E = -26072.45

Mo	-3.180419	-0.528077	-0.017382
O	-1.197392	-0.024343	-0.096538
O	4.508209	0.144059	0.445948
O	-3.610621	-0.704230	1.624291
O	-3.416523	-1.984480	-0.871053
Mo	0.140071	1.106279	0.103277
O	0.765535	1.685323	-1.525055
O	-0.380946	2.436451	0.951181
O	1.501130	0.321098	0.966048
Mo	3.041525	-0.611782	0.045743
O	2.588135	-0.311306	-1.602530
O	3.107502	-2.268754	0.396796
O	-4.049151	0.750585	-0.741068
H	1.423556	1.055160	-1.912955

$[HMo_3O_{10}]^-$ VI 3/4
E = -26070.03

Mo	3.207117	-0.555484	-0.059078
O	1.501570	0.080397	-0.860392
O	-4.412418	0.835708	-0.138061
O	4.535831	0.034316	-0.949882
O	3.236046	-2.259461	-0.005449
Mo	0.144797	1.052591	-0.113102
O	0.426866	0.886285	1.737963
O	0.090342	2.683558	-0.475632
O	-1.603376	0.160145	-0.083700
Mo	-3.315703	-0.443904	-0.058303
O	-3.710115	-1.393457	1.531832
O	-3.588167	-1.528841	-1.323357
O	3.162011	0.125266	1.530244
H	1.370418	0.774145	1.971733

$[HMo_3O_{10}]^-$ VII 1/2
E = -26072.44

Mo	-1.833280	0.000012	0.092130
O	-1.019748	-1.544564	0.566879
O	-1.020148	1.544566	0.567217
O	-2.649429	0.000222	-1.614293
O	-3.235104	-0.001276	1.028248
Mo	0.983099	-1.515606	-0.030294
O	1.386933	-2.605632	-1.243519
O	-0.001258	-0.000035	-0.988749
Mo	0.982799	1.515719	-0.030218
O	2.160303	0.000192	0.071183
O	1.373321	2.240757	1.445942
O	1.373654	-2.240614	1.445904
O	1.386298	2.605857	-1.243410
H	-3.608496	-0.001060	-1.611150

$[HMo_3O_{10}]^-$ VII 3/4
E = -26069.72

Mo	0.002877	-1.714224	-0.034149
O	1.732601	-1.008189	0.367831
O	-1.728672	-1.012862	0.369151
O	0.004580	-3.332312	-0.974845
O	0.004111	-1.833501	1.873810
Mo	1.636938	0.898293	-0.022921
O	2.433744	1.367100	-1.426268
O	0.000481	-0.193575	-1.101727
Mo	-1.640040	0.893362	-0.022605
O	-0.003019	1.835111	0.313331
O	-2.593316	1.465031	1.244971
O	2.587258	1.471739	1.246068
O	-2.437395	1.357961	-1.427034
H	0.006470	-4.184125	-0.535948

$[Mo_2O_7]^{2-}$ I 1/2
E = -18053.67

O	-2.488477	-0.307114	-1.593904
O	2.485810	0.411516	1.570806
Mo	-1.902201	0.000289	-0.000071
O	2.485500	-1.567187	-0.427237
O	-2.487032	1.533541	0.533112
Mo	1.902367	0.000112	-0.000162
O	-2.485041	-1.228550	1.062310
O	2.488339	1.153697	-1.142353
O	0.000027	0.001990	-0.001513

$[Mo_2O_7]^{2-}$ I 3/4
E = -18050.27

O	-2.493636	-0.601296	1.480538
O	2.687517	0.508413	-1.493601
Mo	-1.880757	0.034147	-0.004430
O	2.638412	0.761117	1.406762
O	-2.535002	-0.865206	-1.326995
Mo	1.940934	0.059057	-0.005476
O	-2.364631	1.687975	-0.152013
O	1.742110	-1.917107	0.162609
O	0.009299	-0.063218	-0.025295

$[CH_3O]$ I 1/2
E = -3129.96

C	0.570291	0.000067	0.013477
H	1.002236	0.908987	0.453855
H	1.002052	-0.907169	0.457555
H	0.866253	-0.002399	-1.052729
O	-0.786536	0.000022	0.007557

$[MoO_4]^-$ I 1/2
E = -10049.16

Mo	-0.042668	-0.000040	0.006635
O	0.263474	-0.010610	1.709038
O	1.733952	0.004292	-0.761301
O	-0.887110	-1.409309	-0.499995
O	-0.886308	1.415838	-0.482574

$[MoO_4]^-$ I 3/4
E = -10045.67

Mo	0.112685	0.000008	-0.000018
O	-1.232515	-0.000205	1.490858
O	-1.231839	0.000007	-1.491287
O	0.936250	1.512011	0.000366
O	0.936509	-1.511857	0.000157

$[MoO_4]^-$ II 1/2
E = -10038.65

Mo	0.152469	-0.137317	0.117060
O	1.489058	1.240992	0.013125
O	2.104472	-0.647811	-0.422013

O	-1.915822	-0.103397	0.471541
O	-2.478171	0.231131	-0.677216

$[MoO_4]^-$ II 3/4
E = -10045.67

Mo	0.329285	0.005681	-0.000130
O	0.933778	1.613309	0.000226
O	1.442836	-1.302538	0.000327
O	-1.438857	-0.444055	-0.000095
O	-2.666505	0.103460	0.000223

$[MoO_4]^-$ II 5/6
E = -10045.27

Mo	0.340625	-0.000023	-0.053931
O	1.114477	1.526981	0.169023
O	1.116128	-1.526280	0.168470
O	-1.661518	0.000589	-0.586469
O	-2.357370	-0.001170	0.532115

$[MoO_4]^-$ II 7/8
E = -10041.83

Mo	0.385024	0.001963	-0.189183
O	1.476405	1.136464	0.470620
O	0.135981	-1.901966	0.326671
O	-1.777550	-0.272209	0.086844
O	-1.856214	1.027405	0.109075

$[HMoO_4]^-$ I 1/2
E = -10067.57

Mo	-0.067334	-0.002623	0.001043
O	-0.571282	0.744804	-1.464183
O	1.857973	-0.303286	-0.066310
O	-0.406073	1.041589	1.326167
O	-0.825464	-1.531019	0.202034
H	2.386774	0.493462	-0.025469

$[HMoO_4]^-$ I 3/4
E = -10064.38

Mo	-0.168443	-0.000090	0.006594
O	-1.056575	-1.468158	0.066179
O	1.431888	0.070612	1.146966
O	1.333590	-0.028461	-1.319406
O	-1.104816	1.437972	-0.009668
H	2.241896	-0.091916	0.650492

$[HMoO_4]^-$ II 1/2
E = -10062.54

Mo	0.341016	0.004939	0.140862
O	0.754202	1.588031	-0.332487
O	1.384508	-1.209217	-0.455328
O	-1.393633	-0.475110	0.011709
O	-2.609285	0.073769	-0.194881
H	0.591015	-0.027233	1.851676

$[HMoO_4]^-$ II 3/4
E = -10062.15

Mo	0.352165	0.000005	0.142570
O	0.948019	1.491264	-0.456600
O	0.948472	-1.491051	-0.456636
O	-1.659649	-0.000078	0.572828
O	-2.176117	-0.000156	-0.637454
H	0.723279	-0.000068	1.834948

$[HMoO_4]^-$ III 1/2
E = -10062.18

Mo	0.375612	-0.010562	0.000014
O	0.577459	1.698238	0.000025
O	1.773125	-0.998970	-0.000105
O	-1.412673	-0.753956	0.000145
O	-2.620770	-0.003595	-0.000096
H	-2.312851	0.909876	-0.000333

$[HMoO_4]^-$ III 3/4
E = -10062.14

Mo	0.379845	-0.001007	-0.070155
O	1.319382	1.408408	0.282991
O	0.936919	-1.624537	0.172176
O	-1.508573	0.199360	-0.653001
O	-2.415647	0.124253	0.503084
H	-2.610128	-0.817561	0.504530

$[HMoO_4]^-$ IV 1/2
E = -10061.52

Mo	0.289444	-0.062587	-0.000069
O	1.502903	1.432749	0.000098
O	0.903968	-1.634930	0.000146
O	-1.474125	-0.010254	-0.000147
O	-2.759310	0.368929	0.000225
H	2.455854	1.376692	0.000310

$[Mo_2O_5]^-$ I 1/2
E = -13949.63

Mo	-1.579842	0.012252	-0.003939
Mo	1.315043	-0.054988	-0.009239
O	-0.015743	-0.984544	0.881524
O	0.031582	1.019231	-0.812920
O	-3.645126	0.018604	-0.004303
O	2.479596	-1.109342	-0.706293
O	2.539886	1.280411	0.711177

$[Mo_2O_5]^-$ I 3/4
E = -13956.34

Mo	-1.545550	-0.000278	-0.000003
Mo	1.303367	-0.000175	-0.000003
O	-0.020036	-0.002567	1.300922
O	-0.020025	-0.000120	-1.300926
O	-3.233568	0.000358	0.000011
O	2.276344	-1.400366	-0.001122
O	2.268748	1.405074	0.001147

$[Mo_2O_5]^-$ I 5/6
E = -13956.32

Mo	-1.577479	-0.186827	-0.000007
Mo	1.290836	0.042174	-0.000001
O	0.016209	-0.162384	1.314761
O	0.016234	-0.162523	-1.314769
O	-3.029737	0.691501	0.000030
O	2.473903	-1.188205	0.000086
O	2.028269	1.581038	-0.000068

$[Mo_2O_5]^-$ I 7/8
E = -13953.21

Mo	1.538307	-0.000131	0.000000
Mo	-1.327113	0.000005	0.000000
O	-0.052920	-0.000311	1.324252
O	-0.052918	-0.000316	-1.324251
O	3.598077	0.000356	-0.000000
O	-2.299820	1.405287	-0.000003
O	-2.301190	-1.404353	0.000001

$[HMo_4O_{12}]^-$ I 1/2
E = -32027.89

Mo	-0.000040	2.194452	0.085745
O	-3.027868	-0.199835	1.453291
O	-0.000118	2.397773	1.752115
O	-0.000005	3.740971	-0.558505
O	1.611445	1.460040	-0.556148
Mo	-2.604009	-0.113789	-0.175318
Mo	2.604080	-0.113736	-0.175313
O	4.032352	-0.002556	-1.055344
O	3.027825	-0.199880	1.453318
O	-1.611443	1.460038	-0.556291
O	-0.000187	-0.295927	0.840286
O	-4.032236	-0.002618	-1.055419
Mo	0.000017	-2.223720	0.022779
O	-1.744432	-1.640286	-0.734453
O	1.744386	-1.640145	-0.734503
O	0.000064	-3.701189	0.797990
H	-0.000240	-0.225757	1.797782

$[HMo_4O_{12}]^-$ I 3/4
E = -32028.19

Mo	0.000037	2.193167	0.075369
O	-3.019745	-0.190136	1.465772
O	-0.000010	2.402213	1.740889
O	0.000031	3.737098	-0.574963
O	1.612010	1.455404	-0.561360
Mo	-2.607854	-0.113058	-0.166403
Mo	2.607857	-0.113105	-0.166396
O	4.042376	-0.001939	-1.036066
O	3.019811	-0.190283	1.465757
O	-1.611895	1.455361	-0.561438
O	-0.000072	-0.299315	0.837904
O	-4.042389	-0.001829	-1.036036
Mo	-0.000041	-2.221125	0.015268
O	-1.755075	-1.642596	-0.724869
O	1.754968	-1.642567	-0.724923
O	-0.000016	-3.718979	0.756292
H	0.000090	-0.226400	1.795174

$[HMo_4O_{12}]^-$ II 1/2
E = -32026.97

Mo	-2.300929	-1.993791	0.076662
O	-2.183424	-2.309488	1.721820
O	-0.847659	-2.610870	-0.818604
O	-3.681266	-2.743149	-0.522918
Mo	-2.096661	1.718925	-0.110011
Mo	1.631236	2.099974	0.118561
O	2.373411	3.586456	0.205856
O	2.682466	0.610206	0.053479
O	-0.271846	2.026249	0.051111
O	-2.905205	2.401333	1.191323
O	-2.676522	2.445814	-1.506281
O	-2.413597	-0.108094	-0.176044
Mo	2.942955	-1.327521	-0.060329
O	3.593733	-1.699942	-1.581789
O	4.013254	-1.826623	1.157055
H	0.110392	-2.481035	-0.442616
O	1.375702	-2.074092	0.149683

$[HMo_4O_{12}]^-$ II 3/4
E = -32028.09

Mo	-0.747626	1.883361	-0.622063
O	0.051920	0.403698	-1.239367
O	0.257215	2.673760	0.508218
O	-1.137685	2.944258	-1.869640
Mo	-2.763214	-0.530938	0.789532
Mo	0.261104	-1.628442	-0.937285
O	0.374635	-2.750876	-2.164282
O	1.886810	-1.353881	0.140920
O	-1.492896	-1.587790	0.036163
O	-4.304211	-0.952973	0.254855
O	-2.721361	-0.640776	2.470609
O	-2.299813	1.222670	0.229821
Mo	3.059321	-0.009441	0.598378
O	4.401321	-0.664934	1.380074
O	3.572935	0.798838	-0.788611
H	1.594240	1.848519	1.416411
O	2.211528	1.175609	1.764242

$[HMo_4O_{12}]^-$ III 1/2
E = -32027.96

Mo	0.103968	-2.358885	0.437780
O	3.726635	-0.025163	0.312782
O	0.116109	-2.145161	2.102663
O	-0.125771	-3.997411	0.153927
O	-1.417696	-1.487425	-0.338439
Mo	2.258568	-0.016279	-0.505900
Mo	-2.502052	0.017570	-0.436689
O	-3.348597	0.022351	-1.886261
O	-3.618463	0.023879	0.815889
O	1.728795	-1.912182	-0.293776
O	2.430782	-0.017510	-2.181219
Mo	0.137538	2.357610	0.437479
O	1.755286	1.887349	-0.294069
O	-0.067824	3.999383	0.152897
O	-1.398055	1.508030	-0.336713

O	0.147853	2.144328	2.102406
H	0.650603	-0.004427	0.347142

$[HMo_4O_{12}]^-$ III 3/4

E = -32027.86

Mo	0.053405	-2.036671	-0.487116
O	-4.118858	-0.047758	-0.624832
O	0.767331	-0.578352	-1.527226
O	0.345058	-3.520868	-1.208145
O	1.433470	-1.514301	0.777249
Mo	-2.857581	-0.155925	0.504844
Mo	2.603679	-0.071929	0.587588
O	3.415719	0.087298	2.047200
O	3.770931	-0.350105	-0.587423
O	-1.628575	-1.713726	0.058127
O	-3.304872	-0.111942	2.139919
Mo	0.024411	2.130745	-0.448779
O	-1.314082	1.049897	0.108594
O	-0.232756	3.684289	0.142729
O	1.669389	1.526682	0.285879
O	0.064213	2.232562	-2.133756
H	0.459832	-0.330690	-2.401109

$[HMo_4O_{12}]^-$ IV 1/2

E = -32027.83

Mo	0.078595	2.454291	0.275643
Mo	-2.735657	0.042837	-0.377068
Mo	-0.014370	-2.447430	0.272574
Mo	2.660253	-0.045774	-0.240451
O	1.677495	-1.527020	0.282564
O	-0.378165	-2.924919	1.852739
O	-1.201338	1.328164	-0.250935
O	-3.763456	0.016065	1.213921
O	4.173273	-0.072994	0.491359
O	1.742493	1.484347	0.258593
O	-1.245982	-1.299362	-0.317366
O	-3.438136	0.081864	-1.883280
O	0.078404	-3.799880	-0.733665
O	2.888689	-0.063275	-1.904982
O	-0.222474	2.979266	1.853613
O	0.180351	3.780337	-0.763994
H	-3.459732	-0.025533	2.122128

$[HMo_4O_{12}]^-$ IV 3/4

E = -32027.94

Mo	0.376034	2.527600	0.081698
Mo	-2.698724	0.401293	-0.164636
Mo	-0.356119	-2.431627	0.015686
Mo	2.653440	-0.397941	-0.053847
O	1.414260	-1.738458	0.276449
O	-1.005453	-2.926623	1.499494
O	-1.155917	1.691097	-0.338635
O	-3.016370	-0.411394	1.553438
O	3.887148	-0.467225	1.082090
O	1.788828	1.239724	0.025945
O	-1.349942	-1.111613	-0.647390
O	-4.079442	0.762626	-1.045752
O	-0.326613	-3.741104	-1.047082
O	3.341788	-0.605394	-1.570601
O	0.290172	3.171128	1.636387
O	0.681314	3.778626	-1.004065
H	-2.692665	-1.302801	1.723926

$[HMo_4O_{12}]^-$ V 1/2

E = -32027.84

Mo	0.550231	-2.357670	-0.147041
O	2.575060	-0.140520	1.744019
O	0.967301	-2.635230	1.477479
O	0.766161	-3.770070	-1.042291
O	-1.144809	-1.763910	-0.276291
Mo	2.553800	0.521590	-0.019811
Mo	-2.716640	-0.569890	0.064249
O	-3.808170	-0.582080	-1.233051
O	-3.398540	-0.786330	1.600879
O	1.698960	-1.008130	-0.774991
O	4.110210	0.644481	-0.610651
Mo	-0.465870	2.482930	-0.062411
O	1.379970	1.969660	-0.147541
O	-0.852060	3.378880	-1.435401
O	-1.441120	0.974120	0.020479
O	-0.711580	3.439960	1.301639
H	2.165010	-0.999010	1.936449

$[HMo_4O_{12}]^-$ V 3/4

E = -32027.84

Mo	0.534087	-2.363722	-0.127620
O	2.582143	-0.140908	1.727570
O	0.965995	-2.629199	1.494993
O	0.747617	-3.783052	-1.012198
O	-1.165225	-1.780883	-0.247364
Mo	2.553667	0.510182	-0.040095
Mo	-2.720693	-0.550930	0.048774
O	-3.767895	-0.534553	-1.284647
O	-3.458340	-0.759760	1.560427
O	1.674749	-1.017163	-0.774707
O	4.106949	0.613701	-0.642016
Mo	-0.445879	2.480699	-0.045647
O	1.401784	1.976176	-0.163622
O	-0.856554	3.378799	-1.409986
O	-1.415616	0.968101	0.050041
O	-0.673192	3.433193	1.324623
H	2.171008	-0.997211	1.927774

$[HMo_4O_{12}]^-$ VI 1/2

E = -32027.80

Mo	0.284953	2.009839	-0.592472
O	0.554553	2.331439	-2.214886
O	0.266048	3.448978	0.269761
O	-1.602551	1.564805	-0.554629
Mo	2.266879	-0.156521	0.563460
Mo	-2.293691	0.214974	0.526633
O	-1.973082	0.509445	2.150608
O	-3.960900	0.265512	0.342331
O	2.211185	1.541264	-0.033802
O	0.323859	0.097627	0.161925
O	2.959318	-0.116240	2.080293
Mo	-0.059095	-2.155196	-0.505803
O	1.628468	-1.930678	0.372897
O	-0.258268	-3.814513	-0.368339
O	-1.767373	-1.529735	0.043712
O	0.136669	-1.837557	-2.139876
H	3.496660	-0.592848	-0.536314

$[HMo_4O_{12}]^-$ VI 3/4

E = -32025.91

Mo	-2.331886	0.167142	0.482488
O	-2.347660	-0.064795	2.147373
O	-3.875084	0.556640	-0.050986
O	-1.376916	1.860237	0.353528
Mo	-0.389233	-2.342588	-0.413042
Mo	0.221637	2.346831	-0.436412
O	0.091747	2.377061	-2.109046
O	0.582944	3.909465	0.056321
O	-2.253423	-1.765299	-0.041574
O	-0.818869	-0.415835	-0.751659
O	-0.311007	-3.445757	-1.647078
Mo	2.559277	-0.387479	0.418561
O	1.440433	-1.779468	0.084541
O	3.921641	-0.485216	-0.563701
O	1.627421	1.220945	0.100232
O	3.066459	-0.426475	2.023534
H	-0.492857	-3.256056	1.021127

$[HMo_4O_{12}]^-$ VII 1/2

E = -32026.44

Mo	-3.489760	-1.501506	-0.340532
O	-5.023331	-2.022046	-0.695006
O	-2.296101	-2.773582	0.249886
Mo	-2.021696	1.854947	-0.218897
Mo	0.826516	-0.043269	1.033620
O	1.037993	0.267379	2.666421
O	2.390223	-0.052162	0.188639
O	-0.327185	1.273460	0.293707
O	-2.699409	2.814171	0.975449
O	-1.931268	2.766993	-1.619369
O	-3.085488	0.345756	-0.496099
Mo	4.265877	-0.269305	-0.535014
O	4.703648	1.196231	-1.295397
O	5.312317	-0.612057	0.770181
H	-1.378062	-2.574922	0.528315
O	4.261613	-1.555877	-1.657413
O	0.029326	-1.540955	0.872282

$[HMo_4O_{12}]^-$ VII 3/4

E = -32026.94

Mo	-2.491610	-1.836439	-0.306961
O	-2.957018	-2.790378	-1.594132
O	-2.623210	-2.664422	1.422486
Mo	-2.335592	1.830148	-0.207904
Mo	0.491986	0.100186	0.726688
O	0.605269	0.013717	2.391106
O	2.097813	0.091143	-0.011570
O	-0.481231	1.608989	0.265690
O	-3.187184	2.515263	1.071039
O	-2.522784	2.793646	-1.571560
O	-2.866525	0.129235	-0.511806
Mo	4.103021	-0.058349	-0.330479
O	4.669799	1.470629	-0.834399
O	4.837833	-0.526164	1.137065
H	-1.814004	-2.839247	1.909016
O	4.361638	-1.234687	-1.538938
O	-0.488622	-1.238687	0.159334

$[MoO_3]^-$ I 1/2

E = -7992.37

Mo	0.163696	-0.091196	-0.000117
O	-1.293047	1.185225	0.000090
O	-1.814632	-0.787418	0.000236
O	2.248275	0.080974	0.000287

$[MoO_3]^-$ I 3/4

E = -8002.08

Mo	-0.000092	-0.000033	-0.116213
O	1.092680	1.303483	0.203317
O	0.582998	-1.597556	0.203372
O	-1.675196	0.294245	0.203428

$[MoO_3]^-$ I 5/6

E = -7999.13

Mo	-0.060565	0.000218	0.000033
O	-0.819135	1.559214	-0.000062
O	1.976997	-0.011809	-0.000047
O	-0.839897	-1.548550	-0.000062

$[MoO_3]^-$ I 7/8

E = -7995.59

Mo	-0.000024	0.005276	0.000240
O	0.000488	-1.730501	-0.000413
O	-1.855852	0.851090	-0.000423
O	1.855488	0.851713	-0.000423

$[Mo_3O_{10}]^-$ I 1/2

E = -26054.87

Mo	1.931572	0.000551	0.038577
O	0.962546	1.584429	0.394374
O	0.963594	-1.584003	0.394159
O	2.622070	0.000897	-1.492986
O	3.211971	0.000919	1.125220
Mo	-0.926925	1.611618	-0.013891
O	-1.325455	2.845439	-1.078174
O	-0.317142	-0.000133	-1.125851
Mo	-0.925922	-1.612161	-0.013907
O	-2.128473	-0.000622	-0.201199
O	-1.538545	-2.023327	1.502989
O	-1.539970	2.022679	1.502878
O	-1.323898	-2.846316	-1.077994

$[Mo_3O_{10}]^-$ I 3/4

E = -26052.11

Mo	-0.000432	1.728459	-0.068871
O	-1.787919	0.990562	0.366928

O	1.787376	0.991233	0.367183
O	-0.000290	3.024802	-1.113264
O	-0.001648	2.288609	1.814726
Mo	-1.744215	-0.835701	-0.020211
O	-2.770432	-1.232133	-1.288184
O	0.000027	-0.083379	-1.018324
Mo	1.744717	-0.834926	-0.020054
O	0.000480	-1.891195	0.012344
O	2.362858	-1.580207	1.359832
O	-2.361982	-1.581180	1.359750
O	2.771159	-1.230724	-1.288027

$[Mo_3O_{10}]^-$ II 1/2

E = -26054.55

Mo	2.049985	-0.000629	-0.198273
O	0.588564	1.386482	-0.102415
O	0.588027	-1.386987	-0.099366
O	2.719101	-0.002283	-1.721573
O	3.164397	0.721992	1.163198
Mo	-1.184160	1.722315	0.026686
O	-1.550164	2.555631	1.446945
O	-1.734260	2.622423	-1.289789
O	-1.975991	0.000505	0.042110
Mo	-1.185110	-1.721740	0.026989
O	-1.733964	-2.620733	-1.290755
O	-1.553617	-2.555724	1.446216
O	3.164150	-0.721020	1.164567

$[Mo_3O_{10}]^-$ II 3/4

E = -26052.58

Mo	1.962570	-0.590684	-0.240782
O	0.889060	1.178214	-0.319091
O	0.220959	-1.591149	-0.140859
O	2.966393	-1.212077	-1.429942
O	3.481276	0.402173	0.929512
Mo	-0.735295	1.910319	-0.094718
O	-0.842438	2.729144	1.379655
O	-1.101509	2.984500	-1.347251
O	-1.913381	0.413324	-0.102178
Mo	-1.570546	-1.432199	0.091153
O	-2.418327	-2.333570	-1.056516
O	-2.022333	-1.953680	1.632387
O	2.542468	-0.025917	1.737103

$[Mo_3O_{10}]^-$ III 1/2

E = -26054.41

Mo	3.209210	-0.659796	-0.009221
O	1.491835	0.378248	-0.098984
O	-3.420623	-1.346093	-1.525739
O	2.922724	-2.185126	-0.726360
O	3.647563	-0.842555	1.632162
Mo	0.009000	1.371142	0.011842
O	0.008422	2.301504	1.411515
O	-0.117137	2.410852	-1.302833
O	-1.534986	0.217385	0.037816
Mo	-3.162735	-0.628718	-0.027181
O	-4.416460	0.442499	0.306935
O	-3.291367	-2.004433	1.257053
O	4.418782	0.193922	-0.862626

$[Mo_3O_{10}]^-$ III 3/4

E = -26051.82

Mo	-2.605127	-0.855159	-0.000008
O	-1.416408	0.498195	-0.000193
O	2.474029	-1.804393	1.393853
O	-2.472043	-1.805454	1.392897
O	-2.474111	-1.804180	-1.394009
Mo	-0.000009	1.895138	-0.000003
O	0.000126	2.799821	-1.435371
O	-0.000049	2.799832	1.435360
O	1.416241	0.498039	0.000183
Mo	2.605130	-0.855170	-0.000006
O	4.382721	-0.173306	-0.000965
O	2.472311	-1.805305	-1.393052
O	-4.382785	-0.173494	0.001387

$[CH_3Mo_2O_7]$ I 1/2

E = -19133.78

O	2.736860	-1.417600	0.105661
O	2.570092	0.819029	-1.465066
Mo	2.078345	0.116338	-0.025430
O	0.214549	0.059581	0.076284
O	-2.604161	1.058444	-0.561204
O	-2.146512	-0.833847	1.541145
Mo	-1.623307	-0.391579	0.015702
O	2.806130	1.154934	1.362662
O	-1.866610	-1.681588	-1.019390
C	-3.561981	1.953765	-0.032741
H	-4.480925	1.868052	-0.613348
H	-3.173571	2.969251	-0.118256
H	-3.767997	1.728583	1.015887

$[CH_3Mo_2O_7]$ I 3/4

E = -19130.52

O	2.448960	-0.843308	1.451360
O	2.731466	-0.842692	-1.297657
Mo	1.886093	-0.200853	0.002865
O	0.078973	-0.394318	-0.188686
O	-1.392575	1.673531	-0.730904
O	-2.443352	-0.253858	1.591044
Mo	-1.881350	-0.332566	0.004150
O	2.212227	1.679601	0.076281
O	-2.748112	-1.335765	-1.030891
C	-0.880836	2.683605	0.022628
H	-1.020628	3.643308	-0.485098
H	0.228549	2.521359	0.029281
H	-1.222802	2.671794	1.061070

$[CH_2O]$ I 1/2

E = -3115.29

C	0.000000	-0.524279	0.000000
O	-0.000000	0.670794	-0.000000
H	0.939280	-1.110338	0.000000
H	-0.939280	-1.110338	0.000000

$[CH_2O]$ I 3/4

E = -3112.38

C	-0.048886	0.593453	-0.000000
O	-0.048886	-0.704649	0.000000
H	0.342205	1.038239	0.924402
H	0.342205	1.038239	-0.924402

$[HMo_4O_{13}]^-$ I 1/2

E = -34076.89

Mo	2.393277	-0.050816	-0.059357
O	0.058685	2.740744	1.580854
O	2.741096	-0.058185	1.580889
O	3.874633	-0.082220	-0.844883
O	1.605460	-1.674830	-0.631400
Mo	0.050886	2.392434	-0.059256
Mo	-0.050761	-2.392481	-0.059254
O	-0.082282	-3.873659	-0.845129
O	-0.058127	-2.740722	1.580881
O	1.675113	1.604992	-0.631636
O	0.000085	0.000244	0.628561
O	0.082211	3.873624	-0.845118
Mo	-2.393444	0.050821	-0.059307
O	-1.605436	1.674780	-0.631145
O	-3.874592	0.082281	-0.845224
O	-1.675048	-1.604998	-0.631288
O	-2.741676	0.058170	1.580839
H	0.000842	0.000029	1.591701

$[HMo_4O_{13}]^-$ I 3/4

E = -34073.75

Mo	-0.002605	-2.318594	-0.075196
O	2.861126	-0.132291	1.451632
O	-0.004159	-2.452246	1.827898
O	-0.004963	-4.003340	-0.133730
O	-1.616036	-1.586265	-0.804480
Mo	2.432053	-0.021036	-0.166093
Mo	-2.432244	-0.015535	-0.165998
O	-3.871190	0.050481	-1.025371
O	-2.860326	-0.126003	1.452044
O	1.612621	-1.590468	-0.804202
O	0.000416	-0.119417	0.694903
O	3.870570	0.041547	-1.026438
Mo	0.002854	2.381195	0.060824
O	1.612770	1.639961	-0.580163
O	0.004376	3.926897	-0.587109
O	-1.608614	1.643177	-0.580107
O	0.003121	2.580434	1.727008
H	-0.000126	-0.072988	1.656411

$[HMo_4O_{13}]^-$ II 1/2

E = -34076.67

Mo	-0.037860	2.163649	-0.212015
O	-3.593292	-0.402035	1.701816
O	-0.326319	3.757456	0.216122
O	-0.031254	2.141288	-1.886486
O	1.701732	1.905031	0.465151
Mo	-2.782047	-0.105836	0.258067
Mo	2.508463	0.173294	0.283666
O	3.698217	0.416553	-0.874020
O	3.225645	-0.082425	1.786286
O	-1.633823	1.336366	0.464427
O	0.582591	-0.194370	-0.182192
O	-3.926935	0.190585	-0.937503
Mo	0.123812	-2.088071	-0.285002
O	-1.728349	-1.549913	-0.207917
O	0.230925	-2.891115	-1.752135
O	2.247584	-1.864858	-0.068841
O	0.201063	-3.202360	0.977917
H	2.698297	-2.489088	0.500926

$[HMo_4O_{13}]^-$ II 3/4

E = -34074.19

Mo	-0.074656	2.187801	0.314069
O	3.357101	-0.207543	-1.905621
O	0.163290	3.775849	-0.163481
O	-0.137463	2.236540	1.985996
O	-1.789583	1.854133	-0.435842
Mo	2.723318	-0.047621	-0.354329
Mo	-2.437547	0.065531	-0.406674
O	-3.802153	0.099347	0.569521
O	-2.885875	-0.227994	-2.004687
O	1.556543	1.402778	-0.298238
O	-0.611932	-0.156469	0.370954
O	4.002092	0.173541	0.716825
Mo	-0.088313	-2.028045	0.501397
O	1.765646	-1.549349	0.109660
O	-0.106578	-2.703870	2.017021
O	-2.045215	-2.034808	-0.143618
O	0.181105	-3.266698	-0.999211
H	-2.333491	-2.625647	-0.841664

$[HMo_4O_{13}]^-$ III 1/2

E = -34076.53

Mo	-0.211296	2.005208	0.492970
Mo	-2.839264	-0.099590	-0.461850
Mo	0.457619	-2.028819	0.536200
Mo	2.839300	0.223229	-0.559841
O	2.181401	-1.454415	-0.102310
O	0.586031	-2.690748	2.083299
O	-1.833663	1.527054	-0.296447
O	-4.217142	0.447910	-1.243138
O	4.162495	0.620004	0.394721
O	1.433341	1.400509	-0.283258
O	-0.513962	-0.563909	0.512907
O	-2.239764	-1.434271	-1.624383
O	-3.356897	-0.709062	1.009228
O	-0.271645	-3.152140	-0.524924
O	3.328963	0.250139	-2.166112
O	-0.216122	1.850018	2.157378
O	-0.130652	3.652595	0.210173
H	-1.646126	-2.150669	-1.331176

$[HMo_4O_{13}]^-$ III 3/4

E = -34073.38

Mo	0.212367	-2.174901	0.387160
Mo	2.826462	0.105693	-0.343425
Mo	-0.480877	2.347258	0.384630

Mo	-2.748099	-0.303630	-0.461531
O	-1.957528	1.349236	-0.480930
O	-0.875439	3.185190	1.796858
O	1.829454	-1.497606	-0.310330
O	4.409941	-0.322149	-0.696726
O	-3.759680	-0.455895	0.871427
O	-1.412712	-1.630366	-0.410101
O	0.088862	0.423133	0.618398
O	2.270096	1.263130	-1.675225
O	2.823177	0.861614	1.156359
O	0.588895	3.129419	-0.713852
O	-3.677318	-0.496787	-1.850379
O	0.171316	-2.129203	2.057326
O	0.288483	-3.799368	-0.006033
H	1.685797	2.031626	-1.461383

$[HMo_4O_{13}]^-$ IV 1/2
E = -34076.46

Mo	0.343039	-1.955347	-0.857073
O	1.378818	0.414192	2.013420
O	0.356320	-3.614903	-0.639029
O	0.219875	-1.741466	-2.511600
O	-1.252460	-1.453243	0.076563
Mo	2.462385	0.187362	0.750920
Mo	-2.478118	-0.304181	0.868230
O	-4.026209	-0.631726	0.307530
O	-2.496386	-0.473271	2.536524
O	2.005788	-1.434296	-0.171262
O	0.546971	0.573656	-0.765416
O	3.922820	-0.259630	1.440203
Mo	-0.536726	1.968471	-0.778215
O	2.926242	1.837282	-0.016925
O	-1.127726	2.273792	-2.329215
O	-1.972281	1.424074	0.378133
O	0.339334	3.317389	-0.218049
H	2.226850	2.500339	-0.129207

$[HMo_4O_{13}]^-$ IV 3/4
E = -34073.16

Mo	-1.175779	1.503740	-1.151773
O	-0.318298	-0.318389	1.727182
O	-1.940526	2.989866	-1.002636
O	-1.046461	1.271003	-2.805697
O	0.442283	1.750551	-0.256196
Mo	-1.738434	-0.938168	0.990673
Mo	1.721211	1.167608	1.056552
O	2.991107	2.224517	0.844379
O	1.260176	1.099906	2.901301
O	-2.390925	0.363680	-0.189129
O	-0.317319	-0.849972	-1.035864
O	-2.929893	-0.978524	2.161852
Mo	1.296437	-1.574128	-0.968799
O	-1.591825	-2.752264	0.588557
O	2.048751	-1.634424	-2.481502
O	2.251166	-0.464598	0.236778
O	1.092515	-3.151544	-0.330486
H	-0.750294	-3.078633	0.212296

$[HMo_4O_{13}]^-$ V 1/2
E = -34076.40

Mo	2.219446	-0.093526	0.602196
O	0.041104	3.734637	-0.133527
O	2.352143	-0.266969	2.250045
O	3.971213	0.134333	-0.050772
O	1.918827	-1.761818	-0.078493
Mo	0.089415	2.096128	-0.494766
Mo	-0.012210	-2.024976	-0.571419
O	0.165680	-2.410483	-2.192860
O	-0.217557	-3.438189	0.308977
O	1.823198	1.723856	0.268388
O	0.281217	-0.075637	0.115177
O	0.263628	1.974818	-2.158189
Mo	-2.510470	0.041545	0.472852
O	-1.726339	1.645482	-0.078398
O	-4.169536	0.144507	0.220053
O	-1.845597	-1.368737	-0.524417
O	-2.254882	-0.208766	2.117461
H	4.155636	0.578576	-0.879805

$[HMo_4O_{13}]^-$ V 3/4
E = -34074.28

Mo	0.251533	-2.223223	-0.550280
O	-4.058940	-0.114218	-0.432910
O	0.213426	-3.161792	-1.919050
O	0.120065	-3.263202	1.050590
O	2.169017	-1.745238	-0.147710
Mo	-2.585731	-0.194300	0.376470
Mo	2.360012	0.137960	0.458810
O	2.445400	-0.136870	2.112700
O	3.892655	0.504248	-0.115310
O	-1.617082	-1.607398	-0.224310
O	0.767028	-0.331837	-0.765000
O	-2.895962	-0.381298	2.022450
Mo	-0.110860	2.445960	-0.367320
O	-1.586658	1.380712	0.111690
O	-0.403537	4.008102	0.170310
O	1.453206	1.864238	0.425970
O	0.043091	2.523229	-2.036170
H	-0.761795	-3.298105	1.431340

$[HMo_4O_{13}]^-$ VI 1/2
E = -34076.28

Mo	2.581126	-1.771554	-0.170354
O	1.027775	-2.482724	0.194118
O	3.779096	-2.388493	0.857971
O	3.011066	-2.065502	-1.783256
Mo	1.864831	1.888661	0.254832
Mo	-1.851335	1.875519	-0.270977
O	-2.270018	2.581990	-1.731690
O	-2.630652	2.728297	0.942009
O	-2.386644	0.101954	-0.257004
O	-0.007577	1.962604	-0.024378
O	2.641136	2.864892	-0.867743
O	2.232079	2.477418	1.782466
O	2.408767	0.164519	0.089627
Mo	-2.581622	-1.745699	0.181885
O	-2.447509	-1.914888	1.846563
O	-4.093126	-2.300382	-0.299927
O	-1.293245	-2.688314	-0.681940
H	-0.315204	-2.701882	-0.340732

$[HMo_4O_{13}]^-$ VI 3/4
E = -34073.12

Mo	2.664635	-1.572677	-0.402361
O	1.182327	-2.416199	-0.074675
O	3.870281	-1.593548	1.147315
O	3.606993	-2.019233	-1.735408
Mo	1.665204	1.880933	0.444320
Mo	-1.934232	1.764449	-0.493596
O	-2.350972	2.261917	-2.040209
O	-2.834488	2.671456	0.591481
O	-2.323122	-0.047250	-0.290558
O	-0.128283	2.037234	-0.213437
O	2.567079	3.232988	0.006891
O	1.617670	1.768889	2.121727
O	2.357657	0.383672	-0.288569
Mo	-2.434150	-1.835902	0.330202
O	-2.076469	-1.861247	1.968464
O	-3.988605	-2.432046	0.109051
O	-1.263320	-2.876642	-0.614190
H	-0.275222	-2.825629	-0.402765

$[HMo_4O_{13}]^-$ VII 1/2
E = -34076.22

Mo	0.165102	1.864782	-0.178049
O	-3.910022	0.000185	1.626477
O	0.119220	3.110054	0.946402
O	-0.275774	2.428186	-1.699070
O	2.059685	1.594836	-0.441684
Mo	-2.978644	-0.000224	0.227522
Mo	2.859961	0.000626	0.145137
O	4.423544	0.000684	-0.467729
O	2.994983	0.000867	1.821487
O	-1.945741	1.405653	0.187594
O	0.218652	-0.000324	0.358568
O	-4.126901	-0.000134	-1.267019
Mo	0.166829	-1.865303	-0.178113
O	-1.946401	-1.406080	0.188629
O	-0.275873	-2.428377	-1.698733
O	2.060256	-1.594643	-0.441624
O	0.119749	-3.110433	0.946461
H	-5.079441	0.001217	-1.170948

$[HMo_4O_{13}]^-$ VII 3/4
E = -34074.16

Mo	-0.806949	1.973852	-0.426327
O	2.778415	-0.240014	2.139144
O	-1.254770	3.135151	-1.557307
O	0.318852	2.646530	0.659712
O	-2.305621	1.274805	0.483315
Mo	3.186805	0.012618	0.528241
Mo	-2.873497	-0.529297	0.693856
O	-2.909039	-0.942624	2.323310
O	-4.412489	-0.756951	0.053054
O	4.800065	-0.403847	0.286684
O	-0.092424	0.527628	-1.219934
O	2.975567	1.825119	0.115571
Mo	0.232840	-1.456952	-0.942999
O	2.086932	-0.995743	-0.558903
O	0.532305	-2.581261	0.598904
O	-1.633474	-1.488544	-0.229803
O	0.212222	-2.287296	-2.375317
H	2.181294	2.287085	0.436225

$[HMo_4O_{13}]^-$ VIII 1/2
E = -34075.72

Mo	3.309397	-1.415227	0.285991
O	3.650486	-1.510800	-1.351194
O	1.878204	-2.501294	0.726612
O	4.655861	-1.917127	1.145598
Mo	1.891195	1.917234	0.285705
Mo	-0.878447	-0.001375	-1.082086
O	-0.047676	-1.476396	-0.879763
O	-1.081429	0.270948	-2.721707
O	-2.449374	-0.029320	-0.254013
O	0.234568	1.359239	-0.347180
O	2.691053	2.815846	-0.877245
O	1.718120	2.879881	1.642154
O	2.874623	0.377749	0.706386
Mo	-4.302583	-0.290195	0.515142
O	-4.539581	0.910346	1.706654
O	-5.444550	-0.129560	-0.743594
O	-4.378353	-1.851399	1.202597
H	1.082802	-2.423208	0.157961

$[HMo_4O_{13}]^-$ VIII 3/4
E = -34073.43

Mo	-3.027686	0.298415	-0.789873
O	-3.987703	-0.754431	0.099163
O	-2.016453	-0.626316	-2.013450
O	-4.021810	1.380817	-1.599994
Mo	-0.386878	0.949334	1.524400
Mo	0.679907	-2.158360	0.310341
O	-0.473257	-2.461663	-0.939263
O	1.383018	-3.497387	1.058350
O	1.971228	-0.797806	-0.209717
O	-0.096540	-0.824900	1.614183
O	-0.665368	1.579551	3.054204
O	0.981080	1.704309	0.835928
O	-1.879353	1.203474	0.407163
Mo	2.678467	0.790107	-0.833857
O	3.489238	1.985098	0.396519
O	4.117536	0.387451	-1.629196
O	1.677889	1.531575	-1.965154
H	-1.476047	-1.417042	-1.732376

$[HMo_4O_{13}]^-$ IX 1/2
E = -34075.72

Mo	-0.899618	-0.039177	0.923653
Mo	-4.385072	-0.294129	-0.472736
Mo	1.923425	1.937383	-0.209446
Mo	3.381104	-1.410765	-0.285988
O	3.064488	0.453055	-0.258733
O	1.944633	2.752949	-1.669286
O	-2.422724	-0.184089	0.021416

O	-5.110641	-1.499366	0.495011
O	2.579594	-2.175798	1.196374
O	5.023057	-1.735259	-0.237350
O	0.214814	1.308959	0.166557
O	-5.090696	1.231569	-0.170895
O	-4.498380	-0.694477	-2.128222
O	2.436802	3.001473	0.975881
O	2.752178	-2.043221	-1.702744
O	-0.008450	-1.492535	0.865584
O	-1.188572	0.336336	2.529640
H	1.597926	-2.195913	1.203829

$[HMo_4O_{13}]^-$ IX 3/4

E = -34073.48

Mo	0.858316	1.763327	-1.275840
Mo	2.122110	-0.937136	0.727502
Mo	-1.520258	1.106125	1.353479
Mo	-1.470991	-1.709153	-0.783919
O	-1.964577	-0.621531	0.628485
O	-1.348291	1.139907	3.020837
O	2.037619	0.350804	-0.572262
O	2.505558	-2.709420	0.165516
O	-1.900279	-0.873350	-2.363895
O	-2.263537	-3.184296	-0.717867
O	-0.071673	1.774037	0.481772
O	3.546002	-0.679899	1.597090
O	0.823595	-0.811695	1.809004
O	-2.840229	2.080637	0.989311
O	0.214471	-1.940490	-0.711586
O	-0.224165	1.139068	-2.474482
O	1.704995	3.177322	-1.640266
H	-1.301364	-0.101621	-2.584595

$[HMo_4O_{13}]^-$ X 1/2

E = -34075.57

Mo	-0.600486	-0.629116	-0.815190
Mo	-3.859443	0.142422	0.385989
Mo	1.895992	1.959328	-0.012117
Mo	2.837690	-1.301114	0.421806
O	3.098258	0.580444	0.440727
O	2.448247	2.826407	-1.339842
O	-2.153870	0.268000	-0.612773
O	-5.207768	0.384303	-0.620011
O	3.960149	-2.069145	-0.566601
O	1.151392	-1.434271	-0.238785
O	0.350123	1.042669	-0.397245
O	-3.718684	-1.511471	0.905341
O	-3.897729	1.211652	1.706464
O	1.689973	3.026661	1.265886
O	2.962192	-1.941201	1.971058
O	-0.508220	-0.908671	-2.444764
O	-1.332162	-2.115435	-0.019154
H	-2.232828	-2.083428	0.417098

$[HMo_4O_{13}]^-$ X 3/4

E = -34073.93

Mo	0.315671	-1.363882	0.522331
Mo	3.595581	0.113399	-0.282709
Mo	-1.068810	2.004218	0.180901
Mo	-3.160699	-0.641402	-0.362189
O	-2.743810	1.201238	-0.178159
O	-1.114130	2.809758	1.657691
O	2.216231	-1.029112	-0.027559
O	3.738460	1.234869	0.970961
O	-4.504619	-1.062512	0.563101
O	-1.661289	-1.464542	0.228441
O	0.098461	0.602528	0.215481
O	5.232751	-0.848181	-0.332719
O	3.487071	0.898839	-1.775089
O	-0.666760	3.108358	-1.022839
O	-3.473249	-1.005552	-1.977899
O	0.457521	-1.769832	2.117371
O	0.437311	-2.883132	-0.629709
H	1.335231	-3.059782	-0.922699

$[HMo_4O_{13}]^-$ XI 1/2

E = -34075.48

Mo	-4.136154	0.088309	-0.149872
O	-4.360973	-0.080849	-1.830244
O	-4.833978	1.541755	0.417641
O	-4.742144	-1.263979	0.690625
Mo	-0.465553	-0.945209	0.273987
Mo	3.162963	-1.123153	-0.184414
O	3.882852	-1.711569	-1.581298
O	3.962707	-1.781157	1.135770
O	3.208090	0.761045	-0.127720
O	1.363539	-1.491794	-0.144959
O	-1.264009	-1.917514	-0.822799
O	-0.676483	-1.602575	1.788457
O	-2.125927	0.323601	0.274705
Mo	1.787417	2.008023	0.035694
O	0.318704	0.945632	0.244030
O	2.005074	3.015757	1.361744
O	1.665585	2.962448	-1.340811
H	-1.868550	1.218839	0.512282

$[HMo_4O_{13}]^-$ XI 3/4

E = -34073.14

Mo	4.150066	0.051253	0.276840
O	4.471157	-0.315728	1.908812
O	4.972939	1.471989	-0.194713
O	4.516948	-1.263242	-0.743885
Mo	0.491568	-0.538399	-0.657174
Mo	-2.911803	-1.388219	0.276511
O	-3.047821	-2.050583	1.812690
O	-4.002901	-2.165168	-0.735562
O	-3.230075	0.478332	0.329014
O	-1.200047	-1.496005	-0.369206
O	1.118088	-1.893139	0.576816
O	0.833308	-0.780575	-2.252308
O	2.139615	0.492821	0.035909
Mo	-2.070101	1.944356	0.050808
O	-0.428287	1.183058	-0.251199
O	-2.570161	2.856001	-1.267778
O	-2.024072	2.944800	1.399103
H	1.901809	1.401884	0.245113

$[HMo_4O_{13}]^-$ XII 1/2

E = -34075.16

Mo	3.994251	-1.048712	-0.116624
O	2.159675	-1.051991	-0.046070
O	4.751908	-1.880701	1.143489
O	4.643384	-1.525152	-1.601794
Mo	1.912268	1.001330	0.243292
Mo	-1.912267	1.001128	-0.243405
O	-1.718032	1.614067	-1.785690
O	-1.659161	2.224939	0.870450
O	-3.863055	0.798089	-0.124695
O	0.000046	0.231559	-0.000540
O	1.658385	2.225726	-0.869716
O	1.717931	1.613373	1.785888
O	3.863160	0.798175	0.124682
Mo	-3.994174	-1.048758	0.116555
O	-4.641742	-1.524234	1.602694
O	-4.753353	-1.881234	-1.142332
O	-2.159556	-1.052190	0.044607
H	-0.000032	-0.732910	-0.000198

$[HMo_4O_{13}]^-$ XII 3/4

E = -34072.69

Mo	4.118526	-0.906076	-0.153591
O	2.298824	-1.091987	-0.011267
O	5.009463	-1.712742	1.033296
O	4.740018	-1.254461	-1.685120
Mo	1.874005	0.919853	0.387945
Mo	-1.830938	0.688764	-0.527181
O	-1.703371	1.104275	-2.123508
O	-1.531840	2.151914	0.725114
O	-3.702685	0.979092	0.075237
O	0.043107	0.016683	0.005156
O	1.457210	2.228550	-0.573710
O	1.665936	1.332199	1.991557
O	3.820899	0.911089	0.177102
Mo	-4.137384	-0.826178	0.234638
O	-4.609317	-1.306152	1.783719
O	-5.259897	-1.369438	-0.905131
O	-2.372453	-1.222050	-0.187459
H	0.136102	-0.943023	0.004078

$[HMo_4O_{13}]^-$ XIII 1/2

E = -34075.48

Mo	-0.465557	-0.945351	-0.274223
Mo	-4.136009	0.088370	0.150013
Mo	1.787366	2.008025	-0.035741
Mo	3.162839	-1.123105	0.184609
O	3.208061	0.761065	0.128066
O	2.005228	3.015644	-1.361842
O	-2.125775	0.323529	-0.274842
O	-4.742502	-1.263423	-0.690905
O	3.962616	-1.781025	-1.135609
O	1.363410	-1.491930	0.145055
O	0.318787	0.945552	-0.244235
O	-4.360602	-0.081367	1.830360
O	-4.833519	1.542215	-0.416849
O	1.665291	2.962555	1.340674
O	3.882743	-1.711561	1.581463
O	-0.676217	-1.602373	-1.788870
O	-1.264331	-1.917913	0.822120
H	-1.868369	1.218777	-0.512332

$[HMo_4O_{13}]^-$ XIII 3/4

E = -34073.14

Mo	-0.491568	-0.538285	-0.657225
Mo	-4.150091	0.051226	0.276834
Mo	2.070204	1.944309	0.050849
Mo	2.911742	-1.388299	0.276491
O	3.230125	0.478241	0.329018
O	2.570308	2.855979	-1.267702
O	-2.139664	0.492892	0.035788
O	-4.517320	-1.262463	-0.744802
O	4.002914	-2.165297	-0.735469
O	1.200038	-1.495962	-0.369347
O	0.428361	1.183078	-0.251199
O	-4.470851	-0.316986	1.908593
O	-4.972906	1.472431	-0.193458
O	2.024192	2.944710	1.399175
O	3.047562	-2.050684	1.812677
O	-0.833255	-0.780441	-2.252372
O	-1.118270	-1.892740	0.576976
H	-1.901950	1.401952	0.245109

$[HMoO_3]$ I 1/2

E = -8017.96

Mo	0.053131	-0.003588	0.000218
O	1.103621	-1.358560	-0.000397
O	0.788059	1.547935	-0.000389
O	-1.866186	-0.236913	-0.000335
H	-2.435453	0.530999	-0.000191

$[HMoO_3]$ I 3/4

E = -8017.59

Mo	0.082986	-0.000008	-0.006623
O	0.892491	-1.533738	0.020024
O	0.893337	1.533326	0.020003
O	-1.922978	0.000356	-0.097604
H	-2.388202	0.000807	0.738783

$[HMo_2O_7]$ I 1/2

E = -18065.11

Mo	1.883743	0.017323	0.027410
Mo	-1.854208	-0.054323	-0.017964
O	0.020551	0.132479	-0.106838
O	-2.666456	1.618731	-0.181078
O	-2.378940	-1.038071	-1.259346
O	2.350223	-1.480018	0.611580
O	2.712703	0.246344	-1.642995
O	-2.279865	-0.768952	1.428729
O	2.472274	1.219678	1.034496
H	-3.084407	2.112459	0.526879

$[HMo_2O_7]$ I 3/4

E = -18059.67

Mo	1.869633	-0.003020	0.015650

Mo	-1.882085	0.006398	-0.016879
O	0.001962	0.000006	-0.169915
O	-2.194297	1.593420	0.812353
O	-2.659576	0.068678	-1.480166
O	2.392301	-1.455747	0.661214
O	2.729173	0.214132	-1.640586
O	-2.180256	-1.693529	0.668540
O	2.365282	1.256886	0.999354
H	-3.113718	-0.012636	1.245279

$[HMo_2O_7]$ II 1/2
E = -18064.41

O	-2.622508	1.271592	-0.046005
O	-2.455019	-1.454785	0.151476
Mo	-1.580167	-0.033286	0.033146
O	-0.213000	-0.038662	-1.234754
O	2.645625	-0.638024	1.080990
O	1.874073	1.641425	-0.285849
Mo	1.325678	0.112548	0.010267
O	-0.118997	0.132401	1.245688
O	1.873197	-1.134588	-1.297861
H	2.821549	-1.563861	1.267199

$[HMo_2O_7]$ II 3/4
E = -18062.20

O	-2.612343	1.249508	-0.066784
O	-2.445661	-1.493659	0.034569
Mo	-1.571475	-0.062516	-0.006760
O	-0.162927	-0.030278	-1.268352
O	2.415322	-1.105653	0.940159
O	1.900524	1.752526	0.047278
Mo	1.270700	0.232181	0.017962
O	-0.207831	0.050122	1.284556
O	2.433045	-1.072410	-1.194049
H	2.071524	-1.927215	1.310478

$[CH_3Mo_2O_5]$ I 1/2
E = -15039.61

Mo	-1.392235	-0.142449	-0.086777
Mo	1.446283	0.035129	0.012022
O	-0.002489	0.032665	1.233198
O	0.098449	-0.045636	-1.314991
O	-2.290134	-1.498619	0.229831
O	2.445587	-1.307306	0.075033
O	2.363001	1.437749	0.036647
C	-2.507562	1.623718	0.113423
H	-2.987529	1.820977	-0.856081
H	-1.865352	2.469335	0.367442
H	-3.287078	1.523996	0.870050

$[CH_3Mo_2O_5]$ I 3/4
E = -15037.38

Mo	-1.361999	-0.269047	-0.159520
Mo	1.497584	0.058638	0.033388
O	-0.016209	1.080935	0.440955
O	0.274497	-1.185119	-0.681304
O	-2.649097	-1.174144	0.390896
O	2.356234	-0.474058	1.376033
O	2.536779	0.746220	-1.094669
C	-2.833295	1.845084	0.197198
H	-3.686403	1.443994	-0.338041
H	-2.193551	2.575009	-0.281398
H	-2.832484	1.796996	1.278529

$[CH_3Mo_2O_5]$ II 1/2
E = -15038.43

Mo	-1.764616	0.212023	-0.000044
Mo	1.015895	-0.347757	0.000025
O	-0.355137	0.014345	-1.294095
O	-0.354967	0.014931	1.293995
O	-3.436027	0.279510	0.000127
O	2.333571	0.959257	0.000069
O	1.428582	-1.959379	0.000243
C	3.715406	1.221616	-0.000188
H	3.965998	1.802464	0.888969
H	3.965339	1.803959	-0.888551
H	4.294339	0.295413	-0.001170

$[CH_3Mo_2O_5]$ II 3/4
E = -15038.34

Mo	-1.785360	0.167592	0.000100
Mo	1.037678	-0.348119	-0.000023
O	-0.367975	-0.066079	-1.286892
O	-0.367885	-0.066737	1.287034
O	-3.428239	0.483123	-0.000332
O	2.295291	1.014163	0.000107
O	1.533062	-1.935751	-0.000369
C	3.668709	1.320072	0.000054
H	3.899324	1.910981	0.887897
H	3.899904	1.907920	-0.889676
H	4.277101	0.413027	0.001808

$[CH_3Mo_2O_5]$ III 1/2
E = -15038.10

Mo	-1.186598	-0.001021	0.026762
Mo	1.647317	0.000377	-0.005627
O	0.237418	-0.000918	1.265413
O	0.223442	-0.000759	-1.246963
O	-2.998620	-0.000014	-0.117694
O	2.601692	-1.381296	-0.015947
O	2.599387	1.383637	-0.015685
C	-4.396906	0.002344	0.008508
H	-4.796232	-0.908920	-0.437942
H	-4.800584	0.869590	-0.514998
H	-4.678494	0.047112	1.061241

$[CH_3Mo_2O_5]$ III 3/4
E = -15038.30

Mo	1.201920	-0.490994	0.000006
Mo	-1.566584	0.154430	-0.000042
O	-0.258377	-0.265368	-1.283906
O	-0.258112	-0.263451	1.284042
O	2.975379	0.016269	-0.000337
O	-2.908192	-0.859171	0.001073
O	-2.049448	1.766126	-0.001035
C	3.844952	1.125685	0.000220
H	4.872129	0.759525	0.017274
H	3.670457	1.742249	0.884127
H	3.693577	1.724581	-0.899898

$[CH_3Mo_2O_5]$ IV 1/2
E = -15037.98

Mo	-1.606223	-0.150366	-0.000087
Mo	1.393812	-0.238836	0.000038
O	-0.258342	-1.406683	-0.000278
O	-0.102747	1.238908	-0.000106
O	-3.260648	-0.334038	0.000384
O	2.284083	-0.281365	-1.433138
O	2.283791	-0.281422	1.433393
C	0.130652	2.643830	-0.000006
H	-0.823644	3.170675	-0.000211
H	0.696172	2.914856	-0.891744
H	0.695741	2.914806	0.892022

$[CH_3Mo_2O_5]$ IV 3/4
E = -15037.75

Mo	-1.665772	-0.145524	-0.168566
Mo	1.346939	-0.233656	0.030214
O	-0.021281	-1.396920	-0.238701
O	0.050172	1.239964	-0.087321
O	-3.118425	-0.377317	0.620132
O	2.512909	-0.233616	-1.178501
O	2.083987	-0.362262	1.533826
C	0.142117	2.655732	0.057419
H	-0.822794	3.090926	-0.199517
H	0.907047	3.038686	-0.618167
H	0.395113	2.902750	1.088471

$[CH_3Mo_2O_5]$ V 1/2
E = -15037.28

Mo	-1.730367	0.088241	0.001161
Mo	1.139962	-0.300574	-0.000345
O	-0.137427	1.160114	0.000672
O	-0.438145	-1.354301	0.001198
O	-3.383023	0.323778	-0.004310
O	2.037994	-0.608395	1.387086
O	2.036229	-0.608430	-1.388937
C	2.628246	1.922038	0.000020
H	3.580956	1.410599	0.000557
H	2.260146	2.336681	0.928065
H	2.261407	2.336383	-0.928680

$[CH_3Mo_2O_5]$ V 3/4
E = -15037.26

Mo	-1.734299	0.094279	0.001010
Mo	1.135070	-0.305157	-0.000191
O	-0.128752	1.156967	0.002291
O	-0.444849	-1.353846	-0.000285
O	-3.386127	0.330845	-0.003651
O	2.034543	-0.611847	1.386408
O	2.031943	-0.608791	-1.389086
C	2.678089	1.916564	0.000025
H	3.623194	1.391792	0.000224
H	2.310407	2.329349	0.929073
H	2.311406	2.329747	-0.929264

$[Mo_3O_9]^-$ I 1/2
E = -24009.48

Mo	-2.179199	0.001154	0.000106
O	-0.717302	1.377152	0.004057
O	-0.718913	-1.376335	-0.001414
O	-3.082802	-0.000447	1.436014
O	-3.080231	0.003212	-1.437405
Mo	1.060068	1.718796	0.000169
O	1.513895	2.586696	-1.373804
O	1.519432	2.588214	1.371327
O	1.858637	-0.001008	-0.000605
Mo	1.058134	-1.719859	-0.000153
O	1.512993	-2.588169	1.373166
O	1.514523	-2.589791	-1.371974

$[Mo_3O_9]^-$ I 3/4
E = -24006.19

Mo	-2.178403	0.098668	-0.012319
O	-0.648447	1.381078	0.068099
O	-0.841236	-1.371085	0.302241
O	-3.266061	0.226304	1.284495
O	-2.871143	0.049375	-1.561498
Mo	1.150971	1.635231	0.037898
O	1.625516	2.521121	-1.319046
O	1.674296	2.441799	1.426260
O	1.862856	-0.108614	-0.017920
Mo	0.909997	-1.778790	0.077224
O	1.601462	-2.951894	1.061022
O	1.479288	-1.952409	-1.783368

$[Mo_3O_9]^-$ II 1/2
E = -24009.21

Mo	-0.000168	-0.751039	-0.000061
Mo	-2.649572	0.369656	0.000058
O	-1.399374	-0.085931	1.285494
O	-1.396494	-0.080840	-1.285052
O	-0.000795	-2.403348	0.000034
O	-3.088540	2.005632	0.003067
O	-4.035726	-0.605727	-0.003480
O	1.400323	-0.087165	-1.285715
O	1.396213	-0.080840	1.284777
Mo	2.649734	0.369463	-0.000014
O	4.036614	-0.604858	0.004620
O	3.087813	2.005658	-0.003657

$[Mo_3O_9]^-$ II 3/4
E = -24006.03

Mo	0.035421	-0.815986	0.000256
Mo	-2.702498	0.372412	-0.000160
O	-1.381999	-0.151260	1.220414
O	-1.378939	-0.147457	-1.219874
O	0.049958	-2.475047	0.001198
O	-3.026677	2.281267	0.003672
O	-4.037811	-0.651688	-0.004304

O	1.418149	-0.091876	-1.291758
O	1.414751	-0.084892	1.291281
Mo	2.640664	0.402902	-0.000137
O	4.062922	-0.523323	0.003878
O	3.018312	2.057806	-0.004292

$[Mo_2O_6]$ I 1/2
E = -16002.82

Mo	-1.429658	-0.000072	-0.000012
Mo	1.429639	-0.000174	0.000013
O	0.000010	-0.000603	-1.260991
O	-0.000079	-0.000769	1.260992
O	-2.387399	-1.366901	-0.000030
O	-2.387346	1.366814	0.000043
O	2.385231	1.368138	0.000031
O	2.389682	-1.365387	-0.000049

$[Mo_2O_6]$ I 3/4
E = -16000.11

Mo	-1.413871	-0.019281	-0.000036
Mo	1.428347	0.038670	0.000067
O	-0.008760	-0.028436	-1.278451
O	-0.008937	-0.028603	1.278478
O	-2.376705	-1.388262	-0.000040
O	-2.371160	1.352802	0.000060
O	2.178807	1.512412	-0.000085
O	2.510759	-1.521706	-0.000124

$[Mo_2O_6]$ I 5/6
E = -15997.25

Mo	-1.476259	-0.058000	0.000002
Mo	1.476094	0.057827	-0.000003
O	-0.000103	-0.000391	-1.213424
O	-0.000096	-0.000405	1.213424
O	-2.209598	-1.548383	-0.000005
O	-2.548301	1.518547	0.000000
O	2.208467	1.548654	0.000007
O	2.550493	-1.517113	0.000001

$[Mo_2O_6]$ I 7/8
E = -15993.80

Mo	-1.458904	-0.016113	0.011076
Mo	1.488062	0.039559	-0.002480
O	-0.009715	0.020242	-1.205655
O	0.033466	-0.048617	1.213571
O	-2.473434	-1.666610	-0.012039
O	-2.510624	1.604261	-0.026644
O	2.257286	1.512552	0.027916
O	2.549940	-1.544920	-0.042275

$[HMo_2O_7]^-$ I 1/2
E = -18070.63

O	-2.542783	1.354936	0.641266
O	-2.631537	-0.703190	-1.255734
Mo	-1.696759	0.038986	-0.036575
O	0.049669	0.601108	-0.768004
O	1.487709	-1.712421	0.512431
O	2.070741	1.066614	1.242893
Mo	1.660317	0.069891	-0.054544
O	-1.220176	-1.101947	1.177958
O	2.896842	0.154223	-1.196077
H	0.646833	-1.847399	0.989145

$[HMo_2O_7]^-$ I 3/4
E = -18067.90

O	-2.542668	1.333490	0.191199
O	-2.506221	-1.435896	-0.111803
Mo	-1.550497	-0.035197	0.012884
O	-0.288181	0.126016	-1.308373
O	2.449037	-0.898810	1.152539
O	1.948398	1.693256	-0.052764
Mo	1.320670	0.148973	-0.026814
O	-0.212170	-0.161437	1.284991
O	2.114824	-1.082255	-1.310232
H	1.948564	-1.373490	1.820603

$[HMo_2O_7]^-$ II 1/2
E = -18069.71

Mo	-1.935109	0.003030	-0.002025
Mo	1.935096	0.003026	0.002022
O	-0.000055	-0.739338	0.000023
O	2.023279	1.353883	1.034873
O	2.864942	-1.305957	0.590705
O	-2.864973	-1.305122	-0.592483
O	-2.404674	0.410235	1.583670
O	2.404229	0.411977	-1.583362
O	-2.022705	1.355066	-1.033389
H	0.000200	-1.700306	-0.000160

$[HMoO_4]$ I 1/2
E = -10062.80

Mo	-0.025872	-0.031946	-0.000700
O	-1.179583	1.455852	0.022887
O	1.752767	0.487395	-0.242059
O	-0.230648	-0.841227	1.452311
O	-0.511761	-0.997479	-1.282628
H	2.440427	0.505393	0.425332

$[HMoO_4]$ I 3/4
E = -10059.39

Mo	-0.220671	0.000260	-0.001970
O	-1.014472	1.484624	-0.085200
O	1.558578	-0.218119	-0.952970
O	1.530636	0.048055	1.151503
O	-1.183555	-1.380743	0.043857
H	2.138689	0.518550	-1.174778

$[HMoO_4]$ II 1/2
E = -10061.31

Mo	0.140216	0.077101	-0.000068
O	1.565594	-1.136671	0.000058
O	0.444959	1.708654	0.000111
O	-1.530913	-0.422600	-0.711110
O	-1.530613	-0.422624	0.711303
H	2.518700	-1.052324	-0.000027

$[HMoO_4]$ II 3/4
E = -10059.68

Mo	-0.216240	0.113164	-0.058773
O	-0.952567	-1.641276	0.026899
O	-1.261279	1.367008	0.247363
O	1.793089	0.312436	-0.501268
O	1.763766	-0.385854	0.601273
H	-1.661973	-1.971400	-0.525669

$[HMoO_4]$ III 1/2
E = -10060.21

Mo	-0.372142	0.005024	-0.118998
O	-1.573096	1.098120	0.303126
O	-0.786143	-1.590864	0.193785
O	1.413621	0.651052	-0.032501
O	2.504350	-0.228594	0.097614
H	3.160123	0.351269	0.501712

$[HMoO_4]$ III 3/4
E = -10057.83

Mo	0.300144	-0.057289	0.029803
O	1.723435	-0.865443	-0.241570
O	0.350397	1.889895	0.129644
O	-1.425748	-0.858865	0.484195
O	-1.915285	0.051980	-0.525851
H	-2.468441	0.665604	-0.023095

$[HMoO_4]$ IV 1/2
E = -10059.55

Mo	0.363606	-0.006121	0.166743
O	0.830951	1.492263	-0.388342
O	1.269883	-1.190670	-0.575675
O	-1.512084	-0.368415	0.167256
O	-2.609385	0.103284	-0.302200
H	0.893633	-0.034625	1.788494

$[HMoO_4]$ IV 3/4
E = -10058.16

Mo	0.430598	-0.000004	0.092952
O	1.227986	1.419409	-0.324302
O	1.228129	-1.419325	-0.324344
O	-1.982133	-0.000056	0.413586
O	-2.777752	-0.000000	-0.478258
H	0.345039	-0.000034	1.802558

$[Mo_2O_7]^-$ I 1/2
E = -18052.01

O	-2.483888	1.381718	0.435895
O	1.910615	-1.545680	-0.569578
Mo	-1.563510	0.012608	0.022076
O	1.882281	1.363623	-1.109239
O	-2.582997	-1.317537	-0.267323
Mo	1.410980	-0.066280	0.052342
O	-0.368674	0.313631	-1.274062
O	2.579931	0.418623	1.184950
O	-0.136487	-0.332604	1.208661

$[Mo_2O_7]^-$ I 3/4
E = -18048.89

O	2.429924	-0.738316	1.450865
O	-2.569404	-0.654718	-1.475438
Mo	1.863234	-0.056592	0.004738
O	-2.581025	-0.659082	1.462556
O	2.651582	1.677647	-0.110506
Mo	-1.932789	-0.070616	-0.003383
O	2.446825	-0.906567	-1.343740
O	-2.091640	1.905623	0.019385
O	0.078901	0.043258	-0.010232

$[O_2]$ I 1/2
E = -4090.36

O	-0.000000	0.000000	0.597111
O	0.000000	0.000000	-0.597111

$[O_2]$ I 3/4
E = -4090.86

O	-0.000000	0.000000	0.597272
O	0.000000	0.000000	-0.597272

$[CH_3Mo_4O_{13}]^-$ I 1/2
E = -35145.37

Mo	-2.329625	0.699653	-0.302497
O	-2.980039	0.534548	-1.844595
O	-1.241161	2.272515	-0.406541
O	-3.553592	1.139798	0.765568
Mo	-0.391557	-1.943358	0.294456
Mo	2.828042	-0.552036	-0.266553
O	3.521541	-1.001737	-1.732305
O	4.045549	-0.480182	0.892244
O	1.973089	1.080798	-0.448357
O	1.529934	-1.765577	0.245042
O	-0.681541	-2.987448	-0.993002
O	-0.662928	-2.757398	1.738910
O	-2.416709	-1.299395	0.199490
Mo	0.534270	2.164733	0.232743
O	-0.478973	0.002591	0.134246
O	0.543389	2.136582	1.907409
O	1.109364	3.679137	-0.196251
C	-3.525918	-2.141883	-0.020067
H	-4.441552	-1.608258	0.242725
H	-3.428490	-3.027676	0.610453
H	-3.565337	-2.444266	-1.069861

$[CH_3Mo_4O_{13}]^-$ I 3/4
E = -35142.71

Mo	-3.469324	-0.253156	-0.454859
O	-3.209758	0.382116	-1.996168
O	-4.696155	0.592861	0.388645
O	-4.539443	-1.768144	-0.707687

Mo	-0.605449	0.006915	1.027931
Mo	2.359000	-1.782999	-0.190745
O	2.164752	-2.625284	-1.635945
O	3.316672	-2.697996	0.849264
O	3.139130	-0.081038	-0.516913
O	0.763233	-1.370475	0.561318
O	-1.987378	-1.139292	0.353514
O	-0.626678	0.068706	2.679520
O	-2.021235	1.254498	0.379996
Mo	2.390749	1.641399	-0.315716
O	0.707700	1.328127	0.303572
O	3.282291	2.557025	0.780796
O	2.349433	2.457570	-1.787872
C	-1.946852	2.617051	0.029139
H	-2.938382	3.057841	0.154439
H	-1.636305	2.721797	-1.012939
H	-1.233690	3.137986	0.669679

$[CH_3Mo_4O_{13}]^-$ II 1/2
E = -35145.20

Mo	1.631932	-1.764571	-0.127700
O	2.245596	2.046441	1.420555
O	2.045931	-2.234022	1.425976
O	2.494087	-2.791059	-1.137720
O	-0.143572	-2.287570	-0.617320
Mo	1.783172	1.612703	-0.129798
Mo	-1.826086	-1.663240	-0.118484
O	-2.913432	-2.620541	-0.962122
O	-2.148246	-1.920909	1.507140
O	2.389804	-0.107423	-0.638358
O	0.240962	-0.006613	0.801411
O	2.730220	2.553547	-1.147410
Mo	-1.669383	1.817003	-0.117103
O	0.061312	2.295108	-0.613890
O	-2.667259	2.865382	-0.962725
O	-2.231359	0.098459	-0.649280
O	-1.970598	2.103047	1.507650
C	0.255943	-0.003629	2.233301
H	-0.308196	-0.854670	2.616532
H	-0.197334	0.913021	2.612267
H	1.277646	-0.066978	2.609704

$[CH_3Mo_4O_{13}]^-$ II 3/4
E = -35142.83

Mo	0.025143	-2.436350	-0.170222
O	3.128994	-0.000169	1.458969
O	-0.020188	-3.000031	1.418335
O	-0.016284	-3.776848	-1.183356
O	-1.397684	-1.389969	-0.555977
Mo	2.650401	0.000060	-0.152920
Mo	-2.769322	0.000021	-0.161395
O	-4.055416	0.000334	-1.264043
O	-3.179166	-0.000077	1.495535
O	1.669811	-1.528113	-0.555531
O	0.220430	-0.000794	1.417961
O	4.044245	0.000071	-1.086725
Mo	0.025169	2.436381	-0.170185
O	1.670072	1.528569	-0.555057
O	-0.016519	3.777014	-1.183136
O	-1.397262	1.389569	-0.556070
O	-0.020458	2.999688	1.418506
C	-0.178318	0.000112	2.707883
H	-1.295092	0.000182	2.643550
H	0.101343	0.923750	3.225563
H	0.100569	-0.923224	3.226606

$[CH_3Mo_4O_{13}]^-$ III 1/2
E = -35145.14

Mo	1.930641	-0.369711	0.812822
O	0.408182	3.774309	-0.367146
O	1.986925	-0.659538	2.450400
O	3.692742	-0.189179	0.233265
O	1.597324	-1.964233	-0.028815
Mo	0.238222	2.106293	-0.448694
Mo	-0.323026	-2.028229	-0.622872
O	-0.096046	-2.346044	-2.253992
O	-0.723823	-3.452373	0.170138
O	1.731926	1.531864	0.568738
O	0.064841	-0.178828	0.236725
O	0.499307	1.681709	-2.054272
Mo	-2.536908	0.296609	0.404403
O	-1.594470	1.879663	-0.016623
O	-4.155223	0.619207	0.092297
O	-2.075473	-1.165125	-0.638993
O	-2.415926	-0.052773	2.044671
C	4.144370	0.388660	-0.963717
H	5.235640	0.373318	-0.971876
H	3.792376	1.423717	-1.026095
H	3.768439	-0.166682	-1.828589

$[CH_3Mo_4O_{13}]^-$ III 3/4
E = -35142.89

Mo	1.660233	-1.281482	0.744671
O	1.986872	3.516954	0.476375
O	2.407103	-1.699711	2.168432
O	2.723982	-1.731583	-0.763169
O	0.315221	-2.716148	0.272266
Mo	1.350657	2.155443	-0.281565
Mo	-1.358181	-1.952552	-0.473521
O	-1.032280	-2.167067	-2.106995
O	-2.476173	-3.101472	0.021719
O	2.048815	0.649846	0.449008
O	-0.253048	-0.787350	0.815883
O	1.747586	2.220077	-1.919658
Mo	-2.206437	1.341694	0.266175
O	-0.524816	2.100597	-0.105769
O	-3.383223	2.359667	-0.362148
O	-2.415046	-0.312332	-0.528679
O	-2.462276	1.260089	1.922652
C	3.668411	-0.933197	-1.432435
H	4.189811	-1.561387	-2.159911
H	4.407079	-0.514464	-0.741103
H	3.187485	-0.107823	-1.965639

$[CH_3Mo_4O_{13}]^-$ IV 1/2
E = -35145.11

Mo	-0.323853	-2.328552	-0.362836
O	-1.270499	-0.855335	-0.006980
O	-0.718791	-3.555319	0.725900
O	-0.630085	-2.878627	-1.924722
Mo	2.843153	-0.664149	0.292382
Mo	0.897909	2.407948	-0.202268
O	1.020721	3.183877	-1.692710
O	1.145182	3.545574	1.015160
O	-0.687020	1.611687	-0.024663
O	2.242735	1.042072	-0.094736
O	4.266282	-0.982150	-0.539140
O	3.164392	-0.805332	1.933844
O	1.527264	-1.881066	-0.201992
Mo	-2.558713	0.762972	0.098671
O	-2.919130	1.401584	1.609529
O	-3.180862	1.750368	-1.105385
O	-3.860791	-0.610006	0.018222
C	-4.073273	-1.601282	0.981748
H	-3.219813	-2.284735	1.048427
H	-4.958049	-2.179136	0.702800
H	-4.234538	-1.152231	1.969819

$[CH_3Mo_4O_{13}]^-$ IV 3/4
E = -35141.82

Mo	-0.522910	2.361024	-0.053990
O	0.770906	1.340964	0.367559
O	-0.555099	3.801099	1.181324
O	-0.233256	3.098357	-1.550305
Mo	-3.170148	-0.206830	0.124478
Mo	-0.085616	-2.342022	-0.227651
O	-0.091512	-3.280931	-1.626093
O	-0.174521	-3.368837	1.103289
O	1.523294	-1.357905	-0.155119
O	-1.497288	-1.208036	-0.244298
O	-4.332079	-0.353551	-1.101599
O	-3.732809	-0.439098	1.706757
O	-2.081477	1.485087	-0.056771
Mo	3.020357	-0.307608	0.247538
O	3.047598	0.235559	1.834302
O	4.332732	-1.339453	0.083372
O	3.373476	1.070264	-0.953703
C	3.238324	2.470512	-0.943251
H	4.153049	2.910478	-1.348108
H	3.075388	2.846249	0.069718
H	2.391212	2.760348	-1.567549

$[CH_3Mo_4O_{13}]^-$ V 1/2
E = -35145.00

Mo	-0.123726	1.817301	-0.387217
Mo	-3.043870	-0.012357	0.142054
Mo	0.581959	-1.818451	-0.600927
Mo	3.008676	0.186280	0.441965
O	2.396873	-1.508445	-0.145701
O	-0.015944	-3.165470	0.216332
O	-2.033610	1.450895	-0.383143
O	-2.683585	-0.485230	1.906133
O	2.962598	0.252373	2.125079
O	1.839823	1.364594	-0.295818
O	-0.199383	-0.266076	-0.045963
O	-4.660844	0.444050	0.089259
O	-2.844658	-1.337057	-0.880216
O	0.456765	-2.064738	-2.259503
O	4.585273	0.481389	-0.069075
O	-0.088958	2.953474	0.841012
O	-0.030491	2.614144	-1.853981
C	-1.772410	-1.381938	2.502753
H	-0.795390	-0.904923	2.605370
H	-1.658838	-2.287087	1.902712
H	-2.149775	-1.644067	3.493324

$[CH_3Mo_4O_{13}]^-$ V 3/4
E = -35142.72

Mo	-0.168754	1.476613	0.811387
Mo	-3.211832	0.284302	-0.509586
Mo	0.870438	-1.981909	0.216687
Mo	3.111383	0.515076	-0.513943
O	2.531157	-1.285282	-0.353939
O	1.057360	-2.881691	1.628607
O	-2.087564	1.412375	0.414410
O	-3.559758	-1.252858	0.489733
O	4.536609	0.771378	0.345041
O	1.732571	1.456766	0.196582
O	-0.174490	-0.523016	0.519674
O	-4.681295	1.057093	-0.797115
O	-2.521648	-0.162278	-1.978093
O	0.194356	-2.980579	-0.958917
O	3.377836	0.909775	-2.128150
O	-0.073608	1.916363	2.404666
O	-0.281517	2.955868	-0.433084
C	-2.905869	-2.500871	0.548736
H	-2.190360	-2.501985	1.374893
H	-2.373008	-2.718397	-0.380335
H	-3.653358	-3.277133	0.726808

$[CH_3Mo_4O_{13}]^-$ VI 1/2
E = -35144.92

Mo	0.160035	-1.642081	-0.173438
O	4.225325	-0.686849	1.449211
O	0.315731	-2.641728	1.157859
O	0.071256	-2.596421	-1.543581
O	-1.840206	-1.411934	-0.023795
Mo	3.530152	-0.143811	0.012587
Mo	-3.329863	-0.422939	0.252830
O	-4.555763	-0.830286	-0.828158
O	-3.915803	-0.603103	1.822046
O	2.034717	-1.129945	-0.366933
O	0.021532	0.417998	-0.006197
O	4.657408	-0.271121	-1.232738
Mo	-0.935662	1.920322	-0.316474
O	3.083111	1.651962	0.233960
O	-0.736544	2.473383	-1.895073
O	-2.718624	1.347811	-0.033971
O	-0.505891	3.150941	0.756024
C	2.606508	2.291955	1.402430
H	2.321403	3.311380	1.143241
H	3.389353	2.306375	2.165638
H	1.724404	1.782247	1.796074

$[CH_3Mo_4O_{13}]^-$ VI 3/4
E = -35142.72

Mo	-0.168718	-1.476027	0.813663
O	-2.518143	0.162765	-1.978266
O	-0.279375	-2.955801	-0.430367
O	-0.075226	-1.914992	2.407280
O	1.733354	-1.455956	0.201104
Mo	-3.210299	-0.284537	-0.510900
Mo	3.109813	-0.515391	-0.515532
O	4.538005	-0.770911	0.338749
O	3.370479	-0.912039	-2.130189
O	-2.087063	-1.412560	0.414473
O	-0.174878	0.523430	0.520496
O	-4.679047	-1.057783	-0.800839
Mo	0.870467	1.982036	0.217349
O	-3.560337	1.252107	0.488470
O	1.059565	2.880527	1.629788
O	2.530197	1.285216	-0.355895
O	0.193320	2.982021	-0.956526
C	-2.906727	2.500176	0.549409
H	-3.654486	3.276035	0.728094
H	-2.373403	2.718937	-0.379095
H	-2.191636	2.500406	1.375942

$[CH_3Mo_4O_{13}]^-$ VII 1/2

E = -35144.85

Mo	-0.276823	-1.861382	-0.121279
O	3.728892	0.086721	1.798063
O	-0.281258	-3.101209	1.010387
O	0.263602	-2.425209	-1.610597
O	-2.156311	-1.627770	-0.499698
Mo	2.791793	0.068879	0.400938
Mo	-3.014697	-0.045660	0.037174
O	-4.542076	-0.082191	-0.660901
O	-3.241551	-0.042702	1.703718
O	1.788268	-1.363662	0.373934
O	-0.405359	0.009903	0.393435
O	3.941305	0.076221	-1.063444
Mo	-0.351794	1.873629	-0.152827
O	1.741758	1.464379	0.332387
O	0.157120	2.431009	-1.655202
O	-2.224035	1.566275	-0.514567
O	-0.391420	3.130742	0.959028
C	5.295479	-0.246500	-1.249537
H	5.720863	0.436749	-1.987673
H	5.383276	-1.270246	-1.621859
H	5.855352	-0.157174	-0.313865

$[CH_3Mo_4O_{13}]^-$ VII 3/4

E = -35142.70

Mo	0.427813	-0.181324	1.533818
O	4.431683	-0.956604	0.059532
O	0.370177	-0.096438	3.186166
O	1.427480	-1.679810	0.824872
O	-1.136872	-1.324768	1.013098
Mo	3.321017	0.222259	-0.399836
Mo	-2.316078	-1.543388	-0.348450
O	-1.556698	-2.200656	-1.705450
O	-3.604964	-2.530255	0.098787
O	2.126746	0.613455	0.970384
O	-0.546205	1.313056	0.625685
O	2.400804	-0.333871	-1.914624
Mo	-1.983108	1.844364	-0.349188
O	4.172837	1.621348	-0.784880
O	-1.509920	2.608807	-1.772645
O	-2.886785	0.219737	-0.733745
O	-2.976283	2.888321	0.522329
C	1.973722	-1.612715	-2.343337
H	2.108175	-1.672326	-3.425255
H	0.918424	-1.748069	-2.101536
H	2.550022	-2.402156	-1.855677

$[CH_3Mo_4O_{13}]^-$ VIII 1/2

E = -35144.46

2.423620	0.553668	-0.660872
-0.417821	2.317207	2.176143
3.556712	0.499584	0.578055
3.236879	0.946438	-2.077481
1.643046	-1.095848	-0.895605
-0.480071	2.348394	0.500465
0.658442	-2.186683	0.412205
-0.180917	-3.754126	-0.215357
1.939765	-2.803170	1.264573
1.150941	1.903442	-0.318150
0.344757	-0.396766	1.217280
-0.789895	3.940091	0.075105
-2.579390	-0.403338	-0.572046
-1.882005	1.337235	-0.166488
-3.513086	-0.359694	-1.982883
-1.201557	-1.490058	-0.772254
-3.540511	-0.934654	0.721885
-0.266377	-1.282899	2.044592
-1.357529	-1.268484	2.061932
0.213067	-1.444274	3.009833
-0.976988	-3.573022	-0.727483

$[CH_3Mo_4O_{13}]^-$ IX 1/2

E = -35144.40

Mo	0.710893	-0.734329	1.429511
Mo	2.372178	0.479272	-0.843198
Mo	-1.251119	1.954570	0.228033
Mo	-2.066739	-1.154685	-0.742552
O	-2.216072	0.728794	-0.838505
O	-2.356052	2.873186	1.095560
O	2.308088	0.157551	1.078578
O	3.407267	-0.618073	-1.622251
O	-3.588848	-1.800492	-0.444789
O	-1.023788	-1.448168	0.741939
O	-0.254990	0.964244	1.441565
O	0.668642	0.042722	-1.039278
O	2.763523	2.067475	-1.275885
O	-0.337034	3.033577	-0.668967
O	-1.499301	-1.880503	-2.149217
O	0.732335	-1.032873	3.058461
O	1.353689	-2.348918	0.774556
C	1.151695	-3.128484	-0.372713
H	1.920930	-3.901954	-0.398318
H	1.205126	-2.521215	-1.277869
H	0.165157	-3.596918	-0.317033

$[CH_3Mo_4O_{13}]^-$ IX 3/4

E = -35143.66

Mo	0.592423	-0.156511	0.923488
Mo	3.618006	0.345689	-0.446760
Mo	-2.309200	1.847845	-0.110613
Mo	-2.540330	-1.564458	-0.347487
O	-3.198179	0.201884	-0.450108
O	-3.129534	2.746774	1.053625
O	2.039138	0.990173	0.299247
O	4.628568	-0.785350	0.686560
O	-3.432125	-2.471496	0.755550
O	-0.814065	-1.367666	0.204062
O	-0.679873	1.320561	0.477784
O	3.353810	-0.355147	-1.956270
O	4.616411	1.673641	-0.680579
O	-2.195094	2.760436	-1.521214
O	-2.614219	-2.322947	-1.848346
O	0.609614	-0.235081	2.574665
O	1.908203	-1.529689	0.327191
C	1.772534	-2.796067	-0.055823
H	0.872602	-3.332271	0.220707
H	4.209339	-1.636021	0.849017
H	2.383863	-3.111771	-0.894523

$[CH_3Mo_4O_{13}]^-$ X 1/2

E = -35144.27

-0.549181	2.482755	0.022289
-0.503929	3.926171	-0.831851
1.281344	1.795853	-0.521477
-0.198553	2.756748	1.643004
-2.462773	-0.863080	-0.153312
0.799105	-2.564809	0.075759
1.306752	-3.903830	-0.807775
0.849058	-2.949617	1.710961
1.902660	-1.075913	-0.282033
-0.877561	-2.063372	-0.428807
-3.501900	-0.979852	-1.463779
-3.165310	-1.595104	1.193304
-2.423473	2.210207	0.106184
2.632552	0.663634	-0.089891
-0.888655	0.481141	-0.620960
3.166277	0.907503	1.484476
3.933610	0.872723	-1.137388
-2.970745	1.088123	0.720462
-2.651611	1.019274	1.769841
-4.061719	1.153185	0.689119
-0.132305	0.060500	-1.036065

$[CH_3Mo_4O_{13}]^-$ XI 1/2

E = -35144.27

0.036909	-1.610495	0.709142
3.800232	-0.124392	0.991515
0.204009	-2.410310	2.153253
-0.220965	-3.469965	-0.518686
-1.756745	-1.346366	-0.011868
2.834034	-0.026741	-0.389100
-2.863703	0.153176	-0.272850
-3.287586	0.174608	-1.913795
-4.271323	0.043069	0.656786
1.612676	-1.457485	-0.425262
0.185702	0.204372	1.114188
3.834910	-0.134734	-1.746484
0.088829	1.981845	0.311960
1.916960	1.552977	-0.439193
-0.163723	2.725776	-1.445562
-1.890278	1.602358	0.190430
0.285282	3.154833	1.465773
-0.773883	-3.474347	-1.595331
-0.832167	-4.410463	-2.164736
-1.229291	-2.566719	-2.009699
0.676675	2.798341	-1.906721

$[CH_3Mo_4O_{13}]^-$ XII 1/2

E = -35144.16

2.013241	-0.243218	0.856773
-0.062129	4.052384	0.031231
2.947155	-0.559034	2.200501
3.152425	0.726200	-0.757052
1.991429	-1.648849	-0.396384
0.041019	2.389934	-0.229446
0.079379	-2.159097	-0.417708
0.167960	-1.768626	-2.321714
-0.029805	-3.773119	-0.019129
1.265239	1.652450	0.865635
0.213961	-0.831097	1.040714
0.588805	2.088364	-1.818322
-2.602620	-0.047262	0.284074
-1.610700	1.567377	0.032989
-4.114957	0.194090	-0.417111
-1.823272	-1.442402	-0.573911
-2.859164	-0.357863	1.917532
2.761319	0.625088	-1.909187
3.216813	1.263336	-2.672960
2.001944	-0.109941	-2.201670
-0.705012	-1.597894	-2.685193

$[CH_3Mo_4O_{13}]^-$ XIII 1/2

E = -35144.16

1.851025	0.531309	0.917384
-1.049583	3.486197	0.111568
2.205668	0.938564	2.480365
3.502832	0.374881	-0.071855
1.667940	-1.462260	0.766282
-0.642314	2.030975	-0.621855
0.550302	-2.202517	-0.459284
0.823365	-1.449942	-1.954215
0.898380	-3.845072	-0.596946
1.451977	2.221686	-0.168618
-0.063860	0.469631	0.444731
-0.310826	2.290988	-2.253602
-2.392703	-0.550079	0.407999
-2.269378	1.025273	-0.622891
-3.913846	-1.133132	0.011840
-1.267632	-2.023622	0.014851
-2.449994	-0.234058	2.053789
3.809008	0.055176	-1.272918
4.857948	0.086285	-1.549773
3.040208	-0.265521	-1.972053
2.062446	2.568164	-0.821341

$[CH_3Mo_4O_{13}]^-$ XIV 1/2

E = -35144.16

1.851937	-0.529994	0.917394
0.895058	3.845198	-0.596817
2.207403	-0.936881	2.480289
3.503083	-0.371607	-0.072511
1.454237	-2.221487	-0.167321
0.548330	2.202375	-0.459124
-0.640549	-2.030942	-0.621985
-0.307593	-2.289728	-2.253624
-1.046706	-3.486939	0.110517
1.666667	1.463419	0.766624
-0.063083	-0.469546	0.444532
0.822427	1.450025	-1.953992
-2.393729	0.548193	0.408009
-1.269803	2.022353	0.014225
-3.915546	1.129848	0.012326
-2.268901	-1.027163	-0.622613
-2.450101	0.232297	2.053853
3.808509	-0.050681	-1.273425
4.857397	-0.080629	-1.550597
3.039069	0.269690	-1.972007
2.063838	-2.567818	-0.821076

$[CH_3Mo_4O_{13}]^-$ XV 1/2

E = -35144.15

0.687466	-1.868426	0.770704
3.776412	1.814895	0.071132
0.585220	-2.822689	2.118886
1.855456	-2.908842	-0.462493
-0.678448	-1.988228	-0.673696
2.323326	1.042694	-0.305092
-2.208186	-0.946422	-0.544835
-3.055837	-1.326594	-1.947148
-3.202730	-1.397199	0.736470
2.118713	-0.452579	0.676278
-0.564965	-0.222201	1.114889
2.289930	0.631070	-1.951842
-1.035272	2.202073	0.266786
0.841422	2.150168	0.050110
-1.461607	3.675485	-0.408896
-2.023914	0.966623	-0.720263
-1.490520	2.304955	1.884504
2.283937	-2.694161	-1.601578
2.905499	-3.456314	-2.073620
2.054517	-1.766437	-2.128946
-0.804730	-0.067787	2.030971

$[CH_3Mo_4O_{13}]^-$ XVI 1/2

E = -35143.92

-0.043822	-1.422234	1.096206
4.215085	0.208024	-0.396348
0.033368	-2.221121	2.537863
-0.239107	-3.002686	-0.161140
-2.008902	-1.008179	0.660910
2.561654	0.004253	-0.643298
-2.493625	0.191234	-0.604969
-2.471099	-0.424314	-2.192154
-4.096460	0.587955	-0.279087
1.951223	-1.142203	0.592607
-0.074355	-0.130792	-0.816064
2.342298	-0.560642	-2.234638
0.088107	1.860076	0.479851
1.732683	1.758899	-0.390503
0.166781	3.308731	1.306437
-1.564690	1.917529	-0.363593
0.041579	0.586240	1.688517
-0.589869	-3.153143	-1.325725
-0.670808	-4.170290	-1.716806
-0.832809	-2.301434	-1.964679
0.098391	-0.228804	-1.753815

$[CH_3Mo_4O_{13}]^-$ XVII 1/2

E = -35143.85

1.829583	-0.042886	0.968109
-0.118746	3.811505	-0.566826
1.939525	-0.220017	2.621365
3.875573	0.176716	0.309143
1.919880	-1.635125	-0.040265
-0.050932	2.131464	-0.552616
0.048580	-1.850032	-0.756053
0.251054	-1.825709	-2.407073
-0.109277	-3.634662	-0.073993
1.572762	1.797378	0.365362
0.006433	-0.141074	0.317361
0.182072	1.640999	-2.147105
-2.481023	-0.077050	0.481131
-1.801542	1.641313	-0.022806
-4.126441	0.032683	0.148047
-1.912857	-1.588487	-0.428589
-2.350197	-0.301126	2.146055
4.193666	-0.085517	-0.835796
5.235625	0.058433	-1.141458
3.463903	-0.470681	-1.556282
-1.028176	-3.892664	0.043114

$[CH_3Mo_4O_{13}]^-$ XVIII 1/2

E = -35143.85

1.829594	-0.043596	0.968327
-0.117726	3.811423	-0.567447
1.939986	-0.221489	2.621465
3.875449	0.176462	0.308403
1.919581	-1.635517	-0.040362
-0.050173	2.131374	-0.552296
0.048181	-1.849936	-0.756168
0.250734	-1.826255	-2.407173
-0.110310	-3.634069	-0.073054
1.572868	1.797182	0.366463
0.006483	-0.141336	0.317673
0.183312	1.640008	-2.146473
-2.481268	-0.076391	0.480827
-1.800938	1.641857	-0.022344
-4.126584	0.034070	0.147464
-1.913207	-1.587700	-0.429074
-2.350835	-0.300993	2.145700
4.192662	-0.084776	-0.837008
5.234394	0.059319	-1.143389
3.462412	-0.469075	-1.557474
-1.029249	-3.891654	0.044038

$[CH_3Mo_4O_{13}]^-$ XIX 1/2

E = -35143.83

Mo	2.927214	0.388279	0.324293
O	1.910130	-0.993116	0.964210
O	2.782382	1.858840	1.131728
O	4.561279	-0.003942	0.174040
Mo	0.729148	-1.184084	-0.710032
Mo	-2.309775	-0.828975	0.183254
O	-2.765177	-0.762662	1.796630
O	-3.371216	-1.910012	-0.522654
O	-2.750666	0.885196	-0.467768
O	-0.624458	-1.968542	0.677788
O	1.365843	-2.573497	-1.367658
O	-0.798056	-0.945012	-1.512967
O	1.939872	0.166457	-1.281912
Mo	-1.247116	2.041667	-0.157306
O	-0.639129	2.734754	-1.574522
O	-1.575478	3.249013	0.980100
O	-0.239200	0.710302	0.492896
C	-0.308916	-2.313083	2.020774
H	0.553835	-2.978023	2.018405
H	-1.172015	-2.817451	2.453702
H	-0.075147	-1.417562	2.595227

$[CH_3Mo_4O_{13}]^-$ XIX 3/4

E = -35142.25

Mo	3.412824	0.586386	0.402732
O	2.673900	-1.090735	0.070775
O	3.716339	0.901478	2.034737
O	4.787053	0.930954	-0.519917
Mo	0.997591	-0.406568	-0.786864
Mo	-1.899347	-1.476692	0.370842
O	-1.853452	-2.180692	1.898927
O	-2.589467	-2.564108	-0.710435
O	-3.119127	-0.049986	0.506700
O	0.075195	-2.079757	-0.083690
O	1.067589	-0.525756	-2.433286
O	-0.768727	0.133746	-0.176094
O	1.839741	1.318408	-0.251413
Mo	-2.420032	1.666011	-0.027236
O	-2.540345	1.903014	-1.685148
O	-3.519093	2.729331	0.703266
O	-0.946876	2.573560	0.730816
C	0.579831	-3.389407	0.096974
H	1.666606	-3.368668	0.013376
H	0.153311	-4.047405	-0.663334
H	0.295759	-3.746931	1.088288

$[CH_3Mo_4O_{13}]^-$ XX 1/2

E = -35143.83

2.026067	0.089487	0.847974
-0.125173	3.675820	-0.048050
2.260796	0.015665	2.500525
4.042157	0.092356	0.084074
1.898185	-1.530854	-0.148365
-0.043908	1.840057	-0.594908
0.017140	-2.000274	-0.469477
0.160508	-2.468677	-2.075954
-0.097162	-3.383984	0.471991
1.833426	1.808777	0.084146
0.113649	0.027395	0.387971
0.079106	1.705850	-2.248393
-2.715975	0.012818	0.377294
-1.962156	1.607887	-0.156136
-4.333256	-0.011769	-0.099136
-1.825080	-1.401910	-0.459414
-2.668658	-0.141508	2.057227
4.264433	-0.162532	-1.085053
3.454637	-0.402370	-1.782114
5.300585	-0.163685	-1.440187
0.747852	4.013194	0.171649

$[CH_3Mo_4O_{13}]^-$ XXI 1/2

E = -35143.72

-1.171604	1.592417	-0.713663
-1.638841	1.885299	-2.280706
0.683817	2.269086	-0.563645
-1.784298	2.942110	0.490746
-1.574551	-1.446133	0.538322
1.511548	-1.847118	-0.448481
1.202144	-1.924438	-2.094031
2.672932	-3.016689	-0.149821
2.412725	-0.240954	-0.024731
0.021800	-2.482906	0.539995
-2.683395	-2.344250	-0.350272
-2.072898	-1.376083	2.142682
-2.673066	0.397593	0.074437
1.963416	1.516281	0.507939
-0.349932	-0.131462	-0.261903
1.412376	1.468338	2.099168
3.352691	2.464491	0.451420
-3.926446	0.715060	0.417231
-4.430091	1.456850	-0.192058
-4.471831	-0.011771	1.003509
-2.597802	2.734689	0.965562

$[CH_3Mo_4O_{13}]^-$ XXII 1/2

E = -35143.72

-1.573971	1.446450	-0.538570
-2.071543	1.376243	-2.143149
0.022756	2.482780	-0.539784
-2.682820	2.345381	0.349216
-1.171954	-1.591931	0.713896
1.962692	-1.516760	-0.508208
1.411181	-1.468266	-2.099261
3.351644	-2.465478	-0.452352
2.412920	0.240115	0.024768
0.683103	-2.269344	0.563608
-1.785773	-2.941553	-0.490010
-1.639147	-1.884343	2.281033
-2.673032	-0.396459	-0.074291
1.512108	1.846495	0.448741
-0.349669	0.131521	0.261909
1.202473	1.923589	2.094261
2.673933	3.015742	0.150496

-3.926521	-0.713686	-0.417034
-4.471404	0.012699	-1.004358
-4.430761	-1.454380	0.193117
-2.599665	-2.734267	-0.964210

$[CH_3Mo_4O_{13}]^-$ XXIII 1/2
E = -35143.72

-1.175037	1.589365	-0.714474
-1.641846	1.879032	-2.282220
0.677899	2.271520	-0.563159
-1.793678	2.938698	0.487697
-1.570272	-1.449410	0.539497
1.516377	-1.842614	-0.449810
1.206896	-1.919245	-2.095398
2.680391	-3.009882	-0.152262
2.414395	-0.234827	-0.024945
0.028374	-2.482540	0.538470
-2.678592	-2.351326	-0.346051
-2.065473	-1.379685	2.144759
-2.672991	0.390049	0.074183
1.958102	1.520184	0.509151
-0.348166	-0.131627	-0.261271
1.405044	1.468675	2.099564
3.344574	2.472603	0.455847
-3.928017	0.703990	0.414731
-4.432698	1.444090	-0.195775
-4.472477	-0.024504	0.999832
-2.606475	2.728850	0.962592

$[CH_3Mo_4O_{13}]^-$ XXIV 1/2
E = -35143.72

-1.571455	1.448668	-0.538764
-2.069155	1.378867	-2.143335
0.026948	2.482630	-0.540110
-2.678922	2.349362	0.349004
-1.174454	-1.590273	0.714136
1.960060	-1.519552	-0.508329
1.408573	-1.470458	-2.099370
3.347806	-2.469924	-0.452304
2.412989	0.236731	0.024241
0.679526	-2.270422	0.563614
-1.790208	-2.939079	-0.489885
-1.642463	-1.881814	2.281192
-2.673524	-0.392578	-0.074435
1.515056	1.844414	0.448874
-0.349482	0.131852	0.261577
1.204872	1.921385	2.094297
2.678914	3.011911	0.151554
-3.927589	-0.707719	-0.417007
-4.471650	0.019920	-1.003570
-4.432832	-1.447987	0.192878
-2.603702	-2.730072	-0.964056

$[CH_3Mo_4O_{13}]^-$ XXV 1/2
E = -35143.72

-2.523386	0.481122	-0.266887
-3.159909	0.362702	-1.818698
-1.701060	2.148180	-0.175949
-3.801436	0.561112	0.826685
-0.115891	-1.946809	0.238592
2.978503	-0.221661	-0.246014
3.643685	-0.535595	-1.763720
4.222663	-0.127520	0.888539
1.976055	1.288821	-0.273397
1.733719	-1.542898	0.237781
-0.250412	-3.056740	-1.013896
-0.343187	-2.756670	1.687174
-2.230280	-1.570570	0.042889
0.203744	1.963800	0.323303
-0.496701	0.026377	0.084839
0.343078	2.332980	1.931491
0.522062	3.519332	-0.740719
-3.184106	-2.513615	-0.017483
-4.209499	-2.180460	-0.079527
-2.864859	-3.526057	-0.217174
-0.291971	3.981167	-0.960375

$[CH_3Mo_4O_{13}]^-$ XXVI 1/2
E = -35143.71

-2.525520	0.530325	-0.266242
-3.144960	0.427500	-1.825929
-1.670894	2.172546	-0.145943
-3.814044	0.619624	0.814984
-0.165024	-1.943434	0.225968
2.967975	-0.271104	-0.231330
3.686759	-0.603168	-1.720580
4.174245	-0.178457	0.943343
2.000853	1.266056	-0.317232
1.696892	-1.569055	0.220836
-0.317189	-3.045417	-1.031330
-0.392094	-2.758271	1.671737
-2.265919	-1.529628	0.051966
0.228770	1.959250	0.316078
-0.499949	0.033998	0.072071
0.395213	2.316661	1.924348
0.629692	3.500553	-0.741507
-3.238866	-2.453993	0.007673
-4.258643	-2.100572	-0.025695
-2.943798	-3.473560	-0.192480
1.546352	3.522976	-1.029878

$[CH_3Mo_4O_{13}]^-$ XXVII 1/2
E = -35143.65

2.215695	0.844102	-0.018296
3.034587	0.895034	1.705823
0.913543	2.251048	0.539294
3.164637	1.522903	-1.203169
0.597194	-1.997407	-0.201529
-2.691331	-0.832748	0.140373
-3.576493	-1.400915	1.450941
-3.735795	-0.704604	-1.168399
-1.952463	0.817844	0.566103
-1.321309	-2.022621	-0.262278
0.925209	-2.664938	1.309853
1.097708	-3.049514	-1.413460
2.698804	-1.159960	-0.051557
-0.828981	2.258935	-0.026885
0.574033	-0.115187	-0.500916
-0.899155	2.382981	-1.700552
-1.528539	3.654015	0.593269
3.772317	-1.776385	0.444707
4.734006	-1.300351	0.286741
3.684104	-2.836955	0.639326
3.501609	0.085888	1.952254

$[CH_3Mo_4O_{13}]^-$ XXVIII 1/2
E = -35143.64

-2.426587	0.614209	-0.167814
-3.336841	0.460908	-1.573238
-1.491236	2.234026	-0.329512
-3.461584	0.810179	1.145497
-0.286059	-1.921232	-0.008346
2.879190	-0.409694	-0.089677
3.765709	-0.889883	-1.440994
3.943635	-0.159704	1.193022
1.913167	1.135655	-0.484930
1.631038	-1.675550	0.356285
-0.301274	-2.755816	-1.444732
-0.482351	-3.036047	1.529422
-2.331247	-1.515997	0.168915
0.392738	2.150754	0.099851
-0.498081	0.028466	-0.052975
0.506962	2.275472	1.763985
0.837564	3.643628	-0.518233
-3.351219	-2.360489	0.360125
-4.325424	-1.922680	0.529409
-3.228676	-3.371526	-0.011210
-1.392144	-3.215138	1.792323

$[CH_3Mo_4O_{13}]^-$ XXIX 1/2
E = -35143.54

1.438387	-1.116264	0.965781
2.206168	3.396030	-0.418329
1.789338	-1.739945	2.468675
3.079349	-1.170968	-0.484584
0.703912	-2.416505	-0.213368
1.346339	1.943267	-0.395470
-1.137796	-1.867660	-0.574068
-1.014277	-1.908427	-2.257464
-2.043085	-3.177428	-0.031877
1.997602	0.818727	0.884439
-0.344694	-0.506121	0.800942
1.577639	1.092777	-1.869640
-2.103358	1.260257	0.253103
-0.429713	2.243127	-0.088176
-3.198966	2.408689	-0.276132
-2.461372	-0.392266	-0.479663
-2.093279	1.236780	2.153679
2.811208	-1.146200	-1.675768
3.607853	-0.914099	-2.388971
1.814855	-1.396295	-2.048208
-1.268930	0.888685	2.511196

$[CH_3Mo_4O_{13}]^-$ XXX 1/2
E = -35143.42

1.760154	-0.149357	1.060810
-0.027855	3.950266	-0.375569
2.155865	-0.371874	2.669170
3.794294	0.273443	0.261411
1.884568	-1.600310	-0.162804
-0.068138	2.284526	-0.566433
0.038553	-1.934019	-0.768000
0.223282	-1.287827	-2.340479
0.064804	-3.617186	-0.780727
1.397962	1.630811	0.432523
-0.074216	-0.580994	0.769881
0.171747	1.542634	-2.275052
-2.416833	-0.137667	0.531692
-1.683032	1.570206	0.100555
-4.002445	0.051355	0.003907
-1.854732	-1.618634	-0.488639
-2.544441	-0.429955	2.181773
4.037918	0.102931	-0.913905
5.043624	0.327969	-1.289852
3.278258	-0.284235	-1.603748
0.227330	0.576918	-2.429461

$[CH_3Mo_4O_{13}]^-$ XXXI 1/2
E = -35143.32

1.534549	-0.128558	1.183095
0.326501	3.719839	-1.233240
1.792062	-0.455383	2.791132
3.525851	0.433789	0.452914
1.832695	-1.554077	-0.066909
0.146141	2.107095	-0.760373
0.103953	-2.085133	-0.730615
0.438493	-2.319443	-2.365978
-0.191975	-3.594006	-0.059671
1.162565	1.751591	0.757197
-0.257089	-0.551635	0.677834
0.799889	1.079146	-1.960756
-2.402244	0.176702	0.565954
-1.595505	1.725173	-0.451995
-3.975035	-0.121798	-0.005066
-1.720758	-1.309091	-1.016365
-2.392149	0.549567	2.217177
3.754650	0.471204	-0.740614
4.760419	0.752586	-1.074530
2.982444	0.240521	-1.484729
-2.295895	-1.594191	-1.725819

$[CH_3Mo_4O_{13}]^-$ XXXII 1/2
E = -35143.19

0.010679	2.371991	-0.216420
-2.992339	-0.000169	1.568297
0.157521	2.912199	1.369738
-0.007586	3.752515	-1.169597
1.530454	1.462268	-0.784452
-2.525037	0.000127	-0.042701
2.546380	0.000051	-0.067157
4.003604	-0.001364	-0.879670
2.679276	0.000920	1.772355
-1.680841	1.592734	-0.578289
-0.090841	0.001112	0.943322
-3.952408	-0.000205	-0.922764
0.010574	-2.372574	-0.216240

-1.680095	-1.592158	-0.578517
-0.007201	-3.753707	-1.168551
1.529158	-1.461428	-0.784246
0.157103	-2.911463	1.370401
-0.045717	0.000718	2.277401
-0.253030	-0.938821	2.784337
-0.247550	0.941202	2.784888
1.819393	0.000254	2.287884

$[CH_3Mo_4O_{13}]^-$ XXXIII 1/2

E = -35143.13

-1.840915	1.432541	-0.434488
-1.569936	0.907023	-2.041330
0.004032	2.335955	-0.216629
-2.817835	2.779519	-0.107953
-1.135903	-1.994769	0.831949
2.213872	-1.263777	-0.760812
2.266779	-1.634839	-2.400364
3.469547	-2.079693	0.008215
2.426698	0.601587	-0.506556
0.614689	-1.725092	-0.032696
-2.359107	-2.510494	-0.251165
-1.047516	-2.793731	2.315742
-3.672395	0.146257	-0.508857
1.545322	1.891202	0.555957
-1.235375	0.049200	1.072811
1.152872	1.153073	2.048055
2.516678	3.244942	0.809937
-3.779222	-0.763924	-1.307942
-3.065630	-0.874672	-2.130471
-4.627382	-1.451036	-1.231391
-0.484469	0.381325	1.606344

$[CH_3Mo_4O_{13}]^-$ XXXIV 1/2

E = -35143.06

-2.294375	0.484569	-0.404395
-2.840426	0.253048	-1.978301
-1.356304	2.159524	-0.490048
-3.611685	0.809919	0.588761
-0.139736	-1.930828	0.518552
2.693452	-0.342358	-0.343218
2.203267	-1.169022	-1.976778
4.321109	-0.564988	-0.035545
1.936118	1.355851	-0.210711
1.685558	-1.515643	0.764882
-0.080444	-2.986376	-0.806194
-0.577441	-2.771118	1.907288
-2.246621	-1.548001	0.059095
0.339648	2.291745	0.316472
-0.454640	-0.025536	0.230041
0.204685	2.278210	1.986675
0.774563	3.858686	-0.089268
-3.220641	-2.447802	-0.119206
-4.140019	-2.111836	-0.577220
-2.950376	-3.489406	-0.022009
1.594787	-1.919737	-1.955932

$[CH_3Mo_4O_{13}]^-$ XXXV 1/2

E = -35143.02

-2.408435	0.697427	-0.226966
-3.279905	0.745255	-1.664337
-1.310043	2.295937	-0.290346
-3.445197	1.029354	1.057098
-0.387829	-1.961705	0.181259
2.837224	-0.521118	-0.216578
3.782811	-1.017167	-1.520789
3.847716	-0.331660	1.117699
1.971412	1.032094	-0.656272
1.505910	-1.782949	0.144991
-0.698950	-3.088980	-1.028109
-0.709222	-2.658190	1.675879
-2.504494	-1.355401	0.037559
0.517289	2.148773	0.077171
-0.556166	-0.047289	-0.115326
0.833215	1.556371	1.857975
1.237885	3.647142	-0.062906
-3.591293	-2.129963	0.118824
-4.554866	-1.667406	-0.040438
-3.432168	-3.198468	0.111984
0.688570	2.187685	2.565424

$[CH_3Mo_4O_{13}]^-$ XXXVI 1/2

E = -35142.88

-2.429526	0.567149	-0.289438
-3.163837	0.475576	-1.799972
-1.451473	2.215852	-0.337779
-3.611490	0.845780	0.874848
-0.250635	-1.957843	0.292376
2.827648	-0.366870	-0.361630
3.683097	-0.959104	-1.677578
3.977749	-0.279949	1.159932
1.850971	1.190912	-0.555555
1.613806	-1.650693	0.301353
-0.445997	-3.097383	-0.931662
-0.563222	-2.687414	1.774582
-2.374350	-1.479910	0.049212
0.351053	2.151338	0.223013
-0.522237	-0.023796	0.027336
0.436924	2.065569	1.893171
0.849935	3.703223	-0.177507
-3.398390	-2.341327	0.009088
-4.375303	-1.949214	-0.235247
-3.151284	-3.392486	-0.027663
4.739243	0.301886	1.103851

$[CH_3Mo_4O_{13}]^-$ XXXVII 1/2

E = -35142.80

0.038492	2.103041	-0.172210
-3.482366	0.347091	1.562706
0.071866	2.641492	1.422860
0.152035	3.452184	-1.168993
1.449376	1.006259	-0.492688
-2.950213	0.038109	-0.002716
3.076844	-0.051564	-0.039182
4.164974	-0.164046	-1.334588
3.764526	0.336349	1.461076
-1.640208	1.268614	-0.531911
-0.141387	-0.579802	1.250572
-4.283759	0.130602	-1.022371
-0.236027	-1.927409	-0.178618
-2.183964	-1.637765	-0.105776
-0.031134	-1.559451	-1.804580
1.906897	-1.732464	0.131919
-0.075203	-3.579206	0.112634
0.353440	-0.472689	2.452228
0.673008	-1.362518	2.981801
0.451107	0.523823	2.863554
2.359985	-2.575491	0.088861

$[CH_3Mo_4O_{13}]^-$ XXXVIII 1/2

E = -35142.72

-2.672389	0.310816	-0.110292
-3.534802	0.370399	-1.549235
-1.955604	2.067847	0.100317
-3.770466	0.171717	1.154127
0.017805	-1.865886	0.147400
3.232137	-0.158586	-0.089980
4.025962	-0.694087	-1.490852
4.241832	0.288788	1.194600
1.802406	1.267294	-0.568257
1.714480	-1.272004	0.447127
0.105464	-2.860824	-1.205301
-0.344677	-2.800460	1.499468
-2.190565	-1.672996	-0.156533
-0.061914	2.050762	0.101301
-0.594902	0.076202	-0.065560
0.395157	2.523412	1.638098
0.264618	3.310147	-0.971859
-3.058532	-2.695731	-0.173229
-4.107741	-2.470387	-0.044300
-2.642090	-3.684377	-0.048719
2.172952	1.967228	-1.110751

$[CH_3Mo_4O_{13}]^-$ XXXIX 1/2

E = -35142.71

-2.196337	1.124585	-0.140146
-3.246294	1.696877	-1.308271
-0.679852	2.266471	-0.136277
-2.886235	1.459982	1.353448
-0.904657	-2.105703	-0.034070
2.649392	-1.170902	-0.023612
3.736877	-1.536385	-1.271744
3.246427	-1.337041	1.552772
1.745134	0.623570	-0.304318
0.899261	-2.096244	-0.278776
-1.563400	-3.357440	-0.945855
-1.126478	-2.430265	1.601970
-2.758683	-0.873013	0.053818
1.199637	2.327700	0.077400
-0.798563	-0.225662	-0.918869
1.609280	2.777531	1.644655
1.862736	3.438492	-1.000134
-3.898405	-1.293306	0.632922
-4.460504	-0.573462	1.212297
-4.006902	-2.361362	0.755110
0.118691	0.061136	-1.046315

$[CH_3Mo_4O_{13}]^-$ XL 1/2

E = -35142.62

-2.576385	0.408419	0.049289
-3.390484	0.628488	-1.460246
-1.746717	2.334362	0.019105
-3.497603	0.395038	1.468878
-0.196287	-1.904438	0.141613
2.982598	-0.304616	-0.069913
3.845119	-0.821481	-1.420464
4.064180	-0.029014	1.190347
1.967690	1.188890	-0.466635
1.675615	-1.503690	0.421717
-0.064862	-2.957027	-1.156513
-0.508704	-2.816245	1.510096
-2.230673	-1.653159	-0.289415
0.315771	2.064482	-0.009018
-0.489844	0.134756	0.067956
0.658499	2.721717	1.485284
0.395416	3.293717	-1.149486
-3.105168	-2.592303	-0.669943
-4.012029	-2.239318	-1.140632
-2.752186	-3.612172	-0.728941
-2.105128	2.992883	-0.578545

$[CH_3Mo_4O_{13}]^-$ XLI 1/2

E = -35142.44

-2.310449	0.837654	-0.279938
-2.985983	0.683754	-1.812453
-1.121681	2.288749	-0.420554
-3.511095	1.297492	0.800649
-0.674663	-1.952468	0.329895
2.935544	-0.671439	-0.305587
3.387126	-1.314775	-1.805992
4.180270	-0.480679	0.832531
1.804521	0.932237	-0.496252
1.376068	-1.790304	0.570615
-0.698436	-2.890545	-1.066150
-0.950440	-2.919783	1.684943
-2.639332	-1.303872	0.064612
0.686714	2.196898	0.221043
-0.648313	-0.070474	0.340835
0.719192	2.141447	1.900717
1.328554	3.688004	-0.214308
-3.712316	-2.030816	-0.269209
-4.535079	-1.508934	-0.735927
-3.602951	-3.105939	-0.289850
1.728146	-2.537374	1.060108

$[H_2O]$ I 1/2

E = -2079.39

O	0.000000	0.116100	-0.000000
H	0.761524	-0.464400	-0.000000
H	-0.761524	-0.464400	0.000000

$[CH_3MoO_2]$ I 1/2

E = -7036.70

Mo	0.114670	0.000004	-0.191288
O	0.865529	1.388120	0.371786

O	0.865352	-1.388219	0.371769
C	-1.946682	0.000071	0.228091
H	-2.292941	0.891620	0.749621
H	-2.292909	-0.891210	0.750095
H	-2.397230	-0.000205	-0.782614

$[CH_3MoO_2]$ I 3/4
E = -7034.23

Mo	0.104566	0.058669	0.000012
O	0.342613	1.699398	-0.000030
O	1.420425	-1.350855	-0.000028
C	-1.932608	-0.545112	-0.000034
H	-2.603707	0.315734	0.000459
H	-2.148043	-1.149042	0.888780
H	-2.148676	-1.148481	-0.889054

$[CH_3MoO_4]$ I 1/2
E = -11131.45

Mo	-0.338994	0.034928	0.001669
O	-1.235035	-1.626108	0.039389
O	1.412928	-0.137154	0.558326
O	-0.385086	0.610758	-1.574343
O	-1.182489	1.060728	1.032179
C	2.704944	-0.085026	-0.010928
H	3.305332	0.633337	0.548163
H	2.657660	0.216561	-1.059769
H	3.162542	-1.072501	0.062674

$[CH_3MoO_4]$ I 3/4
E = -11128.22

Mo	-0.489187	-0.027365	-0.011399
O	0.629232	0.928529	1.422622
O	1.430245	0.431897	-0.542828
O	-0.821029	-1.640467	0.363729
O	-1.702897	0.872262	-0.761786
C	2.593310	-0.332693	-0.378680
H	3.408398	0.310276	-0.045539
H	2.841433	-0.734687	-1.368083
H	2.451740	-1.167891	0.310554

$[CH_3MoO_4]$ II 1/2
E = -11129.96

Mo	-0.220933	0.152357	-0.000018
O	1.500126	-0.521396	-0.000071
O	-0.447499	1.800581	0.000031
O	-1.641720	-0.864540	0.712461
O	-1.641816	-0.864539	-0.712382
C	2.892573	-0.324903	0.000034
H	3.317005	-0.793806	-0.889001
H	3.317115	-0.795684	0.888039
H	3.136912	0.739069	0.001207

$[CH_3MoO_4]$ II 3/4
E = -11128.29

Mo	-0.191291	0.281511	0.031942
O	1.388432	-0.695599	-0.311685
O	-0.083665	1.929757	-0.165454
O	-1.871635	-0.776267	0.620328
O	-1.599435	-1.226001	-0.574818
C	2.699675	-0.634095	0.191223
H	3.157269	-1.622279	0.106093
H	2.716566	-0.331890	1.243754
H	3.292774	0.080171	-0.385716

$[CH_3MoO_4]$ III 1/2
E = -11129.84

Mo	0.146201	0.166766	0.000005
O	0.767812	0.866078	1.387599
O	0.767406	0.865242	-1.388195
O	-1.880574	0.379158	0.000141
O	-1.679872	-0.903741	0.000125
C	1.064736	-1.742924	0.000286
H	2.147321	-1.612504	0.000236
H	0.762696	-2.293861	-0.891606
H	0.762941	-2.294149	0.892085

$[CH_3MoO_4]$ IV 1/2
E = -11129.01

Mo	0.662740	0.003312	0.119288
O	0.738737	1.662168	-0.156129
O	2.039619	-0.830826	-0.356975
O	-0.963353	-0.939773	0.106367
O	-2.309634	-0.744217	-0.156454
C	-2.618754	0.629437	-0.062461
H	-3.689419	0.683265	-0.253141
H	-2.076683	1.209736	-0.816157
H	-2.399417	1.012471	0.939506

$[CH_3MoO_4]$ IV 3/4
E = -11126.69

Mo	0.570585	0.071622	0.066797
O	1.037723	-1.809151	-0.149047
O	1.641847	1.184994	-0.542206
O	-1.067459	0.519003	1.042805
O	-1.620167	-0.475432	0.160366
C	-2.576741	0.138174	-0.695656
H	-2.887150	-0.647584	-1.383163
H	-2.132178	0.971589	-1.243933
H	-3.420341	0.483516	-0.099777

$[Mo_3O_{10}]^{2-}$ I 1/2
E = -26057.41

Mo	3.387013	-0.535255	-0.000690
O	1.508284	0.025864	-0.014343
O	-3.699666	-1.492226	-1.389804
O	3.700059	-1.491562	-1.390178
O	3.691480	-1.456646	1.414021
Mo	-0.000062	1.070719	-0.001099
O	-0.000005	2.052696	1.376691
O	-0.000504	2.086525	-1.354481
O	-1.508021	0.025282	-0.013821
Mo	-3.386918	-0.535324	-0.000672
O	-4.395687	0.852670	-0.015373
O	-3.692276	-1.455822	1.414439
O	4.396159	0.852484	-0.014226

$[Mo_3O_{10}]^{2-}$ I 3/4
E = -26053.87

Mo	3.465146	-0.531650	0.000443
O	1.587444	0.040925	-0.020980
O	-3.695189	-1.723658	-1.212587
O	3.692205	-1.723774	-1.212871
O	3.827076	-1.193134	1.542036
Mo	-0.000158	0.952009	-0.006968
O	-0.001210	2.341313	1.140093
O	-0.000706	2.380189	-1.105838
O	-1.586609	0.038984	-0.024133
Mo	-3.464873	-0.531849	0.000422
O	-4.488787	0.809179	-0.307154
O	-3.824441	-1.193188	1.542620
O	4.489613	0.808482	-0.309142

$[Mo_3O_{10}]^{2-}$ II 1/2
E = -26056.27

Mo	-1.531156	-0.995233	-0.014438
O	0.106600	-2.002292	0.275105
O	-1.792441	0.910599	0.266159
O	-2.461216	-1.605683	-1.299562
O	-2.297695	-1.490151	1.429354
Mo	1.628447	-0.826856	-0.014286
O	2.617732	-1.333401	-1.299918
O	0.000206	0.000925	-0.979326
Mo	-0.097202	1.822372	-0.014943
O	1.684691	1.094931	0.269357
O	-0.146592	2.725256	1.434028
O	2.443089	-1.240629	1.428886
O	-0.154840	2.938958	-1.294828

$[Mo_3O_{10}]^{2-}$ II 3/4
E = -26053.83

Mo	-1.623458	-0.871481	-0.012839
O	-0.000165	-1.872782	0.322405
O	-1.773906	0.989490	0.349387
O	-2.485534	-1.367891	-1.390029
O	-2.512130	-1.472977	1.313384
Mo	1.623284	-0.871763	-0.012878
O	2.485254	-1.368372	-1.390057
O	-0.000019	0.104397	-1.070398
Mo	0.000154	1.764172	-0.086911
O	1.774103	0.989198	0.349273
O	0.000470	2.220878	1.864477
O	2.511855	-1.473376	1.313352
O	0.000180	3.141564	-1.070501

$[Mo_3O_{10}]^{2-}$ III 1/2
E = -26053.17

Mo	2.106183	0.002672	0.210913
O	0.522007	-1.300484	0.121698
O	0.519502	1.302372	0.108864
O	2.724343	0.008755	1.780126
O	3.220901	-0.723383	-1.207666
Mo	-1.200585	-1.703642	-0.026284
O	-1.535545	-2.492216	-1.508083
O	-1.734038	-2.643640	1.301346
O	-2.079331	-0.002323	-0.010050
Mo	-1.205158	1.700997	-0.027404
O	-1.732876	2.636249	1.305773
O	-1.551597	2.492858	-1.504912
O	3.219326	0.721665	-1.212526

$[Mo_3O_{10}]^{2-}$ III 3/4
E = -26053.27

Mo	1.980531	-0.345190	-0.208296
O	0.903149	1.370625	-0.238580
O	0.391195	-1.409884	0.041945
O	2.625311	-0.608406	-1.742510
O	3.232857	0.268903	1.128995
Mo	-0.802196	1.929420	0.024967
O	-0.930313	2.860468	1.452904
O	-1.333345	2.872753	-1.299179
O	-1.790627	0.410073	0.171473
Mo	-1.542557	-1.583515	0.045366
O	-2.127162	-2.195645	-1.449334
O	-2.073162	-2.409240	1.456149
O	3.014264	-1.163405	1.202446

$[Mo_3O_{10}]^{2-}$ IV 1/2
E = -26052.43

Mo	1.777999	-0.734689	0.018974
O	1.763207	1.125734	0.179475
O	2.872162	-0.999622	-1.260028
O	2.398623	-1.278646	1.506280
Mo	-0.031383	1.795801	-0.001000
O	-0.183718	2.708741	1.436802
O	0.098387	2.708189	-1.441436
O	-1.805539	1.078798	-0.177176
Mo	-1.753078	-0.782627	-0.017094
O	-2.376805	-1.341710	-1.498048
O	-2.811923	-1.088133	1.283388
O	-0.475269	-2.195680	0.477783
O	0.554798	-2.179718	-0.511665

$[Mo_3O_{10}]^{2-}$ IV 3/4
E = -26052.60

Mo	2.226156	-0.895729	0.048084
O	1.364897	0.847536	0.549295
O	3.240430	-0.626109	-1.312415
O	2.916056	-1.824137	1.315820
Mo	0.001373	1.923553	0.000194
O	-0.487265	2.921653	1.294992
O	0.493126	2.915958	-1.297727
O	-1.365423	0.850118	-0.545744
Mo	-2.227542	-0.893603	-0.047904
O	-2.919281	-1.818584	-1.317158
O	-3.240578	-0.626322	1.314001
O	-0.519164	-1.672879	0.497708
O	0.517271	-1.671897	-0.500734

$[Mo_3O_9]$ I 1/2

E = -24006.19

Mo	-1.998169	-0.360992	0.000305
O	-1.101423	1.301513	0.000534
O	-0.576471	-1.604561	0.003963
O	-2.968385	-0.534153	1.342009
O	-2.961997	-0.536619	-1.345711
Mo	0.686484	1.910761	-0.000162
O	1.018240	2.836254	-1.343407
O	1.019146	2.834244	1.344171
O	1.678074	0.303098	-0.001730
Mo	1.311662	-1.549874	0.000161
O	1.949011	-2.298992	1.343129
O	1.943926	-2.300228	-1.344553

$[Mo_3O_9]$ I 3/4

E = -24003.12

Mo	1.036525	1.731750	-0.011463
O	1.794131	0.001739	-0.000357
O	-0.808377	1.386286	-0.102273
O	1.426958	2.577008	1.372854
O	1.541424	2.621543	-1.329435
Mo	1.040039	-1.729771	-0.011014
O	1.543589	-2.616799	-1.331391
O	1.435761	-2.576124	1.371193
O	-0.805886	-1.388263	-0.096365
Mo	-2.099184	-0.001891	0.035116
O	-2.781945	-0.000162	1.541573
O	-3.226899	-0.005691	-1.492153

$[Mo_3O_9]$ II 1/2

E = -24004.82

Mo	0.000017	-0.730093	-0.000206
Mo	-2.678512	0.372835	0.000103
O	-1.349004	-0.156403	1.235484
O	-1.350572	-0.158581	-1.235651
O	0.000220	-2.376990	0.000321
O	-3.056383	1.999787	-0.001634
O	-4.078628	-0.537361	0.001642
O	1.349614	-0.157052	-1.235661
O	1.349960	-0.157420	1.235623
Mo	2.678444	0.372944	0.000065
O	4.078048	-0.538099	-0.000540
O	3.057010	1.999767	0.000618

$[Mo_3O_9]$ II 3/4

E = -24002.58

Mo	-0.044427	-0.785595	0.000017
Mo	-2.651118	0.395334	-0.000013
O	-1.351778	-0.116292	1.266677
O	-1.352806	-0.117957	-1.266845
O	-0.039316	-2.425251	0.000512
O	-3.023805	2.030178	-0.001098
O	-4.056004	-0.518038	0.001048
O	1.488434	-0.070847	-1.291775
O	1.489118	-0.071919	1.291828
Mo	2.625970	0.342952	-0.000121
O	4.018184	-0.586171	-0.001113
O	3.193241	2.124668	0.001381

$[Mo_4O_{12}]^-$ I 1/2

E = -32012.34

Mo	0.014304	-2.531924	-0.034201
O	-0.001774	-3.506942	1.338150
O	-0.056102	-3.515687	-1.398605
O	-1.355820	-1.371311	-0.008972
Mo	2.707953	-0.000041	0.024308
Mo	-2.810409	0.000051	0.032277
O	-3.755585	0.000016	-1.374151
O	1.626120	-1.502607	-0.071531
O	3.816128	-0.000216	-1.236120
Mo	0.014405	2.531914	-0.034225
O	1.626184	1.502547	-0.071940
O	-0.056094	3.515979	-1.398407
O	-1.355754	1.371341	-0.009137
O	-0.001513	3.506633	1.338342
O	-3.670361	0.000134	1.491877
O	3.571743	0.000108	1.462658

$[Mo_4O_{12}]^-$ I 3/4

E = -32009.01

Mo	-0.784838	-2.410286	-0.236105
O	-0.755936	-3.240638	-1.701880
O	-1.246388	-3.481591	0.977448
O	0.823079	-1.684781	0.122573
Mo	-2.483893	0.782806	0.118303
Mo	2.595433	-0.772631	0.206893
O	3.373634	-0.973104	1.698625
O	-2.039953	-0.976155	-0.400873
O	-2.275227	-0.029916	1.840325
Mo	0.697331	2.488203	-0.187232
O	-1.177199	2.057647	-0.235497
O	0.984109	3.579883	1.063012
O	1.593831	0.956799	0.073929
O	1.128377	3.201465	-1.650734
O	3.548979	-1.074274	-1.161552
O	-4.083478	1.202184	-0.110134

$[Mo_4O_{12}]^-$ II 1/2

E = -32011.98

Mo	2.615069	-0.209861	0.474086
O	0.200229	3.685640	0.074364
O	3.206278	-0.132037	2.045990
O	3.926130	-0.240953	-0.578565
O	1.589429	-1.761832	0.256627
Mo	-0.092555	2.126663	-0.469881
Mo	-0.138570	-2.002346	-0.576056
O	-0.274370	-2.838510	-2.018094
O	1.521405	1.229068	0.114464
O	-0.675646	-0.231620	-0.775589
O	-0.207767	2.264144	-2.136599
Mo	-2.309881	-0.163839	0.577642
O	-1.698651	1.690139	0.354421
O	-3.809570	-0.240029	-0.169924
O	-1.628470	-2.026035	0.559435
O	-2.537831	-0.088716	2.243064

$[Mo_4O_{12}]^-$ II 3/4

E = -32008.87

Mo	0.075366	-2.120026	0.187222
O	-2.846689	-0.000611	-1.844829
O	-0.109429	-3.136718	-1.133416
O	0.197189	-3.065545	1.567429
O	1.913609	-1.582426	-0.051219
Mo	-2.716523	-0.000329	-0.165187
Mo	2.948841	0.000379	-0.197276
O	4.604796	0.000305	-0.418946
O	-1.790800	-1.513558	0.378208
O	0.084707	0.000329	0.186329
O	-4.266924	-0.000663	0.493168
Mo	0.074662	2.120056	0.187155
O	-1.791888	1.513240	0.377966
O	0.194893	3.066995	1.566546
O	1.913188	1.583095	-0.049019
O	-0.109970	3.135138	-1.134764

$[Mo_4O_{12}]^-$ III 1/2

E = -32011.54

Mo	-0.541462	-0.012147	-1.008767
Mo	-4.057987	-0.000337	0.399893
Mo	2.357311	1.724408	0.272656
Mo	2.372960	-1.714280	0.281039
O	3.276104	2.513167	-0.889744
O	-2.114634	-0.013400	-0.177793
O	3.296443	-2.498227	-0.880937
O	0.660693	1.367915	-0.347542
O	-4.127906	-0.244551	2.087450
O	-4.712929	1.519515	-0.021596
O	0.671057	-1.378296	-0.336195
O	-4.860340	-1.260115	-0.428405
O	-0.520350	-0.017498	-2.674380
O	2.322792	2.669628	1.657721
O	2.351268	-2.654890	1.669495
O	3.070986	0.009125	0.631618

$[Mo_4O_{12}]^-$ III 3/4

E = -32008.90

Mo	-0.357291	-0.277984	1.337797
Mo	-3.620383	-0.126140	-0.492788
Mo	1.750730	1.929551	-0.265740
Mo	2.525542	-1.621028	-0.442209
O	1.397767	2.654015	-1.748081
O	-2.013076	-0.425257	0.313638
O	2.086795	-2.258469	-1.951116
O	0.161634	1.415933	0.604796
O	-4.439448	1.169561	0.210320
O	-3.459279	0.106732	-2.154209
O	-0.631064	-0.128994	2.979724
O	-4.747343	-1.638177	-0.283887
O	0.957909	-1.386973	0.790691
O	2.636276	3.013420	0.676062
O	3.826654	-2.383309	0.333305
O	2.655536	0.363424	-0.490809

$[Mo_4O_{12}]^-$ IV 1/2

E = -32011.23

Mo	0.490117	0.047199	0.812420
Mo	4.029005	-0.082038	-0.344599
Mo	-2.259525	1.826382	-0.246474
Mo	-2.484757	-1.840855	-0.215991
O	-3.163665	2.714589	0.856432
O	1.997540	-0.066545	-0.095339
O	-3.391229	-2.583880	1.000609
O	-2.845978	0.121661	-0.370063
O	-0.444912	1.580637	0.379059
O	4.370114	0.083749	-2.006590
O	4.658187	1.233249	0.542033
O	-0.617735	-1.272295	0.402793
O	4.621590	-1.564199	0.257925
O	0.784193	0.027445	2.451174
O	-2.288370	2.606601	-1.733070
O	-2.497650	-2.622122	-1.713083

$[Mo_4O_{12}]^-$ V 1/2

E = -32010.81

Mo	-0.405729	-1.508618	-0.030258
Mo	-3.880338	-0.014998	-0.023627
Mo	0.869982	2.009412	0.017311
Mo	3.176090	-0.513609	0.011415
O	2.626663	1.294993	0.040600
O	0.622818	2.948134	-1.358413
O	-2.286578	-0.905968	-0.089943
O	4.113405	-0.840796	-1.349239
O	1.600337	-1.408690	-0.033458
O	-0.164091	0.521564	0.010269
O	-4.542406	0.421288	1.470885
O	-4.326289	1.016663	-1.287427
O	0.587103	2.957949	1.379566
O	4.072639	-0.897793	1.384463
O	-0.510236	-2.500774	-1.374370
O	-0.533387	-2.460553	1.339154

$[Mo_4O_{12}]^-$ V 3/4

E = -32008.63

Mo	-0.530270	-1.023159	0.846965
Mo	-3.885763	-0.007486	-0.452214
Mo	1.359404	2.031438	0.162829
Mo	2.861332	-0.980243	-0.526215
O	2.782126	0.906957	-0.384033
O	0.933786	3.099337	-1.066325
O	-2.382855	-0.534940	0.468406
O	2.981745	-1.444655	-2.139754
O	1.295810	-1.527977	0.216448
O	0.003226	0.876589	0.533923
O	-5.184706	0.684249	0.385040
O	-3.825046	0.394623	-2.093616
O	1.797433	2.922786	1.522266
O	4.186298	-1.595254	0.311034
O	-1.040507	-2.449443	-0.366982
O	-0.522001	-1.440158	2.448929

$[CH_2Mo_2O_7]$ I 1/2

E = -19118.90

O	2.631367	0.320831	1.374228

O	2.631771	0.317697	-1.374380
Mo	1.692611	0.117307	-0.000007
O	0.556915	-1.388614	0.001503
O	-2.582871	1.235659	-0.000096
O	-1.936715	-1.035348	1.376676
Mo	-1.148667	-0.506704	0.000073
O	0.085329	1.077477	-0.001032
O	-1.936208	-1.036439	-1.376453
C	-2.331364	2.419307	-0.000706
H	-3.160811	3.135575	-0.000063
H	-1.293343	2.773151	-0.002042

$[CH_2Mo_2O_7]$ I 3/4

E = -19115.67

O	2.535254	0.920050	1.207404
O	2.732214	-0.223620	-1.280159
Mo	1.698939	0.134088	-0.012548
O	0.565681	-1.277052	0.550536
O	-2.537352	1.145270	-0.688409
O	-2.042690	-0.396463	1.474825
Mo	-1.120788	-0.515789	0.077259
O	0.082573	0.950836	-0.505809
O	-1.847388	-1.537955	-1.023995
C	-2.585237	2.343415	-0.178249
H	-2.994795	2.381733	0.839978
H	-1.682435	2.940708	-0.363495

$[H_3Mo_4O_{13}]^-$ I 1/2

E = -34108.96

Mo	-2.756326	0.129384	0.375994
O	-0.319790	-3.392618	0.780053
O	-2.828348	0.164474	2.065706
O	-4.321789	0.195198	-0.256501
O	-1.721197	1.491802	-0.256228
Mo	-0.054710	-1.979996	-0.497702
Mo	0.185896	1.949158	-0.487216
O	0.379646	2.475285	-2.046611
O	-0.092205	3.438239	0.682013
O	-1.888233	-1.422728	-0.160264
O	0.559771	-0.075399	-0.324682
O	-0.010010	-2.554452	-2.050068
Mo	2.504018	0.010125	0.441042
O	1.872671	-2.011505	0.211071
O	3.862237	-0.116967	-0.540071
O	1.986332	1.788070	0.353204
O	2.956947	-0.249903	2.044270
H	0.728793	3.739523	1.081186
H	-1.227925	-3.469839	1.083589
H	2.097971	-2.669809	0.871094

$[H_3Mo_4O_{13}]^-$ I 3/4

E = -34108.97

Mo	-2.760560	0.128578	0.373564
O	-0.316733	-3.386294	0.789733
O	-2.837585	0.163798	2.063108
O	-4.323754	0.193731	-0.264755
O	-1.720947	1.488053	-0.257301
Mo	-0.053995	-1.979813	-0.495542
Mo	0.184890	1.948751	-0.484912
O	0.380739	2.481437	-2.041784
O	-0.093392	3.432976	0.690356
O	-1.886888	-1.420466	-0.161522
O	0.564299	-0.075350	-0.330573
O	-0.008369	-2.561483	-2.045177
Mo	2.506822	0.010980	0.438112
O	1.874616	-2.010261	0.211425
O	3.863281	-0.112898	-0.545820
O	1.985918	1.788142	0.355832
O	2.963594	-0.250675	2.040092
H	0.727161	3.729726	1.093849
H	-1.224973	-3.465425	1.092340
H	2.098954	-2.666789	0.873574

$[H_3Mo_4O_{13}]^-$ II 1/2

E = -34108.41

Mo	-2.568140	0.307934	-0.006858
O	-0.600732	-3.301309	1.233773
O	-2.925607	0.379349	1.637274
O	-4.011488	0.595922	-0.817194
O	-1.428207	1.728927	-0.570209
Mo	-0.322368	-2.170790	0.055375
Mo	0.326890	2.258765	-0.150453
O	0.527281	3.754428	-0.883580
O	0.559511	2.483014	1.513506
O	-2.045659	-1.381558	-0.551210
O	-0.188616	-0.259533	0.816019
O	-0.285540	-2.988121	-1.679178
Mo	2.590639	-0.377543	-0.069927
O	1.652019	-1.939125	-0.164447
O	4.136455	-0.412281	-0.707499
O	1.659887	1.170754	-0.817004
O	2.683986	0.321402	1.698792
H	-0.042966	-0.173054	1.761617
H	2.184792	1.125980	1.895621
H	-1.143033	-2.939249	-2.111349

$[H_3Mo_4O_{13}]^-$ II 1/2

E = -34108.52

Mo	2.91798	-0.00413	0.25265
O	0.03658	3.29584	1.18311
O	3.90132	-0.06852	1.62724
O	3.90597	0.21098	-1.10003
O	1.93068	-1.51686	0.09929
Mo	-0.00851	1.72940	-0.41839
Mo	-0.00527	-1.88754	-0.29914
O	-0.05106	-2.95988	-1.56066
O	0.03835	-2.78344	1.40468
O	1.73497	1.45623	0.43309
O	-0.04396	-0.05164	-0.97292
O	-0.03007	2.65783	-1.79099
Mo	-2.89158	0.01401	0.25846
O	-1.72454	1.47256	0.50581
O	-3.74593	0.19314	-1.18636
O	-1.92307	-1.52887	0.26315
O	-4.00066	-0.00906	1.53439
H	-0.85308	-2.91112	1.74130
H	0.83469	3.08780	1.68577
H	-0.73987	3.10374	1.72388

$[H_3Mo_4O_{13}]^-$ III 3/4

E = -34108.43

Mo	-2.568521	0.305440	-0.004861
O	-0.593409	-3.304064	1.230854
O	-2.924547	0.376914	1.639592
O	-4.012999	0.591713	-0.813823
O	-1.430617	1.727436	-0.569862
Mo	-0.319024	-2.171026	0.053773
Mo	0.324416	2.258881	-0.152017
O	0.522747	3.754189	-0.886448
O	0.558142	2.484773	1.511509
O	-2.044430	-1.383275	-0.549575
O	-0.186906	-0.259855	0.815755
O	-0.284374	-2.985926	-1.682147
Mo	2.589994	-0.374539	-0.068820
O	1.653416	-1.937172	-0.167449
O	4.137741	-0.408745	-0.701669
O	1.658309	1.171855	-0.818811
O	2.680754	0.321846	1.701313
H	-0.039317	-0.174325	1.761153
H	2.183597	1.127792	1.897569
H	-1.143255	-2.938707	-2.111786

$[H_3Mo_4O_{13}]^-$ IV 1/2

E = -34108.37

Mo	-0.315092	2.146340	-0.032304
O	-2.900356	-0.250761	1.713569
O	-0.570830	3.273653	1.153989
O	-0.245720	2.997633	-1.748409
O	1.648714	1.879607	-0.298031
Mo	-2.595312	-0.280950	0.057236
Mo	2.551400	0.312528	0.039433
O	4.114014	0.482152	-0.561647
O	2.653697	-0.027699	1.706083
O	-2.041565	1.353424	-0.590533
O	-0.176894	0.236131	0.766600
O	-4.069756	-0.579528	-0.690883
Mo	0.283064	-2.275810	-0.245645
O	-1.511397	-1.784140	-0.446133
O	0.630857	-3.729874	-0.977551
O	1.736460	-1.120214	-0.817332
O	0.861602	-2.415757	1.548175
H	-0.036398	0.186197	1.716220
H	1.539594	-1.790879	1.849324
H	0.655665	3.199124	-2.014954

$[H_3Mo_4O_{13}]^-$ IV 3/4

E = -34108.37

Mo	0.313740	-2.146103	-0.032267
O	2.901694	0.248845	1.713040
O	0.568702	-3.272655	1.154935
O	0.244199	-2.998016	-1.748155
O	-1.649547	-1.878297	-0.297808
Mo	2.596123	0.279654	0.056826
Mo	-2.552296	-0.311082	0.039068
O	-4.114704	-0.480126	-0.562825
O	-2.655014	0.029272	1.705613
O	2.040445	-1.353915	-0.591053
O	0.176663	-0.235756	0.766725
O	4.070445	0.577206	-0.691988
Mo	-0.281577	2.275146	-0.245031
O	1.512941	1.783972	-0.445056
O	-0.628513	3.729457	-0.976849
O	-1.736141	1.121269	-0.816981
O	-0.860138	2.415467	1.548827
H	0.036508	-0.185652	1.716375
H	-1.539131	1.791580	1.849682
H	-0.657254	-3.199566	-2.014495

$[H_3Mo_4O_{13}]^-$ V 1/2

E = -34107.92

Mo	2.272940	0.194994	0.570614
O	-0.483991	3.373980	0.415084
O	1.342138	-0.576361	2.131536
O	3.852822	0.302002	1.102210
O	2.073568	-1.373554	-0.460330
Mo	-0.199845	2.052229	-0.583590
Mo	0.220121	-1.794205	-0.879370
O	0.196636	-2.185167	-2.495182
O	0.313352	-3.309485	0.314289
O	1.683566	1.815767	-0.208602
O	-0.039099	0.048998	-0.215534
O	-0.266189	2.544366	-2.184081
Mo	-2.218827	-0.247864	0.587730
O	-2.116565	1.331489	-0.283643
O	-3.834900	-0.410435	0.980108
O	-1.808253	-1.732204	-0.329939
O	-1.359635	-0.220746	2.224107
H	-0.558790	-3.667955	0.502278
H	1.374280	-1.540410	2.132281
H	-0.367417	-0.277301	2.239095

$[H_3Mo_4O_{13}]^-$ V 3/4

E = -34107.95

Mo	2.297218	-0.195595	0.566875
O	0.050269	3.381041	0.195872
O	1.305610	0.182212	2.234790
O	3.910663	-0.105657	1.022178
O	1.806784	-1.761795	-0.261925
Mo	0.177136	1.962131	-0.698079
Mo	-0.124854	-1.875442	-0.774429
O	-0.153879	-2.167124	-2.408058
O	-0.295922	-3.467513	0.284293
O	1.967627	1.472079	-0.377562
O	-0.016513	-0.015839	-0.121902
O	0.110640	2.341638	-2.332211
Mo	-2.245982	0.128514	0.603261
O	-1.883345	1.567712	-0.382588
O	-3.873544	0.230866	0.971606
O	-2.066552	-1.474913	-0.223357
O	-1.418349	0.162776	2.258034
H	-1.208513	-3.666488	0.510857
H	1.739781	-0.098189	3.041658
H	-0.426918	0.177278	2.273694

$[H_3Mo_4O_{13}]^-$ VI 1/2

E = -34107.74

Mo	0.787264	-2.158598	-0.226212
O	2.586863	0.548277	1.693269
O	1.476442	-2.295430	1.530033
O	1.408970	-3.444131	-1.086390
O	-1.096560	-2.211820	-0.329999
Mo	2.338270	0.813102	0.034558
Mo	-2.338284	-0.813210	0.034505
O	-3.779379	-1.411447	-0.587219
O	-2.586826	-0.548355	1.693224
O	2.064683	-0.784779	-0.835940
O	0.000174	-0.000041	0.584686
O	3.779339	1.411463	-0.587103
Mo	-0.787278	2.158736	-0.226229
O	1.096473	2.211597	-0.330475
O	-1.409055	3.444567	-1.085897
O	-2.064607	0.784769	-0.835778
O	-1.476378	2.295199	1.530107
H	-0.000256	0.000146	1.546510
H	-1.964832	1.530043	1.867598
H	1.965157	-1.530433	1.867616

$[H_3Mo_4O_{13}]^-$ VI 3/4
E = -34107.73

Mo	0.801133	-2.155661	-0.221348
O	2.575973	0.572259	1.690960
O	1.479682	-2.275128	1.540493
O	1.437879	-3.443998	-1.066799
O	-1.081690	-2.207225	-0.355613
Mo	2.334584	0.824538	0.029244
Mo	-2.334563	-0.824509	0.029203
O	-3.774409	-1.430770	-0.587461
O	-2.576303	-0.572275	1.690861
O	2.075328	-0.778321	-0.835389
O	-0.000214	0.000016	0.581273
O	3.774554	1.430767	-0.587148
Mo	-0.801146	2.155652	-0.221234
O	1.081711	2.207150	-0.355802
O	-1.437993	3.443980	-1.066640
O	-2.075258	0.778350	-0.835478
O	-1.479444	2.275116	1.540729
H	0.000279	0.000136	1.543063
H	-1.955998	1.502929	1.879579
H	1.956882	-1.503277	1.879186

$[H_3Mo_4O_{13}]^-$ VII 1/2
E = -34107.71

Mo	-2.384186	0.408112	-0.093192
O	0.021048	-2.579517	1.784731
O	-3.006549	0.579192	1.454139
O	-3.669178	0.609465	-1.157926
O	-1.376796	1.965712	-0.560993
Mo	-0.459169	-2.376927	-0.046342
Mo	0.391879	2.353291	-0.025781
O	0.648875	3.897925	-0.631718
O	0.602606	2.449289	1.654703
O	-2.046796	-1.463268	-0.440391
O	-0.237697	-0.025576	0.581712
O	-0.531978	-3.913549	-0.691385
Mo	2.397593	-0.420910	-0.238298
O	1.348609	-1.878199	-0.629356
O	3.965681	-0.518459	-0.804754
O	1.741877	1.325613	-0.779558
O	2.483887	-0.087105	1.643202
H	-0.208015	0.005224	1.542329
H	2.107437	0.747260	1.953982
H	0.814934	-2.114431	2.076221

$[H_3Mo_4O_{13}]^-$ VII 3/4
E = -34107.73

Mo	2.337590	-0.612062	-0.089658
O	0.226953	2.585544	1.783342
O	2.935450	-0.824780	1.462171
O	3.605766	-0.932026	-1.145668
O	1.201523	-2.079072	-0.556794
Mo	0.664690	2.331927	-0.051199
Mo	-0.595240	-2.314753	-0.029909
O	-0.984629	-3.830165	-0.638771
O	-0.819262	-2.392683	1.649969
O	2.160759	1.276580	-0.453684
O	0.234490	0.009091	0.581328
O	0.870323	3.851253	-0.708582
Mo	-2.349566	0.624419	-0.234966
O	-1.186071	2.000131	-0.599754
O	-3.903179	0.865488	-0.797765
O	-1.845413	-1.170329	-0.786236
O	-2.474737	0.286678	1.643979
H	0.196014	-0.023154	1.541572
H	-2.180648	-0.582936	1.947041
H	-0.605047	2.200085	2.083838

$[H_3Mo_4O_{13}]^-$ VIII 1/2
E = -34107.39

Mo	-0.034791	2.187648	-0.143174
O	-2.726065	0.461750	1.722045
O	-0.089552	2.384364	1.542054
O	-0.071170	3.713340	-0.846879
O	1.535231	1.367470	-0.675734
Mo	-2.704884	-0.126597	-0.077125
Mo	2.702184	-0.061222	-0.069646
O	4.195546	0.215490	-0.774195
O	2.749738	0.450896	1.759116
O	-1.537257	1.261679	-0.732563
O	0.075088	-0.519062	0.932722
O	-4.199899	0.113298	-0.784071
Mo	-0.005818	-2.018568	0.077375
O	-1.935051	-1.802479	-0.244904
O	0.039146	-2.442912	-1.803018
O	1.979914	-1.721974	-0.342341
O	0.098097	-3.321224	1.124087
H	1.973228	0.920409	2.088234
H	0.918482	-2.424857	-2.185657
H	-1.982853	1.006474	2.014815

$[H_3Mo_4O_{13}]^-$ VIII 3/4
E = -34107.36

Mo	-0.037591	2.191786	-0.153181
O	-2.699241	0.451378	1.734244
O	-0.093404	2.397057	1.530553
O	-0.073874	3.714828	-0.862471
O	1.534600	1.372334	-0.677029
Mo	-2.700147	-0.129967	-0.067345
Mo	2.697224	-0.061220	-0.060325
O	4.198265	0.221906	-0.744855
O	2.727579	0.450235	1.769051
O	-1.540431	1.264835	-0.735925
O	0.073203	-0.517903	0.921975
O	-4.204550	0.113840	-0.752137
Mo	-0.002483	-2.020371	0.068806
O	-1.929522	-1.802311	-0.265768
O	0.043152	-2.452851	-1.810693
O	1.979338	-1.722290	-0.352057
O	0.095473	-3.321676	1.117809
H	1.948213	0.918866	2.092365
H	0.923592	-2.434421	-2.190887
H	-1.950652	0.990926	2.022844

$[H_3Mo_4O_{13}]^-$ XI 1/2
E = -34106.97

Mo	-3.212776	-1.553131	-0.201889
O	-3.960799	-0.640899	1.272245
O	-1.815548	-2.636222	0.330623
O	-4.410788	-2.237559	-1.137088
Mo	-1.943783	1.717493	-0.509902
Mo	0.949625	0.416764	1.078755
O	0.076739	-1.032519	1.352741
O	1.288966	1.162013	2.545854
O	2.448834	0.102960	0.179319
O	-0.166224	1.554337	0.008939
O	-2.885064	2.188369	0.813838
O	-2.121336	2.847933	-1.736424
O	-2.536487	0.054132	-1.050425
Mo	4.239053	-0.388167	-0.604927
O	4.109539	-1.256220	-2.062687
O	5.342777	0.906442	-0.623906
O	4.583568	-1.579029	0.890466
H	-1.039349	-2.240760	0.792795
H	3.907223	-1.648872	1.567774
H	-3.850261	0.315458	1.365889

$[H_3Mo_4O_{13}]^-$ IX 3/4
E = -34106.97

Mo	-3.210210	-1.555398	-0.199111
O	-3.961951	-0.640865	1.271569
O	-1.812738	-2.635541	0.339006
O	-4.405697	-2.243708	-1.134726
Mo	-1.944724	1.715774	-0.512637
Mo	0.949507	0.423216	1.076934
O	0.074207	-1.023025	1.359301
O	1.292220	1.175147	2.539819
O	2.446815	0.102435	0.176693
O	-0.165588	1.557170	0.002441
O	-2.884348	2.185277	0.812842
O	-2.127118	2.845194	-1.739362
O	-2.534986	0.050766	-1.050758
Mo	4.237831	-0.391142	-0.604086
O	4.109904	-1.263694	-2.059307
O	5.341280	0.903604	-0.625949
O	4.581012	-1.577248	0.895408
H	-1.038231	-2.237371	0.801500
H	3.903858	-1.645118	1.572114
H	-3.850680	0.315522	1.364354

$[H_3Mo_4O_{13}]^-$ X 1/2
E = -34106.93

Mo	-3.379752	-1.334980	-0.277147
O	-3.895938	-0.945952	1.287943
O	-2.085022	-2.662160	-0.249439
O	-4.687879	-1.873556	-1.178456
Mo	-1.850195	1.828245	-0.303936
Mo	0.961383	0.014444	0.854779
O	-0.077186	-1.339115	0.994208
O	1.528104	0.463318	2.375319
O	2.326601	-0.343820	-0.219213
O	-0.070801	1.450685	0.105484
O	-2.861394	1.876448	1.282624
O	-2.240113	3.101473	-1.303513
O	-2.659046	0.222272	-0.988109
Mo	4.341423	-0.280424	-0.544709
O	4.890336	-1.557111	-1.526934
O	4.895473	1.273478	-0.967715
O	4.645993	-0.633338	1.339984
H	-1.278377	-2.441076	0.265797
H	3.931396	-0.468578	1.961405
H	-3.386081	1.102747	1.537843

$[H_3Mo_4O_{13}]^-$ X 3/4
E = -34106.94

Mo	-3.361746	-1.352885	-0.265721
O	-3.889957	-0.952441	1.292443
O	-2.051904	-2.664704	-0.215826
O	-4.659029	-1.917111	-1.166912
Mo	-1.861154	1.823661	-0.321650
Mo	0.961082	0.051882	0.856200
O	-0.079301	-1.295440	1.038272
O	1.541398	0.538337	2.360054
O	2.317573	-0.335701	-0.219531
O	-0.073946	1.470005	0.076702
O	-2.862711	1.867881	1.271029
O	-2.271570	3.087285	-1.325518
O	-2.655154	0.203946	-0.991596
Mo	4.331954	-0.296552	-0.543837
O	4.869794	-1.596271	-1.501510
O	4.900370	1.243644	-0.996400
O	4.634679	-0.616357	1.346837
H	-1.253919	-2.428353	0.305868
H	3.926182	-0.423881	1.967322
H	-3.379898	1.091194	1.532733

$[H_3Mo_4O_{13}]^-$ XI 1/2
E = -34106.65

Mo	-0.443980	-0.955249	-0.294181
Mo	-4.158335	-0.045885	0.197542
Mo	1.705280	2.000421	-0.051583
Mo	3.232646	-1.067374	0.246096
O	3.178009	0.815702	-0.032440
O	1.924862	3.181641	-1.234504

O	-2.152528	0.195519	-0.342652
O	-5.046563	-1.188820	-0.695284
O	4.122594	-1.858703	-1.227362
O	1.412326	-1.382879	0.102824
O	0.258572	0.960604	-0.410655
O	-4.519953	0.011322	1.857667
O	-4.185243	1.733006	-0.619628
O	1.557446	2.751029	1.449892
O	3.749661	-1.344676	1.807571
O	-0.629133	-1.733310	-1.762617
O	-1.158636	-1.885509	0.904359
H	-2.029300	1.077761	-0.714168
H	4.850938	-2.476099	-1.151780
H	-5.008601	2.218593	-0.622107

$[H_3Mo_4O_{13}]^-$ XI 3/4
E = -34106.65

Mo	-0.443608	-0.956443	-0.286808
Mo	-4.159762	-0.046084	0.193530
Mo	1.704017	2.000800	-0.050527
Mo	3.234941	-1.066365	0.241752
O	3.177793	0.817324	-0.031833
O	1.922368	3.182628	-1.233076
O	-2.151488	0.196071	-0.335230
O	-5.042377	-1.189926	-0.703784
O	4.118621	-1.853429	-1.237567
O	1.413921	-1.381837	0.105899
O	0.258461	0.959477	-0.409702
O	-4.529943	0.011762	1.851839
O	-4.182624	1.732868	-0.623909
O	1.555495	2.750749	1.451192
O	3.757407	-1.349082	1.800432
O	-0.632729	-1.738888	-1.752460
O	-1.153681	-1.883593	0.916613
H	-2.026214	1.078788	-0.704944
H	4.847452	-2.470845	-1.167016
H	-5.005677	2.218938	-0.629115

$[H_3Mo_4O_{13}]^-$ XII 1/2
E = -34106.38

Mo	0.484311	-0.891226	0.082325
Mo	4.232621	0.066925	-0.039492
Mo	-1.742659	2.004383	-0.063474
Mo	-3.195571	-1.163524	-0.146548
O	-3.149452	0.710965	-0.077110
O	-2.153459	2.699371	1.666454
O	2.211565	0.346825	0.152692
O	4.704566	-1.321559	0.830019
O	-3.872926	-1.785501	1.516010
O	-1.346279	-1.403466	-0.191276
O	-0.189304	1.026283	-0.069269
O	4.646155	-0.076878	-1.687985
O	4.917821	1.484905	0.629382
O	-2.014141	3.099174	-1.299407
O	-3.908125	-1.619323	-1.585649
O	0.768068	-1.560422	1.588644
O	1.229855	-1.821036	-1.094457
H	1.930529	1.262975	0.223015
H	-4.542099	-2.465516	1.600451
H	-2.848700	2.272417	2.174044

$[H_3Mo_4O_{13}]^-$ XII 3/4
E = -34106.40

Mo	0.491551	-0.917917	0.149769
Mo	4.224829	0.085271	-0.074139
Mo	-1.752619	1.983629	-0.051165
Mo	-3.180114	-1.133311	-0.178968
O	-3.199271	0.730508	0.094105
O	-2.046531	2.765534	1.663279
O	2.198964	0.340881	0.119012
O	4.739579	-1.206149	0.912702
O	-4.016622	-1.908108	1.341722
O	-1.343772	-1.433304	-0.072487
O	-0.220106	0.974628	-0.106426
O	4.624746	-0.200374	-1.706856
O	4.881179	1.574577	0.453835
O	-2.086285	3.008518	-1.331095
O	-3.726996	-1.452111	-1.723763
O	0.794565	-1.499738	1.688467

O	1.219464	-1.916071	-0.982325
H	1.906324	1.256041	0.133907
H	-4.701902	-2.575255	1.290440
H	-2.668912	2.346691	2.263431

$[CH_3Mo_2O_7]^-$ I 1/2
E = -19139.21

O	2.763950	-0.953613	1.128119
O	2.696801	0.012370	-1.506167
Mo	1.868551	-0.049637	-0.012042
O	0.093346	-0.881198	-0.291814
O	-1.907917	1.372699	-0.609798
O	-2.022408	-0.445298	1.624373
Mo	-1.642065	-0.379965	-0.019426
O	1.557426	1.539110	0.573720
O	-2.688676	-1.392354	-0.873184
C	-1.525405	2.565620	0.051446
H	-1.787054	3.406928	-0.593858
H	-0.448749	2.571299	0.245418
H	-2.064327	2.657639	0.999429

$[CH_3Mo_2O_7]^-$ I 3/4
E = -19136.05

O	2.544417	-0.767354	1.457286
O	2.550142	-0.979543	-1.335847
Mo	1.923131	-0.148878	0.004787
O	0.136602	-0.148186	0.002640
O	-2.040244	1.624380	-0.029331
O	-2.498326	-0.948332	1.455802
Mo	-1.875356	-0.313647	-0.001300
O	2.586601	1.635678	-0.138664
O	-2.485529	-0.992782	-1.443774
C	-1.022535	2.576626	0.008337
H	-1.467832	3.578924	-0.009095
H	-0.342102	2.497212	-0.849691
H	-0.410692	2.499264	0.917395

$[CH_3Mo_2O_7]^-$ II 1/2
E = -19138.27

Mo	1.847627	-0.148262	0.000113
Mo	-1.857442	-0.122558	0.000276
O	0.007725	0.777786	0.009727
O	-2.055682	-1.071969	1.399526
O	-2.935110	1.208553	0.007815
O	2.657808	0.403164	-1.399693
O	2.661956	0.370372	1.410181
O	-2.054714	-1.054323	-1.410895
O	1.621120	-1.835742	-0.019343
C	0.121477	2.215056	0.001183
H	-0.874298	2.651116	0.049465
H	0.623082	2.523559	-0.917440
H	0.709756	2.526669	0.866008

$[CH_3Mo_2O_7]^-$ II 3/4
E = -19135.40

Mo	1.807279	-0.161815	0.000289
Mo	-1.793987	-0.204680	-0.000595
O	0.041217	0.851795	0.000206
O	-2.184700	-0.977895	1.463854
O	-2.736942	1.534795	0.009421
O	2.659905	0.310816	-1.404309
O	2.659539	0.307208	1.406358
O	-2.186283	-0.967302	-1.469989
O	1.448811	-1.831779	-0.001935
C	0.190118	2.279440	-0.001642
H	-0.798730	2.732999	0.013615
H	0.730264	2.581199	-0.901584
H	0.757114	2.580852	0.881842

$[H_2]$ I 1/2
E = -31.73

H	0.000000	0.000000	0.372080
H	-0.000000	-0.000000	-0.372080

$[MoO_3]$ I 1/2
E = -7999.00

Mo	0.000086	-0.000071	-0.184904
O	1.605651	-0.148590	0.323684
O	-0.674239	1.464910	0.323470
O	-0.931864	-1.315945	0.323591

$[MoO_3]$ I 3/4
E = -7997.30

Mo	0.023016	0.000076	-0.133289
O	0.861911	1.403792	0.241510
O	-1.842379	0.001059	0.216870
O	0.859634	-1.405252	0.241385

$[MoO_3]$ I 5/6
E = -7994.59

Mo	0.000129	0.061056	0.000016
O	1.611032	-1.020115	-0.000025
O	0.004314	1.711938	-0.000032
O	-1.616022	-1.012369	-0.000025

$[MoO_3]$ I 7/8
E = -7991.01

Mo	-0.000141	-0.006658	-0.023175
O	1.702387	-0.960741	0.040935
O	0.014687	1.932091	0.039784
O	-1.716336	-0.936397	0.040953

$[Mo_3O_{11}]^-$ I 1/2
E = -28100.94

Mo	1.267652	1.715875	0.065650
O	2.071783	-0.027806	-0.131065
O	-2.112963	0.943745	-1.709244
O	-0.431386	1.313609	0.353625
O	1.468105	2.625452	-1.347221
Mo	1.277416	-1.711395	-0.096724
O	1.683790	-2.586194	-1.476302
O	1.793500	-2.584964	1.246143
O	-0.537934	-1.404714	-0.023424
Mo	-2.048170	-0.198193	0.213075
O	-2.362831	-0.018626	1.849191
O	-3.257820	-1.220613	-0.334379
O	1.938343	2.559556	1.370400
O	-2.861305	1.417552	-0.753227

$[Mo_3O_{11}]^-$ II 1/2
E = -28098.56

Mo	3.348089	-0.515628	-0.007260
O	1.801885	0.426080	-0.268516
O	-2.945501	-1.238324	-1.536055
O	3.364999	-2.135650	-0.976066
O	3.565048	-0.919683	1.611451
Mo	0.033137	1.181738	-0.007871
O	0.281496	2.525971	0.967395
O	-0.461230	1.647040	-1.541581
O	-1.665126	0.419208	0.759551
Mo	-3.328903	-0.563584	-0.018365
O	-4.517858	0.651206	-0.170098
O	-3.823136	-1.767598	1.083157
O	4.676047	0.365065	-0.555366
O	-0.551320	-0.511577	0.801977

$[Mo_3O_{11}]^-$ II 3/4
E = -28098.27

Mo	2.075950	-0.662453	0.019541
O	1.309739	1.118600	-0.243506
O	-2.348105	-1.601968	-1.541833
O	1.620867	-2.354946	-0.693230
O	2.733234	-0.803010	1.554623
Mo	-0.255498	2.070913	-0.022424
O	-0.352783	2.700912	1.535041
O	-0.317555	3.354805	-1.109509
O	-1.670508	0.900593	-0.336701
Mo	-1.867426	-0.999540	-0.042491
O	-3.106424	-1.145110	1.092182
O	-0.982258	-2.796573	0.503378
O	3.381385	-0.537846	-1.053266
O	-0.020977	-0.982284	0.531037

[Mo_2O_7] I 1/2
E = -18046.39

O	-2.329493	-1.504192	0.569812
O	-2.457411	0.176935	-1.581086
Mo	-1.869994	-0.010080	-0.025200
O	-2.703361	1.294258	1.037573
O	2.329612	-1.505488	-0.566309
O	2.457084	0.180634	1.580782
Mo	1.870002	-0.010048	0.025200
O	0.000003	0.171613	-0.000385
O	2.703525	1.291908	-1.040385

[Mo_2O_7] I 3/4
E = -18046.39

O	-2.328898	-1.503865	0.571068
O	-2.458340	0.175695	-1.580913
Mo	-1.870015	-0.010103	-0.025238
O	-2.702946	1.294980	1.036995
O	2.330080	-1.505442	-0.566037
O	2.456224	0.180661	1.581035
Mo	1.870004	-0.010031	0.025150
O	0.000010	0.171785	-0.001468
O	2.703928	1.291890	-1.040218

[Mo_2O_7] II 1/2
E = -18045.68

O	-2.510212	1.372408	0.079852
O	-0.010276	0.000078	1.262220
Mo	-1.554357	0.000073	0.038759
O	1.669735	1.577744	-0.844830
O	-2.509323	-1.372848	0.079248
Mo	1.391497	0.000014	0.071126
O	2.815562	0.000380	0.954491
O	1.669011	-1.578633	-0.843878
O	-0.269479	0.000415	-1.263996

[Mo_2O_7] II 3/4
E = -18045.72

O	-2.511893	1.372486	0.082675
O	-0.006256	0.000407	1.259538
Mo	-1.555773	0.000037	0.039326
O	2.824141	-0.000951	0.951993
O	-2.511614	-1.372711	0.082930
Mo	1.392500	0.000022	0.067819
O	1.670401	-1.577160	-0.836213
O	1.671720	1.577520	-0.835469
O	-0.279315	0.000100	-1.267965

[MoO_4] I 1/2
E = -10044.06

Mo	-0.000361	0.023537	0.000105
O	-1.572212	-1.017343	0.001190
O	-0.003590	0.972678	-1.382438
O	0.015455	0.952088	1.396489
O	1.562241	-1.030992	-0.015794

[MoO_4] I 3/4
E = -10044.07

Mo	-0.000060	-0.020699	0.000151
O	1.566968	1.020028	-0.025617
O	-1.571819	1.014048	-0.000340
O	-0.011646	-0.980953	-1.376799
O	0.016814	-0.944451	1.401962

[Mo_2O_5] I 1/2
E = -13953.61

Mo	-1.523799	0.176138	0.000024
Mo	1.278647	-0.043491	-0.000007
O	-0.105546	0.195053	-1.286286
O	-0.105466	0.195831	1.286260
O	-2.940131	-0.685200	-0.000090
O	2.447385	1.156817	-0.000385
O	1.990803	-1.558901	0.000408

[Mo_2O_5] I 3/4
E = -13953.08

Mo	-1.526531	0.224354	-0.000453
Mo	1.333380	-0.049948	0.000094
O	-0.330429	0.097448	-1.312670
O	-0.330509	0.102064	1.312155
O	-2.784541	-0.873168	0.001875
O	2.343519	1.293372	-0.001063
O	2.116003	-1.535346	0.001587

[Mo_2O_5] I 5/6
E = -13950.49

Mo	-1.518325	-0.195464	-0.000067
Mo	1.304662	0.050934	0.000031
O	-0.070013	-0.183439	-1.278738
O	-0.070289	-0.184570	1.278448
O	-3.239133	0.698886	0.000239
O	2.017418	1.569245	0.000526
O	2.483752	-1.141341	-0.000284

[Mo_2O_5] I 7/8
E = -13947.72

Mo	-1.572970	-0.015525	0.000132
Mo	1.302515	0.027752	-0.000131
O	0.123761	-0.011119	-1.318175
O	0.123296	-0.017016	1.317327
O	-3.567008	-0.020058	-0.000231
O	2.247693	1.416077	0.003273
O	2.492149	-1.432081	-0.002198

Table S5: Energies of calculated structures on the M06/def2TZVP level of theory. See Figures for the respective structures.

Ion	Isomer	Singlet/Douplet	Triplet/Quartet	Pentet/Hextet
$[MoO_4]^-$	I	0.00	3.20	-
	II	3.41	3.99	-
$[Mo_2O_8]^{2-}$	I	0.00	2.09	-
	II	2.20	1.56	-
	III	1.87	1.87	-
$[Mo_3O_9]^-$	I	0.00	3.09	-
	II	0.16	3.38	-
$[Mo_2O_6]$	I	0.00	2.68	-
$[Mo_2O_5]^-$	I	0.00	0.22	3.46
$[Mo_2O_6]^-$	I	0.00	3.52	-
$[Mo_2O_7]^-$	I	0.00	2.91	5.64
	II	0.17	3.21	-
$[Mo_2O_8]^-$	I	0.00	2.90	-
	II	2.45	2.38	-
	III	2.50	2.50	-
$[Mo_4O_{13}]^{2-}$	I	0.00	2.52	-
	II	0.41	-	-
	III	0.60	3.30	-
	IV	0.60	4.47	-
	V	0.78	3.19	-
	VI	0.95	2.90	-
	VII	2.87	3.80	-
	VIII	3.25	3.25	-
$[Mo_2O_5]$	I	0.02	0.00	-
$[MoO_3]$	I	0.00	1.80	-
$[Mo_3O_{10}]^-$	I	0.00	2.79	-
	II	0.56	2.76	-
	III	0.68	3.21	-
	IV	1.40	3.86	-
$[Mo_2O_7]$	I	0.00	-	-
	II	1.05	1.05	-
	III	1.60	1.56	-
$[Mo_3O_{10}]^{2-}$	I	0.00	3.18	-
	II	0.85	3.34	-
	III	0.85	3.56	-

	IV	4.40	2.94	-
	V	3.90	4.16	-
$[Mo_3O_9]$	I	0.00	3.12	-
	II	1.22	3.46	-
$[Mo_2O_7]^{2-}$	I	0.00	3.37	6.44
$[MoO_3]^-$	I	0.00	2.90	-
$[Mo_2O_6]^{2-}$	I	0.00	0.45	-

Calculated structures on the M06/def2TZVP level of theory

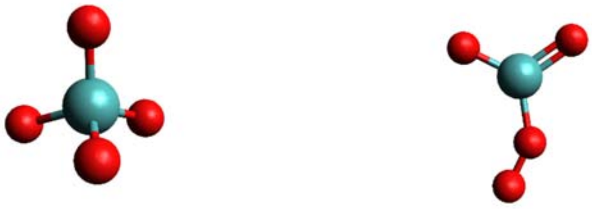

I E=0.00 II E=3.41

Figure S4.1: Calculated Molecules of $[MoO_4]^-$ in lowest multiplicity unless marked with *.

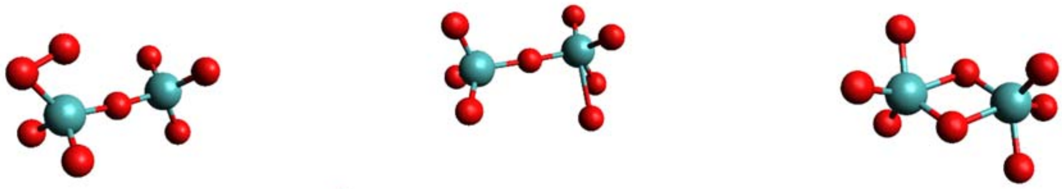

I E=0.00 II* E=1.56 III E=1.87

Figure S4.2: Calculated Molecules of $[Mo_2O_8]^{2-}$ in lowest multiplicity unless marked with *.

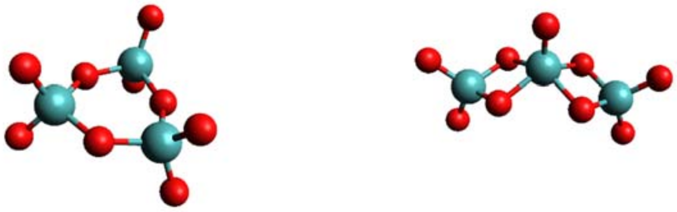

I E=0.00 II E=0.16

Figure S4.3: Calculated Molecules of $[Mo_3O_9]^-$ in lowest multiplicity unless marked with *.

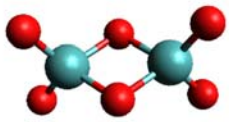

I E=0.00

Figure S4.4: Calculated Molecules of $[Mo_2O_6]$ in lowest multiplicity unless marked with *.

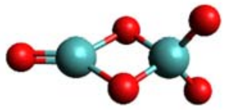

I E=0.00

Figure S4.5: Calculated Molecules of $[Mo_2O_5]^-$ in lowest multiplicity unless marked with *.

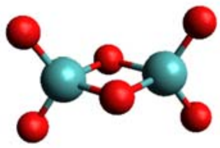

I E=0.00

Figure S4.6: Calculated Molecules of $[Mo_2O_6]^-$ in lowest multiplicity unless marked with *.

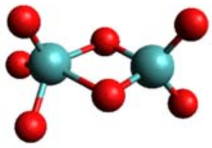

I E=0.00

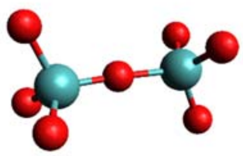

II E=0.17

Figure S4.7: Calculated Molecules of $[Mo_2O_7]^-$ in lowest multiplicity unless marked with *.

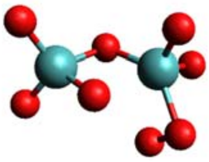

I E=0.00

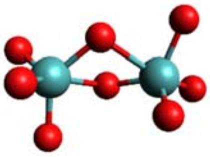

II* E=2.38

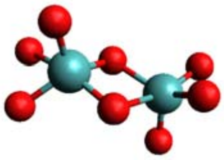

III E=2.50

Figure S4.8: Calculated Molecules of $[Mo_2O_8]^-$ in lowest multiplicity unless marked with *.

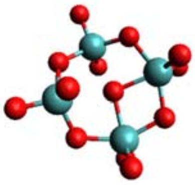

I E=0.00

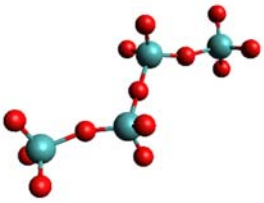

II E=0.41

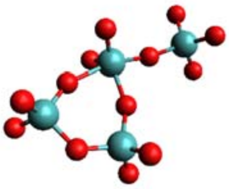

III E=0.60

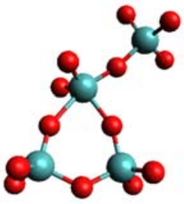

IV E=0.60

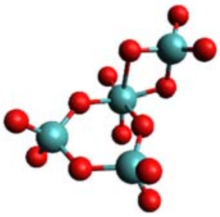

V E=0.78

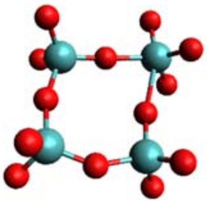

VI E=0.95

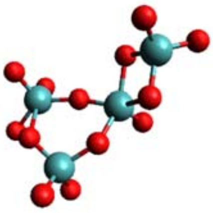

VII E=2.87

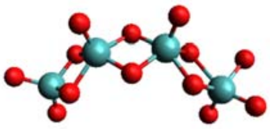

VIII E=3.25

Figure S4.9: Calculated Molecules of $[Mo_4O_{13}]^{2-}$ in lowest multiplicity unless marked with *.

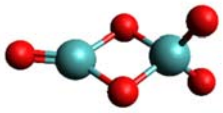

I* E=0.00

Figure S4.10: Calculated Molecules of [Mo_2O_5] in lowest multiplicity unless marked with *.

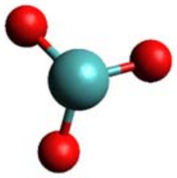

I E=0.00

Figure S4.11: Calculated Molecules of [MoO_3] in lowest multiplicity unless marked with *.

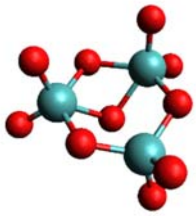

I E=0.00

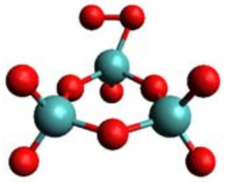

II E=0.56

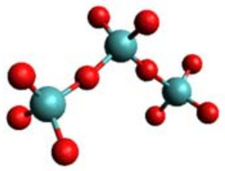

III E=0.68

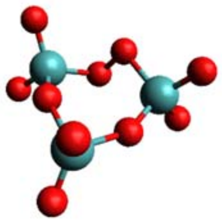

IV E=1.40

Figure S4.12: Calculated Molecules of [Mo_3O_{10}]$^-$ in lowest multiplicity unless marked with *.

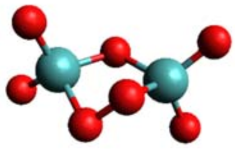

I E=0.00

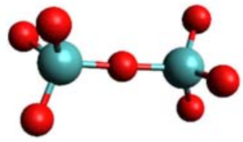

II E=1.05

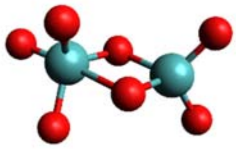

III* E=1.56

Figure S4.13: Calculated Molecules of [Mo_2O_7] in lowest multiplicity unless marked with *.

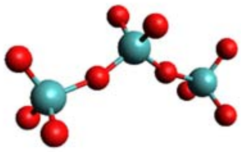

I E=0.00

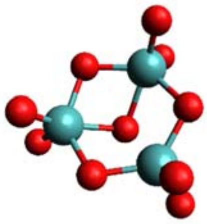

II E=0.85

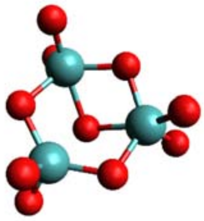

III E=0.85

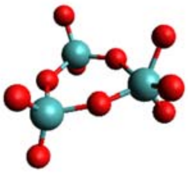

IV* E=2.94

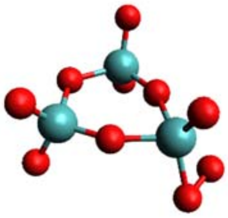

V E=3.90

Figure S4.14: Calculated Molecules of $[Mo_3O_{10}]^{2-}$ in lowest multiplicity unless marked with *.

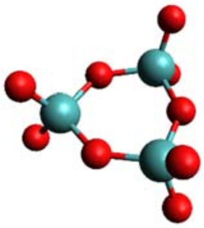

I E=0.00

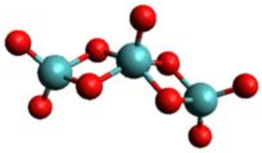

II E=1.22

Figure S4.15: Calculated Molecules of [Mo_3O_9] in lowest multiplicity unless marked with *.

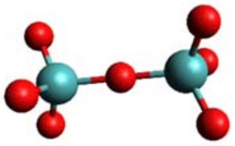

I E=0.00

Figure S4.16: Calculated Molecules of $[Mo_2O_7]^{2-}$ in lowest multiplicity unless marked with *.

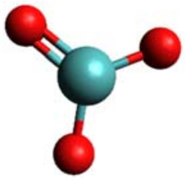

I E=0.00

Figure S4.17: Calculated Molecules of $[MoO_3]^{-}$ in lowest multiplicity unless marked with *.

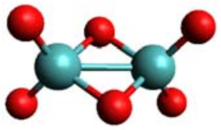

I E=0.00

Figure S4.18: Calculated Molecules of $[Mo_2O_6]^{2-}$ in lowest multiplicity unless marked with *.

Cartesian coordinates (in Ångstrom) of species optimized at the M06/def2TZVP level of theory along the respective ZPE-corrected energy (in eV)

$[MoO_4]^{-}$ I 1/2

E = -10046.92

Mo	-0.039945	-0.004273	0.001240
O	-0.898411	0.842776	1.230894
O	-0.898999	0.077302	-1.489276
O	1.723282	0.750670	-0.211360
O	0.283840	-1.648315	0.463233

$[MoO_4]^{-}$ I 3/4

E = -10043.72

Mo	-0.158060	0.000009	0.010131
O	1.402610	0.002414	-1.162521
O	1.536938	-0.002044	1.083867
O	-1.055098	1.469004	0.014445
O	-1.054636	-1.469423	0.011024

$[MoO_4]^{-}$ II 1/2

E = -10043.51

Mo	0.331928	0.006218	-0.000047
O	0.997349	1.592422	0.000084
O	1.403968	-1.340948	0.000122
O	-1.456601	-0.389933	-0.000056
O	-2.687337	0.105816	0.000095

$[MoO_4]^{-}$ II 3/4

E = -10042.93

Mo	0.335024	0.000002	-0.036200
O	1.117660	1.532566	0.131898
O	1.121515	-1.530630	0.132281
O	-1.660909	-0.001203	-0.599433
O	-2.337141	-0.000743	0.525302

$[Mo_2O_8]^{2-}$ I 1/2

E = -20093.70

Mo	2.021613	0.021579	0.000068
Mo	-1.774121	-0.190522	0.000111
O	-1.763173	1.769293	0.000887
O	0.128502	-0.187157	0.002767
O	-2.194577	-1.070386	-1.408493
O	-2.198181	-1.071310	1.407083
O	2.532863	0.883732	-1.407764
O	2.763896	-1.540766	-0.000613
O	-3.105061	1.219401	-0.001196
O	2.536396	0.884140	1.406386

$[Mo_2O_8]^{2-}$ I 3/4

E = -20091.61

Mo	-1.995393	-0.006758	0.000421
Mo	1.801325	-0.275639	0.000075
O	2.064878	1.812880	0.656087
O	-0.104018	0.032365	0.006199
O	2.381492	-1.058255	1.420060
O	2.374397	-1.056331	-1.423764
O	-2.604639	0.707786	1.455788
O	-2.556604	-1.642852	-0.097334
O	2.061861	1.813128	-0.655906
O	-2.598514	0.873863	-1.363731

$[Mo_2O_8]^{2-}$ II 1/2

E = -20091.50

Mo	-1.463771	-0.000008	-0.000107
Mo	1.610638	-0.000054	-0.000025
O	0.422939	0.123455	1.358707
O	2.630666	-1.390534	0.144281
O	-0.927276	-1.831124	-0.322263
O	-0.925735	1.830974	0.322061
O	-2.510305	-0.433436	1.284248
O	2.629147	1.391401	-0.145435
O	-2.511743	0.434169	-1.283063
O	0.421259	-0.124583	-1.357845

$[Mo_2O_8]^{2-}$ II 3/4

E = -20092.14

Mo	1.797370	-0.223149	-0.006935
Mo	-1.998299	-0.008186	0.003135
O	-2.608036	-0.319105	1.589807
O	-2.532905	1.552930	-0.506056
O	1.662760	2.318715	-0.053219
O	2.141938	-1.839957	0.454575
O	2.647493	0.788768	1.180065
O	-2.624658	-1.196906	-1.083040
O	2.458375	-0.012259	-1.581314
O	-0.090090	-0.077676	0.019133

$[Mo_2O_8]^{2-}$ III 1/2

E = -20091.83

Mo	1.565641	0.097191	-0.010741
Mo	-1.565748	-0.097323	0.010783
O	-0.170699	-0.845588	-0.900609
O	-2.875427	-0.153855	-1.121043
O	-2.031977	-1.084481	1.330829
O	2.028655	1.084422	-1.331848
O	2.020643	-1.777710	-0.288782
O	-2.018604	1.777956	0.289762
O	2.876917	0.155542	1.119214
O	0.171056	0.844406	0.902258

$[Mo_2O_8]^{2-}$ III 3/4

E = -20091.83

Mo	1.565852	0.097365	-0.010500
Mo	-1.565838	-0.097418	0.010469
O	-0.172674	-0.839754	-0.906902
O	-2.879008	-0.149704	-1.117500
O	-2.028434	-1.090514	1.327354
O	2.027048	1.090109	-1.328142
O	2.019611	-1.777504	-0.293986
O	-2.018899	1.777564	0.295150
O	2.879538	0.150939	1.116854
O	0.172747	0.839143	0.907337

$[Mo_3O_9]^{-}$ I 1/2

E = -24003.41

Mo	-2.177189	-0.000242	-0.000007
O	-0.723082	1.355504	0.005406
O	-0.722705	-1.355600	-0.005678
O	-3.095329	-0.005154	1.427850
O	-3.095541	0.004489	-1.427727
Mo	1.061471	1.710283	0.000439
O	1.504184	2.584546	-1.377058
O	1.511203	2.587565	1.373729
O	1.887210	0.000220	-0.000015
Mo	1.061899	-1.710061	-0.000448
O	1.504617	-2.584104	1.377187
O	1.511995	-2.587360	-1.373610

$[Mo_3O_9]^{-}$ I 3/4

E = -24000.32

Mo	-1.008335	-1.735187	-0.070090
O	-1.871596	-0.043261	-0.158423
O	0.752832	-1.338984	0.029132
O	-1.525209	-2.602161	1.287412
O	-1.345898	-2.649533	-1.453790
Mo	-1.109209	1.681029	0.035140
O	-1.768799	2.775804	-1.071251
O	-1.381294	2.255857	1.603524
O	0.671924	1.450014	-0.260030
Mo	2.168486	0.139148	-0.056547
O	2.772424	-0.718022	1.626210
O	3.428168	0.424088	-1.122428

$[Mo_3O_9]^{-}$ II 1/2

E = -24003.25

Mo	-0.000190	-0.717041	-0.000097
Mo	-2.653342	0.356195	0.000068
O	-1.392994	-0.064910	1.295087
O	-1.391328	-0.061744	-1.294799
O	-0.000705	-2.372463	0.000063
O	-3.141550	1.981060	0.001886
O	-4.016150	-0.655270	-0.002132
O	1.394069	-0.066357	-1.295368
O	1.390884	-0.061513	1.294473
Mo	2.653467	0.355998	-0.000019
O	4.017076	-0.654381	0.003689
O	3.141040	1.981032	-0.002645

$[Mo_3O_9]^{-}$ II 3/4

E = -24000.03

Mo	0.046892	-0.791064	0.000013
Mo	-2.712513	0.360721	-0.000032
O	-1.376895	-0.135663	1.219697
O	-1.377020	-0.135924	-1.219746
O	0.060728	-2.453560	-0.000063
O	-3.106124	2.254058	-0.000085
O	-4.025043	-0.697423	0.000226
O	1.410838	-0.067115	-1.303071
O	1.411248	-0.068128	1.303186
Mo	2.644500	0.396103	0.000015
O	4.049649	-0.560083	-0.000584
O	3.063507	2.043592	0.000467

$[Mo_2O_6]$ I 1/2

E = -15998.84

Mo	-1.427378	-0.000078	-0.000019
Mo	1.427358	-0.000179	0.000020
O	0.000024	-0.000667	-1.267467
O	-0.000101	-0.000882	1.267468
O	-2.390709	-1.365709	-0.000036
O	-2.390572	1.365672	0.000061
O	2.388339	1.367054	0.000036
O	2.393125	-1.364120	-0.000067

$[Mo_2O_6]$ I 3/4

E = -15996.15

Mo	-1.405826	-0.020153	0.000008
Mo	1.409213	0.035813	-0.000026
O	0.008624	-0.038699	-1.298717
O	0.008697	-0.038881	1.298689
O	-2.379630	-1.384636	-0.000070
O	-2.357270	1.358676	0.000100
O	2.165887	1.511399	0.000051
O	2.535911	-1.490074	0.000046

$[Mo_2O_5]^{-}$ I 1/2

E = -13945.94

Mo	-1.481406	-0.010140	-0.017307
Mo	1.320206	-0.106121	0.019351
O	-0.170400	-1.052032	0.909355
O	-0.139065	1.126857	-0.701747

O	-3.528312	0.046479	-0.081230
O	2.472850	-1.082943	-0.744500
O	2.211224	1.572007	0.607391

$[Mo_2O_5]^-$ I 3/4
E = -13952.89

Mo	-1.533644	-0.000268	0.000000
Mo	1.299864	-0.000032	0.000000
O	-0.040226	-0.000531	1.308465
O	-0.040226	-0.000525	-1.308465
O	-3.224182	0.000618	0.000000
O	2.267603	-1.408802	-0.000003
O	2.264376	1.410813	0.000003

$[Mo_2O_5]^-$ I 5/6
E = -13952.67

Mo	-1.553835	-0.133373	-0.000001
Mo	1.297460	0.033551	0.000005
O	-0.014826	-0.140871	1.322215
O	-0.014859	-0.140964	-1.322240
O	-3.139311	0.486240	0.000022
O	2.442254	-1.236202	0.000040
O	2.072708	1.555861	-0.000057

$[Mo_2O_5]^-$ I 7/8
E = -13949.43

Mo	1.514076	0.000100	-0.000008
Mo	-1.313341	0.000199	0.000003
O	-0.023253	0.001627	1.338634
O	-0.023522	0.001806	-1.338702
O	3.565464	0.000177	-0.000010
O	-2.290468	1.404675	0.000138
O	-2.282077	-1.409853	-0.000032

$[Mo_2O_6]^-$ I 1/2
E = -16002.22

Mo	1.415536	-0.000083	0.000102
Mo	-1.394983	-0.000031	-0.000090
O	-0.046627	0.000253	-1.329771
O	-0.047423	0.000473	1.330185
O	2.349784	1.422867	-0.000138
O	2.349771	-1.423025	-0.000172
O	-2.357010	-1.404815	0.000039
O	-2.356401	1.404848	-0.000204

$[Mo_2O_6]^-$ I 3/4
E = -15998.70

Mo	1.488918	0.041515	-0.000181
Mo	-1.522994	-0.014010	0.000121
O	0.072655	0.034324	-1.224141
O	0.072959	0.038548	1.224066
O	2.357801	1.495719	-0.001714
O	2.523320	-1.603427	0.002263
O	-2.359156	-1.506861	-0.000447
O	-2.488682	1.397290	0.000286

$[Mo_2O_7]^-$ I 1/2
E = -18039.05

O	-2.849866	-1.564737	0.207394
O	-2.636218	1.467254	0.185232
Mo	-1.758923	0.064906	-0.131662
O	-0.163211	-1.033315	0.636029
O	2.900366	0.305545	-1.247106
O	2.679260	-1.395446	0.163832
Mo	1.456188	0.068753	0.118678
O	-0.058256	0.249024	-1.141798
O	1.717284	1.269966	1.264586

$[Mo_2O_7]^-$ I 3/4
E = -18047.84

O	-2.586668	-1.314154	0.275152
O	-2.483715	1.378045	-0.449483
Mo	-1.560082	0.011084	-0.024954
O	-0.375475	0.322632	1.282649
O	2.595335	0.396424	-1.184553
O	1.905155	-1.537568	0.598108
Mo	1.411052	-0.065032	-0.052945
O	-0.130661	-0.346266	-1.211315
O	1.858431	1.384114	1.098415

$[Mo_2O_7]^-$ I 5/6
E = -18044.93

O	-2.767205	-0.202869	-1.220421
O	-1.638346	1.643890	0.469316
Mo	-1.536756	0.047280	-0.066791
O	-2.278480	-1.145405	1.220020
O	2.283434	1.566030	0.327915
O	2.993379	-1.127759	-0.369954
Mo	1.752794	-0.015008	-0.017969
O	0.064946	-0.206110	-1.068255
O	0.208075	-0.697210	1.086372

$[Mo_2O_7]^-$ I 7/8
E = -18042.20

O	-3.063686	-1.075195	-0.112347
O	-2.212691	1.664285	0.096341
Mo	-1.765461	0.024003	-0.009655
O	-0.170098	-0.549853	1.070716
O	2.367009	1.212803	-1.126181
O	2.587127	-1.451353	0.098754
Mo	1.503545	-0.172587	0.017271
O	-0.133353	-0.376927	-1.110566
O	2.000752	1.356305	1.043298

$[Mo_2O_7]^-$ II 1/2
E = -18047.66

O	-2.473475	0.803870	1.388414
O	2.474027	-0.878990	-1.368101
Mo	-1.875609	0.034186	0.000511
O	2.459647	1.614867	-0.075147
O	-2.653693	-1.697115	-0.009774
Mo	1.921500	-0.009388	0.000422
O	-2.458582	0.817554	-1.385701
O	2.489104	-0.752956	1.435266
O	-0.077953	-0.037419	0.010148

$[Mo_2O_7]^-$ II 3/4
E = -18044.63

O	-2.558328	-0.680101	-1.458881
O	2.416493	-0.825963	1.412704
Mo	-1.916005	-0.066788	-0.000065
O	2.416530	-0.831437	-1.409370
O	-2.160067	1.895472	-0.000654
Mo	1.860185	-0.055958	0.000180
O	-2.558947	-0.678531	1.459165
O	2.677641	1.677854	-0.003485
O	0.059731	0.087121	-0.000084

$[Mo_2O_8]^-$ I 1/2
E = -20093.23

Mo	1.656100	0.009832	-0.026165
Mo	-1.344173	-0.274381	-0.073738
O	-1.396780	1.965672	-0.047149
O	0.205559	-0.603525	-1.130462
O	-2.599795	-0.371417	-1.192704
O	-1.566202	-1.575358	0.974759
O	2.488056	1.352713	-0.671558
O	2.763153	-1.208303	0.422579
O	-2.064628	1.380819	0.905964
O	0.533022	0.448283	1.263060

$[Mo_2O_8]^-$ I 3/4
E = -20090.33

Mo	1.793739	0.020275	-0.038384
Mo	-1.406116	-0.275372	-0.096039
O	-1.529167	1.938200	-0.033815
O	0.151765	-0.416954	-1.085587
O	-2.661554	-0.521591	-1.197367
O	-1.447617	-1.566330	1.004135
O	2.631192	1.391879	-0.610621
O	2.740324	-1.323221	0.416362
O	-2.265305	1.349499	0.868031
O	0.345339	0.487776	1.344586

$[Mo_2O_8]^-$ II 1/2
E = -20090.78

Mo	1.671030	-0.060891	0.015161
Mo	-1.670853	0.060492	0.015234
O	-0.054618	1.054829	-0.446896
O	-2.489916	-1.545994	-0.612982
O	0.054871	-1.051578	-0.449971
O	1.849453	-0.135066	1.688550
O	2.490253	1.546070	-0.612276
O	-2.857655	1.053151	-0.707935
O	2.858664	-1.053482	-0.706495
O	-1.851979	0.134167	1.688432

$[Mo_2O_8]^-$ II 3/4
E = -20090.85

Mo	1.664069	0.067704	-0.000109
Mo	-1.668383	-0.057475	0.036424
O	-0.150342	-1.261374	-0.238596
O	-2.660648	-0.682040	1.247281
O	0.130551	0.519353	0.990555
O	1.625139	0.807016	-1.517243
O	2.383257	-1.693676	-0.100995
O	-2.453227	-0.308075	-1.458701
O	2.967948	0.724878	0.880483
O	-1.820031	1.840213	0.006562

$[Mo_2O_8]^-$ III 1/2
E = -20090.74

Mo	1.440593	0.191922	-0.010751
Mo	-1.574471	-0.128961	0.009233
O	0.152981	1.066946	-0.863651
O	-2.751912	-0.518817	1.173833
O	-1.984849	-0.962399	-1.397694
O	2.229920	1.242960	1.056123
O	2.567646	-0.420265	-1.244291
O	-2.128702	1.694251	-0.050384
O	2.589098	-1.575899	0.562464
O	0.028675	-0.857322	0.771571

$[Mo_2O_8]^-$ III 3/4
E = -20090.74

Mo	1.572536	-0.129100	-0.008609
Mo	-1.441065	0.193639	0.011565
O	-0.147496	1.069408	0.855630
O	-2.589403	-1.576456	-0.562447
O	-2.233903	1.243807	-1.053699
O	1.982139	-0.969569	1.394296
O	2.149083	1.687565	0.055367
O	-2.565029	-0.419179	1.246466
O	2.746327	-0.517942	-1.178169
O	-0.031943	-0.856464	-0.772961

$[Mo_4O_{13}]^{2-}$ I 1/2
E = -34052.47

Mo	0.548335	2.187139	-0.454668
O	-2.028936	0.097373	2.088828
O	0.798249	3.858655	-0.292065
O	0.795269	1.835846	-2.092998
O	1.889223	1.469566	0.581981
Mo	-2.250362	0.485736	0.454110
Mo	2.092451	-0.529577	0.541300
O	3.495069	-0.508327	-0.416358

O	2.524987	-0.782301	2.161098
O	-1.301433	2.062572	-0.005226
O	0.138732	-0.186853	0.003112
O	-3.866889	0.982335	0.298696
Mo	-0.431676	-2.081895	-0.538564
O	-2.141035	-1.011310	-0.600315
O	-0.390859	-2.580430	-2.158193
O	1.397646	-2.235646	0.007984
O	-1.093448	-3.323842	0.412019

$[Mo_4O_{13}]^{2-}$ I 3/4
E = -34049.95

Mo	-0.117967	-1.955884	-0.445680
O	2.709972	0.195996	1.992704
O	0.087517	-3.390517	0.891641
O	-0.180818	-2.503213	-2.029101
O	-1.931462	-1.851462	0.212789
Mo	2.688698	-0.069143	0.311534
Mo	-2.468356	-0.007710	0.382491
O	-3.817775	0.010324	-0.648741
O	-3.008213	0.037913	1.990618
O	1.830442	-1.645712	-0.058279
O	-0.419754	-0.004779	-0.188354
O	4.297390	-0.141474	-0.250929
Mo	-0.051402	2.057573	-0.340416
O	1.796269	1.293085	-0.527663
O	-0.161539	2.804655	-1.854967
O	-1.867908	1.834834	0.157572
O	0.398270	3.229961	0.796085

$[Mo_4O_{13}]^{2-}$ II 1/2
E = -34052.05

Mo	-4.964745	0.329593	-0.228081
O	-5.358886	1.568268	0.890450
O	-5.938591	-1.050771	0.070730
O	-5.231825	0.889728	-1.826649
Mo	-1.520183	-1.104186	0.229189
Mo	1.520230	1.104256	0.229102
O	1.534250	1.831261	1.751425
O	1.411468	2.339537	-0.914232
O	3.050149	0.159477	-0.018013
O	-0.000092	0.000194	0.079438
O	-1.412290	-2.338230	-0.915559
O	-1.532697	-1.832803	1.750753
O	-3.050492	-0.159336	-0.015245
Mo	4.964659	-0.329642	-0.228163
O	5.357398	-1.568348	0.890841
O	5.233542	-0.889735	-1.826444
O	5.938270	1.050649	0.071751

$[Mo_4O_{13}]^{2-}$ III 1/2
E = -34051.87

Mo	0.560268	-1.108316	0.006417
Mo	4.133464	0.106166	-0.003518
Mo	-1.564098	1.990786	0.001376
Mo	-3.337880	-0.910378	-0.004674
O	-3.163487	1.001933	-0.007520
O	-1.506671	2.977520	1.377826
O	2.229956	-0.359795	0.006021
O	5.071456	-1.321510	0.188734
O	-4.177594	-1.441248	1.380537
O	-1.662343	-1.444828	0.001976
O	-0.229988	0.770578	0.001699
O	4.538648	0.856414	-1.495613
O	4.470454	1.186907	1.289338
O	-1.497197	2.987060	-1.367831
O	-4.168349	-1.445793	-1.393699
O	0.592004	-2.083625	1.380579
O	0.596403	-2.094466	-1.359953

$[Mo_4O_{13}]^{2-}$ III 3/4
E = -34049.17

Mo	0.424002	-1.166346	0.013551
Mo	4.078826	0.049915	-0.017330
Mo	-1.510288	2.011773	-0.003750
Mo	-3.305321	-0.894526	-0.002915
O	-3.126723	1.012357	-0.010392
O	-1.450376	2.994141	1.386023
O	2.369594	-0.395090	0.014462
O	4.958259	-0.641721	1.282753
O	-4.162021	-1.393659	1.385486
O	-1.630926	-1.503376	0.008452
O	-0.193984	0.787443	0.001357
O	4.802330	-0.347565	-1.511140
O	4.317197	1.932285	0.250074
O	-1.437706	2.994607	-1.392810
O	-4.148178	-1.405444	-1.395475
O	0.667635	-2.012460	1.474472
O	0.676995	-2.025801	-1.438426

$[Mo_4O_{13}]^{2-}$ IV 1/2
E = -34051.87

Mo	-3.336056	-0.916169	-0.016794
O	-3.167534	0.996565	-0.038200
O	-1.659015	-1.444318	0.024851
O	-4.196901	-1.436083	1.359625
O	-4.141261	-1.468553	-1.413978
Mo	-1.571827	1.990487	0.003871
O	-1.500066	3.018846	-1.341310
O	-1.526299	2.944753	1.403412
O	-0.233587	0.775060	-0.016119
Mo	0.562554	-1.101183	0.022311
O	0.597828	-2.053920	1.411996
O	0.601727	-2.109355	-1.327803
O	5.054331	-1.268034	0.461189
O	4.601197	0.581073	-1.597619
Mo	4.136337	0.105813	-0.012733
O	2.229795	-0.347012	0.010343
O	4.436989	1.396499	1.081175

$[Mo_4O_{13}]^{2-}$ IV 3/4
E = -34047.99

Mo	2.468502	1.852851	-0.185734
O	3.137558	0.026943	-0.050297
O	0.695857	1.317110	0.366159
O	3.174038	2.911680	0.957236
O	2.494473	2.426717	-1.794204
Mo	2.573574	-1.718305	-0.208275
O	2.745616	-2.288085	-1.806050
O	3.391595	-2.736826	0.888690
O	0.782934	-1.526156	0.243460
Mo	-0.451822	-0.129870	0.778368
O	-0.637590	-0.264320	2.440189
O	-1.925729	-0.093042	-0.340595
O	-3.903400	0.518658	1.408854
O	-4.147802	1.068528	-1.744046
Mo	-3.886786	-0.034436	-0.471090
O	-4.281962	-1.625819	-0.940475
O	-5.218790	0.420846	0.826416

$[Mo_4O_{13}]^{2-}$ V 1/2
E = -34051.69

Mo	-2.513686	-1.205854	-0.553027
Mo	-1.524366	1.991910	0.168574
Mo	0.772332	-0.948988	0.831245
O	-2.804934	0.627339	-0.317312
O	-0.731846	-1.476227	-0.426861
O	0.020075	1.160339	0.135518
O	-1.568615	3.272900	-0.957932
O	-1.881761	2.612703	1.717349
O	-3.362792	-2.083144	0.627632
O	-3.082908	-1.701382	-2.075462
O	2.363110	0.076220	1.205857
O	1.906141	-0.650270	-1.293263
Mo	3.215814	0.210633	-0.488257
O	3.464042	1.803678	-1.064498
O	4.701270	-0.647494	-0.548576
O	-0.145334	-0.736226	2.229562
O	1.385557	-2.508866	0.985680

$[Mo_4O_{13}]^{2-}$ V 3/4
E = -34049.27

Mo	-2.229732	-1.452858	-0.593294
Mo	-1.777182	1.848619	0.058799
Mo	0.850722	-0.509080	1.185286
O	-2.702440	0.385605	-0.734022
O	-0.838563	-1.470551	0.532199
O	-0.204005	1.209347	0.610402
O	-1.588479	3.102212	-1.074107
O	-2.679197	2.446101	1.373744
O	-3.531074	-2.342033	0.051948
O	-1.856181	-2.078726	-2.126837
O	2.511982	0.625575	1.051296
O	1.528648	-0.731188	-0.963271
Mo	3.019103	0.238479	-0.691200
O	3.132514	1.624777	-1.690250
O	4.475606	-0.659269	-0.820979
O	0.586609	-0.591730	2.834076
O	1.884294	-2.177214	1.167949

$[Mo_4O_{13}]^{2-}$ VI 1/2
E = -34051.51

Mo	2.874829	-0.404634	0.065982
O	3.937898	-0.645084	-1.229805
O	3.754236	-0.651690	1.491220
O	2.230202	1.300205	0.027325
Mo	-0.268904	-2.407236	-0.065920
Mo	0.629944	2.451845	-0.057993
O	0.694667	3.367788	-1.499076
O	0.641707	3.508659	1.284427
O	-0.753169	1.413230	-0.035002
O	-0.427816	-3.321866	-1.486413
O	-1.493113	-1.133960	0.005743
O	-0.412002	-3.457987	1.258843
O	1.480466	-1.618980	-0.035293
Mo	-3.016246	0.296447	0.054978
O	-4.249595	-0.897237	0.095374
O	-3.225157	1.228979	1.477161
O	-3.331344	1.241722	-1.339007

$[Mo_4O_{13}]^{2-}$ VI 3/4
E = -34049.57

Mo	-3.029595	-0.030545	0.069637
O	-4.006738	-0.005457	-1.331070
O	-3.899513	0.099034	1.533641
O	-1.767443	-1.531503	0.094245
Mo	-0.100783	2.373864	-0.085921
Mo	-0.114806	-2.330861	-0.052116
O	0.081233	-2.929139	-1.631625
O	-0.042355	-3.641639	1.035590
O	1.186522	-1.120903	0.343412
O	-0.191282	3.310587	-1.502910
O	1.510144	1.472365	-0.055914
O	-0.187347	3.416515	1.254861
O	-1.488167	1.195312	-0.046101
Mo	2.855623	0.068544	0.108131
O	4.011757	0.763789	-0.945746
O	3.499488	0.179931	1.664720
O	3.338896	-1.634153	-0.621692

$[Mo_4O_{13}]^{2-}$ VII 1/2
E = -34049.60

Mo	-2.032391	1.797305	-0.261494
O	2.199844	-1.256872	0.228303
O	-1.956551	-2.497647	-1.617157
O	-2.726694	2.116944	1.264251
O	-2.604320	0.218676	-0.890256
Mo	3.572823	-0.188387	-0.360739
Mo	-2.126620	-1.757593	-0.105229
O	-3.961505	-1.385425	0.309626
O	-2.217031	-3.034094	1.032032
O	-2.452104	3.054362	-1.340182
O	2.515806	1.300874	-0.190411

O	4.064662	-0.505175	-1.966384
Mo	0.937727	0.302701	0.696595
O	4.938691	-0.185909	0.668997
O	-0.173862	1.701292	-0.085544
O	-0.516748	-0.920690	0.419340
O	1.044230	0.585032	2.329435

$[Mo_4O_{13}]^{2-}$ VII 3/4
E = -34048.66

Mo	-0.293022	1.960442	-0.265660
O	-0.703328	-1.861756	0.332730
O	1.094702	0.663559	1.135770
O	-1.493145	2.758342	-1.156994
O	0.719124	3.238795	0.168800
Mo	-2.072134	-0.535069	0.798009
Mo	2.479608	-0.321338	0.625312
O	3.750171	0.645172	-0.001085
O	3.104229	-1.242141	1.929899
O	-1.520741	1.202355	1.107306
O	-1.880068	-0.368179	-1.050959
O	-1.723058	-1.420721	2.480731
Mo	-0.047693	-1.134567	-1.340373
O	-3.748822	-0.740115	0.963702
O	0.458879	0.800927	-1.510667
O	1.783193	-1.439813	-0.718561
O	-0.191621	-2.076135	-2.721436

$[Mo_4O_{13}]^{2-}$ VIII 1/2
E = -34049.22

Mo	3.753904	0.875291	-0.123721
Mo	1.301513	-0.980090	0.172026
Mo	-1.672013	-0.901432	-0.160516
Mo	-3.844993	0.955599	0.069588
O	2.363345	-0.153804	1.332606
O	2.456537	0.018977	-1.140967
O	-2.860578	-0.025083	1.276700
O	-2.742734	0.443173	-1.306799
O	-0.085271	-0.482465	-1.130016
O	-0.261235	-1.128681	1.199000
O	1.828517	-2.568372	-0.048546
O	-2.221784	-2.426189	-0.560491
O	-5.456465	0.406287	-0.103030
O	4.850902	0.082378	1.243622
O	3.286465	2.460271	0.256778
O	-3.840477	2.637528	0.373185
O	5.106121	1.001796	-1.168270

$[Mo_4O_{13}]^{2-}$ VIII 3/4
E = -34049.21

Mo	-3.751813	-0.875806	-0.122758
Mo	-1.301544	0.981743	0.170323
Mo	1.670816	0.900676	-0.158953
Mo	3.844559	-0.955709	0.069089
O	-2.373306	0.164419	1.329882
O	-2.453350	-0.020131	-1.140989
O	2.858394	0.020669	1.277825
O	2.744010	-0.439347	-1.307483
O	0.085777	0.487693	-1.133222
O	0.258356	1.118926	1.202376
O	-1.819805	2.572275	-0.053073
O	2.221042	2.427007	-0.551867
O	5.456150	-0.405374	-0.099533
O	-4.858833	-0.090941	1.242128
O	-3.281858	-2.459739	0.258453
O	3.839712	-2.638596	0.367520
O	-5.101883	-1.004108	-1.169953

$[Mo_2O_5]$ I 1/2
E = -13950.02

Mo	-1.472057	0.230405	0.000014
Mo	1.211748	-0.060468	0.000103
O	-0.154693	0.329096	-1.330291
O	-0.154759	0.329448	1.330431
O	-2.586237	-0.995293	-0.000297
O	2.456020	1.071826	-0.000573
O	1.806289	-1.627249	0.000111

$[Mo_2O_5]$ I 3/4
E = -13950.04

Mo	-1.547814	0.058950	0.000098
Mo	1.318210	-0.014422	-0.000086
O	-0.080465	0.054261	-1.278437
O	-0.079886	0.053626	1.278517
O	-3.189747	-0.214065	-0.000171
O	2.348765	1.311433	0.000283
O	2.206754	-1.439024	-0.000252

$[MoO_3]$ I 1/2
E = -7997.08

Mo	-0.000014	-0.000030	-0.200076
O	1.209138	-1.049738	0.350144
O	0.304732	1.571934	0.350095
O	-1.513795	-0.522039	0.350162

$[MoO_3]$ I 3/4
E = -7995.28

Mo	-0.021221	0.000089	-0.133236
O	-0.864834	-1.404503	0.240605
O	1.845913	0.002356	0.218118
O	-0.869670	1.401681	0.240767

$[Mo_3O_{10}]^{-}$ I 1/2
E = -26048.74

Mo	-0.914745	-1.600972	-0.016999
O	0.968439	-1.614004	0.398588
O	-2.146488	0.000736	-0.261436
O	-1.323628	-2.854354	-1.060113
O	-1.557554	-1.958090	1.505659
Mo	1.907188	-0.000695	0.036237
O	2.640898	-0.001027	-1.477801
O	-0.258585	0.000167	-1.109186
Mo	-0.913484	1.601653	-0.016998
O	0.969708	1.613396	0.398500
O	-1.556015	1.959013	1.505713
O	3.170220	-0.001160	1.148304
O	-1.321530	2.855400	-1.059983

$[Mo_3O_{10}]^{-}$ I 3/4
E = -26045.95

Mo	-1.727279	-0.774370	-0.016234
O	-0.079490	-2.022395	-0.183402
O	-1.742970	1.055360	0.365649
O	-2.886302	-1.152340	-1.176384
O	-2.203086	-1.503794	1.433982
Mo	1.652777	-0.897668	-0.037623
O	2.767533	-1.369046	-1.205995
O	-0.011963	-0.061417	-1.018323
Mo	0.064821	1.750292	-0.072371
O	1.823173	0.933818	0.335534
O	0.237994	2.303553	1.805705
O	2.083310	-1.652692	1.412987
O	0.062628	3.058117	-1.107058

$[Mo_3O_{10}]^{-}$ II 1/2
E = -26048.17

Mo	2.052023	-0.001153	-0.200152
O	0.589182	1.373524	-0.144872
O	0.587852	-1.374254	-0.140930
O	2.759468	-0.003435	-1.708668
O	3.152182	0.718482	1.179307
Mo	-1.186087	1.713126	0.022693
O	-1.521933	2.502796	1.478520
O	-1.747722	2.669554	-1.252231
O	-2.003292	0.001030	-0.005926
Mo	-1.188087	-1.712002	0.023090
O	-1.748497	-2.666796	-1.253595
O	-1.527443	-2.502452	1.477688
O	3.151492	-0.718299	1.181149

$[Mo_3O_{10}]^{-}$ II 3/4
E = -26045.98

Mo	1.908925	-0.284265	-0.271788
O	0.997978	1.395678	-0.085826
O	0.475638	-1.453482	-0.061363
O	2.541293	-0.388801	-1.808082
O	3.184949	0.062696	1.352627
Mo	-0.809227	1.912320	0.025746
O	-1.170975	2.411321	1.606679
O	-1.180034	3.141992	-1.081404
O	-1.638863	0.340120	-0.399124
Mo	-1.498506	-1.577885	0.015800
O	-2.136322	-2.573476	-1.207211
O	-1.996557	-1.972611	1.594634
O	3.016637	-1.226833	1.297844

$[Mo_3O_{10}]^{-}$ III 1/2
E = -26048.06

Mo	3.237843	-0.648233	-0.008339
O	1.488145	0.340618	-0.063158
O	-3.482111	-1.338730	-1.524275
O	3.180505	-1.897472	-1.177474
O	3.441956	-1.315116	1.554645
Mo	0.006304	1.344876	0.012793
O	-0.010480	2.281557	1.411362
O	-0.073461	2.392417	-1.302652
O	-1.558075	0.214306	0.005999
Mo	-3.194248	-0.618816	-0.027643
O	-4.438739	0.466419	0.315439
O	-3.322328	-1.991115	1.261416
O	4.512618	0.438527	-0.359563

$[Mo_3O_{10}]^{-}$ III 3/4
E = -26045.52

Mo	-2.766632	-0.813238	0.000022
O	-1.435276	0.420100	0.000107
O	2.735146	-1.771528	1.399079
O	-2.736219	-1.770723	1.399672
O	-2.735292	-1.771639	-1.398990
Mo	0.000022	1.766528	-0.000008
O	-0.000083	2.691343	-1.422379
O	0.000255	2.690775	1.422718
O	1.435181	0.419987	-0.000386
Mo	2.766603	-0.813290	-0.000019
O	4.461056	0.063869	0.000690
O	2.736423	-1.770927	-1.399577
O	-4.461157	0.063749	-0.000908

$[Mo_3O_{10}]^{-}$ IV 1/2
E = -26047.34

Mo	1.901973	-0.962442	0.025924
O	1.311155	0.726551	0.301238
O	3.037695	-1.003383	-1.222062
O	2.600516	-1.604890	1.422249
Mo	0.004039	2.212635	-0.000053
O	-0.282495	3.130400	1.398496
O	0.293487	3.130486	-1.397972
O	-1.307475	0.730567	-0.301600
Mo	-1.905489	-0.955817	-0.025992
O	-2.606506	-1.595942	-1.422160
O	-3.041459	-0.991722	1.221925
O	-0.405233	-2.033206	0.597663
O	0.397568	-2.034338	-0.597141

$[Mo_3O_{10}]^{-}$ IV 3/4
E = -26044.87

Mo	2.307896	-0.808357	0.031902
O	1.230287	0.650838	0.751333
O	2.903593	-0.511724	-1.528100
O	3.453771	-1.360289	1.155339

Mo 0.005491 1.818136 -0.000107
O -0.683364 2.791819 1.199820
O 0.691269 2.791614 -1.202097
O -1.215041 0.644464 -0.747774
Mo -2.312011 -0.802899 -0.034555
O -3.426271 -1.378136 -1.177783
O -2.951258 -0.474840 1.501791
O -0.642889 -2.116682 0.144040
O 0.632679 -2.123183 -0.082080

$[Mo_2O_7]$ I 1/2
E = -18043.17
O -2.669010 -1.124350 -0.480787
O -2.223596 1.490348 0.049079
Mo -1.497762 -0.006780 -0.084398
O -0.499411 -0.518666 1.505928
O 2.666688 1.128272 -0.476530
O 2.227328 -1.488962 0.044451
Mo 1.497855 0.006801 -0.084348
O 0.000140 0.000821 -1.263259
O 0.497374 0.512426 1.507031

$[Mo_2O_7]$ II 1/2
E = -18042.11
O -2.456228 0.135405 -1.588796
O 2.456238 0.139573 1.588390
Mo -1.872707 -0.010343 -0.023138
O 2.354590 -1.487545 -0.605689
O -2.703468 1.331184 0.996598
Mo 1.872642 -0.010289 0.023137
O -2.354403 -1.486022 0.609537
O 2.703629 1.328549 -0.999897
O -0.000015 0.147178 -0.000135

$[Mo_2O_7]$ II 3/4
E = -18042.11
O -2.456501 0.136994 -1.588455
O 2.456502 0.137026 1.588452
Mo -1.872634 -0.010344 -0.023082
O 2.353858 -1.486877 -0.607822
O -2.703494 1.330144 0.998034
Mo 1.872634 -0.010343 0.023083
O -2.353857 -1.486866 0.607850
O 2.703491 1.330123 -0.998066
O -0.000000 0.148058 0.000001

$[Mo_2O_7]$ III 1/2
E = -18041.56
O -2.511225 1.370360 0.083325
O -0.004150 -0.000267 1.268111
Mo -1.548249 0.000002 0.045581
O 2.835543 0.000493 0.946782
O -2.508770 -1.372072 0.081869
Mo 1.400242 0.000149 0.074749
O 1.626016 -1.568844 -0.863903
O 1.624218 1.568639 -0.864580
O -0.284599 0.000901 -1.283336

$[Mo_2O_7]$ III 3/4
E = -18041.61
O -2.508512 1.368437 0.100947
O 0.002763 -0.000467 1.262577
Mo -1.543940 -0.000060 0.050474
O 2.855435 0.003046 0.944545
O -2.503224 -1.372241 0.099759
Mo 1.416369 0.000501 0.076182
O 1.569744 -1.550798 -0.879543
O 1.561462 1.549359 -0.882468
O -0.307921 0.000345 -1.310757

$[Mo_3O_{10}]^{2-}$ I 1/2
E = -26051.01
Mo 3.395824 -0.532156 0.000214
O 1.513076 0.020822 0.006220
O -3.673299 -1.624529 -1.297496
O 3.762258 -1.308406 -1.488570
O 3.674008 -1.625208 1.296871
Mo 0.000130 1.063730 0.000029
O -0.004802 2.068670 1.364597
O 0.005719 2.067638 -1.365290
O -1.513541 0.021894 -0.005291
Mo -3.395987 -0.532046 -0.000179
O -4.406758 0.845291 -0.187910
O -3.762419 -1.309289 1.488092
O 4.405933 0.845595 0.188442

$[Mo_3O_{10}]^{2-}$ I 3/4
E = -26047.84
Mo 3.388404 -0.523353 -0.000022
O 1.527669 0.064720 0.000058
O -4.011890 -1.307750 -1.436127
O 3.689803 -1.471997 -1.402822
O 3.690086 -1.471531 1.403029
Mo -0.047496 1.028795 0.000025
O -0.101416 2.023478 1.372476
O -0.101314 2.023586 -1.372348
O -1.460926 -0.151488 -0.000090
Mo -3.377627 -0.621348 -0.000015
O -3.462683 1.365154 -0.000081
O -4.011241 -1.307798 1.436317
O 4.434691 0.842130 -0.000347

$[Mo_3O_{10}]^{2-}$ II 1/2
E = -26050.16
Mo -1.708699 -0.630766 -0.014416
O -0.340524 -1.983693 0.276352
O -1.547922 1.286755 0.276291
O -2.756083 -1.018309 -1.299809
O -2.569239 -0.947960 1.431335
Mo 1.400618 -1.164485 -0.014457
O 2.259595 -1.878894 -1.299493
O 0.000123 0.000195 -0.995898
Mo 0.308009 1.795200 -0.014589
O 1.888341 0.697155 0.275807
O 0.463508 2.698334 1.431549
O 2.105897 -1.749615 1.431713
O 0.496680 2.896299 -1.299677

$[Mo_3O_{10}]^{2-}$ II 3/4
E = -26047.67
Mo -1.621892 -0.866191 -0.014533
O -0.000050 -1.870619 0.329068
O -1.785764 0.999286 0.333181
O -2.483127 -1.377031 -1.391101
O -2.516262 -1.459607 1.316500
Mo 1.621837 -0.866280 -0.014556
O 2.483026 -1.377153 -1.391140
O -0.000008 0.109649 -1.088024
Mo 0.000042 1.759474 -0.084345
O 1.785809 0.999194 0.333170
O 0.000213 2.138510 1.882515
O 2.516200 -1.459747 1.316458
O 0.000037 3.155749 -1.045100

$[Mo_3O_{10}]^{2-}$ III 1/2
E = -26050.16
Mo 1.761235 -0.463985 0.014433
O 1.417886 1.429271 -0.275578
O 0.529038 -1.942085 -0.275230
O 2.646905 -0.698191 -1.432023
O 2.841707 -0.749404 1.299100
Mo -0.478414 1.757152 0.014544
O -0.771707 2.836181 1.298623
O -0.720411 2.639672 -1.432542
O -1.947046 0.513158 -0.273679
Mo -1.282684 -1.293017 0.014546
O -1.927646 -1.942304 -1.432319
O -2.069832 -2.087260 1.298648
O 0.000390 0.000178 0.996504

$[Mo_3O_{10}]^{2-}$ III 3/4
E = -26047.46
Mo 2.143812 0.331899 -0.024720
O 0.483635 1.376240 -0.445638
O 1.179568 -1.399602 0.068381
O 3.219032 0.362708 -1.362101
O 2.865262 0.741696 1.474606
Mo -1.321425 1.373016 -0.025497
O -1.738470 2.436041 1.239658
O -2.050140 2.045792 -1.416874
O -2.191058 -0.347596 -0.044993
Mo -0.669317 -1.685720 0.016706
O -0.874465 -2.028644 -1.647431
O -1.076473 -3.097993 0.879210
O -0.620508 -0.189414 1.431112

$[Mo_3O_{10}]^{2-}$ IV 1/2
E = -26046.61
Mo 1.591213 -0.787720 -0.040468
O 1.757880 1.090271 -0.040228
O 2.133470 -1.231698 -1.597424
O 2.750908 -1.285503 1.125757
Mo -0.014387 1.827045 0.023398
O -0.021292 2.619877 1.543158
O -0.020747 2.880462 -1.329100
O -1.775010 1.063216 -0.041956
Mo -1.578676 -0.811854 -0.040737
O -2.113792 -1.265307 -1.597466
O -2.730678 -1.325958 1.126099
O 0.012349 -1.617717 1.240125
O 0.016623 -2.121866 -0.125482

$[Mo_3O_{10}]^{2-}$ IV 3/4
E = -26048.07
Mo 2.072367 0.333788 0.066194
O 0.434985 1.237147 0.044534
O 3.093802 1.501185 -0.697550
O 2.648583 0.195475 1.655905
Mo -1.460064 1.640076 -0.025826
O -2.020880 2.456059 1.374422
O -1.937751 2.363735 -1.505029
O -1.867675 -0.297317 0.018578
Mo -0.898787 -1.877368 0.004649
O -1.232000 -2.741985 -1.437800
O -1.355448 -2.851906 1.339532
O 0.836550 -1.453703 0.104132
O 2.903875 -0.915296 -1.133059

$[Mo_3O_{10}]^{2-}$ V 1/2
E = -26047.11
Mo 2.091424 0.000254 -0.207764
O 0.542238 1.271766 -0.141522
O 0.542735 -1.272472 -0.142798
O 2.743495 0.000753 -1.763221
O 3.215005 -0.720143 1.205868
Mo -1.202736 1.688543 0.025647
O -1.520624 2.459362 1.525051
O -1.726836 2.648894 -1.295972
O -2.118886 -0.000275 -0.001712
Mo -1.202183 -1.688739 0.025587
O -1.727045 -2.649682 -1.295286
O -1.519007 -2.459186 1.525389
O 3.214775 0.720680 1.205986

$[Mo_3O_{10}]^{2-}$ V 3/4
E = -26046.86
Mo 1.972963 -0.369997 -0.212652
O 0.918368 1.344340 -0.290759
O 0.364580 -1.414880 0.050496

O	2.616320	-0.707940	-1.735022
O	2.980371	-1.168479	1.233378
Mo	-0.772337	1.941575	0.025685
O	-0.839917	2.907085	1.439490
O	-1.341460	2.856207	-1.307867
O	-1.760554	0.424426	0.247063
Mo	-1.567661	-1.556045	0.052214
O	-2.169069	-2.123798	-1.454163
O	-2.101545	-2.442461	1.426236
O	3.259842	0.243953	1.098606

$[Mo_3O_9]$ I 1/2
E = -24000.12

Mo	-1.113825	-1.689854	0.000004
O	-1.714792	0.101605	-0.001069
O	0.769473	-1.535979	0.001294
O	-1.658933	-2.514967	1.342475
O	-1.657058	-2.515962	-1.342617
Mo	-0.906518	1.809498	-0.000106
O	-1.349760	2.694426	-1.342047
O	-1.349654	2.692609	1.343060
O	0.945533	1.434478	-0.000307
Mo	2.020342	-0.119654	0.000077
O	3.008210	-0.177510	1.342192
O	3.006991	-0.178649	-1.342847

$[Mo_3O_9]$ I 3/4
E = -23997.01

Mo	1.041147	1.720791	-0.010858
O	1.829097	0.003095	0.006433
O	-0.806002	1.368063	-0.092887
O	1.430083	2.582643	1.367524
O	1.531786	2.612318	-1.337113
Mo	1.047196	-1.717430	-0.010444
O	1.537703	-2.605550	-1.339079
O	1.442775	-2.579641	1.365836
O	-0.801301	-1.370952	-0.087576
Mo	-2.109226	-0.003436	0.033398
O	-2.804774	-0.002394	1.537697
O	-3.249733	-0.007188	-1.484337

$[Mo_3O_9]$ II 1/2
E = -23998.91

Mo	0.000005	-0.717407	-0.000210
Mo	-2.683236	0.367487	0.000124
O	-1.352141	-0.151558	1.240934
O	-1.353719	-0.153591	-1.241072
O	0.000269	-2.367843	0.000345
O	-3.079803	1.992396	-0.001336
O	-4.081418	-0.549596	0.001399
O	1.352633	-0.152100	-1.241112
O	1.353115	-0.152311	1.240993
Mo	2.683168	0.367607	0.000010
O	4.080651	-0.550572	-0.000159
O	3.080744	1.992318	0.000407

$[Mo_3O_9]$ II 3/4
E = -23996.66

Mo	-0.039353	-0.744872	0.000047
Mo	-2.660449	0.377842	-0.000036
O	-1.348306	-0.097259	1.276111
O	-1.348850	-0.098436	-1.276203
O	-0.027885	-2.387662	0.000443
O	-3.088843	2.001092	-0.000658
O	-4.041931	-0.573941	0.000636
O	1.480604	-0.041200	-1.303902
O	1.480954	-0.041625	1.303823
Mo	2.629998	0.330255	-0.000107
O	3.988480	-0.655144	-0.000617
O	3.272248	2.087246	0.000871

$[Mo_2O_7]^{2-}$ I 1/2
E = -18049.32

Mo	-1.903884	0.000003	-0.000008
Mo	1.903885	0.000007	0.000018
O	-0.000001	0.000111	0.000053
O	2.494708	-1.324121	0.941347
O	2.495081	-0.153305	-1.617264
O	-2.495213	0.152965	1.617259
O	-2.494770	1.324228	-0.941155
O	2.495184	1.477153	0.675984
O	-2.494991	-1.477084	-0.676272

$[Mo_2O_7]^{2-}$ I 3/4
E = -18045.95

Mo	1.952371	-0.053781	-0.002603
Mo	-1.883212	-0.039147	-0.002319
O	0.014182	-0.000534	-0.001959
O	-2.507513	0.682703	1.441148
O	-2.508079	0.803580	-1.378515
O	2.709999	-0.529047	-1.478443
O	1.631418	1.904667	0.098547
O	-2.407001	-1.689654	-0.072184
O	2.703908	-0.683840	1.417249

$[Mo_2O_7]^{2-}$ I 5/6
E = -18042.89

Mo	-1.893703	0.190368	0.021237
Mo	1.923888	-0.016657	0.022001
O	0.036148	-0.054269	-0.013578
O	2.538394	-1.571898	0.474496
O	2.540968	0.410100	-1.541286
O	-2.487003	1.754266	-0.342662
O	-2.103369	-1.631262	-0.941960
O	2.460357	1.161941	1.175802
O	-3.143966	-0.980865	0.962186

$[Mo_2O_7]^{2-}$ I 7/8
E = -18039.50

Mo	-1.857915	0.151102	0.037120
Mo	1.973188	-0.055161	0.044782
O	0.058922	-0.208369	0.038466
O	2.707935	0.150490	1.599085
O	2.833771	-1.120327	-1.014446
O	-2.441795	1.719116	-0.330343
O	-2.090745	-1.684894	-0.910347
O	1.431902	1.650416	-0.812641
O	-3.105172	-1.010124	1.000242

$[MoO_3]^{-}$ I 1/2
E = -8000.05

Mo	0.000043	-0.000034	-0.089409
O	0.055667	-1.716803	0.156484
O	1.459291	0.906752	0.156476
O	-1.515185	0.810228	0.156439

$[MoO_3]^{-}$ I 3/4
E = -7997.15

Mo	0.060233	0.000079	0.000011
O	0.826655	1.557831	-0.000021
O	-1.977425	-0.004488	-0.000016
O	0.834549	-1.553758	-0.000021

$[Mo_2O_6]^{2-}$ I 1/2
E = -16000.16

Mo	-1.320098	-0.000034	0.000005
Mo	1.320138	-0.000009	-0.000006
O	-0.000015	-0.000100	-1.444001
O	0.000010	-0.000042	1.444000
O	-2.232915	-1.481317	0.000010
O	-2.233051	1.481169	-0.000018
O	2.232391	1.481602	-0.000008
O	2.233370	-1.481086	0.000019

$[Mo_2O_6]^{2-}$ I 3/4
E = -15999.71

Mo	-1.526597	-0.000072	-0.000004
Mo	1.526595	-0.000061	0.000004
O	0.000010	-0.000496	-1.224561
O	-0.000018	-0.001195	1.224560
O	-2.452483	-1.470747	-0.000139
O	-2.451195	1.471415	0.000146
O	2.450297	1.471930	0.000146
O	2.453400	-1.470211	-0.000151

Table S6: Energies of calculated structures on the M06/def2TZVP level of theory. See Figures for the respective structures.

Ion	Isomer	Singlet/Douplet	Triplet/Quartet	Pentet/Hextet
$[MoO_4]^-$	I	0.00	3.13	-
	II	3.39	3.93	-
$[Mo_2O_8]^{2-}$	I	0.00	2.01	-
	II	1.94	1.47	-
	III	1.94	1.95	-
$[Mo_3O_9]^-$	I	0.00	3.08	-
	II	0.28	3.36	-
$[Mo_2O_6]$	I	0.00	2.59	-
$[Mo_2O_5]^-$	I	0.09	0.00	3.08
$[Mo_2O_6]^-$	I	0.00	3.15	6.47
$[Mo_2O_7]^-$	I	0.00	2.76	5.34
$[Mo_2O_8]^-$	I	0.00	-	-
	II	1.06	2.45	-
	III	2.30	2.29	-
	IV	2.41	2.41	-
$[Mo_4O_{13}]^{2-}$	I	0.00	2.58	-
	II	0.06	3.40	-
	III	0.41	2.99	-
	IV	0.49	3.15	-
	V	2.74	2.69	-
	VI	3.21	3.21	-
$[Mo_2O_5]$	I	0.17	0.00	-
$[MoO_3]$	I	0.00	1.59	-
$[Mo_3O_{10}]^-$	I	0.00	2.71	-
	II	0.07	2.72	-
	III	0.42	2.49	-
	IV	1.24	3.52	-
$[Mo_2O_7]$	I	0.00	0.00	-
$[Mo_3O_{10}]^{2-}$	I	0.00	3.14	-
	II	1.26	3.68	-
	III	4.99	3.04	-
	IV	4.13	4.26	-
$[Mo_3O_9]$	I	0.00	2.96	-
	II	1.27	3.43	-

$[Mo_2O_7]^{2-}$	I	0.00	3.33	6.31
$[MoO_3]$	I	0.00	2.92	-
$[Mo_2O_6]^{2-}$	I	0.00	0.31	-

Calculated structures on the B3LYP/def2TZVP level of theory

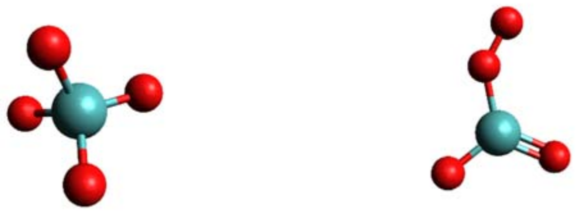

I E=0.00 II E=3.39

Figure S5.1: Calculated Molecules of $[MoO_4]^-$ in lowest multiplicity unless marked with *.

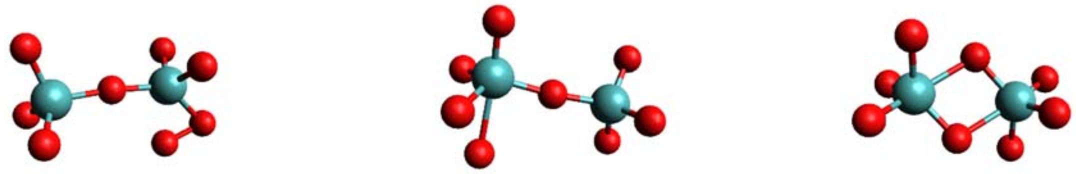

I E=0.00 II* E=1.47 III E=1.94

Figure S5.2: Calculated Molecules of $[Mo_2O_8]^{2-}$ in lowest multiplicity unless marked with *.

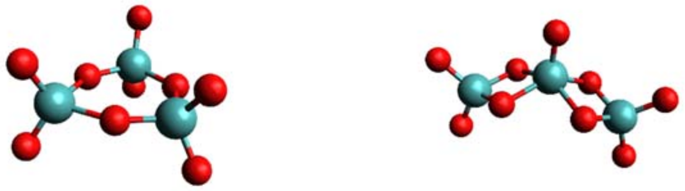

I E=0.00 II E=0.28

Figure S5.3: Calculated Molecules of $[Mo_3O_9]^-$ in lowest multiplicity unless marked with *.

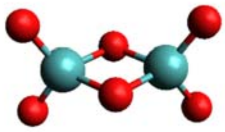

I E=0.00

Figure S5.4: Calculated Molecules of $[Mo_2O_6]$ in lowest multiplicity unless marked with *.

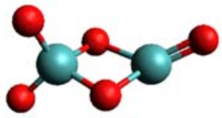

I* E=0.00

Figure S5.5: Calculated Molecules of $[Mo_2O_5]^-$ in lowest multiplicity unless marked with *.

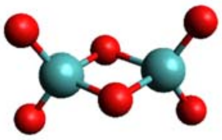

I E=0.00

Figure S5.6: Calculated Molecules of $[Mo_2O_6]^-$ in lowest multiplicity unless marked with *.

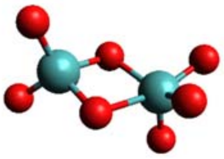

I E=0.00

Figure S5.7: Calculated Molecules of $[Mo_2O_7]^-$ in lowest multiplicity unless marked with *.

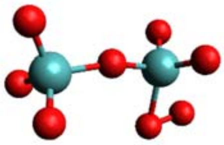

I E=0.00

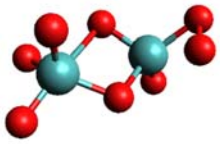

II E=1.06

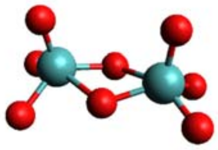

III* E=2.29

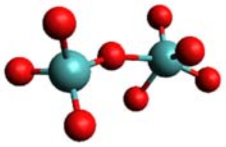

IV* E=2.41

Figure S5.8: Calculated Molecules of $[Mo_2O_8]^-$ in lowest multiplicity unless marked with *.

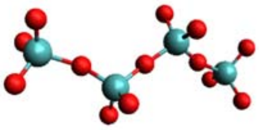

I E=0.00

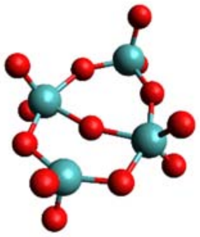

II E=0.06

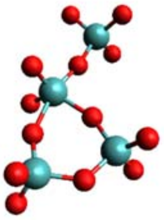

III E=0.41

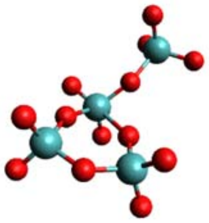

IV E=0.49

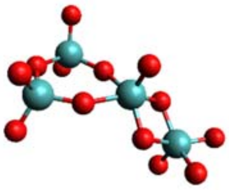

V* E=2.69

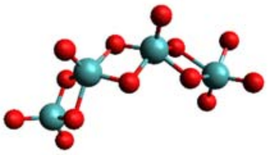

VI E=3.21

Figure S5.9: Calculated Molecules of $[Mo_4O_{13}]^{2-}$ in lowest multiplicity unless marked with *.

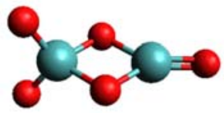

I* E=0.00

Figure S5.10: Calculated Molecules of [Mo_2O_5] in lowest multiplicity unless marked with *.

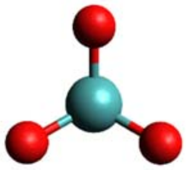

I E=0.00

Figure S5.11: Calculated Molecules of [MoO_3] in lowest multiplicity unless marked with *.

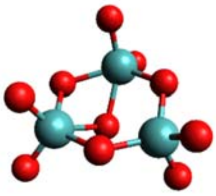

I E=0.00

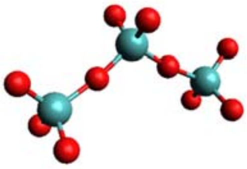

II E=0.07

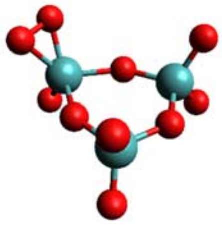

III E=0.42

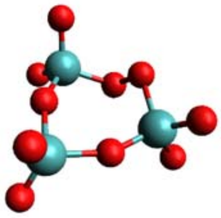

IV E=1.24

Figure S5.12: Calculated Molecules of [Mo_3O_{10}]$^-$ in lowest multiplicity unless marked with *.

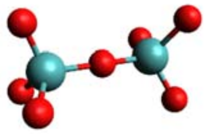

I E=0.00

Figure S5.13: Calculated Molecules of $[Mo_2O_7]$ in lowest multiplicity unless marked with *.

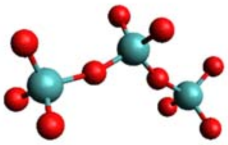

I E=0.00

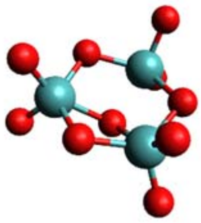

II E=1.26

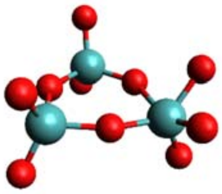

III* E=3.04

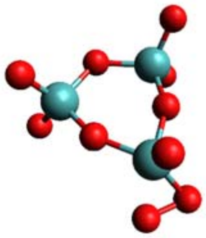

IV E=4.13

Figure S5.14: Calculated Molecules of $[Mo_3O_{10}]^{2-}$ in lowest multiplicity unless marked with *.

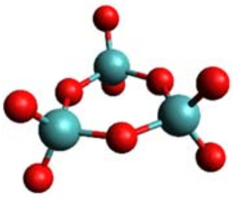

I E=0.00

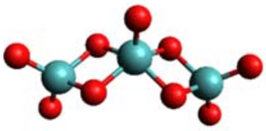

II E=1.27

Figure S5.15: Calculated Molecules of $[Mo_3O_9]$ in lowest multiplicity unless marked with *.

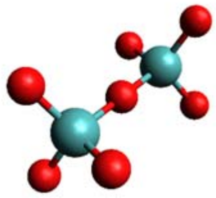

I E=0.00

Figure S5.16: Calculated Molecules of $[Mo_2O_7]^{2-}$ in lowest multiplicity unless marked with *.

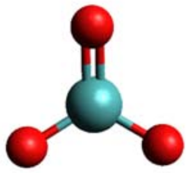

I E=0.00

Figure S5.17: Calculated Molecules of $[MoO_3]^{-}$ in lowest multiplicity unless marked with *.

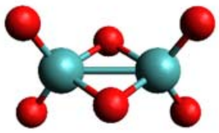

I E=0.00

Figure S5.18: Calculated Molecules of $[Mo_2O_6]^{2-}$ in lowest multiplicity unless marked with *.

Cartesian coordinates (in Ångstrom) of species optimized at the B3LYP/def2TZVP level of theory along the respective ZPE-corrected energy (in eV)

$[MoO_4]^-$ I 1/2

E = -10052.75

Mo	-0.040749	-0.000232	0.005774
O	1.478256	0.043220	-1.123659
O	-0.998610	-1.411311	-0.312938
O	0.722564	-0.070159	1.601309
O	-0.988277	1.439470	-0.195027

$[MoO_4]^-$ I 3/4

E = -10049.62

Mo	-0.170746	-0.000002	0.000289
O	1.509296	0.000031	-1.014215
O	1.510971	0.000801	1.013028
O	-1.062639	1.491113	-0.000427
O	-1.061212	-1.491932	0.000096

$[MoO_4]^-$ II 1/2

E = -10049.37

Mo	0.331311	0.006078	0.000635
O	0.927628	1.632169	-0.001112
O	1.463206	-1.305610	-0.001499
O	-1.450052	-0.467431	0.000144
O	-2.680167	0.108963	-0.000868

$[MoO_4]^-$ II 3/4

E = -10048.83

Mo	0.346587	0.000017	-0.049118
O	1.131424	1.537351	0.162892
O	1.135313	-1.535580	0.162992
O	-1.659016	-0.000752	-0.579304
O	-2.427303	-0.001108	0.511287

$[Mo_2O_8]^{2-}$ I 1/2

E = -20105.11

Mo	2.031274	0.022829	-0.000013
Mo	-1.780135	-0.189791	-0.000025
O	-1.780150	1.781855	-0.000165
O	0.129740	-0.167417	-0.000347
O	-2.196963	-1.080731	-1.417659
O	-2.196472	-1.080601	1.417836
O	2.553554	0.886886	-1.417565
O	2.762861	-1.557596	0.000014
O	-3.144003	1.207469	0.000203
O	2.552950	0.886686	1.417879

$[Mo_2O_8]^{2-}$ I 3/4

E = -20103.10

Mo	-2.004226	-0.004246	-0.000004
Mo	1.806024	-0.277400	0.000001
O	2.091696	1.813541	0.666652
O	-0.106623	0.026172	0.000196
O	2.377850	-1.065336	1.436676
O	2.378115	-1.063581	-1.437538
O	-2.609252	0.803633	1.422416
O	-2.574122	-1.653052	0.000111
O	2.091702	1.814089	-0.665627
O	-2.608809	0.803176	-1.422866

$[Mo_2O_8]^{2-}$ II 1/2

E = -20103.17

Mo	-1.571973	-0.096751	0.011829
Mo	1.571616	0.096555	-0.011884
O	0.151504	0.818905	0.921742
O	2.065120	-1.747336	-0.341740
O	-0.151606	-0.819802	-0.920865
O	-2.061709	1.748452	0.339980
O	-2.062810	-1.140610	1.296038
O	2.869933	0.115042	1.161436
O	-2.870268	-0.115702	-1.161300
O	2.061709	1.142079	-1.294999

$[Mo_2O_8]^{2-}$ II 3/4

E = -20103.65

Mo	1.794501	-0.187667	-0.020158
Mo	-2.013515	0.004492	0.006444
O	-2.593850	0.122680	1.642239
O	-2.616350	1.355071	-0.908206
O	1.763639	2.126347	-0.069065
O	2.039940	-1.802528	0.545035
O	2.792548	0.755153	1.179163
O	-2.610235	-1.466503	-0.704153
O	2.469855	-0.133695	-1.611232
O	-0.095721	0.005142	-0.001783

$[Mo_2O_8]^{2-}$ III 1/2

E = -20103.17

Mo	1.571856	0.096801	-0.011912
Mo	-1.571886	-0.096755	0.011794
O	-0.151552	-0.819821	-0.920917
O	-2.869933	-0.115554	-1.161634
O	-2.062300	-1.141565	1.295368
O	2.062972	1.140614	-1.295990
O	2.064560	-1.747709	-0.339685
O	-2.064912	1.747659	0.341375
O	2.869885	0.115792	1.161758
O	0.151436	0.820343	0.920344

$[Mo_2O_8]^{2-}$ III 3/4

E = -20103.17

Mo	1.571337	0.097187	-0.011551
Mo	-1.571362	-0.097305	0.011559
O	-0.151724	-0.814339	-0.925869
O	-2.870857	-0.109124	-1.160614
O	-2.061534	-1.149294	1.289378
O	2.059166	1.148767	-1.290505
O	2.068347	-1.745651	-0.346635
O	-2.066994	1.745764	0.348195
O	2.872031	0.111284	1.159383
O	0.151699	0.813214	0.926629

$[Mo_3O_9]^-$ I 1/2

E = -24017.29

Mo	-2.179894	0.000601	0.000022
O	-0.727768	1.363180	0.002474
O	-0.728225	-1.361949	-0.001280
O	-3.097569	-0.001480	1.444832
O	-3.096566	0.001776	-1.445430
Mo	1.063465	1.726685	0.000167
O	1.514755	2.602114	-1.390578
O	1.517661	2.603971	1.388780
O	1.870243	-0.000515	0.000425
Mo	1.062639	-1.727197	-0.000117
O	1.514112	-2.603731	1.389891
O	1.515753	-2.603840	-1.389493

$[Mo_3O_9]^-$ I 3/4

E = -24014.21

Mo	-1.297109	-1.580502	0.039374
O	-1.835768	0.235508	-0.135120
O	0.520075	-1.514991	-0.144298
O	-1.730250	-2.174359	1.579314
O	-2.002942	-2.563078	-1.161075
Mo	-0.814188	1.845717	-0.072620
O	-1.126900	2.821299	-1.436721
O	-1.178080	2.732391	1.338066
O	0.906628	1.257370	-0.071076
Mo	2.149902	-0.361342	-0.043321
O	2.884374	0.503543	1.580685
O	3.360191	-0.793017	-1.147797

$[Mo_3O_9]^-$ II 1/2

E = -24017.00

Mo	-0.000085	-0.741549	-0.000117
Mo	-2.668270	0.366414	0.000054
O	-1.403917	-0.078797	1.293732
O	-1.401244	-0.074175	-1.293266
O	-0.000203	-2.412620	0.000222
O	-3.129711	2.012666	0.002771
O	-4.058096	-0.630215	-0.003091
O	1.404290	-0.079798	-1.293927
O	1.400926	-0.073363	1.293110
Mo	2.668328	0.366362	-0.000003
O	4.058191	-0.630215	0.004357
O	3.129909	2.012580	-0.003562

$[Mo_3O_9]^-$ II 3/4

E = -24013.93

Mo	0.042003	-0.815185	0.000046
Mo	-2.722926	0.374346	-0.000044
O	-1.385188	-0.145597	1.222166
O	-1.384920	-0.145181	-1.222158
O	0.050217	-2.493355	0.000035
O	-3.072653	2.270942	0.000327
O	-4.075934	-0.662498	-0.000268
O	1.423191	-0.085794	-1.300911
O	1.422940	-0.086180	1.300712
Mo	2.658723	0.404143	-0.000008
O	4.092792	-0.532384	-0.000071
O	3.046107	2.072703	0.000204

$[Mo_2O_6]$ I 1/2

E = -16008.03

Mo	-1.437950	-0.000078	-0.000017
Mo	1.437927	-0.000175	0.000018
O	0.000014	-0.000591	-1.268554
O	-0.000099	-0.000811	1.268556
O	-2.406212	-1.379387	-0.000038
O	-2.406183	1.379271	0.000060
O	2.403969	1.380641	0.000039
O	2.408633	-1.377796	-0.000066

$[Mo_2O_6]$ I 3/4

E = -16005.44

Mo	-1.419521	0.017385	-0.000020
Mo	1.427781	-0.033487	0.000014
O	-0.001192	0.030483	-1.294340
O	-0.001294	0.030955	1.294318
O	-2.378981	-1.372754	0.000296
O	-2.396845	1.396183	-0.000242
O	2.520153	1.513212	0.000044
O	2.214795	-1.513547	-0.000047

$[Mo_2O_5]^-$ I 1/2

E = -13954.56

Mo	-1.599492	0.008290	0.001116
Mo	1.316033	-0.023243	0.002923
O	0.004471	1.078742	0.741178
O	-0.025374	-1.065917	-0.770321
O	-3.664216	0.009003	-0.000665
O	2.547572	-1.056039	0.751539
O	2.625707	1.112712	-0.742932

$[Mo_2O_5]^-$ I 3/4
E = -13961.01

Mo	1.506724	-0.097453	0.035629
Mo	-1.329636	0.030114	-0.008135
O	0.209381	-0.540779	-1.225938
O	0.202791	0.340543	1.295167
O	3.154478	0.361491	-0.137242
O	-2.361555	-1.302883	0.329075
O	-2.134808	1.495157	-0.405408

$[Mo_2O_5]^-$ I 5/6
E = -13961.10

Mo	-1.576361	-0.168250	0.000000
Mo	1.302046	0.039209	0.000000
O	-0.000283	-0.154932	1.320971
O	-0.000291	-0.154959	-1.320967
O	-3.099595	0.615481	0.000000
O	2.483308	-1.214503	0.000009
O	2.057014	1.586378	-0.000015

$[Mo_2O_5]^-$ I 7/8
E = -13958.01

Mo	1.543353	-0.000858	0.000003
Mo	-1.332205	0.000379	-0.000001
O	-0.039207	0.001877	1.330533
O	-0.039218	0.001852	-1.330539
O	3.600258	0.002960	-0.000010
O	-2.318910	1.414640	-0.000009
O	-2.311448	-1.418815	0.000014

$[Mo_2O_6]^-$ I 1/2
E = -16002.18

Mo	-1.619785	-0.024493	-0.145261
Mo	1.650232	-0.012430	-0.069327
O	0.033223	-0.605271	-1.102253
O	-0.008842	-0.412622	1.079664
O	-2.727679	-0.956032	1.223714
O	-2.459645	1.321126	-0.773186
O	1.849347	1.619493	1.039343
O	3.153748	-0.772853	-0.340692

$[Mo_2O_6]^-$ I 3/4
E = -16011.49

Mo	1.407107	-0.000065	-0.000066
Mo	-1.437733	-0.000049	0.000264
O	0.068613	-0.000947	1.326711
O	0.067022	-0.000016	-1.326175
O	2.381190	-1.412280	-0.000935
O	2.379479	1.413295	0.000220
O	-2.367249	1.441727	-0.000567
O	-2.368265	-1.441182	-0.000293

$[Mo_2O_6]^-$ I 5/6
E = -16008.34

Mo	1.423978	-0.005283	-0.013685
Mo	-1.446678	-0.076787	0.080261
O	0.138666	-0.507038	1.253058
O	0.089444	0.667138	-1.107510
O	2.270510	-1.327715	-0.711221
O	2.531172	1.177420	0.561113
O	-2.275569	1.699092	-0.214856
O	-2.635044	-1.278032	-0.130109

$[Mo_2O_6]^-$ I 7/8
E = -16005.02

Mo	-1.492657	-0.047773	-0.028688
Mo	1.523186	-0.057136	0.095294
O	-0.057745	0.445136	-1.122285
O	-0.101144	-0.497137	1.172142
O	-2.379233	-1.412635	-0.570278
O	-2.539770	1.481525	0.557863
O	2.177658	1.783978	-0.333661
O	2.739955	-1.250092	-0.053464

$[Mo_2O_7]^-$ I 1/2
E = -18049.62

O	-2.797090	-1.633017	0.333135
O	-2.824804	1.382170	0.109184
Mo	-1.814799	0.032882	-0.130282
O	-0.154262	-0.797586	0.756532
O	2.821586	-0.208291	-1.311261
O	2.737277	-1.345203	0.361995
Mo	1.505562	0.120636	0.101706
O	-0.079347	0.302670	-1.106219
O	1.920132	1.493287	1.006661

$[Mo_2O_7]^-$ I 3/4
E = -18058.10

O	-2.611024	-1.332505	0.214312
O	-2.495757	1.410827	-0.393463
Mo	-1.572446	0.012115	-0.024052
O	-0.375506	0.259456	1.301154
O	2.610705	0.454164	-1.180407
O	1.928265	-1.569528	0.548920
Mo	1.421323	-0.064078	-0.051846
O	-0.133538	-0.296310	-1.222620
O	1.870252	1.346704	1.130571

$[Mo_2O_7]^-$ I 5/6
E = -18055.34

O	-2.710255	-0.548518	-1.198507
O	-1.815074	1.708195	0.168128
Mo	-1.559874	0.042049	-0.051567
O	-2.380958	-0.904946	1.366025
O	2.425598	1.563829	0.220391
O	2.971527	-1.253564	-0.206304
Mo	1.796023	-0.016975	-0.011754
O	0.097610	-0.203429	-1.069392
O	0.171771	-0.493205	1.052092

$[Mo_2O_7]^-$ I 7/8
E = -18052.76

O	-2.932789	-1.353341	0.000710
O	-2.530320	1.520596	-0.001336
Mo	-1.821321	-0.044204	-0.000216
O	-0.144661	-0.279251	1.081314
O	2.747041	-0.947380	-1.011984
O	2.745395	-0.951043	1.009687
Mo	1.498613	0.142782	0.000360
O	-0.143856	-0.281013	-1.080558
O	1.953407	1.773897	0.001409

$[Mo_2O_8]^-$ I 1/2
E = -20104.52

Mo	2.043094	0.023632	0.000002
Mo	-1.733963	-0.251843	-0.000005
O	-1.806173	1.854152	-0.000058
O	0.078970	-0.338460	-0.000126
O	-2.299297	-1.048905	-1.391376
O	-2.299140	-1.048981	1.391388
O	2.432059	0.933213	-1.414465
O	2.889774	-1.479729	-0.000501
O	-3.051285	1.394501	0.000132
O	2.432157	0.932316	1.415022

$[Mo_2O_8]^-$ II 1/2
E = -20103.47

Mo	1.605833	0.111874	-0.042446
Mo	-1.465339	-0.201839	-0.007455
O	0.059206	0.295074	-1.121936
O	-2.967645	0.791380	0.600291
O	-2.080167	-1.627674	-0.708521
O	1.891355	1.509742	0.883115
O	2.909827	-0.038219	-1.149836
O	-2.249813	1.539501	-0.433083
O	2.029262	-1.479415	0.889502
O	-0.329623	-0.518072	1.302447

$[Mo_2O_8]^-$ II 3/4
E = -20102.07

Mo	1.581987	0.123021	-0.008777
Mo	-1.460779	-0.186306	0.009057
O	-0.025701	0.788672	-0.845652
O	-2.536263	1.551036	-0.611857
O	-2.239112	-1.324935	-0.994156
O	1.998267	1.081221	1.332452
O	2.770064	0.404977	-1.221640
O	-2.612212	0.490316	1.220584
O	2.165869	-1.671382	0.184472
O	-0.157258	-0.987661	0.934325

$[Mo_2O_8]^-$ III 1/2
E = -20102.22

Mo	1.692592	0.048084	0.030758
Mo	-1.692369	0.045695	-0.033851
O	-2.657351	1.441813	-0.120545
O	-2.359736	-1.164927	-1.091316
O	2.657141	1.446839	0.006872
O	0.024501	0.470253	1.028685
O	2.359223	-1.073935	1.181006
O	-2.201411	-0.940502	1.476071
O	2.200907	-1.055105	-1.397602
O	-0.024446	0.383225	-1.066931

$[Mo_2O_8]^-$ III 3/4
E = -20102.23

Mo	1.721345	-0.043117	0.027285
Mo	-1.721480	0.043315	0.027272
O	-2.011217	-0.131366	1.692090
O	-2.932007	1.094986	-0.621351
O	2.012834	0.131416	1.691812
O	0.120990	-1.061652	-0.292705
O	2.930559	-1.095467	-0.622617
O	-2.383115	-1.453773	-0.919520
O	2.383797	1.453854	-0.919164
O	-0.121130	1.060965	-0.294968

$[Mo_2O_8]^-$ IV 1/2
E = -20102.11

Mo	1.971052	0.030347	-0.020782
Mo	-1.893098	-0.011255	0.064268
O	-2.370553	-0.164820	1.690092
O	-1.479880	1.782301	-0.420522
O	0.217163	0.041068	0.372421
O	2.813218	-1.537207	0.590069
O	2.825262	1.262055	0.843860
O	-3.346289	0.005275	-0.903872
O	2.254264	0.173232	-1.701566
O	-1.322441	-1.662135	-0.698781

$[Mo_2O_8]^-$ IV 3/4
E = -20102.12

Mo	1.965728	-0.031662	-0.020069
Mo	-1.890301	0.011733	0.063835
O	-1.281245	1.651626	-0.694637
O	-1.487061	-1.787635	-0.407857
O	0.217083	-0.059696	0.397661
O	2.799039	1.545291	0.575914

O	2.228548	-0.180823	-1.703435
O	-3.330910	0.009968	-0.921082
O	2.844085	-1.250091	0.840255
O	-2.385533	0.175984	1.683409

$[Mo_4O_{13}]^{2-}$ I 1/2
E = -34071.74

Mo	-1.531114	-1.092352	0.367516
O	-1.373760	-2.453099	-0.640145
O	-1.611999	-1.648076	1.972884
O	0.000222	0.000055	0.160460
Mo	-4.952395	0.328257	-0.366341
Mo	1.531218	1.092986	0.367121
O	1.612611	1.648481	1.972545
O	1.373060	2.453866	-0.640249
O	3.052583	0.180217	-0.050771
O	-5.968625	-1.013463	0.022867
O	-5.155884	0.754040	-2.027468
O	-5.352539	1.676281	0.636150
O	-3.052418	-0.179072	-0.049531
Mo	4.952315	-0.328781	-0.366290
O	5.351849	-1.674594	0.639402
O	5.155606	-0.758606	-2.026403
O	5.969165	1.013394	0.019727

$[Mo_4O_{13}]^{2-}$ I 3/4
E = -34069.16

Mo	-3.027604	-0.001611	0.035629
O	-3.958498	0.005748	-1.412833
O	-3.947617	0.041652	1.490528
O	-1.684905	-1.431564	0.053035
Mo	-0.112151	2.424608	-0.051347
Mo	-0.125006	-2.413846	-0.028804
O	-0.058827	-3.251299	-1.524834
O	-0.103208	-3.561635	1.248836
O	1.292946	-1.272931	0.133641
O	-0.123803	3.331605	-1.506556
O	1.407465	1.375895	0.022077
O	-0.132661	3.506812	1.279004
O	-1.586746	1.339889	0.008685
Mo	2.863218	0.055490	0.086791
O	3.985366	0.989511	-0.851266
O	3.460969	0.035755	1.679965
O	3.557622	-1.448802	-0.842187

$[Mo_4O_{13}]^{2-}$ II 1/2
E = -34071.68

Mo	0.123321	2.075398	-0.000181
O	-3.907042	0.001132	1.397758
O	-0.037407	3.058179	1.385851
O	-0.035537	3.059095	-1.385784
O	2.018939	1.838804	0.001013
Mo	-2.904578	-0.000023	0.000123
Mo	2.615942	-0.000027	0.000209
O	3.609186	0.000302	-1.394455
O	3.610431	-0.000024	1.394010
O	-1.777141	1.433773	-0.001906
O	0.477979	-0.000068	-0.000228
O	-3.908520	-0.001320	-1.396453
Mo	0.123301	-2.075380	-0.000113
O	-1.776892	-1.433677	0.000943
O	-0.036253	-3.057757	-1.386557
O	2.019129	-1.838726	0.000523
O	-0.036300	-3.059545	1.385086

$[Mo_4O_{13}]^{2-}$ II 3/4
E = -34068.34

Mo	-0.010115	-2.087011	0.269107
O	4.452664	0.141565	0.387037
O	-0.014886	-2.522571	1.918902
O	0.243926	-3.517495	-0.630950
O	-1.857548	-1.885920	-0.164993
Mo	2.825103	0.144471	-0.159540
Mo	-2.527355	-0.073055	-0.263804
O	-3.241930	-0.081080	-1.819445
O	-3.759893	-0.161392	0.922332
O	1.841718	-1.444533	0.061710
O	-0.429960	0.036148	0.043285
O	2.736787	-0.437541	-2.006846
Mo	-0.203047	2.092898	0.217840
O	1.763396	1.540698	0.299200
O	0.098071	3.258184	-0.996770
O	-2.058064	1.803710	-0.088462
O	-0.218360	2.864390	1.741085

$[Mo_4O_{13}]^{2-}$ III 1/2
E = -34071.32

Mo	0.558365	-1.123741	-0.001511
Mo	4.141858	0.104254	0.000823
Mo	-1.552346	2.008775	-0.000407
Mo	-3.357261	-0.910425	0.001044
O	-3.149655	1.005269	0.000587
O	-1.487446	3.004305	1.386315
O	2.236219	-0.373382	-0.001168
O	5.093111	-1.342047	-0.000990
O	-4.200637	-1.435529	1.402332
O	-1.673651	-1.451504	-0.000681
O	-0.231076	0.762437	-0.000235
O	4.507657	1.036096	-1.410917
O	4.505892	1.031452	1.416065
O	-1.488401	3.003021	-1.388102
O	-4.203662	-1.436078	-1.398225
O	0.595846	-2.110203	1.381729
O	0.595070	-2.107864	-1.386446

$[Mo_4O_{13}]^{2-}$ III 3/4
E = -34068.75

Mo	-0.419519	-1.174126	-0.029976
Mo	-4.088849	0.054151	0.040499
Mo	1.505617	2.027272	0.002155
Mo	3.324055	-0.897845	0.010392
O	3.121995	1.014209	0.019893
O	1.453751	3.012946	-1.401822
O	-2.373323	-0.400780	-0.029642
O	-5.092405	-0.888119	-1.031027
O	4.201387	-1.390414	-1.383700
O	1.639823	-1.506108	-0.019321
O	0.196872	0.780743	-0.011915
O	-4.696221	-0.000509	1.647349
O	-4.375756	1.782885	-0.663066
O	1.421495	3.014042	1.404037
O	4.159579	-1.407946	1.423621
O	-0.663224	-2.018095	-1.507795
O	-0.680815	-2.042474	1.432273

$[Mo_4O_{13}]^{2-}$ IV 1/2
E = -34071.25

Mo	-2.863716	-1.220833	-0.552409
Mo	-1.909780	2.012432	0.129621
Mo	0.616624	-0.994554	0.806838
O	-3.154569	0.621174	-0.423884
O	-1.107072	-1.452820	-0.120959
O	-0.424165	1.098004	0.370486
O	-1.745290	3.200490	-1.100403
O	-2.453682	2.758729	1.579354
O	-3.891464	-2.056490	0.528514
O	-3.189168	-1.769041	-2.138537
O	2.075840	-0.124299	0.116377
O	4.125542	-0.192925	-2.104621
Mo	3.941070	0.216888	-0.433488
O	4.326963	1.885996	-0.200804
O	5.008765	-0.753836	0.521078
O	0.429024	-0.674096	2.462277
O	1.132238	-2.614031	0.770671

$[Mo_4O_{13}]^{2-}$ IV 3/4
E = -34068.59

Mo	-2.478002	-1.365306	-0.558723
Mo	-1.732311	1.931350	0.111269
Mo	0.840283	-0.644210	0.985948
O	-2.854233	0.503990	-0.493107
O	-0.821485	-1.493914	0.118437
O	-0.177334	1.139115	0.521171
O	-1.552467	3.105008	-1.124726
O	-2.434968	2.676523	1.488541
O	-3.612571	-2.226740	0.398451
O	-2.566894	-1.933797	-2.173888
O	2.534492	0.477098	1.118530
O	1.754663	-0.712415	-1.077635
Mo	3.234685	0.210728	-0.582048
O	3.472287	1.672640	-1.466200
O	4.698999	-0.705463	-0.619583
O	0.446977	-0.862797	2.612215
O	1.823097	-2.335203	0.926452

$[Mo_4O_{13}]^{2-}$ V 1/2
E = -34069.00

Mo	-2.008116	1.930871	-0.205344
O	2.259474	-1.309030	0.070559
O	-2.255506	-2.267801	-1.810214
O	-2.812686	2.699089	1.108681
O	-2.702612	0.303741	-0.533443
Mo	3.603614	-0.132349	-0.382849
Mo	-2.187142	-1.775035	-0.178251
O	-3.920920	-1.507967	0.572302
O	-2.146087	-3.234950	0.753134
O	-2.133291	2.935416	-1.598222
O	2.495858	1.286278	0.015899
O	4.077254	-0.208418	-2.036039
Mo	0.936092	0.134021	0.754949
O	4.996992	-0.235956	0.625389
O	-0.218036	1.626559	0.229309
O	-0.480165	-1.057060	0.233830
O	1.031370	0.143175	2.429162

$[Mo_4O_{13}]^{2-}$ V 3/4
E = -34069.04

Mo	2.297500	1.773702	0.144680
O	-2.015965	-1.247326	0.003486
O	3.197850	0.106113	0.368984
O	2.370057	2.681488	1.596883
O	3.028692	2.670440	-1.121466
Mo	-3.586509	0.082890	0.399388
Mo	2.472782	-1.662210	0.246557
O	3.322304	-2.569061	-0.936897
O	2.595209	-2.453006	1.763603
O	-3.572851	0.329547	2.093655
O	-2.179026	1.152993	-0.388657
O	-4.476442	-1.574527	0.006446
Mo	-0.782506	-0.120283	-0.817138
O	-4.977625	0.896158	-0.253416
O	0.609301	1.301869	-0.265468
O	0.764210	-1.389974	-0.229348
O	-0.772362	-0.293736	-2.498614

$[Mo_4O_{13}]^{2-}$ VI 1/2
E = -34068.53

Mo	3.735656	0.904513	-0.119601
Mo	1.317942	-1.018647	0.163967
Mo	-1.687659	-0.945620	-0.151975
Mo	-3.828620	0.996941	0.066307
O	2.366454	-0.147338	1.328949
O	2.456143	-0.005991	-1.145617
O	-2.853364	-0.004202	1.276294
O	-2.757143	0.408598	-1.312965
O	-0.090380	-0.561693	-1.141277
O	-0.257133	-1.170945	1.194936
O	1.872053	-2.616359	-0.029732
O	-2.274896	-2.480820	-0.522675
O	-5.473990	0.501929	-0.073684
O	4.858363	0.180704	1.249473
O	3.232276	2.500873	0.225224
O	-3.755906	2.695224	0.337811

O	5.106595	1.029787	-1.169903

$[Mo_4O_{13}]^{2-}$ VI 3/4
E = -34068.53

Mo	-3.732456	-0.904855	-0.118350
Mo	-1.317517	1.020352	0.162161
Mo	1.686622	0.945376	-0.149877
Mo	3.827410	-0.997477	0.065474
O	-2.379475	0.158353	1.324627
O	-2.450866	0.003713	-1.146017
O	2.851804	-0.000411	1.277791
O	2.756269	-0.405042	-1.312974
O	0.090789	0.572134	-1.145806
O	0.253678	1.156222	1.199855
O	-1.862547	2.620703	-0.033414
O	2.275024	2.482356	-0.510635
O	5.472673	-0.501459	-0.072339
O	-4.869478	-0.192930	1.246604
O	-3.227998	-2.500915	0.225630
O	3.755101	-2.696624	0.331920
O	-5.101285	-1.028927	-1.172136

$[Mo_2O_5]$ I 1/2
E = -13958.16

Mo	-1.477639	0.229540	-0.000015
Mo	1.219695	-0.062158	0.000080
O	-0.155346	0.333847	-1.334245
O	-0.155559	0.333723	1.334362
O	-2.623093	-0.988195	-0.000139
O	2.469699	1.083101	-0.000179
O	1.818506	-1.641228	-0.000138

$[Mo_2O_5]$ I 3/4
E = -13958.33

Mo	-1.557725	0.000076	0.000053
Mo	1.330858	0.000096	-0.000040
O	-0.081852	-0.002438	-1.277893
O	-0.081824	0.003314	1.277821
O	-3.238368	0.000016	-0.000123
O	2.298060	1.389095	-0.002582
O	2.295038	-1.390891	0.002706

$[MoO_3]$ I 1/2
E = -8001.71

Mo	-0.000072	-0.000001	-0.196361
O	0.813210	-1.399526	0.343591
O	0.805996	1.403693	0.343590
O	-1.618829	-0.004162	0.343716

$[MoO_3]$ I 3/4
E = -8000.13

Mo	0.021510	-0.000423	-0.138481
O	0.848240	1.428497	0.249889
O	-1.836330	-0.013483	0.226409
O	0.875161	-1.412793	0.250729

$[Mo_3O_{10}]^{-}$ I 1/2
E = -26063.40

Mo	0.931609	1.619691	-0.014010
O	-0.967018	1.594001	0.396982
O	2.132760	-0.000229	-0.171401
O	1.333204	2.852127	-1.104981
O	1.547813	2.062781	1.511642
Mo	-1.938259	0.000234	0.039934
O	-2.638441	-0.000049	-1.505672
O	0.306425	-0.000292	-1.133608
Mo	0.931081	-1.619968	-0.013977
O	-0.967605	-1.593582	0.397715
O	1.547266	-2.062960	1.511704
O	-3.230079	0.001028	1.139697
O	1.332412	-2.852599	-1.104799

$[Mo_3O_{10}]^{-}$ I 3/4
E = -26060.69

Mo	-1.769644	-0.844277	-0.015423
O	-0.000179	-1.782776	0.150295
O	-1.799621	0.993226	0.361369
O	-2.721712	-1.249687	-1.358875
O	-2.501661	-1.585854	1.330899
Mo	1.769457	-0.844555	-0.015473
O	2.721513	-1.250261	-1.358843
O	0.000012	-0.059860	-1.078762
Mo	0.000163	1.733511	-0.065379
O	1.799865	0.992986	0.361229
O	0.000526	2.214537	1.843192
O	2.501232	-1.586195	1.330946
O	0.000151	3.079317	-1.076004

$[Mo_3O_{10}]^{-}$ II 1/2
E = -26063.34

Mo	3.244053	-0.618424	-0.000347
O	1.524390	0.240052	-0.004548
O	-3.459011	-1.551717	-1.456622
O	3.375754	-1.729455	-1.341380
O	3.461116	-1.545524	1.459817
Mo	0.000001	1.305769	-0.000080
O	0.003950	2.306602	1.368512
O	-0.003965	2.307433	-1.368068
O	-1.524401	0.240040	0.003649
Mo	-3.244048	-0.618418	0.000434
O	-4.496124	0.547500	0.108228
O	-3.377744	-1.723792	1.345368
O	4.496003	0.546993	-0.114993

$[Mo_3O_{10}]^{-}$ II 3/4
E = -26060.68

Mo	-2.820302	-0.800465	-0.000190
O	-1.453296	0.406371	-0.000356
O	2.821539	-1.763224	1.417389
O	-2.825113	-1.760570	1.419024
O	-2.821470	-1.764478	-1.416566
Mo	-0.000007	1.735657	-0.000004
O	0.000077	2.659222	-1.439857
O	-0.000548	2.658516	1.440297
O	1.453538	0.406669	-0.000350
Mo	2.820345	-0.800408	0.000236
O	4.484592	0.105701	0.000792
O	2.824945	-1.761649	-1.418267
O	-4.484450	0.105826	-0.002327

$[Mo_3O_{10}]^{-}$ III 1/2
E = -26062.98

Mo	2.056069	-0.002180	-0.199917
O	0.594358	1.376011	-0.129241
O	0.592183	-1.377561	-0.119893
O	2.746820	-0.007013	-1.733117
O	3.168428	0.735260	1.179124
Mo	-1.187990	1.729785	0.024622
O	-1.536399	2.544286	1.479733
O	-1.748941	2.666859	-1.282573
O	-1.988342	0.001860	0.017637
Mo	-1.191598	-1.727693	0.025489
O	-1.748920	-2.661174	-1.285815
O	-1.548040	-2.544204	1.477528
O	3.167330	-0.733859	1.183100

$[Mo_3O_{10}]^{-}$ III 3/4
E = -26060.91

Mo	1.900555	-0.356502	-0.276535
O	1.036165	1.353880	-0.096901
O	0.427157	-1.481204	-0.056674
O	2.522689	-0.498187	-1.831331
O	3.211224	-0.013423	1.332803
Mo	-0.743414	1.978659	0.037047
O	-1.002027	2.717238	1.557795
O	-1.119131	3.066147	-1.226879
O	-1.653989	0.398835	-0.119721
Mo	-1.560913	-1.564771	0.030678
O	-2.221767	-2.376757	-1.327812
O	-2.062392	-2.152993	1.562841
O	2.981872	-1.314815	1.302134

$[Mo_3O_{10}]^{-}$ IV 1/2
E = -26062.16

Mo	1.964624	-0.954518	0.017225
O	1.338008	0.745483	0.156067
O	3.136809	-1.086098	-1.210099
O	2.619787	-1.494441	1.492681
Mo	-0.007905	2.221197	-0.000082
O	-0.176157	3.135163	1.436819
O	0.154965	3.138746	-1.435317
O	-1.345558	0.737854	-0.159124
Mo	-1.957805	-0.967120	-0.017294
O	-2.610094	-1.513850	-1.491504
O	-3.127743	-1.106548	1.211304
O	-0.456215	-2.064678	0.562201
O	0.471898	-2.064319	-0.562231

$[Mo_3O_{10}]^{-}$ IV 3/4
E = -26059.89

Mo	2.374248	-0.860143	0.017093
O	1.385225	0.775827	0.368806
O	3.131344	-0.875397	-1.516282
O	3.364665	-1.302634	1.339408
Mo	0.000441	1.947039	-0.000190
O	-0.333038	2.925374	1.357653
O	0.332115	2.928944	-1.355949
O	-1.382242	0.774131	-0.371130
Mo	-2.374441	-0.859639	-0.017465
O	-3.361409	-1.305831	-1.341146
O	-3.135405	-0.869560	1.514081
O	-0.661457	-2.122410	0.058058
O	0.658895	-2.121545	-0.050550

$[Mo_2O_7]$ I 1/2
E = -18052.53

O	-2.386962	-1.513927	0.572054
O	-2.478623	0.215259	-1.592872
Mo	-1.880993	-0.010541	-0.022484
O	-2.694204	1.289842	1.041488
O	2.386537	-1.512697	-0.575795
O	2.480246	0.211814	1.592775
Mo	1.881025	-0.010705	0.022497
O	0.000008	0.128439	0.001043
O	2.692834	1.292812	-1.038756

$[Mo_2O_7]$ I 3/4
E = -18052.53

O	-2.385401	-1.511142	0.580577
O	-2.480050	0.206395	-1.593448
Mo	-1.880957	-0.010732	-0.022349
O	-2.693672	1.295967	1.034400
O	2.385504	-1.514998	-0.570423
O	2.479462	0.216919	1.592219
Mo	1.880918	-0.010581	0.022361
O	-0.000025	0.129848	-0.000776
O	2.694386	1.288903	-1.042609

$[Mo_3O_{10}]^{2-}$ I 1/2
E = -26066.09

Mo	3.390934	-0.549755	0.000032
O	1.519921	0.059854	0.000758
O	-3.684467	-1.503116	-1.414071
O	3.684339	-1.502902	-1.414220
O	3.684223	-1.506292	1.412024
Mo	-0.000051	1.104950	0.000049
O	-0.000251	2.114982	1.377924
O	-0.000145	2.113400	-1.378975

O	-1.519696	0.059368	0.000646
Mo	-3.390865	-0.549803	0.000028
O	-4.447189	0.821326	0.001461
O	-3.684429	-1.506037	1.412171
O	4.447603	0.821112	0.001707

$[Mo_3O_{10}]^{2-}$ I 3/4
E = -26062.95

Mo	3.336342	-0.676454	-0.000042
O	1.514242	0.100227	0.000652
O	-3.587839	-1.557711	-1.412646
O	3.851042	-1.454961	-1.452963
O	3.851251	-1.457647	1.451415
Mo	0.012629	1.169710	-0.000084
O	0.001075	2.170497	1.387239
O	0.002754	2.170937	-1.387108
O	-1.499668	0.097204	-0.001243
Mo	-3.334375	-0.588256	-0.000015
O	-4.452013	0.735165	-0.001520
O	-3.587437	-1.554198	1.415081
O	3.829964	1.249233	0.001832

$[Mo_3O_{10}]^{2-}$ II 1/2
E = -26064.83

Mo	-1.699092	-0.695099	-0.014163
O	-0.271951	-1.999205	0.280546
O	-1.595561	1.235190	0.280654
O	-2.733689	-1.118345	-1.316436
O	-2.560850	-1.047988	1.437702
Mo	1.451587	-1.123736	-0.014163
O	2.335430	-1.808010	-1.316482
O	0.000024	0.000158	-0.982978
Mo	0.247501	1.818840	-0.014182
O	1.867553	0.764126	0.280738
O	0.372839	2.741176	1.437963
O	2.187936	-1.693884	1.437665
O	0.398294	2.926755	-1.316201

$[Mo_3O_{10}]^{2-}$ II 3/4
E = -26062.40

Mo	-1.626361	-0.878518	-0.015153
O	-0.000111	-1.897810	0.316871
O	-1.781593	1.002041	0.339433
O	-2.509416	-1.389281	-1.394804
O	-2.516055	-1.466524	1.338453
Mo	1.626222	-0.878747	-0.015186
O	2.509153	-1.389655	-1.394861
O	-0.000013	0.094308	-1.068581
Mo	0.000117	1.780014	-0.085262
O	1.781706	1.001841	0.339388
O	0.000350	2.217377	1.866911
O	2.515932	-1.466782	1.338396
O	0.000164	3.175052	-1.074301

$[Mo_3O_{10}]^{2-}$ III 1/2
E = -26061.09

Mo	1.806403	-0.765869	0.017742
O	1.786234	1.105743	0.166118
O	2.886789	-1.064889	-1.290016
O	2.436853	-1.307058	1.522262
Mo	-0.002891	1.817408	-0.000079
O	-0.135724	2.737835	1.454080
O	0.127739	2.740950	-1.452646
O	-1.790012	1.101503	-0.167292
Mo	-1.804100	-0.770246	-0.017820
O	-2.433946	-1.313838	-1.521752
O	-2.881906	-1.072155	1.291378
O	-0.539448	-2.202942	0.476408
O	0.546508	-2.201943	-0.477716

$[Mo_3O_{10}]^{2-}$ III 3/4
E = -26063.05

Mo	2.099150	0.205630	0.069601
O	0.513068	1.220592	0.068264
O	3.195818	1.355219	-0.680424
O	2.673516	-0.009046	1.666591
Mo	-1.355163	1.726215	-0.022987
O	-1.880570	2.572807	1.389937
O	-1.776969	2.477075	-1.521758
O	-1.894682	-0.184386	0.009048
Mo	-1.028249	-1.831815	-0.000430
O	-1.438884	-2.699713	-1.436732
O	-1.515677	-2.765426	1.368945
O	0.734968	-1.486026	0.058858
O	2.881792	-1.006252	-1.165195

$[Mo_3O_{10}]^{2-}$ IV 1/2
E = -26061.96

Mo	2.085181	-0.000090	-0.207604
O	0.554939	1.275809	-0.136631
O	0.555222	-1.275925	-0.134556
O	2.735123	-0.000915	-1.780280
O	3.215007	0.737589	1.208761
Mo	-1.201191	1.705644	0.026531
O	-1.523728	2.487760	1.534275
O	-1.728604	2.656636	-1.317349
O	-2.106415	0.000285	0.010526
Mo	-1.201086	-1.705760	0.026734
O	-1.727382	-2.656404	-1.317794
O	-1.524781	-2.487946	1.534159
O	3.215374	-0.735804	1.209173

$[Mo_3O_{10}]^{2-}$ IV 3/4
E = -26061.82

Mo	1.990355	-0.314602	-0.210072
O	0.887414	1.372465	-0.217577
O	0.413263	-1.425956	0.016482
O	2.638660	-0.566142	-1.763768
O	3.025200	-1.155371	1.211814
Mo	-0.835640	1.936629	0.027798
O	-0.995130	2.871062	1.471105
O	-1.371459	2.868027	-1.324761
O	-1.776272	0.370363	0.165829
Mo	-1.522579	-1.609976	0.045082
O	-2.121366	-2.248109	-1.450395
O	-2.026813	-2.451812	1.474336
O	3.257792	0.302209	1.137192

$[Mo_3O_9]$ I 1/2
E = -24013.84

Mo	1.684567	1.148680	-0.000070
O	1.541583	-0.742259	-0.000862
O	-0.128028	1.706053	-0.000200
O	2.503727	1.707069	1.358646
O	2.504609	1.708147	-1.357800
Mo	0.152529	-2.033172	-0.000079
O	0.226751	-3.023434	-1.357556
O	0.226798	-3.021338	1.358921
O	-1.413627	-0.963967	-0.000775
Mo	-1.837091	0.884495	-0.000028
O	-2.730607	1.314575	1.358503
O	-2.731237	1.315142	-1.357945

$[Mo_3O_9]$ I 3/4
E = -24010.88

Mo	1.040168	1.740671	-0.010076
O	1.808582	0.006476	-0.006118
O	-0.812261	1.378561	-0.082024
O	1.441942	2.596068	1.386035
O	1.528897	2.639625	-1.350398
Mo	1.053231	-1.733500	-0.009677
O	1.545357	-2.626971	-1.352400
O	1.464268	-2.587844	1.384385
O	-0.801405	-1.383027	-0.078332
Mo	-2.113340	-0.007306	0.030981
O	-2.843363	-0.008926	1.540681
O	-3.227329	-0.013252	-1.500778

$[Mo_3O_9]$ II 1/2
E = -24012.57

Mo	-0.000005	-0.731127	-0.000335
Mo	-2.699817	0.373422	0.000067
O	-1.361674	-0.155410	1.242263
O	-1.363530	-0.159304	-1.242516
O	0.001003	-2.396658	0.000754
O	-3.083203	2.015946	-0.002127
O	-4.114333	-0.544548	0.002599
O	1.361561	-0.155830	-1.242536
O	1.362570	-0.156607	1.242348
Mo	2.699637	0.373698	0.000022
O	4.112320	-0.547057	-0.000424
O	3.086251	2.015504	0.000930

$[Mo_3O_9]$ II 3/4
E = -24010.41

Mo	-0.045083	-0.775083	-0.000040
Mo	-2.674879	0.391602	-0.000008
O	-1.362011	-0.109779	1.275766
O	-1.362305	-0.110524	-1.275883
O	-0.036202	-2.433499	0.000357
O	-3.069287	2.037758	-0.000428
O	-4.085011	-0.542799	0.000479
O	1.490873	-0.063303	-1.301827
O	1.490856	-0.062601	1.301860
Mo	2.645478	0.342606	-0.000046
O	4.070578	-0.592459	0.000105
O	3.253553	2.091797	0.000060

$[Mo_2O_7]^{2-}$ I 1/2
E = -18059.79

Mo	-1.910556	-0.000009	-0.000005
Mo	1.910556	0.000004	-0.000010
O	0.000002	-0.000067	0.000022
O	2.503478	1.633053	-0.108827
O	2.503535	-0.722241	1.468673
O	-2.503570	-1.632901	0.110634
O	-2.503516	0.912287	1.358811
O	2.503674	-0.910753	-1.359797
O	-2.503602	0.720650	-1.469442

$[Mo_2O_7]^{2-}$ I 3/4
E = -18056.45

Mo	-1.871176	-0.107663	0.000003
Mo	1.953175	0.019484	-0.000002
O	0.039674	0.026878	0.000047
O	2.556578	1.652215	-0.000866
O	2.536606	-0.801810	1.420483
O	-2.567831	-1.692327	0.000043
O	-2.765955	1.040671	1.152663
O	2.536513	-0.803264	-1.419685
O	-2.766078	1.040577	-1.152690

$[Mo_2O_7]^{2-}$ I 5/6
E = -18053.48

Mo	-1.904326	0.199832	0.046189
Mo	1.935768	-0.014640	0.016410
O	0.047037	0.090715	0.032232
O	2.455192	-1.569871	0.612056
O	2.536210	0.212793	-1.608003
O	-2.588907	1.743904	-0.338186
O	-2.121125	-1.596733	-0.948048
O	2.590943	1.240129	1.041108
O	-3.084418	-1.093191	0.880197

$[Mo_2O_7]^{2-}$ I 7/8
E = -18050.13

Mo	-1.889394	0.160408	0.053593
Mo	1.989463	-0.060644	0.039226
O	0.058598	-0.006964	-0.027136

O	2.695718	-0.010565	1.635145
O	2.775824	-1.144058	-1.081241
O	-2.614716	1.701129	-0.273592
O	-2.115072	-1.636711	-0.951244
O	1.675846	1.739522	-0.722140
O	-3.001562	-1.166116	0.932907

$[MoO_3]^-$ I 1/2
E = -8004.93

Mo	0.000055	-0.000010	-0.123755
O	-1.054359	-1.346055	0.216553
O	1.692946	-0.239827	0.216611
O	-0.638875	1.585932	0.216551

$[MoO_3]^-$ I 3/4
E = -8002.00

Mo	0.060361	0.000113	0.000178
O	0.816070	1.577822	-0.000338
O	-1.973516	-0.013891	-0.000255
O	0.840550	-1.564526	-0.000339

$[Mo_2O_6]^{2-}$ I 1/2
E = -16009.46

Mo	-1.318334	0.000025	-0.000007
Mo	1.318373	0.000001	0.000008
O	-0.000025	0.000057	-1.455472
O	-0.000042	-0.000015	1.455474
O	-2.234159	-1.494388	-0.000007
O	-2.233870	1.494602	0.000023
O	2.234309	1.494374	0.000010
O	2.233584	-1.494765	-0.000030

$[Mo_2O_6]^{2-}$ I 3/4
E = -16009.15

Mo	-1.534406	-0.000067	-0.000005
Mo	1.534402	-0.000054	0.000005
O	0.000010	-0.000352	-1.228029
O	-0.000023	-0.000977	1.228029
O	-2.461322	-1.486055	-0.000122
O	-2.460399	1.486499	0.000130
O	2.459494	1.487015	0.000129
O	2.462265	-1.485498	-0.000136
